# Discovery and Structure–Activity Relationships of 2,5-Dimethoxyphenylpiperidines as Selective Serotonin 5-HT_2A_ Receptor Agonists

**DOI:** 10.1021/acs.jmedchem.4c00082

**Published:** 2024-04-22

**Authors:** Emil M. Ro̷rsted, Anders A. Jensen, Gints Smits, Karla Frydenvang, Jesper L. Kristensen

**Affiliations:** † Lophora, Charlottenlund, Copenhagen 2920, Denmark; ‡ Department of Drug Design and Pharmacology, University of Copenhagen, Universitetsparken 2, Copenhagen Ø 2100, Denmark; § Latvian Institute of Organic Synthesis, Aizkraukles 21, Riga 1006, Latvia

## Abstract

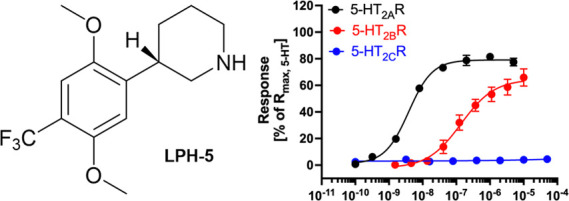

Classical psychedelics such as psilocybin, lysergic acid diethylamide (LSD), and *N,N*-dimethyltryptamine (DMT) are showing promising results in clinical trials for a range of psychiatric indications, including depression, anxiety, and substance abuse disorder. These compounds are characterized by broad pharmacological activity profiles, and while the acute mind-altering effects can be ascribed to their shared agonist activity at the serotonin 2A receptor (5-HT_2A_R), their apparent persistent therapeutic effects are yet to be decidedly linked to activity at this receptor. We report herein the discovery of 2,5-dimethoxyphenylpiperidines as a novel class of selective 5-HT_2A_R agonists and detail the structure–activity investigations leading to the identification of LPH-5 [analogue (*S*)-**11**] as a selective 5-HT_2A_R agonist with desirable drug-like properties.

## Introduction

Serotonin is involved in a multitude of functions in the central nervous system (CNS) as well as in the periphery, where a total of 14 different receptor subtypes mediate the effects of the neurotransmitter.^1,2^ Agonists of 5-HT_2A_R such as psilocybin, LSD, and DMT are often referred to as psychedelics due to their perturbation of perception and state of mind.^3,4^ Several of these compounds are presently receiving substantial attention as possible therapeutics for the treatment of a range of psychiatric disorders.^5−9^ For example, psilocybin has shown promising effects in the treatment of depression, obsessive-compulsive disorder, and substance use disorder.^10−13^

In addition to their 5-HT_2A_R agonist activity, classical psychedelics target numerous other serotonin receptor subtypes and in some cases other monoaminergic receptors as well.^14,15^ While their shared 5-HT_2A_R agonism is believed to be responsible for their acute psychedelic effects, the persistent therapeutic benefits following a single administration of these drug are yet to be causally linked to activation of 5-HT_2A_R.^3,4^ Thus, it remains an open question whether the broad activity profiles of the classical psychedelics are required for therapeutic efficacy. This conundrum has in recent years spawned significant research efforts aimed at developing selective 5-HT_2A_R agonists to probe their possible therapeutic applications.^16^

**Figure 1:**
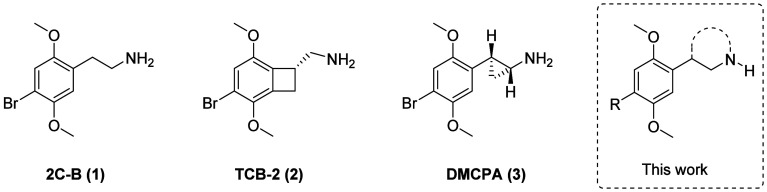
Structures of 2C-B (**1**), two of its previously reported conformationally restricted analogues, TCB-2 (**2**) and DMCPA (**3**), and the ligands investigated in this study.

Research into new ligands selectively targeting the 5-HT_2A_R has taken many avenues. Historically, modifications of the phenethylamine scaffold derived from mescaline, an alkaloid found in *Peyote* cacti, have led to the development of numerous potent 5-HT_2A_R agonists and provided detailed information about their structure–activity relationships.^17−29^ Overall, decorating the 2,5-dimethoxyphenethylamine scaffold (often referred to as “2C-X’s”) with a lipophilic substituent in the 4′-position usually confers increased agonist potency at 5-HT_2_ receptors, including the 5-HT_2A_R, as seen, for example, in 2-(4-bromo-2,5-dimethoxyphenyl)ethan-1-amine (2C-B) (**1**) (Figure 1). The *N*-benzyl phenethylamine (NBOMe) class of compounds derived from N-benzylation of the 2C-X’s include some of the most potent 5-HT_2A_R agonists reported to date. Some of these also exhibit pronounced selectivity for 5-HT_2A_R over the two other 5-HT_2_R subtypes, 5-HT_2B_R and 5-HT_2C_R.^30,31^ However, concerns about their safety profiles may hinder the clinical development of compounds from the NBOMe class.^32^

Representatives of the NBOMe class have been used as tool compounds to investigate the hypothesis that biased agonists mediating selective activation of specific 5-HT_2A_R-coupled downstream pathways may hold therapeutic advantages compared to nonbiased receptor agonists. These investigations have led too the development of biased agonists with promising *in vitro* profiles, but their clinical potential is yet to be investigated.^31,33^

Investigations into receptor–ligand interactions of phenethylamines have guided the design of new ligands and provided information about the importance of the ethylamine chain conformation in relation to their 5-HT_2A_R activity.^15,29^ The functional properties of conformationally restricted 2C-X analogues like (*R*)-(3-bromo-2,5-dimethoxybicyclo[4.2.0]octa-1,3,5-trien-7-yl)methanamine (TCB-2) (**2**) and (1*R*,2*S*)-2-(4-bromo-2,5-dimethoxyphenyl)cyclopropan-1-amine (DMPCA) (**3**) (Figure 1) show that agonist potency at 5-HT_2A_R is very dependent on the spatial orientation of the ethylamine chain,^29,34−36^ with bioactivity typically residing primarily in a single enantiomer of such conformationally restrained compounds, as is also the case for 4-substituted 2,5-dimethoxy amphetamines.^23,35,37^ Several potent 5-HT_2A_R agonists have emerged from these efforts, whereas selectivity for the 5-HT_2A_R toward the other 5-HT_2_Rs remains an unresolved challenge.

In the present work, we were inspired by previous work on restricted phenethylamine structures to investigate the effects of introducing related conformational restraint on the 2C-X scaffold via a bridge between the benzylic position and the nitrogen atom (illustrated in Figure 1).^38^

## Results and Discussion

**Figure 2:**
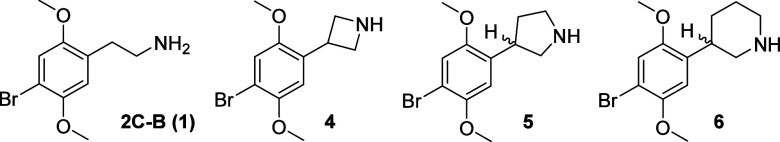
2C-B (1) and its 4-, 5-, and 6-membered constrained analogues.

Initially, we targeted the 4-, 5-, and 6-membered congeners of 2C-B (**1**) (Figure 2) seeking to investigate the effects of restraining the ethylamino side chain at various bond angles, thus also probing the importance of the spatial orientation of the secondary amine. The racemate of phenylpiperidine **6** was reported by Nichols and coworkers in 2013 as an intermediate in the synthesis of a series of structurally constrained NBOMes, but the authors did not report any pharmacological data for this compound.^39^

Given the close structural similarity between the orthosteric sites in 5-HT_2A_R and 5-HT_2C_R,^40^ we chose to characterize the functional properties of the analogues at these two receptor subtypes in a fluorescence-based Ca^2+^ imaging assay in our development of the structure–activity relationships in the pursuit of selective 5-HT_2A_R agonists.^41,42^

**Figure 3:**
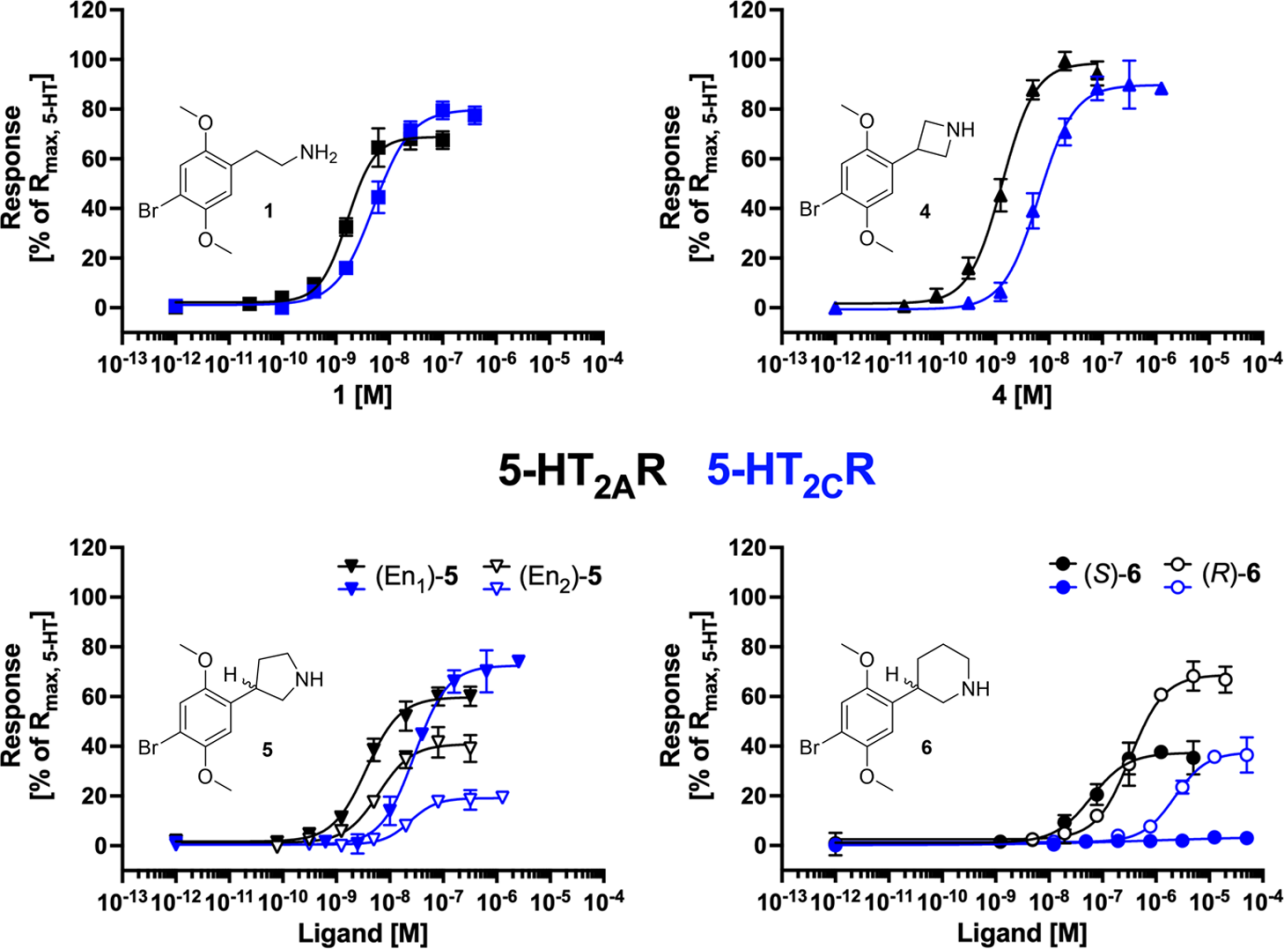
Functional properties exhibited by **1**, **4**, **5**, and **6** at stable 5-HT_2A_R- and 5-HT_2C_R-HEK293 cell lines in a Ca^2+^/Fluo-4 assay. Data are given as mean ± standard deviation (S.D.) values and are from representative experiments performed in duplicate out of at least 3 independent experiments, see Tables 1 and S2.

**Figure 4:**
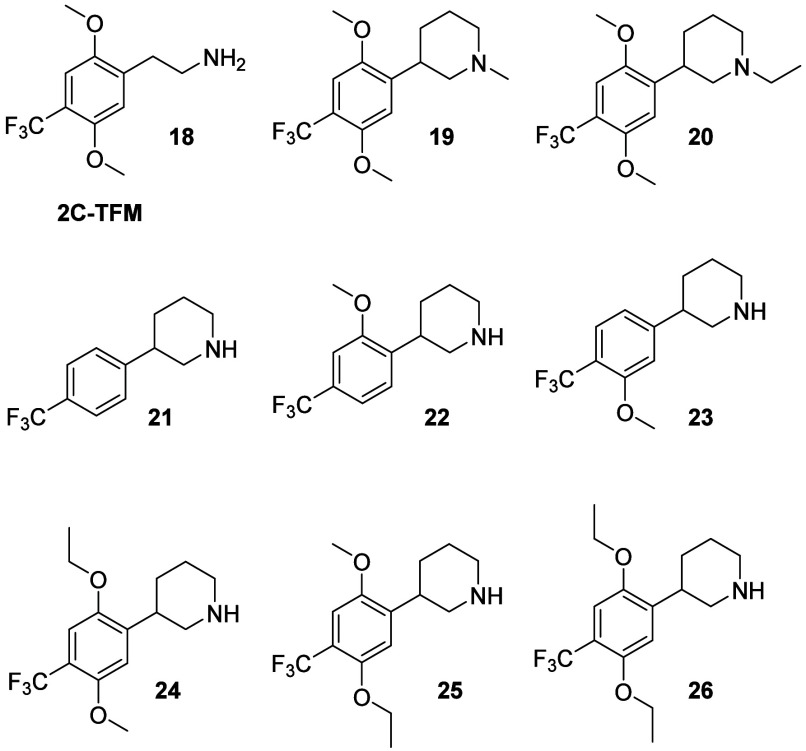
2C-TFM (**18**), the *N*-alkylated analogues **19** and **20**, and analogues **21**–**26** with modifications to the 2′ and/or 5′-position substituents.

We found **4** to be a potent albeit unselective full agonist at 5-HT_2A_R and 5-HT_2C_R (EC_50_ = 1.6 and 5.8 nM, respectively) (Figure 3 and Table 1). This profile was very similar to that of 2C-B (**1**) with EC_50_ values of 1.6 and 4.1 nM at 5-HT_2A_R and 5-HT_2C_R, respectively. The two enantiomers of **5**, **5**_eu_, and **5**_dis_, were both potent and unselective high-efficacious partial agonists at 5-HT_2A_R (EC_50_ = 5.3 and 7.7 nM) and also potent agonists at 5-HT_2C_R (EC_50_ = 26 and 18 nM), but notably displayed different efficacies at this receptor (*R*_max_ = 16% and 73%) (Figure 3). The two enantiomers of the 6-membered analogue **6** displayed decreased agonist potencies at 5-HT_2A_R compared to **4** and **5**, but interestingly, the trend of differential efficacies at 5-HT_2A_R and 5-HT_2C_R observed for the enantiomers of **5** was even more pronounced for **6**. Eutomer **6**_eu_ displayed partial agonism (*R*_max_ = 37%) and an EC_50_ of 69 nM on 5-HT_2A_R, while being devoid of measurable agonist activity at the 5-HT_2C_R (Figure 3 and 4, Table 1). When tested as an antagonist at 5-HT_2C_R, **6**_eu_ mediated concentration-dependent inhibition of the 5-HT EC_90_-induced response through the receptor, with an IC_50_ value of 640 nM. In comparison, distomer **6**_dis_ displayed moderately potent partial agonism at both 5-HT_2A_R (EC_50_ = 370 nM) and 5-HT_2C_R (EC_50_ = 1,900 nM).

**Table 1:**
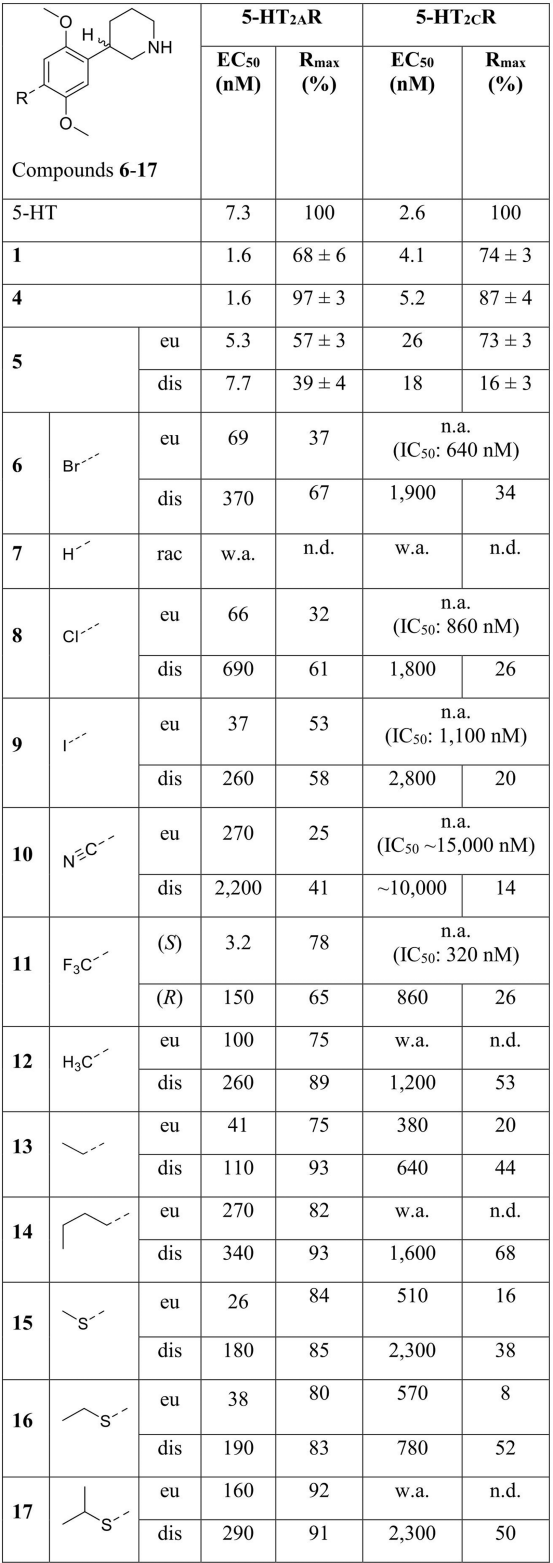
Functional Properties of 1, 4-17 at 5-HT_2A_R and 5-HT_2C_R^a^

a Functional properties exhibited by **4**–**17** at stable 5-HT_2A_R- and 5-HT_2C_R-HEK293 cell lines in the Ca^2+^/Fluo-4 assay. eu: eutomer, dis: distomer. EC_50_ values are given in nM, and *R*_max_ values are given as % of the 5-HT *R*_max_. For the compounds also tested in antagonist mode at 5-HT_2C_R (using 5-HT EC_90_ as an agonist), IC_50_ values are given in nM. All data are based on at least 3 independent experiments. n.a.: no agonist activity: the compound displayed no significant agonist activity or negligible levels of agonist activity at 5-HT_2C_R at concentrations up to 50 μM. w.a.: weak agonist activity: the compound only elicited significant agonist responses at micromolar concentrations, so a complete concentration–response curve could not be obtained. n.d.: not determinable: the *R*_max_ value for the compound could not be determined since a complete concentration–response curve was not obtained in the tested concentration range (up to 50 μM). See Table S2 for full details on the data reported in Table 1.

Encouraged by the profile of **6**_eu_, we set out to investigate whether the structure–activity relationships of 2,5-dimethoxyphenethylamines (2C-X family) would translate to the corresponding phenylpiperidine series. In the first series of analogues we probed the effects of various substituents in the 4-position, including other halogens, trifluoromethyl (TFM), nitrile, alkyl (methyl, ethyl, and *n*-butyl), and thioalkyl (methyl-, ethyl-, and isopropylthio) substituents, as several of the corresponding 2C-Xs have been reported to be significantly more potent than their 4-bromo-substituted analogues.^15^ The structures and functional properties of these analogues at 5-HT_2A_R and 5-HT_2C_R in the Ca^2+^/Fluo-4 assay are given in Table 1.

Several interesting observations can be made from the data in Table 1. Overall, there was a marked difference in the agonist potencies displayed by these analogs at 5-HT_2A_R, clearly indicating that the 4-substituent is an important determinant of the activity of the 2,5-dimethoxyphenylpiperidines. Furthermore, the functional 5-HT_2A_R-over-5-HT_2C_R selectivity observed for **6**_eu_ was retained in the eutomers across the entire series. The *R*_max_ values displayed by all eutomers of **8–17** at 5-HT_2A_R were between 25 and 92%, whereas their efficacies at 5-HT_2C_R were much lower (*R*_max_ ∼ 0–20%).

**Figure 5:**
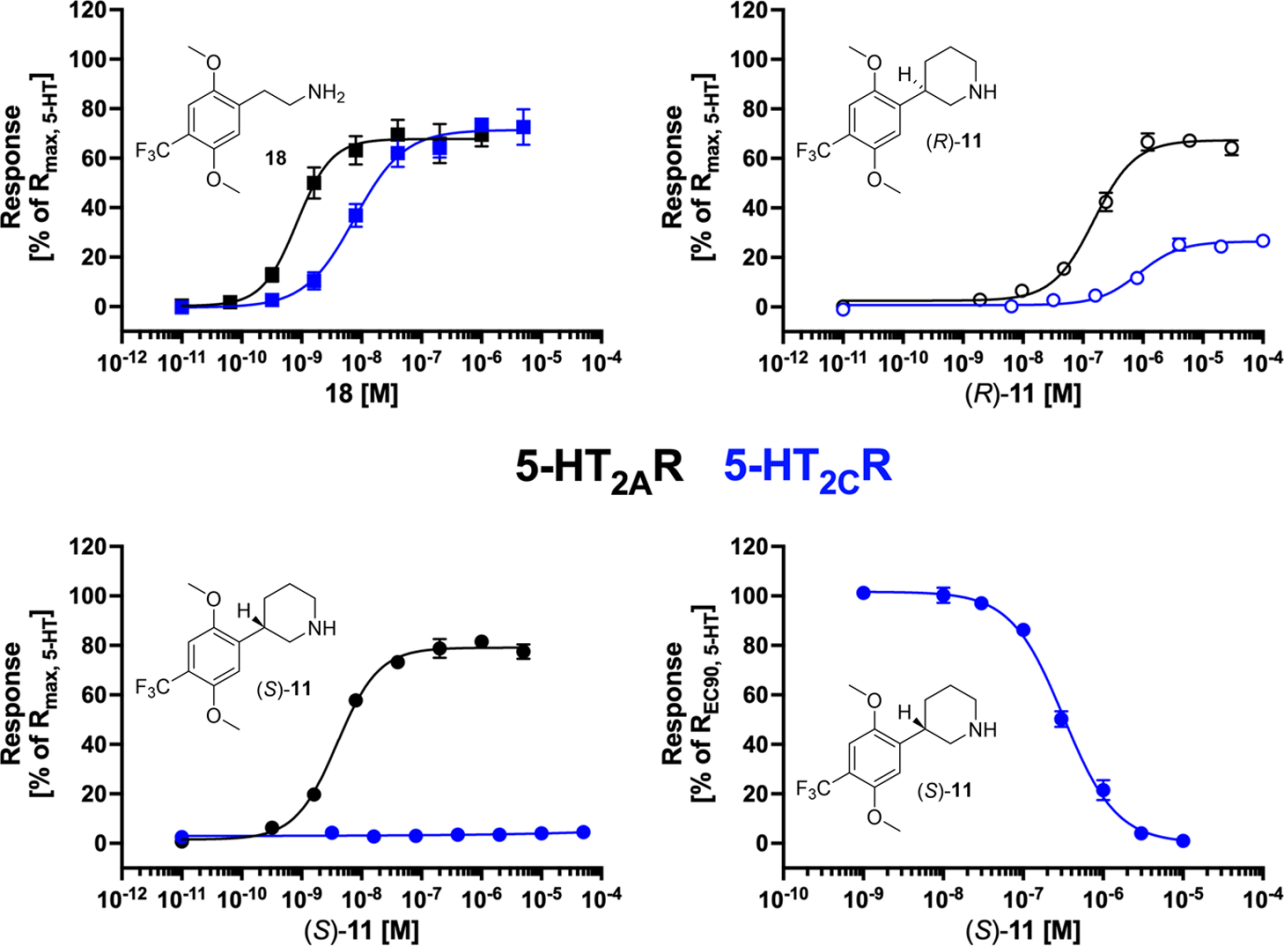
Functional properties exhibited by **18**, (*R*)-**11**, and (*S*)-**11** at stable 5-HT_2A_R- and 5-HT_2C_R-HEK293 cell lines in a Ca^2+^/Fluo-4 assay. Concentration–response relationships for 2C-TFM (**18**), (*R*)-**11**, and (*S*)-**11** at 5-HT_2A_R and 5-HT_2C_R, and concentration–inhibition relationship for (*S*)-**11** tested in antagonist mode at 5-HT_2C_R using 5-HT EC_90_ as agonist (bottom, right). Data are averaged data given as mean ± SEM values based on at least three independent determinations performed in duplicate, see Table S2.

Removal of the 4-substituent was detrimental for receptor activity, as **7** displayed very weak 5-HT_2A_R agonist activity (tested as a racemic mixture). Exchange of the 4-bromo-substituent in **6**_eu_ for chloro- or iodo-substituents provided analogues **8**_eu_ and **9**_eu_ displaying comparable agonist potencies at 5-HT_2A_R. In contrast, the 4-cyano group in **10**_eu_ was unfavorable for both 5-HT_2A_R and 5-HT_2C_R activities, whereas the TFM-substituted derivative **11**_eu_ possessed ∼20-fold higher agonist potency at 5-HT_2A_R than **6**_eu_. Moreover, **11**_eu_ did not elicit measurable agonist activity at 5-HT_2C_R and displayed an IC_50_ value of 320 nM at this receptor when tested as an antagonist (Table 1 and Figure 5). The three alkyl-substituted phenylpiperidines **12**_eu_, **13**_eu_, and **14**_eu_ displayed comparable agonist activities at 5-HT_2A_R with EC_50_ values of 100, 41, and 270 nM, respectively. While **13**_eu_ thus maintained potency comparable to **6**_eu_, the compound also exhibited measurable agonist activity at the 5-HT_2C_R. The thiomethyl and thioethyl derivatives **15**_eu_ and **16**_eu_ were also slightly more potent 5-HT_2A_R agonists than **6**_eu_, whereas the thioisopropyl derivative **17**_eu_ was less potent. Like the ethyl analogue, **13**_eu_, **15**_eu_, and **16**_eu_ did not match the functional subtype selectivity exhibited by **6**_eu_ for 5-HT_2A_R toward 5-HT_2C_R.

In general, the eutomers of the halogen- and TFM-substituted analogues **6**, **8**, **9**, and **11** displayed negligible agonist activity at 5-HT_2C_R, whereas the eutomers of the alkyl and thioalkyl derivatives **12**–**17** all evoked detectable agonist responses at this receptor. In contrast to the differential functionalities exhibited by the eutomers at 5-HT_2A_R and 5-HT_2C_R, the profiles of the distomers of **8**–**17** were similar at the two receptors, where they were weak-to-moderately potent partial agonists with 3–13 fold higher agonist potencies at 5-HT_2A_R (EC_50_ range: 110–2,200 nM) than at 5-HT_2C_R (EC_50_ range: 640–10,000 nM).

From this initial screening of different 4-substituents, the TFM-substituted **11**_eu_ seemed to be the most promising lead for a selective 5-HT_2A_R agonist from the phenylpiperidine series, so we decided to investigate the effects of further modifications to this compound. Before doing so, we established the absolute configuration of the distomer to be (*R*)-**11**, via X-ray crystallography (see the Methods section and Supporting Information for details), thereby showing the eutomer of **11** to be (*S*)-**11**. In all cases, the first eluting enantiomer on chiral high-performance liquid chromatography (HPLC) was also the most potent 5-HT_2A_R agonist and the least efficacious 5-HT_2C_R agonist of the relative enantiomers. Thus, we tentatively assign the (*S*)-configuration to the eutomers of all the phenylpiperidines reported in this study and correspondingly the (*R*)-configuration to all of the distomers.

Next, we investigated the effects of alkylation of the secondary amine as well as the impact of modifications to the 2-MeO and 5-MeO substituents on the phenyl ring in **11**. To facilitate direct comparisons between the phenylethylamine (2C-X) and phenylpiperidine scaffolds, 2C-TFM (**18**), one of the most potent 5-HT_2A_R agonists from the 2C-X family published to date,^21^ was included as a reference. The structures of **18** and phenylpiperidines **19**-**26** are given in Figure 4, and their functional properties at 5-HT_2A_R and 5-HT_2C_R in the Ca^2+^/Fluo-4 assay are given in Table 2.

**Table 2:**
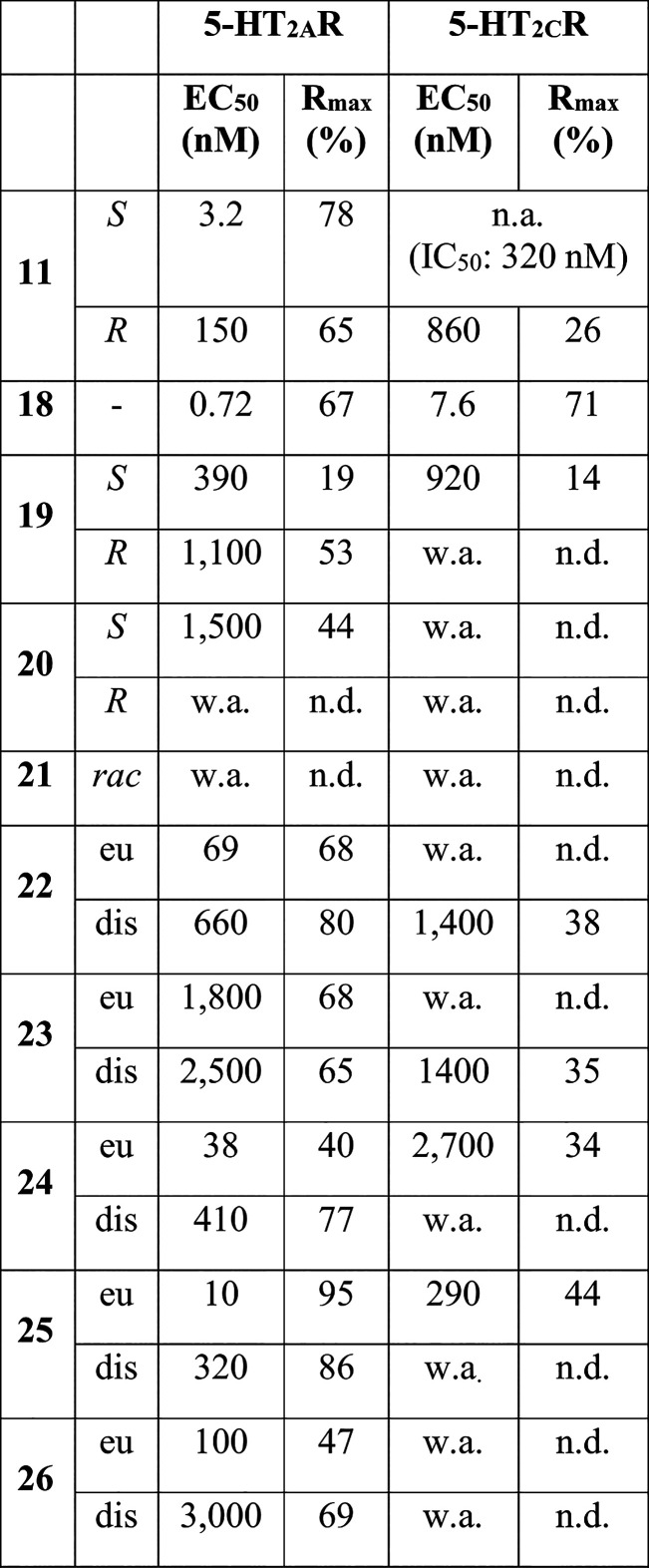
Functional Potencies of **11** and **18**–**28** at 5-HT_2A_R and 5-HT_2C_R^a^

a Functional properties exhibited by **11** and **18**–**26** at stable 5-HT_2A_R- and 5-HT_2C_R-HEK293 cell lines in the Ca^2+^/Fluo-4 assay. eu: eutomer, dis: distomer. EC_50_ values are given in nM, and *R*_max_ as % of the 5-HT *R*_max_. All data are based on at least 3 independent experiments. n.a.: no agonist activity. The compound displayed no significant agonist activity or negligible levels of agonist activity at 5-HT_2C_R at concentrations up to 50 μM. w.a.: weak agonist activity: the compound only elicited significant agonist responses at micromolar concentrations, so a complete concentration–response curve could not be obtained. n.d.: not determinable. The *R*_max_ value for the compound could not be determined since a complete concentration–response curve was not obtained in the tested concentration range (up to 50 μM). As **19** and **20** are direct derivatives of compounds (*S*)-**11** and (*R*)-**11**, absolute configuration has been assigned to the individual enantiomers of these compounds. See Table S2 for full details on the data reported in Table 2.

Glennon and colleagues have reported that sequential N-methylation of 2C–B (**1**) gives analogues with 10-fold reduced affinities at 5-HT_2A_R.^43^ Analogously, the *N*-methyl and *N*-ethyl derivatives **19** and **20** were both substantially less potent than **11**. Further extension of the methyl/ethyl group in this scaffold has been investigated by Nichols et al.^39^ The racemic NBOMe analogue of **6** exhibited a *K*_i_ value of 2 μM at the 5-HT_2A_R in a [^3^H]-ketanserin competition binding assay, and thus N-substitution of the phenylpiperidine scaffold appears to be unfavored for 5-HT_2A_R activity.

Deletion of both methoxy groups on the phenyl ring was detrimental to activity, as compound **21** displayed negligible agonist activity at both 5-HT_2A_R and 5-HT_2C_R, when tested as a racemic mixture (Table 2). Deletion of the 5-MeO in (*S*)-**11** led to a 20-fold drop in agonist potency at 5-HT_2A_R (**22**), whereas deletion of the 2-MeO group (**23**) led to a more than 500-fold drop in potency. These effects are even more pronounced than what we previously have observed for desmethoxy analogues of 2C-B (**1**) and 1-(4-bromo-2,5-dimethoxyphenyl)propan-2-amine (DOB).^44^ Extension of either MeO group to an EtO group was somewhat tolerated with respect to agonist potency at 5-HT_2A_R. Thus, the 2-EtO analogue **24**_eu_ and the 5-EtO analogue **25**_eu_ displayed 10- and 3-fold higher EC_50_ values, respectively, than (*S*)-**11** at 5-HT_2A_R, whereas the 2,5-di-EtO-analogue **26**_eu_ displayed a 30-fold higher EC_50_ value than (*S*)-**11** at the receptor (Table 2). **24**_eu_ and **25**_eu_ displayed 70- and 30-fold higher agonist potencies at 5-HT_2A_R than at 5-HT_2C_R, respectively, but both EtO analogues induced robust activation of the latter receptor. The negligible agonist efficacy displayed by (*S*)**−11** at 5-HT_2C_R suggests that both the 2- and 5-MeO groups in (*S*)-**11** are important for its low intrinsic activity at this receptor.

In summary, in this last series, we observed the same overall trend again; that the 5-HT_2A_R agonist activity primarily resides in one enantiomer (Table 2). However, none of the structural modifications presented in Figure 4 proved beneficial in terms of agonist potency at 5-HT_2A_R or selectivity toward 5-HT_2C_R, when compared to (*S*)-**11**.

2C-TFM (**18**) is a very potent partial agonist of both 5-HT_2A_R and 5-HT_2C_R, with a 10-fold selectivity for 5-HT_2A_R (Figure 4 and Table 2). The phenethylamine side chain in this 2C-X analogue is inherently flexible, allowing it to adopt numerous different conformations. Restricting the conformationally flexibility of the phenethylamine side chain by incorporating it into a piperidine ring in (*R*)-**11** leads to a 100-fold drop in agonist potency at both 5-HT_2A_R and 5-HT_2C_R. With the other enantiomer, (*S*)-**11,** we only see a 4-fold drop in agonist potency at 5-HT_2A_R accompanied by the absence of measurable agonist efficacy at 5-HT_2C_R. We speculate that (*S*)-**11** is unable to adopt a conformation capable of eliciting substantial 5-HT_2C_R activation, thus converting the compound into a competitive antagonist or a very low-efficacious agonist at this receptor, so low that its agonist activity is not detectable in the Ca^2+^/Fluo-4 assay. This profile is also seen with compounds **6**, **7** and **8** for which the eutomers at the 5-HT_2A_R are also *de facto* antagonists at the 5-HT_2C_R in this assay.

Based on the above investigations, compound (*S*)-**11** was selected for further characterization and renamed LPH-5.

**Figure 6:**
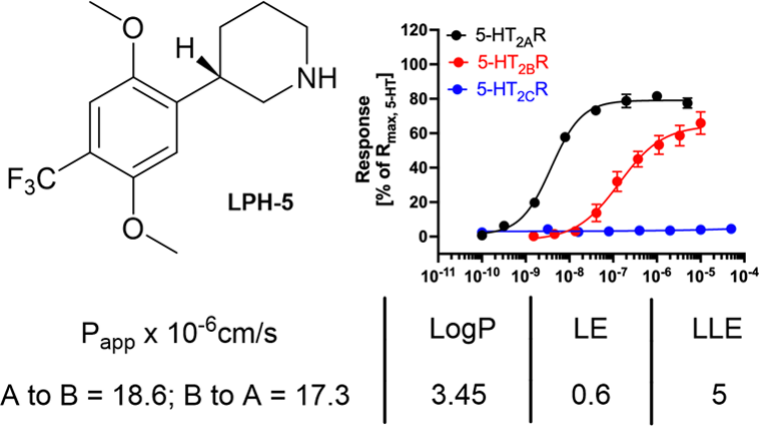
Overview of the functional properties, membrane permeability, Log *P*, LE, and LLE of LPH-5.

Agonism at the 5-HT_2B_R has been linked to cardiac valvular fibrosis,^45^ and in 2023, the FDA issued a regulatory guidance protocol for the clinical development of new 5-HT_2A_R agonists, wherein this pharmacological relationship is specifically mentioned as a safety concern.^46^ In a fluorescence-based Ca^2+^ imaging assay, LPH-5 was found to be a moderately potent partial 5-HT_2B_R agonist (EC_50_: 190 nM; *R*_max_: 65%), thus exhibiting an ∼60 fold selectivity for 5-HT_2A_R over 5-HT_2B_R (Figure 6). As for the *de facto* antagonist activity displayed by (*S*)-**11** (and by the eutomers of several other analogues in this series) at 5-HT_2C_R, the transient nature of the agonist-induced response and the resulting lack of equilibrium conditions in the Ca^2+^/Fluo-4 assay mean that the obtained IC_50_ values in the assay are not applicable for calculations of *K*_i_ or *K*_b_ values for the compounds. While absolute EC_50_ and IC_50_ values should not be compared directly, the 100-fold difference in the average EC_50_ (3.2 nM) and IC_50_ (320 nM) values displayed by (*S*)-**11** at 5-HT_2A_R and 5-HT_2C_R, respectively, is nevertheless noteworthy. Thus, we propose that (*S*)-**11**, in addition to its functional selectivity arising from its distinct intrinsic agonist activities at 5-HT_2A_R and 5-HT_2C_R, also displays substantial potency-based subtype selectivity at the two receptors. In this context, it should be noted that psilocin (the active metabolite of psilocybin currently being investigated in numerous clinical trials) has been reported to be an equipotent and equiefficacious agonist at 5-HT_2A_R, 5-HT_2B_R, and 5-HT_2C_R.^47^

Subsequently, the binding affinities of LPH-5 at all three 5-HT_2_Rs were determined in a [^125^I]-1-(4-iodo-2,5-dimethoxyphenyl)propan-2-amine ([^125^I]DOI) competition binding assay. The selectivity trend observed in the functional data was mirrored in these experiments, as LPH-5 displayed a *K*_i_ value of 1.3 nM for 5-HT_2A_R and a *K*_i_ value of 13 nM at both 5-HT_2B_R and 5-HT_2C_R (Figure S2 and Table S3). Encouraged by the selectivity profile exhibited by LPH-5 at the 5-HT_2_Rs, a broad screen with the compound was performed (see Table S4 for the full list of the targets). The data from this screen indicates the LPH-5 possesses binding affinities at the respective targets in the high nanomolar (>100 nM) or micromolar ranges, and we conclude that it will be possible to obtain selective activation of 5-HT_2A_R with LPH-5 at a suitable dose/exposure of the compound.

We then went on to characterize LPH-5 with respect to lipophilicity (Log *P*, see Table S5) and membrane permeability (MDR1-MDCKII, see Table S6) (Figure 6). LPH-5 displayed high bidirectional membrane permeability, with an efflux ratio of 0.94 and a Log *P* value of 3.45. Ligand efficiency (LE) and ligand lipophilicity efficiency (LLE) are two simple metrics often used to evaluate the properties of ligands at an early stage of the discovery phase.^48^ Using the pEC_50_ value of 8.49 obtained for LPH-5 at 5-HT_2A_R gives a LE of 0.6 and a LLE of 5, which in both cases are favorable values for a CNS-targeted compound.^49^

## Conclusion

In conclusion, we have investigated the structure–activity relationships of 2,5-dimethoxy-phenylpiperidines as a new class of 5-HT_2A_R agonists and found that LPH-5 is a potent and selective 5-HT_2A_R agonist with desirable drug-like properties.

## Methods

### Experimental Section

#### General Experimental Details

All reactions were performed under an atmosphere of argon unless otherwise indicated. Reagents and starting materials were obtained from commercial sources and used as received. Solvents were of chromatography grade or dried either by an SG Water solvent purification system (DCM, DMF, THF) or with 3 Å molecular sieves (DMSO, toluene, MeCN, Et_2_O, EtOH, DME, and MeOH). Reactions sensitive to water were run in flame- or oven-dried (150 °C) glassware under N_2_ or argon. Purification by column chromatography and dry column vacuum chromatography (DCVC) was performed following standard procedures using Merck Kieselgel 60 (40–63 μm or 15–40 μm mesh, respectively. Microwave heated reactions were performed using a Biotage Initiator apparatus in a sealed vial using an external surface sensor for temperature monitoring.

#### Thin-Layer Chromatography (TLC)

For TLC analysis, precoated silica gel 60 F254 plates purchased from Merck were used. EtOAc, *n*-heptane, acetone, toluene, DCM, Et2O, MeOH, Et_3_N, and mixtures thereof were used as eluents. Visualization of the compounds was achieved with UV light (254 nm), iodine on silica or potassium permanganate, anisaldehyde, ninhydrin, or ferric chloride stains. The denoted retention factors (*R*_f_) were rounded to the nearest 0.05.

#### Liquid Chromatography Mass Spectrometry (LCMS)

LC/MS analyses were performed on a Shimadzu Prominence chromatograph connected to the Applied Biosystems API 2000 mass spectrometer, column Phenomenex Gemini 5 μm C18; 50 × 2 mm, eluent MeCN (+0.1% HCOOH)/H_2_O (+0.1% HCOOH).

#### High-Performance Liquid Chromatography (HPLC) Methods

As a part of the essential characterization of new chemical entities, all reported final compounds were assayed for purity by HPLC. All compounds reported are >95% pure by HPLC analysis (see the Supporting Information for representative spectra). HPLC retention times (*t*_R_) for all final compounds are reported in minutes (min) and were determined by different methods, given in parentheses.

#### Method A (Analytical HPLC)

HPLC was recorded on a Thermo Scientific Dionex 3000 UltiMate instrument connected to a Thermo Scientific Dionex 3000 diode array detector using a Gemini-NX 3 μm C18 110A (250 × 4.6 mm) column with UV detection at 205, 210, 254, and 280 nm. MP A: 0.1% TFA in H_2_O (v/v). MP B: 0.1% TFA, 10% H_2_O in MeCN (v/v/v). Flow rate: 1.0 mL/min. Gradient: 0–20 min: 0–100% MP B.

#### Method B (Analytical HPLC)

HPLC was recorded on a Thermo Scientific Dionex 3000 UltiMate instrument connected to a Thermo Scientific Dionex 3000 diode array detector by a Gemini-NX 3 μm C18 110A (250 × 4.6 mm) column with UV detection at 205, 210, 254, and 280 nm. Mobile phase (MP) A: 0.1% TFA in H_2_O (v/v). MP B: 0.1% TFA, 10% H_2_O in MeCN (v/v/v). Flow rate: 1.0 mL/min. Gradient: 0–30 min: 0–100% MP B.

#### Method C (Preparative HPLC)

Preparative HPLC was performed on a Thermo Scientific Dionex 3000 ultimate instrument connected to a Thermo Scientific Dionex 3000 photodiode array detector using a Gemini-NX 5u RP C18 column (250 × 21.2 mm) with UV detection at 254 and 280 nm. MP A: 0.1% TFA, 100% H_2_O (v/v). MP B: 0.1% TFA, 10% H_2_O, 90% MeCN (v/v/v). Flow rate: 20 mL/min. Gradient: 0–25 min: 0–100% MP B, 25–30 min: 100% MP B.

#### Method D (Chiral HPLC)

Enantiomeric excess (ee) of the desired enantiomers was determined using a Thermo Scientific Dionex 3000 UltiMate instrument connected to a Thermo Scientific Dionex 3000 diode array detector using an analytical Phenomenex Lux 5 Amylose-2 (250 × 4.6 mm) chiral column with UV detection at 205, 210, 254, and 280 nm. MP A: 0.1% diethylamine in heptane (v/v). MP B: 0.1% diethylamine in EtOH (v/v). Flow rate: 10.0 mL/min using an isocratic gradient: 10% MP B.

#### High-Resolution Mass Spectrometry (HRMS)

Analysis was performed by matrix-assisted laser ionization time-of-flight mass spectrometry (MALDI-TOF). Analysis was performed in positive ion mode with MALDI ionization on a Thermo QExactive Orbitrap mass spectrometer (Thermo Scientific, Bremen, Germany) equipped with an AP-SMALDI 10 ion source (TransmitMIT, Giessen, Germany) and operated with a mass resolving power of 140,000 at *m*/*z* 200. 2,5-Dihydroxybenzoic acid was used as matrix and lock mass for internal mass calibration, providing a mass accuracy of 3 ppm or better. Samples were prepared using 2,5-dihydroxybenzoic acid as the matrix.

#### Melting Point (MP)

Melting point was measured for recrystallized compounds on a Stanford Research System OptiMelt capillary melting point apparatus with visual inspection, and the values are reported in a range rounded to the nearest 0.5 °C.

#### Nuclear Magnetic Resonance Spectroscopy (NMR)

NMR experiments were performed on a 300, 400, or 600 MHz Bruker instrument or a Varian Mercury (400 MHz) instrument. The obtained spectra were analyzed using MestReNova 11.0 software typically using Whittaker smoother baseline correction. Chemical shifts are reported in ppm (δ) with reference to the deuterated solvent used. Coupling constants (*J*) are reported in Hertz (Hz). Multiplet patterns are designated the following abbreviations or combinations thereof: br (broad), m (multiplet), s (singlet), d (doublet), t (triplet), q (quartet), p (pentet), sex (sextet), and hep (heptet).

## General Procedures

### General Procedure A. The Coupling of Sulfonyl Hydrazines with Boronic Acid to Give Phenyl Azetidines and Phenyl Pyrrolidines

Using the procedure reported by Ley and coworkers, a flame-dried microwave vial, backfilled with argon gas, was charged with Boc-protected sulfonylhydrazone (1 equiv), boronic acid (2 equiv), and dry Cs_2_CO_3_ (1.1 equiv).^50^ The contents of the vial were sealed and subjected to high vacuum for 2 h before re-establishing the argon atmosphere. The contents of the vial were suspended in anhydrous 1,4-dioxane (0.12 M). The suspension was thoroughly degassed before capping the vial. The reaction was heated to 110 °C under vigorous stirring. After 18 h, the reaction was allowed to cool to ambient temperature and filtered over a plug of Celite. The filtrate was concentrated *in vacuo* and immediately subjected to purification by flash column chromatography (1:2 EtOAc/heptane) giving the desired compound with minor impurities as a clear oil. Crude carboxylate was dissolved in 4 M HCl in dioxane (1 mL) and stirred at ambient temperature for 24 h. The pure amine hydrochloride was precipitated out by the addition of Et_2_O (25 mL) and isolated by decantation giving the title compound.

### General Procedure B-1. The Separation of Enantiomeric Mixtures of Free Amines

Analytical amounts of the racemate were dissolved in a mixture of MeOH, EtOH, and diethylamine (10:17:0.1) and separated by enantiomeric separation method 1, 2, or 3 unless otherwise specified. The hydrochloride salts were prepared by dissolving the products in a minimum amount of Et_2_O and treating the solution with 4 M HCl in dioxane. The precipitate was isolated by decantation and redissolved in the minimum amount of MeOH. Et_2_O was added dropwise until nucleation was observed, and the solution was allowed to crystallize at −4 °C overnight giving the pure title compound as white or off-white solids. Enantiomeric excess (ee) of the desired enantiomer was determined using Chiral HPLC Method 1 (ee > 95%).

### General Procedure B-2. The Separation of Enantiomeric Mixtures of Boc-Protected Amines

5–19 mg of the racemate was dissolved in a suitable mixture of the corresponding mobile phases and separated by chiral HPLC using a suitable combination of colomns, mobile phases, and flow rates as specified in conditions 1, 2, 3, 4, or 5 below. Enantiomeric excess (ee) of the desired enantiomer was determined using the same system (ee > 95%). In all cases, the eutomer eluted first.

*Condition* 1: Daicel Chiralpak IG 250 × 30 mm, 5 μm; mobile phase: 5% isopropanol/95% heptane; elution: isocratic; detection: UV 210 nm; flow rate: 40 mL/min.

*Condition* 2: Daicel Chiralpak IF 250 × 30 mm, 5 μm; mobile phase: 2% isopropanol/98% heptane; elution: isocratic; detection: UV 210 nm; flow rate: 40 mL/min.

*Condition* 3: Daicel Chiralpak IG 250 × 30 mm, 5 μm; mobile phase: 30% isopropanol/70% heptane (+0.1% diethylamine); elution: isocratic; detection: UV 210 nm; flow rate: 40 mL/min. Analytical column: Chiralpak IG 250 × 4.6 mm, 5 μm; mobile phase: 30% isopropanol/70% heptane (+0.1% diethylamine); elution: isocratic; detection: UV 210 nm; flow rate: 1 mL/min.

*Condition* 4: Daicel Chiralpak IG 250 × 30 mm, 5 μm; mobile phase: 15% isopropanol/85% heptane (+0.1% diethylamine); elution: isocratic; detection: UV 210 nm; flow rate: 40 mL/min. Analytical column: Chiralpak IG 250 × 4.6 mm, 5 μm; mobile phase: 15% isopropanol/85% heptane (+0.1% diethylamine); elution: isocratic; detection: UV 210 nm; flow rate: 1 mL/min.

*Condition* 5: Daicel Chiralpak IG 250 × 30 mm, 5 μm; mobile phase: 2% isopropanol/98% heptane; elution: isocratic; detection: UV 210 nm; flow rate: 40 mL/min.

### General Procedure C. The Suzuki Coupling of Pyridyl Boronic Acids and 3-Bromopyridine

A flame-dried round-bottom flask equipped with a stir bar and a cooler, backfilled with N_2_ gas, was charged with the appropriate boronic acid (1 equiv), 3-bromopyridine (1.1 equiv), triphenylphosphine (0.15 equiv), and DME (10 M). 2 M aqueous Na_2_CO_3_ (2.7 equiv) was added followed by Pd/C (0.15 mmol). The reaction was stirred at 80 °C for 17 h under a N_2_ atmosphere. The reaction was allowed to cool to ambient temperature and then filtered through a pad of Celite. The filtrate was diluted with H_2_O (50 mL) and EtOAc (50 mL). The phases were separated, and the aqueous phase further extracted with EtOAc (3 × 50 mL). The combined organic phases were washed with H_2_O and brine before being dried over MgSO_4_, filtered, and evaporated *in vacuo*. The crude product was purified by flash column chromatography to give the title compound.

### General Procedure D. Hydrogenantion of Phenylpyridines Using ThalesNano H-Cube

Phenylpyridine was dissolved in glacial AcOH (0.01M). ThalesNano H-Cube was loaded with a fresh catalyst cartridge (Pd(OH)_2_/C). The apparatus was set to run at 100 °C and 80 Barr. The reaction was followed by TLC. Upon complete consumption of starting material, AcOH was removed *in vacuo.*

### General Procedure E. The Suzuki Coupling of Pyridyl Boronic Acids with Aryl Bromides

A flame-dried microwave vial equipped with a stir bar and backfilled with argon gas was charged with the appropriate boronic acid (1 equiv) and [1,1′-bis(di-*tert*-butylphosphino)ferrocene]dichloropalladium(II) (5 mol %) within a glovebox and tightly sealed. Aqueous degassed K_3_PO_4_ solution (0.9 M, 1.5 equiv) was added, followed by addition of the corresponding aryl bromide (1 equiv) in degassed dioxane (0.3 M). The resulting mixture was heated by microwave irradiation at 100 °C for 1 h, then allowed to cool to ambient temperature, filtered through a silica pad, further eluted with EtOAc (50–100 mL), and then evaporated *in vacuo*. The residue was purified by flash column chromatography using mobile phase mixtures of petroleum ether/EtOAc.

### General Procedure F1. Hydrogenation of Phenylpyridines Using Parr Apparatus

Phenylpyridine (1 equiv) was dissolved in glacial AcOH (2.0 M) in a hydrogenation flask. PtO_2_ (0.1 equiv) was added, and the reaction vessel was shaken under 4 bar H_2_ pressure on a Parr apparatus for 24 h. The reaction mixture was washed through a pad of Celite with EtOAc (25 mL), and the filtrate was basified using 10% aq. NaOH solution (150 mL). The phases were separated, and the aqueous layer was further extracted with EtOAc (2 × 50 mL). The combined organic phases were dried over MgSO_4_, filtered, and evaporated *in vacuo*.

### General Procedure F2. Hydrogenation of Phenylpyridines Using Pressure Reactor

To a stirred solution of the substrate (1 equiv) in glacial AcOH (0.25 M) was added PtO_2_ (15 mol %). The reaction mixture was hydrogenated at ambient temperature under 10 bar H_2_ pressure in a Buchi tinyclave steel pressure reactor. After 16 h, the reaction mixture was filtered through a syringe filter and evaporated *in vacuo*. The residue was partitioned between DCM and aq. sat. NaHCO_3_. The organic phase was separated, and the aqueous phase was further extracted with DCM (2×). Combined organic extracts were dried over Na_2_SO_4_, filtered, and evaporated *in vacuo*.

### General Procedure G. Introduction of Boc-Protecting Group

A round-bottom flask was charged with the amine (1 equiv), Boc_2_O (1.5 equiv), and DCM (0.1 M) before Et_3_N (2 equiv) was added. The resulting mixture was stirred for 1 h at ambient temperature and then evaporated to dryness *in vacuo*. The residue was purified by flash column chromatography using mobile phase mixtures of Petroleum ether/EtOAc.

### General Procedure H. Cleavage of Boc-Protecting Group

A round-bottom flask was charged with the Boc-portected amines (1 equiv), and ethereal HCl (40 equiv) was added. The solution was stirred for 3–7 days at ambient temperature to achieve full conversion eventually giving the desired product as a white precipitate. The slurry was centrifuged, and the ethereal layer discarded. The resulting solids were washed with Et_2_O and evaporated to dryness *in vacuo*.

### General Procedure I. Bromination of Phenols

A flame-dried round-bottom flask, equipped with a stir bar and a rubber septum, backfilled with argon gas, was charged with the corresponding phenol (1 equiv) in DCM and AcOH (2:1, 0.1 M), and elemental bromine was added (1.05 equiv) dropwise at 0 °C. The reaction mixture was slowly warmed to ambient temperature overnight and then evaporated directly *in vacuo*. The residue was purified by flash column chromatography using mobile phase mixtures of petroleum ether/EtOAc.^51^

### General Procedure J. Methylation of Phenols

A flame-dried microwave vial equipped with a stir bar and backfilled with argon gas was charged with the corresponding phenol (1 equiv) in acetone (0.25 M). K_2_CO_3_ (8 equiv) was added followed by methyl iodide (6 equiv). The vial was sealed, and the reaction mixture was stirred for 5 h at 60 °C, then evaporated directly *in vacuo*, and partitioned between DCM and H_2_O. Phases were separated, and the aqueous phase was further extracted with DCM. Combined organic extracts were dried over Na_2_SO_4_, filtered, and evaporated *in vacuo*. The residue was purified by flash column chromatography using mobile phase mixtures of petroleum ether/EtOAc.

### General Procedure K. Synthesis of Alkylsulfanes from 1,4-Dibromo-2,5-Dimethoxybenzenes

A flame-dried round-bottom flask equipped with a stirr bar and a rubber septum, backfilled with argon gas, was charged with 1,4-dibromo-2,5-dimethoxybenzene (1 equiv) in dry THF (0.4 M). The resulting solution was cooled to −78 °C. and 2.5 M *n-*BuLi in hexanes (1.1 equiv) was added dropwise. The reaction mixture was stirred at this temperature for 1 h before the dropwise addition of the appropriate disulfide (1.1 equiv). The mixture was allowed to warm to ambient temperature, stirred for 1 h, and then quenched with 1 M HCl. The mixture was concentrated under reduced pressure to half of the initial volume. Et_2_O was added, and phases were separated. The organic phase was washed with H_2_O and NaHCO_3_ and then concentrated *in vacuo*. The residue was purified by flash column chromatography using mobile phase mixtures of petroleum ether/EtOAc.

### General Procedure L. Reductive Amination

A flame-dried round-bottom flask equipped with a stirr bar and a rubber septum was charged with the amine (1 equiv) and MeOH (61.5 mM). The corresponding aldehyde (5 equiv) was added, followed by one drop of AcOH. The resulting mixture was cooled to 0 °C, and then NaCNBH_3_ (3 equiv) was added. The reaction mixture was allowed to warm up to room temperature overnight. DCM was added, and the mixture was washed with 1 M NaOH and brine. The organic phase was dried over anhydrous Na_2_SO_4_, filtered, and evaporated. The residue was taken up in Et_2_O and treated with ethereal 2 M HCl (5 equiv). The resulting suspension was centrifugated. The supernatant was discarded, and the solid was washed with ether and evaporated to dryness *in vacuo*.

### General Procedure M. Ethylation of Phenols

A flame-dried microwave vial equipped with a stir bar and backfilled with argon gas was charged with the corresponding phenol (1 equiv) in acetone (0.25 M). K_2_CO_3_ (8 equiv) was added followed by bromoethane (5 equiv). The vial was sealed, and the reaction mixture was stirred for 5 h at 60 °C, then evaporated directly *in vacuo*, and partitioned between DCM and H_2_O. Phases were separated, and the aqueous phase was further extracted with DCM. Combined organic extracts were dried over Na_2_SO_4_, filtered, and evaporated *in vacuo*. The residue was purified by flash column chromatography using mobile phase mixtures of petroleum ether/EtOAc.

### Enantiomeric Separation Method 1. Chiral HPLC

Analytical amounts of racemate were dissolved in a mixture of MeOH, EtOH, and diethylamine (10:17:0.1) and separated, unless otherwise specified, on a Thermo Scientific Dionex 3000 UltiMate instrument connected to a Thermo Scientific Dionex 3000 diode array detector by a Phenomenex Lux 5 Amylose-2 (250 × 10 mm) chiral column with UV detection at 205, 210, 254, and 280 nm. MP A: 0.1% diethylamine in heptane (v/v). MP B: 0.1% diethylamine in EtOH (v/v). Flow rate: 10.0 mL/min using an isocratic gradient of 30–10% MP B. Loadings were between 1 and 3 mL per injection (3–5 mg/mL). Enantiomeric excess was determined on an identical instrument using a Phenomenex Lux 5 Amylose-2 (250 × 4.6 mm) chiral column (HPLC Method D).

### Enantiomeric Separation Method 2. Chiral SFC

Racemic amine as the hydrochloride was dissolved in MeOH and separated by preparative supercritical fluid chromatography (SFC). Separation was performed on a Diacell AD-H chiralpak colomn (250 × 21.2 mm) connected to a Berger Multigram II operating at 50 mL/min at 35 °C and 100 bar backpressure using stacked injections. MP: CO_2_ (75%) and ethanol + 0.1% diethylamine (25%). UV detection at 290 nM enantiomeric excess (ee) of the enantiomers was determined on a Diacell AD-H chiralpak column, 3 μm, 15 cm (150 × 4.6 mm) connected to an Aurora Fusion A5/Agilent SFC system operating at 4 mL/min at 40 °C and 150 bar backpressure. MP: CO_2_ (75%) and ethanol + 0.1% diethylamine (25%).

### Enantiomeric Separation Method 3. Resolution by Chiral Salt Formation and Crystallization

Racemic amine (1 equiv) was dissolved in MeOH (0.5 M) at room temperature and added over 5 min to a boiling solution of l(+)tartaric acid (1 equiv) in MeOH (70 mM). Upon complete addition, the reaction was left to cool to room temperature for 48 h yielding white crystalline solids which were isolated by filtration. The filtrate was left at 4 °C overnight giving a second crop of solids, isolated by filtration. Crops were combined, redissolved in boiling MeOH (40 mL), and allowed to cool to room temperature giving white solids, which were again subjected to recrystallization from boiling MeOH (20 mL) eventually giving clear prismatic crystals (5% total yield, 96% enantiomeric excess).

Enantiomeric excess (ee) of the desired enantiomer was determined using a Thermo Scientific Dionex 3000 UltiMate instrument connected to a Thermo Scientific Dionex 3000 diode array detector by a Phenomenex Lux 5 Amylose-2 (250 × 4.6 mm) chiral column with UV detection at 205, 210, 254, and 280 nm. MP A: 0.1% diethylamine in heptane (v/v). MP B: 0.1% diethylamine in EtOH (v/v). Flow rate: 10.0 mL/min using an isocratic gradient of 30–10% MP B.

## Compound **1**

### 2-(4-Bromo-2,5-dimethoxyphenyl)ethan-1-amine Hydrochloride

This was synthesized as previously described.^44^ All analytical data were in congruence with reported literature values.

## Compound **4**

### 4-Methoxybenzenesulfonohydrazide

A round-bottom flask equipped with a stir bar and a rubber septum was charged with 4-methoxybenzenesulfonyl chloride (5.17 g, 25 mmol) in THF (125 mL) and cooled to 0 °C. 50% aq. hydrazine solution (3.90 mL, 62.5 mmol) was added dropwise. The reaction mixture was stirred at 0 °C for 1 h before being evaporated *in vacuo*. The crude residue was partitioned between H_2_O (50 mL) and EtOAc (100 mL), and phases were separated. The aqueous phase was further extracted with EtOAc (2 × 100 mL). Combined organic phases were washed with H_2_O water (50 mL) and brine (50 mL), dried over Na_2_SO_4_, filtered, and evaporated *in vacuo* to yield the title compound as a colorless amorphous solid (3.79 g, 75%). ^1^H NMR (600 MHz, CDCl_3_) δ 7.85 (d, *J* = 8.9 Hz, 2H), 7.02 (d, *J* = 8.9 Hz, 2H), 5.55 (s, 1H), 3.89 (s, 3H), 3.59 (s, 2H); ^13^C NMR (151 MHz, CDCl_3_) δ 163.86, 130.63, 127.64, 114.68, 55.86.

### *tert*-Butyl 3-(2-((4-methoxyphenyl)sulfonyl)hydrazineylidene)azetidine-1-carboxylate

To a flame-dried microwave vial, backfilled with argon gas, were added 4-methoxybenzenesulfonohydrazide (3.83 g, 18.91 mmol) and *tert*-butyl 3-oxoazetidine-1-carboxylate (3.24 g, 18.93 mmol). The contents of the vial were dissolved in anhydrous DMSO (13 mL). The vial was capped and heated to 60 °C. The reaction was followed by H NMR. Upon completion, the reaction mixture was poured into H_2_O (350 mL) and the aqueous mixture was extracted with Et_2_O (3 × 100 mL). Combined organic phases were washed with H_2_O (5 × 50 mL) and brine (50 mL), dried over Na_2_SO_4_, filtered, and evaporated *in vacuo* to give the crude compound, in quantitative yields, as an off-white solid with minor impurities. The product was deemed of sufficient purity for use in the subsequent reactions without further purification. TLC *R*_f_ = 0.1 (33% EtOAc in heptane v/v); ^1^H NMR (400 MHz, DMSO-*d*_6_) δ 7.70 (dd, *J* = 8.9, 3.4 Hz, 3H), 7.08 (dd, *J* = 8.8, 4.2 Hz, 3H), 4.48–4.34 (m, 3H), 3.80 (d, *J* = 3.1 Hz, 4H), 1.33 (bs, 9H); ^13^C NMR (101 MHz, DMSO-*d*_6_) δ 163.13, 156.17, 148.44, 130.86, 130.00, 114.85, 79.96, 56.16, 28.38.

### (4-Bromo-2,5-dimethoxyphenyl)boronic Acid

To a flame-dried vessel, backfilled with argon gas, were added 1,4-dibromo-2,5-dimethoxybenzene (2.22 g, 7.5 mmol) and anhydrous THF (75 mL). The reaction was cooled to −78 °C before the slow, dropwise addition of *n*-BuLi solution (2.17 M, 7.5 mmol). The solution was stirred at −78 °C for 20 min before the addition of triisopropyl borate (5.19 mL, 22.5 mmol). The cooling source was removed, and the reaction was allowed to reach ambient temperature and stirred for an additional 20 h before quenching by careful addition of 2 M aq. HCl solution (15 mL). The aqueous mixture was diluted with Et_2_O (100 mL), and phases were separated. The aqueous phase was further extracted with Et_2_O (2 × 100 mL). Combined organic phases were washed with H_2_O (50 mL), dried over Na_2_SO_4_, filtered through a plug of silica, and evaporated to give the title compound as a crude white solid with minor impurities. The product was deemed of sufficient purity for use in the subsequent reactions without further purification.

### 3-(4-Bromo-2,5-dimethoxyphenyl)azetidine Hydrochloride (**4**)

This was synthesized according to general procedure A using *tert*-butyl 3-(2-((4-methoxyphenyl)sulfonyl)hydrazineylidene)azetidine-1-carboxylate (249 mg, 0.70 mmol), (4-bromo-2,5-dimethoxyphenyl)boronic acid (365 mg, 1.40 mmol). The title compound was isolated as a colorless crystalline solid (26 mg, 12%). TLC *R*_f_ = 0.1 (33% EtOAc in heptane v/v); ^1^H NMR (600 MHz, MeOD) δ 7.22 (s, 1H), 6.91 (s, 1H), 4.35 (d, *J* = 3.3 Hz, 2H), 4.33 (s, 2H), 4.30–4.25 (m, 1H), 3.85 (s, 6H); ^13^C NMR (151 MHz, MeOD) δ 153.17, 151.74, 127.91, 117.33, 113.85, 112.06, 57.57, 56.69, 52.50, 34.73; HRMS *m*/*z* calculated for [C_11_H_15_BrNO_2_]^+^ (M + H) 272.0281; found: 272.0282.

## Compound **5**

### *tert*-Butyl (*E*)-3-(((4-methoxyphenyl)sulfonyl)diazenyl)pyrrolidine-1-carboxylate

A flame-dried microwave vial, backfilled with argon gas, was charged with 4-methoxybenzenesulfonohydrazide (1.6 g, 7.9 mmol), *tert*-butyl 3-oxopyrrolidine-1-carboxylate (1.46 g, 7.9 mmol), and anhydrous MeOH (35 mL). The vial was capped and heated to 60 °C for 18 h. The reaction was followed by ^1^H NMR. Upon completion, the reaction mixture was poured into H_2_O (350 mL) and extracted with Et_2_O (3 × 100 mL). Combined organic phases were washed with H_2_O (5 × 50 mL) and brine (50 mL), dried over Na_2_SO_4_, filtered, and evaporated *in vacuo* to give the crude compound, in quantitative yields, as an off-white solid with minor impurities. The product was deemed of sufficient quality for use in the subsequent reactions without further purification. ^1^H NMR (400 MHz, CDCl_3_) δ 7.88 (d, *J* = 8.9 Hz, 2H), 7.04–6.92 (m, 2H), 3.99 (bs, 1H), 3.92 (bs, 1H), 3.87 (bs, 3H), 3.80 (bs, 1H), 3.75 (bs, 1H), 2.68 (m, 2H), 2.54 (s, 1H), 1.44 (s, 10H); ^13^C NMR (101 MHz, CDCl_3_) δ 26.3 (br), 28.4, 30.6 (br), 43.9 (br), 46.5 (br), 49.5 (br), 55.6, 80.1, 80.4, 114.3, 129.6, 131.3, 154.1, 159.9, 163.5.

### *tert*-Butyl 3-(2,5-dimethoxyphenyl)pyrrolidine-1-carboxylate

This was synthesized according to general procedure A using *tert*-butyl (*E*)-3-(((4-methoxyphenyl)sulfinyl)diazenyl)pyrrolidine-1-carboxylate (369.4 mg, 1 mmol), (2,5-dimethoxyphenyl)boronic acid (363.9 mg, 2 mmol). The title compound was isolated as a brown oil (42.6 mg, 13%). TLC *R*_f_ 0.3 (20% EtOAc in heptane v/v); ^1^H NMR (400 MHz, CDCl_3_) δ 6.83–6.66 (m, 3H), 3.79 (s, 4H), 3.76 (s, 4H), 3.64 (tt, *J* = 9.7, 6.9 Hz, 1H), 3.56 (ddd, *J* = 11.2, 8.1, 3.3 Hz, 1H), 3.38 (ddd, J = 10.7, 9.0, 6.9 Hz, 1H), 3.25 (dd, *J* = 10.5, 8.6 Hz, 1H), 2.17 (dtd, *J* = 12.8, 6.6, 3.3 Hz, 1H), 2.05–1.92 (m, 1H), 1.47 (s, 9H). ^13^C NMR (151 MHz, CDCl_3_) δ 154.74, 153.79, 151.88, 131.10, 113.78, 111.45, 111.40, 79.19, 56.08, 51.13, 45.66, 37.71, 31.34, 28.71.

### 3-(2,5-Dimethoxyphenyl)pyrrolidine Hydrochloride

To a round-bottom flask, equipped with a stir bar, charged with *tert*-butyl 3-(2,5-dimethoxyphenyl)pyrrolidine-1-carboxylate (72 mg, 0.23 mmol) and MeOH (2 mL), was added 4 M HCl in dioxane (0.5 mL). The reaction was stirred at ambient temperature for 2 h giving precipitation of the hydrochloride. The precipitate was isolated by decantation and dissolved in the minimum amount of MeOH. Et_2_O was added dropwise until nucleation was observed, and the solution was allowed to crystallize at −4 °C overnight giving the pure title compound as a white solid (54 mg, 94%). ^1^H NMR (600 MHz, MeOD) δ 6.97 (dd, J = 8.4, 0.9 Hz, 1H), 6.88–6.85 (m, 2H), 3.86 (s, 3H), 3.78 (s, 3H), 3.77–3.70 (m, 1H), 3.72–3.66 (m, 1H), 3.58 (ddd, *J* = 11.8, 8.4, 3.6 Hz, 1H), 3.38 (ddd, *J* = 11.6, 9.7, 7.2 Hz, 1H), 3.26 (dd, *J* = 11.0, 9.3 Hz, 1H), 2.40 (dh, *J* = 14.1, 3.6, 3.2 Hz, 1H), 2.22 (dtd, *J* = 13.0, 9.7, 8.4 Hz, 1H); ^13^C NMR (151 MHz, MeOD) 155.30, 152.98, 129.00, 115.59, 113.43, 112.93, 56.30, 56.15, 50.73, 46.84, 40.06, 30.92; HPLC *t*_R_ = 9.88 (Method A).

### 3-(4-Bromo-2,5-dimethoxyphenyl)pyrrolidine Hydrobromide (**5**)

A flame-dried vessel, backfilled with argon gas, was charged with 3-(2,5-dimethoxyphenyl)pyrrolidine hydrochloride (63.3 mg, 0.26 mmol) and glacial AcOH (1 mL). A solution of elemental bromine (14 μL, 0.28 mmol) in glacial AcOH (1 mL) was added dropwise. The reaction was shielded from light and stirred at ambient temperature. The reaction was monitored by TLC. Upon completion, the reaction was diluted with H_2_O (5 mL) and washed with Et_2_O (10 mL). The aqueous mixture was basified with 10% aq. NaOH solution and extracted with EtOAc (15 mL) followed by a mixture of EtOH and chloroform (1:2) (2 × 15 mL). The combined organics were dried over MgSO4, filtered, and evaporated *in vacuo* giving the crude hydrobromide as a brown solid in high purity (59 mg, 62%). Analytical amounts of the racemic mixture were separated and isolated as the two individual enantiomers as their hydrochloride salts using general procedure B-1, method 1, using an isocratic gradient of 25% MP B. Enantiomer 1: Rt 18.03, enantiomer 2: Rt 2= 26.02; ^1^H NMR (600 MHz, DMSO-d6) δ 8.88 (s, 2H), 7.25 (s, 1H), 7.06 (s, 1H), 3.84 (s, 3H), 3.80 (s, 3H), 3.64 (tt, *J* = 9.7, 7.8 Hz, 1H), 3.53 (dd, *J* = 11.3, 8.2 Hz, 1H), 3.41 (ddd, *J* = 11.9, 8.4, 3.8 Hz, 1H), 3.25 (ddd, *J* = 11.5, 9.3, 7.2 Hz, 1H), 3.13 (dd, *J* = 11.3, 9.7 Hz, 1H), 2.26 (dtd, *J* = 12.5, 7.3, 3.8 Hz, 1H), 2.08–1.99 (dtd, 1H).); ^13^C NMR (151 MHz, DMSO-d6) δ 151.42, 149.60, 127.90, 116.11, 112.57, 109.15, 56.88, 56.39, 48.85, 44.88, 36.98, 29.92; HPLC *t*_R_ = 18.30 (Method B); HRMS *m*/*z* calculated for [C_12_H_16_BrNO_2_]+ (M + H) 286.0437, found 286.0439.

## Compound **6**

### 3-(4-Bromo-2,5-dimethoxyphenyl)piperidine (**6**)

A round-bottom flask, equipped with a stir bar, was charged with **7** (1g, 3,87 mmol) and glacial AcOH (19 mL). A solution of elemental bromine (0.19 mL, 3.87 mmol) in glacial AcOH (10 mL) was added dropwise. The mixture was stirred for 30 min until complete precipitation of the product as a white solid. The reaction was diluted with Et_2_O (20 mL), and solids were isolated by filtration. The product was recrystallized from a mixture of boiling MeOH, isopropanol, and Et_2_O to give the product as a white solid (853,5 mg, 58%). Analytical amounts of the racemic mixture were separated and isolated as the two individual enantiomers as their hydrochloride salts using general procedure B-1, method 1, using an isocratic gradient of 25% MP B. Enantiomer 1: Rt 7.200, enantiomer 2: Rt 12.160. MP 253–254 °C; TLC *R*_f_ = 0.15 (0.01% TEA and 25% MeOH in EtOAc v/v/v); ^1^H NMR (400 MHz, MeOD) δ 7.22 (s, 1H), 6.97 (s, 1H), 3.88 (s, 3H), 3.86 (s, 3H) 3.45 (t, *J* = 12.9 Hz, 3H), 3.22–2.99 (m, 2H), 2.11 (d, *J* = 10.7 Hz, 1H), 2.04–1.84 (m, 3H); ^13^C NMR (101 MHz, MeOD) δ 152.76, 151.94, 130.33, 117.44, 113.33, 111.54, 57.61, 56.79, 45.23, 35.27, 28.90, 24.06. HPLC *t*_R_ = 14.07 (Method B); HRMS *m*/*z* calculated for [C13H18BrNO2]+ (M + H) 300.0594, found 300.0588.

## Compound **7**

### 3-(2,5-Dimethoxyphenyl)pyridine

This was synthesized according to general procedure C using (2,5-dimethoxyphenyl)boronic acid (2.184 g, 2,3 mmol). The crude product was purified by flash column chromatography (40% EtOAc in heptane v/v) to give the title compound in quantative yield as a clear oil with minor impurities. The product was deemed of sufficient purity and was used in subsequent reactions without further purification. TLC *R*_f_ = 0.3 (40% EtOAc in heptane v/v); ^1^H NMR (400 MHz, CDCl_3_) δ 8.77 (d, *J* = 1.8 Hz, 1H), 8.54 (dd, *J* = 4.7, 1.6 Hz, 1H), 7.84 (dt, *J* = 7.9, 1.9 Hz, 1H), 7.30 (ddd, *J* = 7.9, 4.8, 0.9 Hz, 1H), 6.92–6.85 (m, 3H), 3.78 (s, 3H), 3.73 (s, 3H); ^13^C NMR (101 MHz, CDCl_3_) δ 153.89, 150.83, 150.16, 148.03, 136.77, 134.10, 127.91, 122.90, 116.56, 113.92, 112.66, 56.19, 55.79; HPLC *t*_R_ = 9.88 (Method A).

### 3-(2,5-Dimethoxyphenyl)piperidine (**7**)

This was synthesized according to general procedure F1 using 3-(2,5-dimethoxyphenyl)pyridine (6.012 g, 27.93 mmol). The hydrochloride salt was prepared by dissolving the product in a minimum amount of Et_2_O and treating the solution with 4 M HCl in dioxane. The precipitate was isolated by decantation and redissolved in the minimum amount of MeOH. Et_2_O was added dropwise until nucleation was observed, and the solution was allowed to crystallize at −4 °C overnight giving the pure title compound as large white crystals (3.44 g, 48%). TLC *R*_f_ = 0.2 (5% TEA and 10% MeOH in EtOAc v/v/v); ^1^H NMR (400 MHz, CDCl3) δ 9.84 (s, 1H), 9.44 (s, 1H), 6.80–6.63 (m, 3H), 3.75 (s, 3H), 3.74 (s, 3H), 3.59–3.39 (m, 3H), 3.06 (q, *J* = 11.4 Hz, 1H), 2.85 (q, *J* = 12.1, 11.6 Hz, 1H), 2.24–2.05 (m, 1H), 1.96 (q, *J* = 13.4, 12.3 Hz, 3H), 1.83–1.65 (m, 1H); ^13^C NMR (101 MHz, CDCl_3_) δ 153.66, 151.38, 129.72, 114.23, 112.32, 111.65, 55.78, 55.75, 47.57, 44.08, 35.35, 28.28, 22.85; HPLC *t*_R_ = 17.04 (Method B).

## Compound **8**

### 3-(4-Chloro-2,5-dimethoxyphenyl)piperidine (**8**)

To a flame-dried round-bottom flask, backfilled with argon gas, was charged with **7** (500 mg, 1.93 mmol), *N*-chlorosuccinimide (310 mg, 2.32 mmol) and MeCN. The solution was cooled (0 °C), TiCl_4_ (0.2 mL, 1.93 mmol) was slowly added, and the reaction was stirred for 10 min. The cooling source was removed, and the reaction was stirred on for an additional 5 min before being quenched with MeOH (8 mL). The reaction was allowed to warm to ambient temperature and then basified (≈pH 9) with aq. NaOH solution (10% v/v) under precipitation of white solid. The solution was clarified by filtration through a fritted glass funnel and washed through with EtOAc (50 mL). The filtrate was washed with sat. aq. Na_2_CO_3_ (50 mL) and brine (50 mL), dried over MgSO_4_, filtered, and concentrated *in vacuo* to give the crude-free base with minimal impurities as a yellow solid (529 mg, 94% crude yield). Analytical amounts of the racemic mixture were separated and isolated as the two individual enantiomers as their hydrochloride salts with minor impurities using general procedure B-1, method 1, using an isocratic gradient of 30% MP B. Enantiomer 1: Rt 6.943, Enantiomer 2: Rt 13.493. Both enantiomers were recrystallized again from mixture of EtOAc, isopropanol, and Et_2_O giving both enantiomers in high purity. MP 235–236 °C; TLC *R*_f_ = 0.2 (5% TEA and 10% MeOH in EtOAc v/v/v); ^1^H NMR (400 MHz, CDCl_3_) δ 7.07 (s, 1H), 6.99 (s, 1H), 3.88 (s, 3H), 3.85 (s, 3H), 3.49–3.38 (m, 3H), 3.15–3.09 (m, 1H), 3.06 (td, *J* = 12.8, 3.5 Hz, 1H), 2.15–2.04 (m, 1H), 2.01–1.86 (m, 3H); ^13^C NMR (101 MHz, CDCl3) δ 152.55, 150.87, 129.61, 122.80, 114.52, 113.63, 57.50, 56.74, 49.05, 45.18, 35.17, 28.95, 24.05; HPLC *t*_R_ = 18.54 (Method A); HRMS *m*/*z* calculated for [C_13_H_18_ClNO_2_]+ (M + H) 256.1099, found 256.1102.

## Compound **9**

### 3-(4-Iodo-2,5-dimethoxyphenyl)piperidine (**9**)

A flame-dried round-bottom flask, equipped with a stir bar, backfilled with argon gas, was charged with **7** (500 mg, 1.9 mmol), TEA (0.53 mL, 3.8 mmol), and DCM. The reaction mixture was cooled to 0 °C in an ice bath, and trifluoroacetic anhydride (483.06 mg, 2.3 mmol) was carefully added under vigorous stirring. The reaction was stirred for 5 min at 0 °C before being allowed to warm to ambient temperature and stirred for approximately 40 min. The reaction was monitored by TLC. Upon completion, the reaction was quenched with H_2_O (20 mL) and phases were separated. The aqueous layer was further extracted with EtOAc (2 × 50 mL). The combined organic layers were washed with H_2_O (50 mL) and brine (50 mL), then dried over MgSO_4_, filtered, and concentrated *in vacuo* to give the crude trifluoroacetamide in quantative yield. TLC *R*_f_ = 0.5 (33% EtOAc in heptane v/v). The crude product was dissolved in MeOH (20 mL) and purged with a flow of argon gas. The reaction was cooled to 0 °C in an ice bath and shielded from light with aluminum foil. AgNO_3_ (355 mg, 2.09 mmol) was added in one portion followed by I_2_ (578 mg, 2.28 mmol) in several small portions. The reaction was stirred at 0 °C for 1.75 h and then washed through a plug of Celite into a mixture of ice and sat. aq. NaHSO_3_. The mixture was allowed to warm to ambient temperature, and organics were evaporated *in vacuo*. The remaining aqueous mixture was extracted with EtOAc (3 × 50 mL). The combined organic phases were washed with H_2_O (50 mL) and brine (50 mL), dried over MgSO_4_, then filtered, and concentrated *in vacuo*, giving the crude iodide as a yellow oil. Major impurities were removed by flash column chromatography (33% EtOAc in heptane v/v). The protected iodide was suspended in MeOH (15 mL), and 25% aq. NaOH solution (2 mL) was added. The reaction was gently warmed until a clear solution was obtained and then left to stir for approximately 2 h until TLC analysis showed complete deprotection of the amine. The reaction was concentrated *in vacuo* and partitioned between a mixture of EtOAc, DCM, and H_2_O (100 mL) (1:1:2, v/v). The aqueous phase was further extracted with DCM (2 × 50 mL). The combined organic phases were washed with H_2_O (50 mL) and brine (50 mL), dried over MgSO_4_, then filtered, and concentrated *in vacuo* to give the pure iodide (471 mg, 71%) as clear oil. Analytical amounts of the racemic mixture were separated and isolated as the two individual enantiomers as their hydrochloride salts using general procedure B-1, method 1, using an isocratic gradient of 30% MP B. Enantiomer 1: Rt 6.95, Enantiomer 2: Rt 10.163. MP 252–255 °C; TLC *R*_f_ = 0.15 (5% TEA and 10% MeOH in EtOAc v/v/v); ^1^H NMR (400 MHz, CDCl_3_) δ 7.38 (s, 1H), 6.87 (s, 1H), 3.85 (s, 3H), 3.84 (s, 3H), 3.48–3.38 (m, 3H), 3.12 (t, *J* = 13.0 Hz, 1H), 3.06 (td, *J* = 11.2, 9.9, 2.2 Hz, 1H), 2.14–2.06 (m, 1H), 2.02–1.87 (m, 3H); ^13^C NMR (101 MHz, CDCl_3_) δ 154.58, 152.99, 131.38, 123.26, 111.92, 84.88, 57.66, 56.78, 48.94, 45.18, 35.40, 28.85, 24.03; HPLC *t*_R_ = 18.96 (Method A); HRMS *m*/*z* calculated for [C_13_H_18_INO_2_]+ (M + H) 348.0455 found 348.0453.

## Compound **10**

### *tert*-Butyl 3-(2,5-dimethoxyphenyl)piperidine-1-carboxylate

A flame-dried round-bottom flask, equipped with a stir bar, backfilled with argon gas, was charged with **7** (1 g, 3.87 mmol) and di-*tert*-butyl dicarbonate (931 mg, 4.26 mmol). The contents of the vessel were suspended in a mixture of TEA in DCM (1:10 v/v) (12 mL). The reaction was stirred at room temperature for 18 h. The reaction was monitored by TLC. Upon complete conversion to carboxylate, the reaction was concentrated *in vacuo*. Major impurities were removed by flash column chromatography (20% EtOAc in heptane v/v) to give the protected amine as a clear oil in quantitative yield. The product was deemed of sufficient purity for use in subsequent reactions and was not further purified. TLC *R*_f_ = 0.35 (20% EtOAc in heptane v/v); ^1^H NMR (400 MHz, CDCl_3_) δ 6.80 (d, *J* = 8.7 Hz, 1H), 6.75 (d, *J* = 3.0 Hz, 1H), 6.71 (dd, *J* = 8.7, 3.0 Hz, 1H), 4.17 (s, 2H), 3.80 (s, 3H), 3.77 (s, 3H), 3.11–2.96 (m, 1H), 2.70 (dd, *J* = 12.8, 11.2 Hz, 2H), 1.94 (d, J = 8.2 Hz, 1H), 1.80–1.68 (m, 1H), 1.67–1.53 (m, 2H), 1.46 (s, 9H); ^13^C NMR (101 MHz, CDCl_3_) δ 155.00, 153.79, 151.60, 133.33, 114.11, 111.58, 110.98, 79.34, 56.19, 55.85, 36.07, 32.04, 28.66, 25.75, 22.84, 14.26; HPLC *t*_R_ = 17.04 (Method A).

### *tert*-Butyl 3-(4-formyl-2,5-dimethoxyphenyl)piperidine-1-carboxylate

A flame-dried round-bottom flask, equipped with a stir bar, backfilled with argon gas, was charged with *tert*-butyl 3-(2,5-dimethoxyphenyl)piperidine-1-carboxylate (1.03 g, 3.2 mmol) and anhyd. DCM (7 mL). The reaction was cooled (−78 °C), and TiCl_4_ (0.87 mL, 8.0 mmol) was added followed by dichloromethyl methyl ether (0.82 mL 9.6 mmol) and then stirred for approximately 2 h while being monitored by TLC. Upon completion, the reaction was allowed to warm to 0 °C under stirring and then poured into ice water (50 mL). Ice was allowed to melt before the mixture was basified with sat. aq. NaHCO_3_ (100 mL), and drops of concentrated NaOH and phases were separated. The aqueous layer was further extracted with a mixture of EtOH and CHCl_3_ (1:2 v/v)(3 × 150 mL). The combined organic layers were dried over MgSO_4_, filtered, and concentrated *in vacuo* to give the crude product as a yellow solid. To ensure full protection of the amine, the crude product was suspended in a mixture of TEA in DCM (1:10 v/v) (10 mL), and di-*tert*-butyl dicarbonate (769 mg, 3.5 mmol) was added. The mixture was left to stir for 16 h. The reaction mixture was concentrated *in vacuo* and purified by repeated flash column chromatography (25% EtOAc in heptane). Two purifications gave the pure title compound as a clear oil (786 mg, 70%). TLC *R*_f_ = 0.3 (25% EtOAc in heptane v/v) (development: ninhydrin); ^1^H NMR (400 MHz, CDCl_3_) δ 10.40 (s, 1H), 7.29 (s, 1H), 6.82 (s, 1H), 4.26–4.01 (m, 2H), 3.89 (s, 3H), 3.83 (s, 3H), 3.12 (tt, *J* = 10.8, 3.7 Hz, 1H), 2.81 (s, 2H), 2.01–1.90 (m, 1H), 1.75 (d, *J* = 10.6 Hz, 1H), 1.67–1.56 (m, 2H), 1.46 (s, 9H); ^13^C NMR (101 MHz, CDCl_3_) δ 189.12, 156.77, 154.78, 151.40, 141.08, 123.14, 108.41, 79.46, 56.24, 55.85, 36.67, 31.87, 29.00, 28.47, 25.25, 22.68, 14.10.

### *tert*-Butyl 3-(4-cyano-2,5-dimethoxyphenyl)piperidine-1-carboxylate

A flame-dried round-bottom flask, equipped with a stir bar, backfilled with argon gas, was charged with of *tert*-butyl 3-(4-formyl-2,5-dimethoxyphenyl)piperidine-1-carboxylate (786 mg, 2.24 mmol), NaN_3_ (219 mg, 3.38 mmol), and MeCN(5 mL). Trifluoromethanesulfonic acid (0.59 mL, 6.75 mmol) was added dropwise over approximately 1 min. The reaction was stirred at room temperature for 3 min before being concentrated *in vacuo* and diluted with H_2_O (2 mL). The aqueous mixture was basified with sat. aq. NaHCO_3_ (5 mL) and drops of NaOH (≈pH 10). The basic aqueous suspension was extracted with a mixture of EtOH in CHCl_3_ (1:2)(3 × 50 mL). The combined organic phases were dried over MgSO_4_, filtered, and concentrated *in vacuo* to a brown gum. To ensure full protection of the amine, the crude product was suspended in a mixture of TEA in DCM (11 mL, 1:10 v/v) and di-*tert*-butyl dicarbonate (540 mg, 2.47 mmol) was added. The mixture was left under stirring for 16 h. The reaction mixture was concentrated *in vacuo* and purified by flash column chromatography (25% EtOAc in heptane) to give the pure nitrile as a white solid (298 mg, 38%). TLC *R*_f_ = 0.3 (25% EtOAc in heptane v/v) (development: ninhydrin); ^1^H NMR (400 MHz, CDCl_3_) δ 6.97 (s, 1H), 6.79 (s, 1H), 4.30–3.96 (m, 2H), 3.89 (s, 3H), 3.81 (s, 3H), 3.09 (ddt, *J* = 10.7, 7.3, 3.7 Hz, 1H), 2.80 (s, 2H), 1.98–1.88 (m, 1H), 1.74 (s, 1H), 1.69–1.54 (m, 3H), 1.46 (s, 10H); ^13^C NMR (101 MHz, CDCl_3_) δ 156.08, 154.89, 151.07, 139.55, 116.77, 114.55, 111.13, 99.27, 79.67, 56.62, 56.20, 36.61, 28.61, 27.56, 25.33.

### 2,5-Dimethoxy-4-(piperidin-3-yl)benzonitrile (**10**)

A round-bottom flask, equipped with a stir bar, was charged with *tert*-butyl 3-(4-cyano-2,5-dimethoxyphenyl)piperidine-1-carboxylate (150 mg. 0.44 mmol) and MeOH (5 mL). 4 M dioxanal HCl was gradually added over 2 h (1.7 mL, 6.8 mmol). The reaction was stirred on for additional 30 min. The reaction was monitored by TLC. Upon full conversion, additional Et_2_O was added until nucleation was observed and reaction was left to crystallize at −4 °C overnight, yielding the pure nitrile as the hydrochloride salt as off green crystals, which were isolated by decantation, then stripped of residual solvent *in vacuo*, and further dried under reduced pressure (78 mg, 62%). Analytical amounts of the racemic mixture were separated and isolated as the two individual enantiomers as their hydrochloride salts in quantitative yields using general procedure B-1, method 1, using an isocratic gradient of 30% MP B, enantiomer 1: Rt 8.527, enantiomer 2: Rt 11.860. MP 252–254 °C; TLC *R*_f_ = 0.1 (25% EtOAc in heptane v/v) (development: ninhydrin); ^1^H NMR (400 MHz, CDCl_3_) δ 7.27 (s, 1H), 7.10 (s, 1H), 3.97 (s, 3H), 3.90 (s, 3H), 3.61–3.41 (m, 3H), 3.19 (t, *J* = 12.3 Hz, 1H), 3.15–3.03 (m, 1H), 2.13 (dd, *J* = 10.0, 3.4 Hz, 1H), 1.98 (tdd, *J* = 16.3, 15.0, 6.7, 3.4 Hz, 3H); ^13^C NMR (101 MHz, CDCl_3_) δ 156.13, 150.75, 136.30, 115.68, 114.75, 111.26, 99.60, 55.83, 55.42, 43.77, 34.24, 27.30, 22.52; HPLC *t*_R_ = 9.75 (Method A). IR *v*_max_ (neat)/cm^–1^ 2225,11(CN); HRMS *m*/*z* calculated for [C_18_H_14_N_2_O_2_]+ (M + H) 247.1441, found 247.1446.

## Compound **11**

### 1,4-Dimethoxy-2-(trifluoromethyl)benzene

To a flame-dried round-bottom flask, backfilled with argon gas, containing a solution of sodium methoxide (40.52 g, 750 mmol) in anhydrous degassed DMSO (150 mL) was added 1-fluoro-4-methoxy-2-(trifluoromethyl)benzene (14.56 g, 75 mmol). The reaction mixture was stirred at 120 °C for 19 h until full consumption of starting material was observed by NMR. The reaction was quenched with ice water (700 mL), and organics were extracted with Et_2_O (3 × 200 mL). The combined organic phases were washed with H_2_O (2 × 200 mL), followed by brine (200 mL), then dried over MgSO_4_, filtered, and concentrated *in vacuo* to give the desired dimethoxybenzene as a clear oil. The oil crystallized into a solid over the course of several days and was of sufficiently high purity to use without further purification (15.21 g, 98%). TLC *R*_f_ = 0.45 (20% EtOAc in heptane v/v); ^1^H NMR (400 MHz, CDCl_3_) δ 7.12 (d, *J* = 3.1 Hz, 1H), 7.02 (dd, *J* = 9.0, 3.1 Hz, 1H), 6.94 (d, *J* = 9.0 Hz, 1H), 3.86 (s, 3H), 3.80 (s, 3H)); ^13^C NMR (151 MHz, CDCl_3_) δ 153.12, 151.70, 126.27, 124.47, 122.66, 120.85, 119.90, 119.69, 119.49, 119.28, 118.27, 113.77, 113.00, 112.96, 56.75, 56.06; HPLC *t*_R_ = 26.79 min (Method A).

### 1-Bromo-2,5-dimethoxy-4-(trifluoromethyl)benzene

A flame-dried round-bottom flask, backfilled with argon gas, was charged with 1,4-dimethoxy-2-(trifluoromethyl)benzene (5.15 g, 25 mmol) and anhydrous DCM (50 mL). The reaction solution was shielded from light and cooled on an ice bath before addition of TfOH (4.43 mL, 50 mmol). The reaction mixture was stirred for 2 min followed by the addition of 1,3-dibromo-5,5-dimethylhydantoin (3.57 g, 12.5 mmol) in one portion. The reaction mixture was stirred on for an additional 5 min before being allowed to warm to ambient temperature and stirred on for a total of 3 h. The reaction was then quenched by careful addition of sat. aq. Na_2_S_2_O_3_ (7 mL) followed by sat. aq. NaHCO_3_ (30 mL). The resulting biphasic system was separated, and the aqueous phase was further extracted with DCM (2 × 50 mL). The combined organic phases were washed with brine (50 mL), dried over Na_2_SO_4_, then filtered, and concentrated *in vacuo* to give a crude off-white solid that was dissolved in boiling isopropanol and allowed to cool to ambient temperature. Et_2_O was added dropwise until turbidity was observed, and then the reaction was allowed to stand at 4 °C overnight giving the desired bromide (4.63 g, 65%) as a colorless crystalline solid. TLC *R*_f_ = 0.6 (10% EtOAc in heptane v/v); ^1^H NMR (400 MHz, CDCl_3_) δ 7.23 (s, 1H), 7.09 (s, 1H), 3.88 (s, 3H), 3.87 (s, 3H); ^13^C NMR (151 MHz, CDCl_3_)) δ 151.77, 149.86, 123.21 (q, *J*_CF_ = 270.9 Hz), 118.51 (q, *J*_CF_ = 31.3), 118.23, 116.37, 110.86 (q, *J*_CF_ = 3.6 Hz), 57.16, 56.95; HPLC *t*_R_ = 28.64 min (Method A).

### 3-(2,5-Dimethoxy-4-(trifluoromethyl)phenyl)pyridine

To a flame-dried 20 mL microwave vial, backfilled with argon gas, was added 1-bromo-2,5-dimethoxy-4-(trifluoromethyl)benzene (909 mg, 3.1 mmol) followed by pyridin-3-ylboronic acid (762 mg, 6.2 mmol) and anhydrous, degassed 1,4-dioxane (3.5 mL). The mixture was further degassed for 10 min before addition of bis(triphenylphosphine)palladium(II) dichloride (109 mg, 0.155 mmol, 5 mol %) followed by 1 M solution of tri-*tert*-butylphosphine in toluene (0.155 mL, 0.155 mmol). Finally, a degassed 2 M aq. solution of Na_2_CO_3_ (3.1 mL, 6.2 mmol) was added before sealing the reaction vial. The reaction mixture was heated at 120 °C using microwave irradiation for 80 min. The reaction was monitored by TLC. Upon complete consumption of the bromide, the reaction mixture was diluted with EtOAc (7 mL) and transferred to a separation funnel containing EtOAc (10 mL) and H_2_O (20 mL). Phases were separated, and the aqueous phase was further extracted with EtOAc (10 mL). The combined organic phases were washed with brine (20 mL), dried over MgSO_4_, filtered, and concentrated *in vacuo* to mixed resins and solids. The crude product was immediately purified by flash column chromatography (40%, EtOAc in heptane), giving the desired phenylpyridine as an off-white solid (727 mg, 83%). TLC *R*_f_ = 0.18 (40% EtOAc in heptane v/v); ^1^H NMR (400 MHz, CDCl_3_)) δ 8.75 (d, *J* = 2.1 Hz, 1H), 8.60 (dd, J = 4.9, 1.7 Hz, 1H), 7.86 (dt, *J* = 7.9, 2.0 Hz, 1H), 7.40–7.31 (m, 1H), 7.19 (s, 1H), 6.97 (s, 1H), 3.90 (s, 3H), 3.80 (s, 3H); ^13^C NMR (151 MHz, CDCl_3_) δ 151.76, 150.14, 150.05, 148.88, 136.88, 133.22, 131.63, 123.49(q, *J*_CF_ = 273.6 Hz), 123.13, 118.99 (q, *J*_CF_ = 31.3 Hz), 115.28, 110.76 (q, *J*_CF_ = 5.4 Hz), 56.85, 56.49; HPLC *t*_R_ = 20.23 (Method B).

### 3-(2,5-Dimethoxy-4-(trifluoromethyl)phenyl)piperidine Hydrochloride (**11**)

This was synthesized according to general procedure D using 3-(2,5-dimethoxy-4-(trifluoromethyl)phenyl)pyridine (4 g, 14.12 mmol). The hydrochloride salt was prepared by dissolving the product in a minimum amount of Et_2_O and treating the solution with 4 M dioxanal HCl. The precipitate was isolated by decantation and redissolved in the minimal amount of MeOH. Et_2_O was added dropwise until nucleation was observed, and the solution was allowed to crystallize at −4 °C overnight giving the pure title compound as a white solid (2.82 g, 69%). The racemic mixture was then separated and isolated as the two individual enantiomers as their hydrochloride salts using general procedure B-1, method 1, using an isocratic gradient of 10% MP B, enantiomer 1: Rt 7.22, enantiomer 2: Rt 11.247. Chiral resolution was also achieved using enantiomeric separation methods 2 and 3. MP 239–241 °C; TLC *R*_f_ = 0.3 (5% TEA and 10% MeOH in EtOAc v/v/v); ^1^H NMR (400 MHz, CDCl_3_) δ 7.19 (s, 1H), 7.12 (s, 1H), 3.92 (s, 3H), 3.90 (s, 3H), 3.59–3.42 (m, 3H), 3.20 (t, *J* = 12.3 Hz, 1H), 3.15–3.04 (m, 1H), 2.16–2.07 (m, 1H), 2.05–1.88 (m, 3H); ^13^C NMR (151 MHz, CDCl_3_) δ 153.18, 151.73, 135.59, 124.92 (q, *J* = 271.6 Hz), 118.81 (q, *J* = 31.3 Hz), 113.75, 110.62 (q, *J* = 5.4 Hz), 57.22, 56.73, 45.19, 35.41, 28.84, 23.98; HPLC *t*_R_ = 11.75 (Method B); HRMS *m*/*z* calculated for [C_14_H_19_F_3_NO_2_]+ (M + H) 290.1362, found 290.1377.

## Compound **12**

### 3-(2,5-Dimethoxy-4-methylphenyl)pyridine

The title compound was prepared according to general procedure E starting from 1-bromo-2,5-dimethoxy-4-methylbenzene (277 mg, 1.20 mmol) giving 263 mg (95%) of the title compound. ^1^H NMR (300 MHz, CDCl_3_) δ: 8.77 (s, 1H); 8.54 (d, *J* = 4.8 Hz, 1H); 7.87 (dt, *J* = 7.9, 1.9 Hz, 1H); 7.33 (dd, *J* = 7.9, 4.8 Hz, 1H); 6.84 (s, 1H); 6.80 (s, 1H); 3.83 (s, 3H); 3.76 (s, 3H), 2.28 (s, 3H). MS: *m*/*z* 230 [M + H]^+^.

### 3-(2,5-Dimethoxy-4-methylphenyl)piperidine Hydrochloride (**12**)

The title compounds were prepared according to general procedure F2 starting from 3-(2,5-dimethoxy-4-methylphenyl)pyridine. The individual enantiomers were transformed to the corresponding Boc-protected amines using general procedure G and separated using general procedure B-2, condition 1, enantiomer 1: Rt 9.38 min (50 mg, 39%), enantiomer 2: Rt 12.84 min (40 mg, 45%). The Boc-protected amines were deprotected as the corresponding hydrochlorides using general procedure H giving the title compounds in quantitative yields. ^1^H NMR (400 MHz, CD_3_OD) 6.81 (s, 1H); 6.76 (s, 1H); 3.80 (s, 3H); 3.79 (s, 3H); 3.45–3.33 (m, 3H); 3.10–2.97 (m, 2H); 2.17 (s, 3H); 2.10–2.02 (m, 1H); 1.98–1.84 (m, 3H). ^13^C NMR (100 MHz, CD_3_OD, *one signal overlapping with CD*_*3*_*OD*) δ: 153.4, 152.0, 127.6, 127.5, 115.2, 110.9, 56.6, 56.5, 45.2, 35.3, 29.1, 24.1, 16.2. MS: *m*/*z* 336 [M + H]^+^.

## Compound **13**

### 4-Ethyl-4-methoxycyclohexa-2,5-dien-1-one

The title compound was prepared as described by Xie and coworkers. All analytical data were in congruence with literature values.^44^

### 2-Bromo-5-ethyl-4-methoxyphenol

The title compound was prepared according to general procedure I starting from known 3-ethyl-4-methoxyphenol (2.083 g, 13.687 mmol) giving 1.810 g (57%) of the desired product with minor impurities. The product was deemed pure enough for subsequent reactions and was not purified further ^1^H NMR (300 MHz, CDCl_3_) δ: 6.88 (s, 1H), 6.84 (s, 1H), 3.76 (s, 3H), 2.55 (q, *J* = 6.1 Hz, 2H), 1.16 (t, *J* = 6.1 Hz, 1H).

### 1-Bromo-4-ethyl-2,5-dimethoxybenzene

The title compound was prepared according to general procedure J starting from 2-bromo-5-ethyl-4-methoxyphenol (1.46 g, 6.318 mmol) giving 400 g (26%) of the desired product with minor impurities. The product was deemed pure enough for subsequent reactions and was not purified further. ^1^H NMR (300 MHz, CDCl_3_) δ: 7.04 (s, 1H), 6.78 (s, 1H), 3.88 (s, 3H), 3.81 (s, 3H), 2.62 (q, *J* = 6.1 Hz, 2H), 1.21 (t, *J* = 6.1 Hz, 3H).

### 3-(4-Ethyl-2,5-dimethoxyphenyl)pyridine

The title compound was prepared according to general procedure E starting from 1-bromo-4-ethyl-2,5-dimethoxybenzene (400 mg, 1.632 mmol) giving 163 mg (41%) of the desired product with minor impurities. The crude product was deemed pure enough for subsequent reactions. ^1^H NMR (300 MHz, CDCl_3_) δ: 8.78 (s, 1H); 8.55 (s, 1H); 7.87 (d, *J* = 8.0 Hz, 1H); 7.39–7.28 (m, 1H); 6.85 (s, 1H); 6.82 (s, 1H); 3.83 (s, 3H); 3.77 (s, 3H); 2.69 (q, *J* = 7.5 Hz, 2H); 1.24 (t, *J* = 7.5 Hz, 3H). MS: *m*/*z* 244 [M + H]^+^.

### 3-(4-Ethyl-2,5-dimethoxyphenyl)piperidine Hydrochloride (**13**)

The title compounds were prepared according to general procedure F2 starting from 3-(4-ethyl-2,5-dimethoxyphenyl)pyridine. The individual enantiomers were transformed to the corresponding Boc-protected amines using general procedure G and separated using general procedure B-2, condition 1, enantiomer 1: Rt 11.35 min (32 mg, 49%), enantiomer 2: Rt 14.10 min (32 mg, 49%). The Boc-portected amines were deprotected as the corresponding hydrochlorides using general procedure H giving the title compounds in quantitative yields. ^1^H NMR (400 MHz, MeOD) δ 6.80 (s, 1H), 6.78 (s, 1H), 3.81 (s, 3H), 3.79 (s, 3H), 3.46–3.33 (m, 3H), 3.12–2.97 (m, 2H), 2.60 (q, *J* = 7.5 Hz, 2H), 2.11–2.01 (m, 1H), 1.98–1.83 (m, 3H), 1.15 (t, *J* = 7.5 Hz, 3H). ^13^C NMR (100 MHz, MeOD; *one signal overlapping with CD*_*3*_*OD*) δ 153.0, 152.2, 133.7, 127.7, 113.8, 111.3, 56.6, 56.5, 45.2, 35.3, 29.1, 24.4, 24.1, 14.9. MS: *m*/*z* 250 [M + H]^+^.

## Compound **14**

### 4-Butyl-4-methoxycyclohexa-2,5-dien-1-one

The title compound was prepared as described by Xie and coworkers. All analytical data were in congruence with literature values.^44^

### 2-Bromo-5-butyl-4-methoxyphenol

The title compound was prepared according to general procedure I starting from known 3-butyl-4-methoxyphenol (1.15 g, 6.380 mmol) giving 934 mg (56%) of the desired product with minor impurities. The crude product was deemed pure enough for subsequent reactions. ^1^H NMR (400 MHz, CDCl_3_) δ 6.88 (s, 1H), 6.82 (s, 1H), 3.76 (s, 3H), 2.53 (t, *J =* 10.0 Hz, 2H), 1.58–1.47 (m, 2H), 1.34 (dd, *J* = 15.1, 7.3 Hz, 2H), 0.91 (t, *J* = 8.0 Hz, 3H).

### 1-Bromo-4-butyl-2,5-dimethoxybenzene

The title compound was prepared according to general procedure J starting from 2-bromo-5-butyl-4-methoxyphenol (934 mg, 3.604 mmol) giving 343 g (35%) of the desired product with minor impurities. The crude product was deemed pure enough for subsequent reactions. ^1^H NMR (300 MHz, CDCl_3_) δ 7.01 (s, 1H), 6.73 (s, 1H), 3.84 (s, 3H), 3.77 (s, 3H), 2.59–2.53 (m, 2H), 1.59–1.49 (m, 2H), 1.36 (dd, *J* = 15.3, 7.2 Hz, 2H), 0.93 (t, *J* = 7.3 Hz, 3H).

### 3-(4-Butyl-2,5-dimethoxyphenyl)pyridine

The title compound was prepared according to general procedure E starting from 1-bromo-4-butyl-2,5-dimethoxybenzene (340 mg, 1.245 mmol) giving 308 mg (91%) of the desired product with minor impurities. The crude product was deemed pure enough for subsequent reactions. ^1^H NMR (300 MHz, CDCl_3_) δ: 8.78 (s, 1H); 8.54 (d, *J* = 4.0 Hz, 1H); 7.92 (d, *J* = 7.9 Hz, 1H); 7.36 (dd, *J* = 7.9, 4.9 Hz, 1H); 6.82 (s, 1H); 6.81 (s, 1H); 3.82 (s, 3H); 3.77 (s, 3H); 2.69–2.60 (m, 2H); 1.67–1.54 (m, 2H); 1.48–1.33 (m, 2H); 0.96 (t, *J* = 7.3 Hz, 3H). MS: *m*/*z* 272 [M + H]^+^.

### 3-(4-Butyl-2,5-dimethoxyphenyl)piperidine Hydrochloride (**14**)

The title compound was prepared according to general procedure F2 starting from *tert*-butyl 3-(4-butyl-2,5-dimethoxyphenyl)piperidine-1-carboxylate. The individual enantiomers were transformed into the corresponding Boc-portected amines using general procedure G and separated using general procedure B-2, condition 2, enantiomer 1: Rt 8.08 min (90, 48% mg), enantiomer 2: Rt 11.15 min (100 mg, 52%). The Boc-protected amines were deprotected as the corresponding hydrochlorides using general procedure H, giving the title compounds in quantitative yield. ^1^H NMR (400 MHz, MeOD) δ 6.78 (s, 2H), 3.80 (s, 3H), 3.79 (s, 3H), 3.45–3.34 (m, 3H), 3.10–2.99 (m, 2H), 2.60–2.55 (m, 2H), 2.10–2.04 (m, 1H), 1.97–1.85 (m, 3H), 1.57–1.49 (m, 2H), 1.38–1.29 (m, 2H), 0.93 (t, *J* = 7.3 Hz, 3H). ^13^C NMR (100 MHz, MeOD) δ 153.1, 152.0, 132.3, 127.7, 114.4, 111.3, 56.6, 56.5, 45.2, 35.2, 33.5, 30.9, 24.1, 23.6, 14.3. MS: *m*/*z* 278 [M + H]^+^.

## Compound **15**

### (4-Bromo-2,5-dimethoxyphenyl)(methyl)sulfane

The title compound was prepared according to general procedure K starting from 1,4-dibromo-2,5-dimethoxybenzene (500 mg, 1.689 mmol) giving 860 mg (49%) of the desired product with minor impurities. The crude product was deemed pure enough for subsequent reactions. ^1^H NMR (300 MHz, CDCl_3_) δ 7.01 (s, 1H), 6.78 (s, 1H), 3.87 (s, 3H), 3.85 (s, 3H), 2.44 (s, 3H).

### 3-(2,5-Dimethoxy-4-(methylthio)phenyl)pyridine

The title compound was prepared according to general procedure E starting from (4-bromo-2,5-dimethoxyphenyl)(methyl)sulfane (568 mg, 2.158 mmol) giving 351 mg (62%) of the desired product with minor impurities. The crude product was deemed pure enough for subsequent reactions. ^1^H NMR (400 MHz, CDCl_3_) δ: 8.76 (dd, *J* = 2.3, 0.9 Hz, 1H); 8.55 (dd, *J* = 4.8, 1.7 Hz, 1H); 7.85 (ddd, *J* = 7.9, 2.3, 1.7 Hz, 1H); 7.32 (ddd, *J* = 7.9, 4.8, 0.9 Hz, 1H); 6.86 (s, 1H); 6.80 (s, 1H); 3.90 (s, 3H); 3.79 (s, 3H); 2.50 (s, 3H). MS: *m*/*z* 262 [M + H]^+^.

### 3-(2,5-Dimethoxy-4-(methylthio)phenyl)piperidine Hydrochloride (**15**)

The title compounds were prepared according to general procedure F2 starting from 3-(2,5-dimethoxy-4-(methylthio)phenyl)pyridine (350 mg, 1.339 mmol). The crude material obtained after filtration of the catalyst and evaporation was purified by flash column chromatography (10% MeOH in EtOAc + 5% Et_3_N). The individual enantiomers were transformed to the corresponding Boc-protected amines using general procedure G and separated using general procedure B-2, condition 3, enantiomer 1: Rt 16.70 min (35 mg, 10%). Enantiomer 2: Rt 21.75 min (35 mg, 10%). Both enantiomers were further converted to the corresponding hydrochlorides using general procedure H in quantitative yields. ^1^H NMR (400 MHz, MeOD) δ: 6.83 (s, 1H), 6.81 (s, 1H), 3.85 (s, 3H), 3.82 (s, 3H), 3.45–3.34 (m, 3H), 3.10–2.98 (m, 2H), 2.41 (s, 3H), 2.10–2.03 (m, 1H), 1.97–1.84 (m, 3H). ^13^C NMR (100 MHz, MeOD) δ 152.9, 152.3, 128.5, 127.1, 111.6, 111.2, 57.2, 56.7, 49.3, 45.2, 35.1, 29.1, 24.1, 14.8. MS: *m*/*z* 268 [M + H]^+^.

## Compound **16**

### (4-Bromo-2,5-dimethoxyphenyl)(ethyl)sulfane

The title compound was prepared according to general procedure K starting from 1,4-dibromo-2,5-dimethoxybenzene (1.50 mg, 5.068 mmol), giving 610 mg (43%) of the desired product with minor impurities. The crude product was deemed pure enough for subsequent reactions.

### 3-(4-(Ethylthio)-2,5-dimethoxyphenyl)pyridine

The title compound was prepared according to general procedure E starting from (4-bromo-2,5-dimethoxyphenyl)(ethyl)sulfane (589 mg, 2.125 mmol), giving 407 mg (69%) of the desired product with minor impurities. The crude product was progressed to subsequent reactions without further purification. MS: *m*/*z* 276 [M + H]^+^.

### 3-(2,5-Dimethoxy-4-(ethylthio)phenyl)piperidine Hydrochloride (**16**)

The title compounds were prepared according to general procedure F2 starting from 3-(2,5-dimethoxy-4-(ethylthio)phenyl)pyridine (407 mg, 1.478 mmol). The crude material obtained after filtration of the catalyst and evaporation was purified by flash column chromatography with (10% MeOH in EtOAc + 5% Et_3_N) a mobile phase. The individual enantiomers were transformed to the corresponding Boc-protected amines using general procedure G and separated using general procedure B-2, condition 4, enantiomer 1: Rt 14.92 min (45 mg, 11%). Enantiomer 2: Rt 19.30 (59 mg, 14%). Both enantiomers were further converted to the corresponding hydrochlorides using general procedure H in quantitative yields. ^1^H NMR (400 MHz, MeOD) δ 6.91 (s, 1H), 6.83 (s, 1H), 3.83 (s, 3H), 3.83 (s, 3H), 3.45–3.33 (m, 3H), 3.12–2.97 (m, 2H), 2.91 (q, *J =* 7.4 Hz, 2H), 2.11–2.02 (m, 1H), 1.99–1.82 (m, 3H), 1.26 (t, *J =* 7.4 Hz, 3H). ^13^C NMR (100 MHz, MeOD, *one signal overlapping with CD*_*3*_*OD*) δ 153.4, 152.5, 128.4, 125.9, 114.1, 112.1, 57.1, 56.7, 45.2, 35.2, 29.0, 27.0, 24.1, 14.69. MS: *m*/*z* 282 [M + H]^+^.

## Compound **17**

### (4-Bromo-2,5-dimethoxyphenyl)(isopropyl)sulfane

The title compound was prepared according to general procedure K starting from 1,4-dibromo-2,5-dimethoxybenzene (1.50 mg, 5.068 mmol) giving 794 mg (54%) of the desired product with minor impurities. The crude product was progressed to subsequent reactions without further purification. MS: *m*/*z* 232 [M + H] ^+^

### 3-(4-(Isopropylthio)-2,5-dimethoxyphenyl)pyridine

The title compound was prepared according to general procedure E starting from (4-bromo-2,5-dimethoxyphenyl)(isopropyl)sulfane (789 mg, 2.709 mmol), giving 589 mg (75%) of the desired product with minor impurities. The crude product was progressed to subsequent reactions without further purification. MS: *m*/*z* 290 [M + H]^+^.

### 3-(4-(Isopropylthio)-2,5-dimethoxyphenyl)piperidine (**17**)

The title compounds were prepared according to general procedure F2 starting from 3-(4-(isopropylthio)-2,5-dimethoxyphenyl)pyridine (590 mg, 2.039 mmol). The crude material obtained after filtration of the catalyst and evaporation was purified by flash column chromatography with a MeOH/EtOAc (+5% Et_3_N) mobile phase. The individual enantiomers were transformed to the corresponding Boc-protected amines using general procedure G and separated using general procedure B-2, condition 4, eEnantiomer 1: Rt 13.01 min (41 mg, 7%). Enantiomer 2: Rt 18.13 min (40 mg, 7%). Both enantiomers were further converted to the corresponding hydrochlorides using general procedure H giving the title compounds in quantitative yields. ^1^H NMR (400 MHz, MeOD) δ 6.97 (s, 1H), 6.85 (s, 1H), 3.82 (s, 3H), 3.81 (s, 3H), 3.49 (p, *J =* 6.6 Hz, 1H), 3.44–3.31 (m, 3H), 3.12–2.96 (m, 2H), 2.10–2.01 (m, 1H), 1.99–1.82 (m, 3H), 1.22 (s, 3H), 1.21 (s, 3H). ^13^C NMR (100 MHz, MeOD, *one signal overlapping with CD*_*3*_*OD*) δ 154.6, 152.2, 129.8, 124.6, 117.2, 112.3, 57.2, 56.6, 45.2, 37.4, 35.2, 29.0, 24.1, 23.3. MS: *m*/*z* 296 [M + H]^+^.

## Compound **18**

### 2-(2,5-Dimethoxy-4-(trifluoromethyl)phenyl)ethan-1-amine Hydrochloride

The title compound was prepared as previously described by Frescas et al.^21^ All analytical data were in congruence with reported literature values.

## Compound **19**

### (*R*)-3-(2,5-Dimethoxy-4-(trifluoromethyl)phenyl)-1-methylpiperidine (**19**_dis_)

The title compound was prepared according to general procedure L starting from (*R*)-**11** (34 mg, 0.104 mmol) and formalin, giving 27 mg (85%) of the desired compound. ^1^H NMR (400 MHz, MeOD) δ 7.17 (s, 1H), 7.07 (s, 1H), 3.89 (s, 3H), 3.87 (s, 3H), 3.61–3.47 (m, 3H), 3.20 (t, *J* = 12.8 Hz, 1H), 3.06 (t, *J* = 12.3 Hz, 1H), 2.92 (s, 3H), 2.18–2.08 (m, 1H), 2.02–1.83 (m, 3H). ^13^C NMR (100 MHz, MeOD) δ 153.2, 151.7, 135.0, 124.9 (q, *J* = 271.7 Hz), 119.0 (q, *J* = 31.1 Hz), 113.7, 110.6 (q, *J* = 5.5 Hz), 58.7, 57.2, 56.7, 55.6, 44.3, 36.2, 28.0, 24.7. MS (*m*/*z*): 304 [M + H]^+^.

### (*S*)-3-(2,5-Dimethoxy-4-(trifluoromethyl)phenyl)-1-methylpiperidine (**19**_eu_)

The title compound was prepared according to general procedure L starting from (*S*)-**11** (40 mg, 0.123 mmol) and formalin, giving 35 mg (94%) of the desired compound. ^1^H NMR (400 MHz, MeOD) δ 7.17 (s, 1H), 7.08 (s, 1H), 3.89 (s, 3H), 3.87 (s, 3H), 3.62–3.47 (m, 3H), 3.21 (t, *J* = 12.6 Hz, 1H), 3.06 (t, *J* = 12.0 Hz, 1H), 2.92 (s, 3H), 2.18–2.08 (m, 1H), 2.03–1.83 (m, 3H). ^13^C NMR (100 MHz, MeOD) δ 153.2, 151.7, 135.0, 124.9 (q, *J* = 271.5 Hz), 119.0 (q, *J* = 31.0 Hz), 113.7, 110.7 (q, *J* = 5.3 Hz), 58.7, 57.2, 56.7, 55.5, 44.3, 36.2, 28.1, 24.7. MS (*m*/*z*): 304 [M + H]^+^.

## Compound **20**

### (*R*)-3-(2,5-Dimethoxy-4-(trifluoromethyl)phenyl)-1-ethylpiperidine (**20**_dis_)

The title compound was prepared according to general procedure L starting from (*R*)-**11** (60 mg, 0.184 mmol) and acetaldehyde, giving 54 mg (92%) of the desired compound. ^1^H NMR (400 MHz, MeOD) δ 7.16 (s, 1H), 7.10 (s, 1H), 3.89 (s, 3H), 3.87 (s, 3H), 3.68–3.52 (m, 3H), 3.23 (q, *J* = 7.3 Hz, 2H), 3.17 (t, *J* = 12.3 Hz, 1H), 3.01 (t, *J* = 12.5 Hz, 1H), 2.19–2.10 (m, 1H), 2.05–1.88 (m, 3H), 1.38 (t, *J* = 7.3 Hz, 3H). ^13^C NMR (100 MHz, MeOD) δ 153.2, 151.7, 135.2, 124.9 (q, *J* = 271.6 Hz), 118.9 (q, *J* = 31.1 Hz), 113.8, 110.7 (q, *J* = 5.3 Hz), 57.2, 56.8, 56.6, 53.7, 53.2, 36.0, 28.6, 24.5, 9.6. MS (*m*/*z*): 318 [M + H]^+^.

### (*S*)-3-(2,5-Dimethoxy-4-(trifluoromethyl)phenyl)-1-ethylpiperidine (**20**_eu_)

The title compound was prepared according to general procedure L starting from (*S*)-**11** (60 mg, 0.184 mmol) and acetaldehyde, giving 57 mg (97%) of the desired compound. ^1^H NMR (400 MHz, MeOD) δ 7.16 (s, 1H), 7.10 (s, 1H), 3.89 (s, 3H), 3.87 (s, 3H), 3.67–3.52 (m, 3H), 3.23 (q, *J* = 7.3 Hz, 2H), 3.16 (t, *J* = 12.4 Hz, 1H), 3.01 (t, *J* = 12.4 Hz, 1H), 2.19–2.10 (m, 1H), 2.04–1.89 (m, 3H), 1.38 (t, *J* = 7.3 Hz, 3H). ^13^C- NMR (100 MHz, MeOD) δ 153.2, 151.7, 135.1, 124.9 (q, *J* = 271.5 Hz), 118.9 (q, *J* = 31.1 Hz), 113.8, 110.7 (q, *J* = 5.3 Hz), 57.2, 56.8, 56.6, 53.7, 53.2, 36.0, 28.6, 24.5, 9.6. MS (*m*/*z*): 318 [M + H]^+^.

## Compound **21**

### 3-(4-(Trifluoromethyl)phenyl)pyridine

The title compound was prepared according to general procedure E starting from 1-bromo-4-(trifluoromethyl)benzene (270 mg, 1.20 mmol), giving 211 mg (79%) of desired product with minor impurities. The crude product was deemed pure enough for subsequent reactions.^1^H NMR (300 MHz, CDCl_3_) δ: 8.90 (s, 1H); 8.70 (d, *J* = 4.8 Hz, 1H); 7.90 (dt, *J* = 8.0, 1.9 Hz, 1H); 7.72 (m, 4H); 7.42 (dd, *J* = 7.9, 4.7 Hz, 1H). MS: *m*/*z* 224 [M + H]^+^.

### 3-(4-(Trifluoromethyl)phenyl)piperidine Hydrochloride (**21**)

The title compound was prepared according to general procedure F2 starting from 3-(4-(trifluoromethyl)phenyl)pyridine (138 mg, 0.6183 mmol). The crude residue was dissolved in Et_2_O (3 mL) and treated with 2 M HCl in Et_2_O (5 equiv). The resulting precipitate was filtered and recrystallized from Et_2_O/MeOH, giving 78 mg (47%) of the desired compound. ^1^H NMR (400 MHz, CDCl_3_) δ: 9.99 (broad s, 1H); 9.75 (broad s, 1H); 7.60 (d, *J* = 8.0 Hz, 2H); 7.33 (d, *J* = 8.0 Hz, 2H); 3.57 (d, *J* = 12.8 Hz, 2H); 3.32–3.39 (m, 1H); 2.84–2.96 (m, 2H); 2.03–2.23 (m, 3H); 1.63–1.73 (m, 1H). ^13^C NMR: (100 MHz, CDCl_3_) δ: 144.51, 129.97 (q, *J* = 32.6 Hz), 127.33, 125.91 (q, *J* = 3.8 Hz), 123.88 (q, *J* = 272.2 Hz), 48.88, 43.87, 39.37, 30.06, 22.49. MS: *m*/*z* 230 [M + H]^+^.

## Compound **22**

### 3-(2-Methoxy-4-(trifluoromethyl)phenyl)pyridine

The title compound was prepared according to general procedure E starting from 1-bromo-2-methoxy-4-(trifluoromethyl)benzene (500 mg, 1.960 mmol) giving 392 mg (79%) of desired product with minor impurities. The product was progressed to subsequent reactions without further purification. MS: *m*/*z* 254 [M + H]^+^.

### 3-(2-Methoxy-4-(trifluoromethyl)phenyl)piperidine Hydrochloride (**22**)

The title compounds were prepared according to general procedure F2 starting from 3-(2-methoxy-4-(trifluoromethyl)phenyl)pyridine. The enantiomers were transformed to the corresponding Boc-protected amines using general procedure G and separated using general procedure B-2, condition 1, enantiomer 1: Rt 9.11 min (22 mg, 31%), enantiomer 2: Rt 10.31 min (19 mg, 32%). The Boc-protected amines were deprotected as the corresponding hydrochlorides using general procedure H giving the title compounds in quantitative yields. ^1^H NMR (400 MHz, MeOD) δ 7.44 (d, *J* = 7.9 Hz, 1H), 7.27 (d, *J* = 8.0 Hz, 1H), 7.24 (s, 1H), 3.94 (s, 3H), 3.54–3.42 (m, 3H), 3.12–3.02 (m, 2H), 2.12–2.05 (m, 1H), 2.01–1.88 (m, 3H). ^13^C NMR (100 MHz, MeOD*; one signal overlapping with CD*_*3*_*OD*) δ 158.7, 134.5, 131.8 (q, *J* = 32.3 Hz), 128.9, 125.5 (q, *J* = 271.4 Hz), 118.7 (q, *J* = 4.0 Hz), 108.5 (q, *J* = 3.7 Hz), 56.4, 45.2, 35.0, 28.9, 24.0. MS: *m*/*z* 260 [M + H]^+^.

## Compound **23**

### 3-(3-Methoxy-4-(trifluoromethyl)phenyl)pyridine

The title compound was prepared according to general procedure E starting from 4-bromo-2-methoxy-1-(trifluoromethyl)benzene (500 mg, 1.96 mmol) giving 391 mg (79%) of desired product with minor impurities. The crude product was deemed pure enough for subsequent reactions. ^1^H NMR (300 MHz, CDCl_3_) δ: 8.86 (s, 1H); 8.66 (d, *J* = 4.7 Hz, 1H); 7.90 (d, *J* = 7.9 Hz, 1H); 7.67 (d, *J* = 8.0 Hz, 1H); 7.43 (dd, *J* = 7.9, 4.8 Hz, 1H); 7.20 (d, *J* = 8.1 Hz, 1H); 7.16 (s, 1H); 3.99 (s, 3H). MS: *m*/*z* 254 [M + H]^+^.

### 3-(3-Methoxy-4-(trifluoromethyl)phenyl)piperidine Hydrochloride (**23**)

The title compounds were prepared according to general procedure F2 starting from 3-(3-methoxy-4-(trifluoromethyl)phenyl)piperidine. The enantiomers were transformed to the corresponding Boc-protected amines using general procedure G and separated using general procedure B-2, condition 2, enantiomer 1: Rt 10.15 min (131 mg, 49%), enantiomer 2: Rt 13.22 min (122 mg, 45%). The Boc-protected amines were deprotected as the corresponding hydrochlorides using general procedure H giving the title compounds in quantitative yields. ^1^H NMR (400 MHz, MeOD) δ 7.54 (d, *J* = 8.0 Hz, 1H), 7.13 (s, 1H), 7.00 (d, *J* = 8.0 Hz, 1H), 3.93 (s, 3H), 3.47–3.43 (m, 2H), 3.19 (t, *J* = 12.1 Hz, 1H), 3.15–3.07 (m, 2H), 2.11–2.06 (m, 2H), 1.99–1.82 (m, 2H). ^13^C NMR (100 MHz, MeOD*; one signal overlapping with CD*_*3*_*OD*) δ 159.3, 148.9, 128.4 (q, *J* = 5.3 Hz), 125.1 (q, *J* = 271.3 Hz), 119.7, 118.8 (q, *J* = 31.1 Hz), 112.5, 56.6, 45.0, 41.6, 30.6, 23.8. MS: *m*/*z* 260 [M + H]^+^.

## Compound **24**

### 1-Bromo-2-ethoxy-5-methoxy-4-(trifluoromethyl)benzene

The title compound was prepared according to general procedure M starting from 2-bromo-4-methoxy-5-(trifluoromethyl)phenol (700 mg, 2.583 mmol) giving 767 mg (99%) of desired product with minor impurities. The crude product was deemed pure enough for subsequent reactions. ^1^H NMR (400 MHz, CDCl_3_) δ: 7.21 (s, 1H), 7.09 (s, 1H), 4.05 (q, *J* = 6.0 Hz), 1.46 (t, *J* = 6.0 Hz).

### 3-(2-Ethoxy-5-methoxy-4-(Trifluoromethyl)phenyl)pyridine

The title compound was prepared according to general procedure E starting from 1-bromo-2-ethoxy-5-methoxy-4-(trifluoromethyl)benzene (755 mg, 2.52 mmol) giving 540 mg (72%) of desired product with minor impurities. The product was deemed pure enough for subsequent reactions and was not purified further. ^1^H NMR (300 MHz, CDCl_3_) δ: 8.78 (dd, *J* = 2.3, 0.9 Hz, 1H), 8.60 (dd, *J* = 4.8, 1.7 Hz, 1H), 7.89 (ddd, *J* = 7.9, 2.3, 1.7 Hz, 1H), 7.36 (ddd, *J* = 7.9, 4.9, 0.9 Hz, 1H), 7.19 (s, 1H), 6.98 (s, 1H), 4.01 (q, *J* = 7.0 Hz, 2H), 3.90 (s, 3H), 1.32 (t, *J* = 7.0 Hz, 3H). MS: *m*/*z* 298 [M + H]^+^.

### 3-(2-Ethoxy-5-methoxy-4-(trifluoromethyl)phenyl)piperidine Hydrochloride (**24**)

The title compounds were prepared according to general procedure F2 starting from 3-(2-ethoxy-5-methoxy-4-(trifluoromethyl)phenyl)pyridine. The enantiomers were transformed to the corresponding Boc-protected amines using general procedure G and separated using general procedure B-2, condition 5, enantiomer 1: Rt 8.65 min (85 mg, 32%), enantiomer 2: Rt 9.43 min (91 mg, 32%). The Boc-protected amines were deprotected as the corresponding hydrochlorides using general procedure H giving the title compounds in quantitative yields. The hydrochorides were further analyzed using a Thermo Scientific Dionex 3000 UltiMate instrument connected to a Thermo Scientific Dionex 3000 diode array detector by a Phenomenex Lux 5 Amylose-2 (250 × 4.6 mm) chiral column with UV detection at 205, 210, 254, and 280 nm. MP A: 0.1% diethylamine in heptane (v/v). MP B: 0.1% diethylamine in EtOH (v/v). Flow rate: 10.0 mL/min using an isocratic gradient of 10% MP B to ensure correct stereochemistry. Enantiomer 1: Rt 5.600, enantiomer 2: Rt 6.31. ^1^H NMR (400 MHz, MeOD) δ 7.14 (s, 1H), 7.07 (s, 1H), 4.08 (q, *J* = 7.0 Hz, 2H), 3.88 (s, 3H), 3.55–3.39 (m, 3H), 3.17 (t, *J* = 12.3 Hz, 1H), 3.11–2.98 (m, 1H), 2.14–2.03 (m, 1H), 2.02–1.86 (m, 3H), 1.44 (t, *J* = 7.0 Hz, 3H). ^13^C NMR (100 MHz, MeOD; *one signal overlapping with CD*_*3*_*OD*) δ 153.1, 151.0, 135.8, 124.9 (q, *J* = 271.5 Hz), 118.8 (q, *J* = 31.0 Hz), 113.6, 111.7 (q, *J* = 5.4 Hz), 65.9, 57.2, 45.2, 35.4, 28.9, 24.0, 15.2. MS: *m*/*z* 304 [M + H]^+^.

## Compound **25**

### 2-Bromo-4-ethoxy-5-(trifluoromethyl)phenol

The title compound was prepared according to general procedure I starting from commercially available 4-ethoxy-3-(trifluoromethyl)phenol (2.00 g, 9.701 mmol) giving 2.60 g (94%) of desired product with minor impurities. The crude product was deemed pure enough for subsequent reactions. ^1^H NMR (400 MHz, CDCl_3_) δ 7.23 (s, 1H), 7.10 (s, 1H), 4.04 (q, *J* = 7.2 Hz, 2H), 1.42 (t, *J* = 7.2 Hz, 3H).

### 1-Bromo-5-ethoxy-2-methoxy-4-(trifluoromethyl)benzene

The title compound was prepared according to general procedure M starting from 2-bromo-4-ethoxy-5-(trifluoromethyl)phenol (1.14 g, 4.013 mmol) giving 1.07 g (89%) of desired product with minor impurities. The crude product was deemed pure enough for subsequent reactions. ^1^H NMR (400 MHz, CDCl_3_) δ: 7.22 (s, 1H), 7.08 (s, 1H), 4.07 (q, *J* = 6.8 Hz, 2H), 3.88 (s, 3H), 1.42 (t, *J* = 6.8 Hz, 3H).

### 3-(5-Ethoxy-2-methoxy-4-(trifluoromethyl)phenyl)pyridine

The title compound was prepared according to general procedure E starting from 1-bromo-5-ethoxy-2-methoxy-4-(trifluoromethyl)benzene (1.00 g, 3.343 mmol) giving 801 mg (81%) of desired product with minor impurities. The crude product was deemed pure enough for subsequent reactions. ^1^H NMR (400 MHz, CDCl_3_) δ 8.75 (s, 1H), 8.61 (s, 1H), 7.87 (dt, *J* = 8.0, 1.8 Hz, 1H), 7.38 (dd, *J* = 7.9, 4.8 Hz, 1H), 7.18 (s, 1H), 6.97 (s, 1H), 4.12 (q, *J* = 7.0 Hz, 2H), 3.80 (s, 3H), 1.44 (t, *J* = 7.0 Hz, 3H). MS: *m*/*z* 298 [M + H]^+^.

### 3-(5-Ethoxy-2-methoxy-4-(trifluoromethyl)phenyl)piperidine Hydrochloride (**25**)

The title compounds were prepared according to general procedure F2 starting from 3-(5-ethoxy-2-methoxy-4-(trifluoromethyl)phenyl)pyridine. The enantiomers were transformed to the corresponding Boc-protected amines using general procedure G and separated using general procedure B-2, condition 1, enantiomer 1: Rt 15.35 min (95 mg, 74%), enantiomer 2: Rt 26.79 min (90 mg, 77%). The Boc-protected amines were deprotected as the corresponding hydrochlorides using general procedure H, giving the title compounds in quantitative yields. The hydrochorides were further analyzed using a Thermo Scientific Dionex 3000 UltiMate instrument connected to a Thermo Scientific Dionex 3000 Diode Array Detector by a Phenomenex Lux 5 Amylose-2 (250 × 4.6 mm) chiral column with UV detection at 205, 210, 254, and 280 nm. MP A: 0.1% diethylamine in heptane (v/v). MP B: 0.1% diethylamine in EtOH (v/v). Flow rate: 10.0 mL/min using an isocratic gradient of 10% MP B to ensure correct stereochemistry. Enatiomer 1 (compound 20): Rt 5.300, enantiomer 2 (compound 19): Rt 5.970. ^1^H NMR (400 MHz, MeOD) δ: 7.15 (s, 1H); 7.05 (s, 1H); 4.12 (q, 2H, *J* = 7.0 Hz); 3.86 (s, 3H); 3.52–3.36 (m, 3H); 3.17–2.98 (m, 2H); 2.13–2.03 (m, 1H); 2.02–1.84 (m, 3H); 1.39 (t, 3H, *J* = 7.0 Hz). ^13^C NMR (100 MHz, MeOD; *one signal overlapping with CD*_*3*_*OD*) δ: 152.6, 151.7, 135.5, 124.9 (q, *J* = 271.6 Hz), 119.3 (q, *J* = 31.0 Hz), 114.8, 110.4 (q, *J* = 5.5 Hz), 66.5, 56.7, 45.2, 35.3, 28.8, 24.0, 15.1. MS: *m*/*z* 304 [M + H]^+^.

## Compound **26**

### 1-Bromo-2,5-diethoxy-4-(trifluoromethyl)benzene

The title compound was prepared according to general procedure M starting from 2-bromo-4-ethoxy-5-(trifluoromethyl)phenol (1.14 mg, 4.013 mmol) giving 1.18 mg (94%) of desired product with minor impurities. The crude product was deemed pure enough for subsequent reactions. ^1^H NMR (400 MHz, CDCl_3_) δ: 7.20 (s, 1H), 7.08 (s, 1H), 4.07 (qd, *J* = 7.0, 2.7 Hz, 4H) 1.44 (dt, *J* = 14.8, 7.0 Hz, 6H).

### 3-(2,5-Diethoxy-4-(trifluoromethyl)phenyl)pyridine

The title compound was prepared according to general procedure E starting from 1-bromo-2,5-diethoxy-4-(trifluoromethyl)benzene (1.12 mg, 3.577 mmol) giving 768 mg (69%) of desired product with minor impurities. The product was deemed pure enough for subsequent reactions and was not purified further. ^1^H NMR (400 MHz, CDCl_3_) δ: 8.78 (s, 1H), 8.60 (s, 1H), 7.90 (dt, *J* = 7.9, 1.9 Hz, 1H), 7.38 (dd, *J* = 7.9, 4.9 Hz, 1H), 7.18 (s, 1H), 6.97 (m, 1H), 4.12 (q, *J* = 7.0 Hz, 2H), 4.01 (q, *J* = 7.0 Hz, 2H), 1.43 (t, *J* = 7.0 Hz, 3H), 1.32 (t, *J* = 6.9 Hz, 3H). MS: *m*/*z* 312 [M + H]^+^.

### 3-(2,5-Diethoxy-4-(trifluoromethyl)phenyl)piperidine Hydrochloride (**26**)

The title compounds were prepared according to general procedure F2 starting from 3-(2,5-diethoxy-4-(trifluoromethyl)phenyl)pyridine. The enantiomers were transformed to the corresponding Boc-protected amines using general procedure G and separated using general procedure B-2, condition 1, enantiomer 1: Rt 7.03 min (45 mg, 34%), enantiomer 2: Rt 8.69 min (30 mg, 34%). The Boc-protected amines were deprotected as the corresponding hydrochlorides using general procedure H giving the title compounds in quantitative yields. The hydrochlorides were further analyzed using a Thermo Scientific Dionex 3000 UltiMate instrument connected to a Thermo Scientific Dionex 3000 diode array detector by a Phenomenex Lux 5 Amylose-2 (250 × 4.6 mm) chiral column with UV detection at 205, 210, 254, and 280 nm. MP A: 0.1% diethylamine in heptane (v/v). MP B: 0.1% diethylamine in EtOH (v/v). Flow rate: 10.0 mL/min using an isocratic gradient of 10% MP B to ensure correct stereochemistry. Enantiomer 1: Rt 5.850, Enantiomer 2: Rt 6.490. ^1^H NMR (400 MHz, MeOD) δ: 7.13 (s, 1H); 7.05 (s, 1H); 4.17–4.03 (m, 4H); 3.54–3.38 (m, 3H); 3.19–2.98 (m, 2H); 2.15–2.03 (m, 1H); 2.02–1.85 (m, 3H); 1.44 (t, 3H, *J* = 7.0 Hz); 1.39 (t, 3H, *J* = 7.0 Hz). ^13^C NMR (100 MHz, MeOD; *one signal overlapping with CD*_*3*_*OD*) δ: 152.5, 151.0, 135.6, 125.0 (q, *J* = 271.6 Hz), 119.3 (q, *J* = 31.0 Hz), 114.8, 111.5 (q, *J* = 5.4 Hz), 66.5, 65.9, 45.2, 35.4, 28.9, 24.0, 15.2, 15.1. MS: *m*/*z* 318 [M + H]^+^.

## Determination of Absolute Stereochemistry

**Figure 7:**
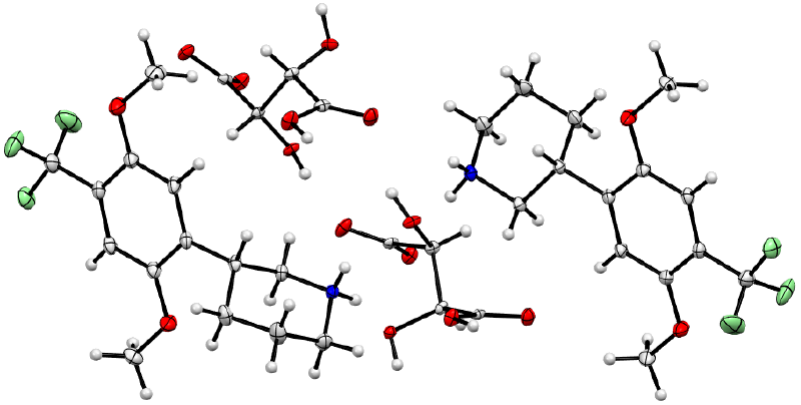
Perspective drawing of (*R*)-**11** as its (2*S,3S*)-tartaric acid salt (see Supporting Information for full experimental details on crystallography and structure elucidation). Displacement ellipsoids of the nonhydrogen atoms are shown at the 50% probability level. Hydrogen atoms have been shown as spheres of arbitrary size. Nitrogen atoms are in blue, fluorine atoms green, and oxygen atoms red.^52^

The distomer of **11** was crystallized as the d-tartaric acid salt yielding prismatic crystals allowing elucidation of its absolute configuration in reference to the known stereochemistry of the tartrate (Figure 7). The crystallized compound was identified as the (*R*) enantiomer of **11**. Thus, the eutomer of **11** was assigned to be the (*S*)-enantiomer.

The relation between relative retention times on chiral-HPLC and potency on 5-HT_2A_R and 5-HT_2C_R remains constant over the series, with the eutomer at the 5-HT_2A_R eluting first, followed by the distomer. Furthermore, the first eluting enantiomer also exhibited the lowest agonist activity at the 5-HT_2C_R. Based on this, we tentatively assign the absolute stereochemistry of the phenylpiperidine series to follow that of compound **11**, e.g., that the eutomers have the (*S*)-configuration and the distomers have the (*R*)-configuration.

## Crystallography

### X-Ray Crystallographic Analysis of the Distomer of Compound **11** in Complex with (2*S*,3*S*)-Tartaric Acid

Single crystals suitable for X-ray diffraction studies were grown from a solution in methanol. A single crystal was mounted and immersed in a stream of nitrogen gas [*T* = 123(1) K]. Data were collected, using graphite-monochromated MoKα radiation (λ = 0.71073 Å) on a Bruker D8 Venture diffractometer. Data collection and cell refinement were performed using the Bruker Apex2 Suite software.^53^ Data reduction using SAINT and multiscan correction for absorption using SADABS-2016-2 was performed within the Apex2 Suite.^54,55^ The crystal data, data collection, and the refinement data are given in Table S1 of this manuscript along with the structure solution and refinement of the deutomer to be (*R*)-**11** as salt with (2*S*,3*S*)-tartaric acid. Fractional atomic coordinates, a list of anisotropic displacement parameters, and a complete list of geometrical data have been deposited in the Cambridge Crystallographic Data Centre (CCDC 2321050).

## Pharmacology

### Ca^2+^ Imaging Assays

The functional characterization of the compounds at human 5-HT_2A_R and 5-HT_2C_R in a Ca^2+^/Fluo-4 assay was performed essentially as previously described.^41,42^ Stable 5-HT_2A_R- and 5-HT_2C_R-HEK293 cells were split into poly-d-lysine-coated black-walled 96-well plates with clear bottom (6 × 10^4^ cells/well).^41^ The following day, the culture medium was aspirated, and the cells were incubated in 50 μL of assay buffer Hank’s Buffered Saline Solution (HBSS) containing 20 mM HEPES, 1 mM CaCl_2_, 1 mM MgCl_2,_ and 2.5 mM probenecid, pH 7.4] supplemented with 6 mM Fluo–4/AM at 37 °C for 1 h. Then, the buffer was aspirated, the cells were washed once with 100 μL of assay buffer, and then 100 μL of assay buffer was added to the cells. The 96-well plate was assayed in a FLEXStation^3^ (Molecular Devices, Crawley, UK) measuring emission [in fluorescence units (FU)] at 525 nm caused by excitation at 485 nm before and up to 90 s after addition of 33.3 μL of compound solution in assay buffer. For compound testing in antagonist mode at 5-HT_2C_R, the compound was added with the assay buffer onto the cells and incubated for 5 min before assaying of the plate in the FLEXStation^3^ using 5-HT (EC_90_) as an agonist. The compound was characterized in duplicate at least three times at both cell lines.

The functional characterization of (*S*)-**11** at human 5-HT_2B_R in a Calcium No Wash^PLUS^ assay was performed by Eurofins. Briefly, 5-HT_2B_R-HEK293 cells were seeded in a total volume of 20 μL into black-walled, clear-bottoom, poly-d-lysine-coated 384-well microplates. On the day of the assay, the culture medium was aspirated and the cells were loaded with 20 μL of dye solution (1× dye loading buffer consisting of 1× dye, 1× additive A, and 2.5 mM probenecid in HBSS/20 mM HEPES) and incubated for 30–60 min at 37 °C. After this incubation, 10 μL of HBSS/20 mM HEPES was added to the wells, and the cells were incubated for 30 min at room temperature. The 384-well plate was assayed in a FLIPR-Tetra (Molecular Devices) measuring fluorescence over 2 min before and after addition of 10 μL of agonist solution (in HBSS/20 mM HEPES). The compound was characterized in duplicate three times at the cell line.

### Radioligand Binding Assays

The binding affinities of (*S*)-**11** at human 5-HT_2A_R, 5-HT_2B_R, and 5-HT_2C_R were determined in [^125^I]DOI competition binding assay by Eurofins. Membrane homogenates from HEK293 cells transfected with the three respective receptors were incubated for 60 min at 22 °C with 0.1 nM [^125^I]DOI in the absence or presence of the test compound in assay buffer (50 mM Tris-HCl, 5 mM MgCl_2_, 10 μM pargyline, 0.1% ascorbic acid, pH 7.4). Nonspecific binding was determined in the presence of 1 μM DOI. Following incubation, the samples were filtered rapidly under vacuum through glass fiber filters (GF/B, Packard) presoaked with 0.3% polyethylenimine and rinsed several times with ice cold wash buffer (50 mM Tris-HCl, pH 7.4) using a 96-sample cell harvester (Unifilter, Packard). The filters were dried and then counted for radioactivity in a scintillation counter (Topcounter, Packard) using a scintillation cocktail (Microscint-0, Packard). The compound was characterized in duplicate at least three times at each receptor.

The binding affinities of (*S*)-**11** at various other targets were estimated in radioligand binding competition assays by Eurofins. The assays were conventional competition binding assays performed with specific radioligands for the respective targets. Specific details for the assays are given in Table S4.

## Supporting Information Available

Full experimental details and additional figures and characterization; extended experimental details, copies of NMR spectra, HPLC chromatograms, and additional assay data (PDF)
Molecular formula strings (CSV)

## Abbreviations

2C-B: 4-bromo-2,5-dimethoxyphenethylamine
5-HT: 5-hydroxytryptamine (serotonin)
5-HT_2_: serotonin 2 class
5- HT_2A_R: serotonin 2A receptor
5-HT_2B_R: serotonin 2B receptor
5-HT_2C_R: serotonin 2C receptor
AcOH: acetic acid; Ca^2+^, calcium
CaCl_2_: calcium chloride
CNS: central nervous dystem
DBH: 1,3-dibromo-5,5-dimethylhydantoin
DCM: dichloromethane
DMPCA: (1*R*,2*S*)-2-(4-bromo-2,5-dimethoxyphenyl)cyclopropan-1-amine
DMSO: dimethyl sulfoxide;
DMT: *N,N*-dimethyltryptamine
DOB: 1-(4-bromo-2,5-dimethoxyphenyl)propan-2-amine
DOI: 1-(4-iodo-2,5-dimethoxyphenyl)propan-2-amine
EC_50_: half maximal effective concentration
Et_2_O: diethyl ether
EtO: ethoxy
EtOAc: ethyl acetate
H_2_: dihydrogen H_2_O, water HBSS, Hank’s buffered saline solution;
HCl: hydrochloric acid
HEPES: 4-(2-hydroxyethyl)-1-piperazineethanesulfonic acid
HPLC: high-performance liquid chromatography
IC_50_: half maximal inhibitory concentration
K_3_PO_4_: tripotassium phosphate
LE: ligand efficiency
LLE,: lipophilicity efficiency;
LSD: Lysergic acid diethylamide;
MeO: methoxy; MeOH, methanol
MgCl_2_: magnesium chloride
MgSO_4_: magnesium sulfate
Na_2_CO_3_: sodium carbonate
Na_2_SO_4_: sodium sulfate
NaHCO_3_: sodium bicarbonate
NaOH: sodium hydroxide
NaOMe: sodium methoxide
NBOMe: *N*-Benzyl phenethylamine
NMR: nuclear magnetic resonance spectroscopy
Pd(OH)_2_/C: palladium hydroxide on carbon, Pearlmans catalyst
PdCl_2_(dtbpf): [1,1′-bis(di-*tert*-butylphosphino)ferrocene]dichloropalladium(II)
PtO_2_: platinum dioxide, Adams catalyst
S.D.: standard deviation
SAR: structure–activity relationships
SnAr: Nucleophilic aromatic substitution
TCB-2: (*R*)-(3-Bromo-2,5-dimethoxybicyclo[4.2.0]octa-1,3,5-trien-7-yl)methanamine
TFM: trifluoromethyl
TfOH: triflic acid
TLC: thin-layer chromatography
TRIS-HCl: tris(hydroxymethyl)aminomethane hydrochloride
