# Supplementary material for: Discovery and Structure–Activity Relationships of 2,5-Dimethoxyphenylpiperidines as Selective Serotonin 5-HT2A Receptor Agonists
Source: J Med Chem. 2024 Apr 22;67(9):7224–44. doi: 10.1021/acs.jmedchem.4c00082 (PMC11089506; doi:10.1021/acs.jmedchem.4c00082)
Supplement: Supplementary file 1 — jm4c00082_si_001.pdf [file jm4c00082_si_001.pdf]

Supporting Information

Discovery and Structure-Activity Relationships of 2,5-dimethoxyphenylpiperidines as Selective Serotonin 5-HT_{2A} Receptor Agonists

Emil M. Rørsted,[a,b] Anders A. Jensen, [a,b] Gints Smits[c] Karla Frydenvang[b] and Jesper L. Kristensen[a,b,]*

[a]Lophora, 2920 Charlottenlund, Copenhagen, Denmark.

[b]Department of Drug Design and Pharmacology, University of Copenhagen, Universitetsparken 2, 2100 Copenhagen Ø, Denmark

E-mail:jesper.kristensen@sund.ku.dk

[c] Latvian Institute of Organic Synthesis, Aizkraukles 21, Riga, Latvia, LV-1006

Table of Contents

NMR Spectre for Compounds 4-17 & 19-26 3

HPLC Chromatograms for Compounds 5-10 26

Chiral HPLC Chromatograms for Compounds 5-6, 8-17 & 19-26 34

X-Ray Crystallography Experimental Details 52

Figure S1: Perspective drawing of Compound 11, enantiomer 2, (*R*)-11. 53

Table S1: Crystal Data for Compound (*R*)-11. 54

Table S2. Extended Functional Properties for Compounds 1 & 4-26 at 5-HT_{2A}R- and 5-HT_{2C}R. 55

Figure S2: Binding Properties Exhibited by (*S*)-11 at Human 5-HT_{2A}R, 5-HT_{2B}R, 5-HT_{2C}R. 58

Table S3: Binding Properties Exhibited by (*S*)-11 at Human 5-HT_{2A}R, 5-HT_{2B}R, 5-HT_{2C}R. 59

Table S4: Off-target screen of (*S*)-11. 60

Table S5: LogP determination of (*S*)-11. 64

Table S6: MDR1-MDCK II permeability assay. 65

References 66

NMR Spectre for Compounds 4-17 & 19-26

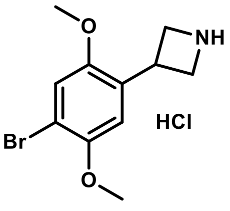

4

^{1}H-NMR (600 Mhz, methanol-d_4)

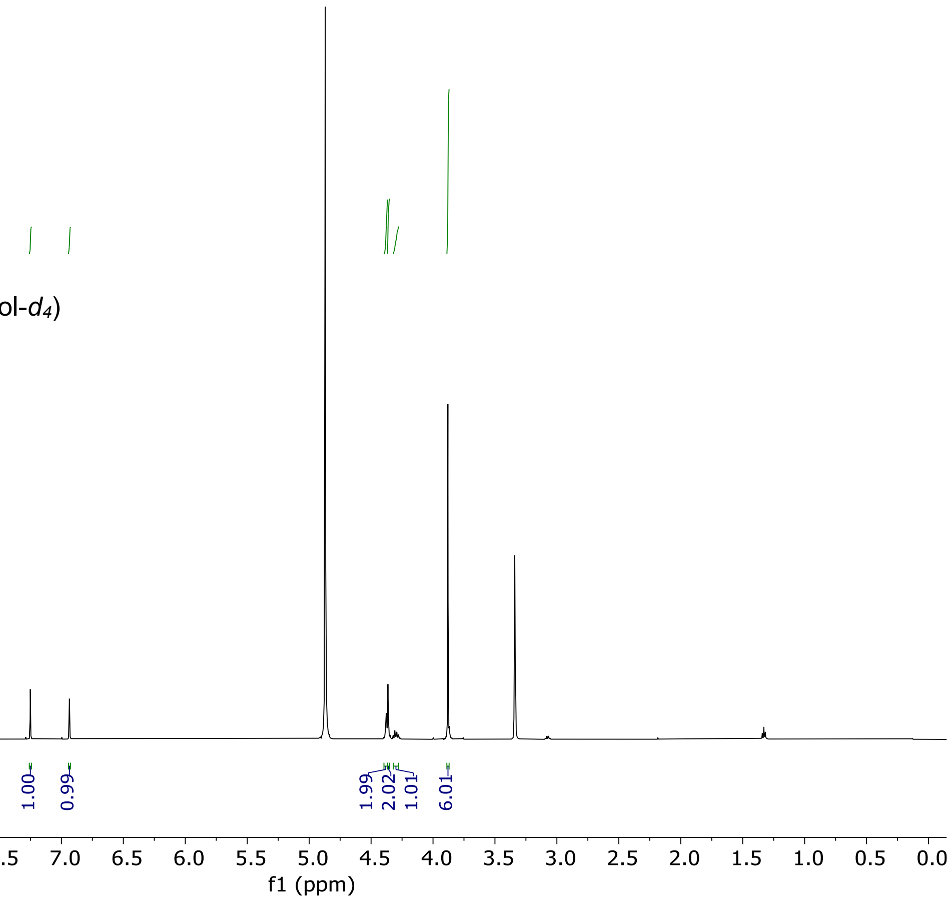

^{13}C-NMR (151 Mhz, methanol-d_4)

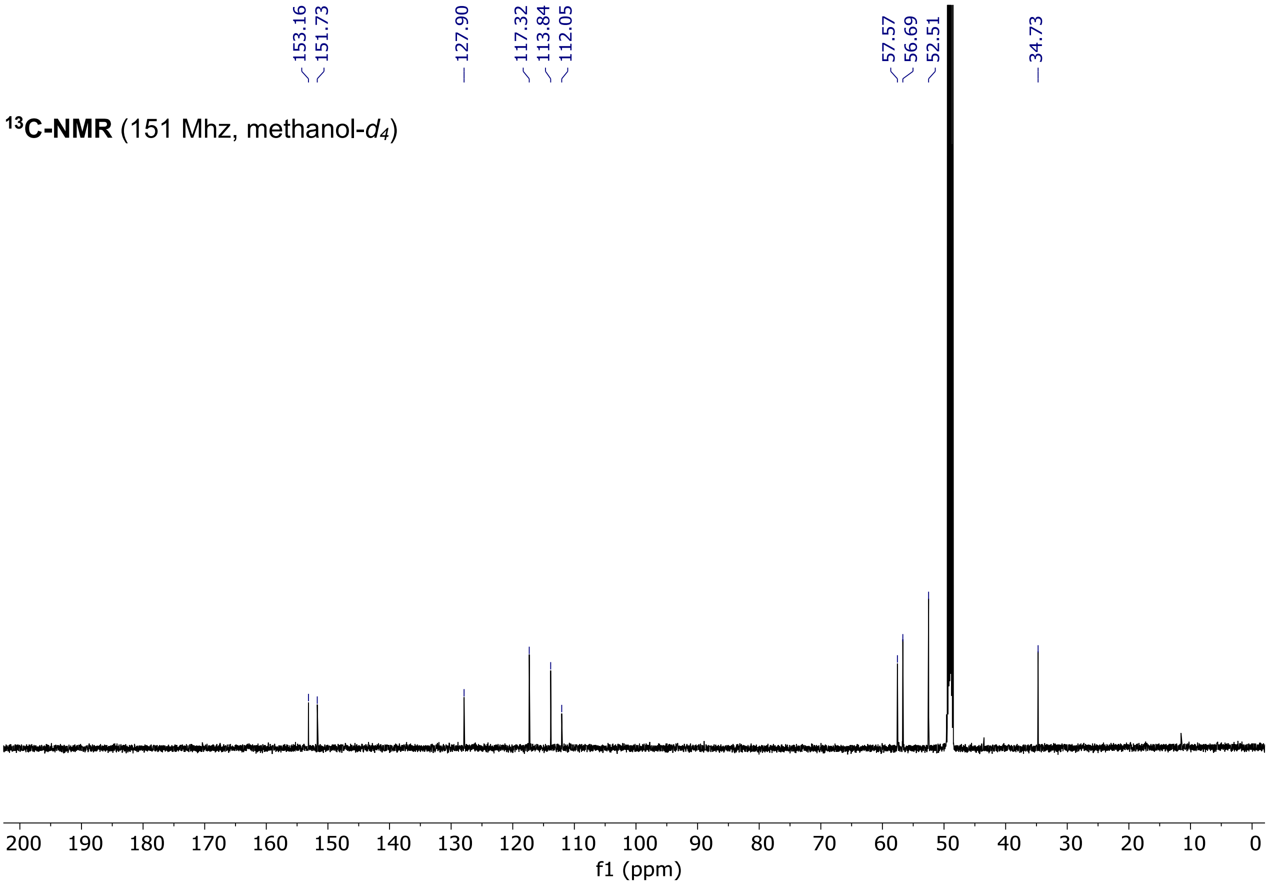

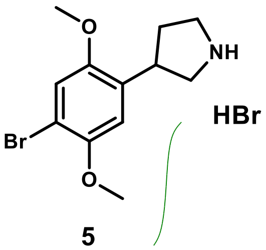

5

[1]**H-NMR** (600 Mhz, DMSO-d_6)

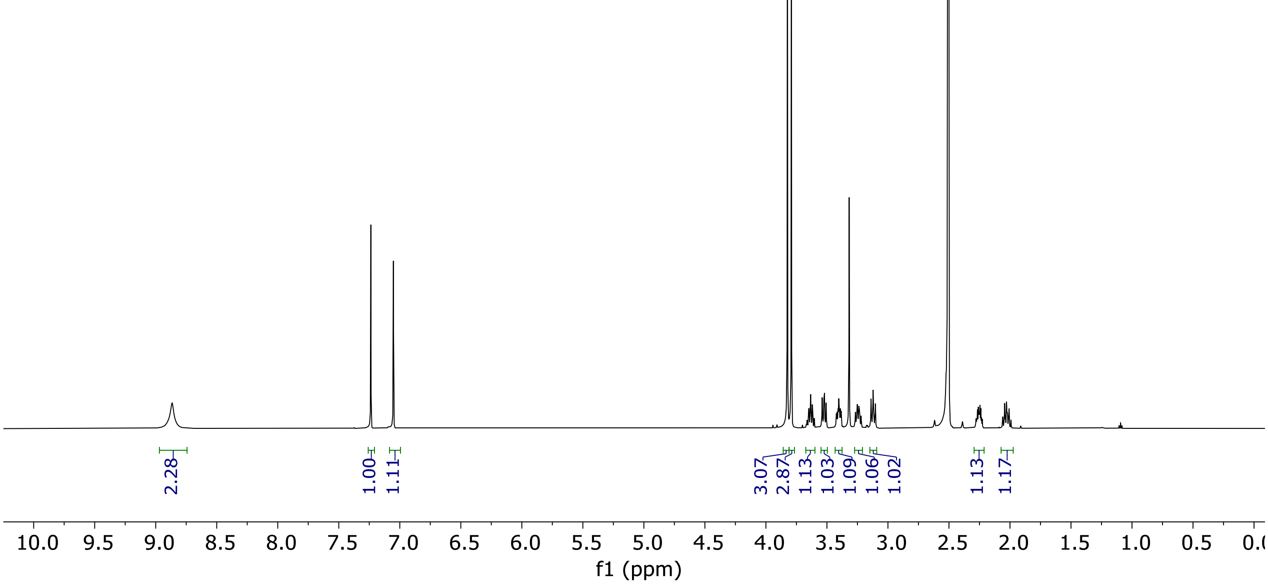

[13]**C-NMR** (151 Mhz, DMSO-d_6)

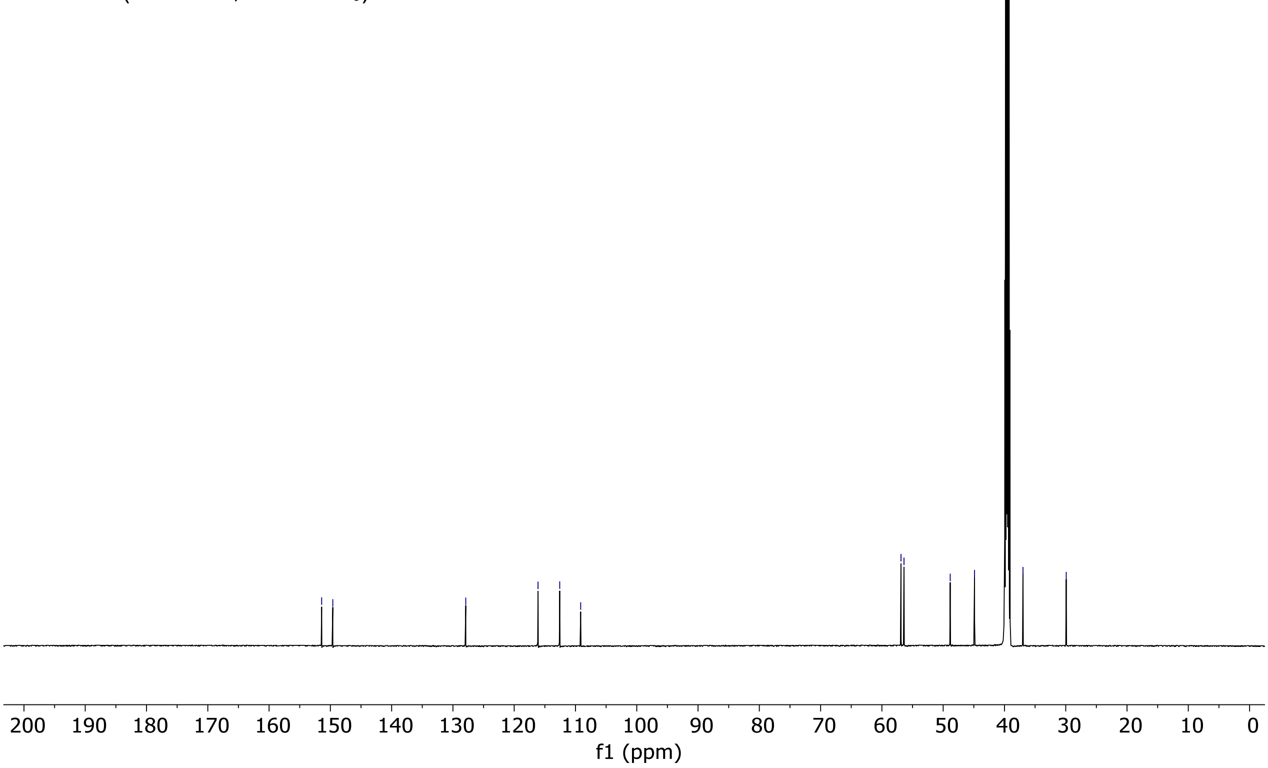

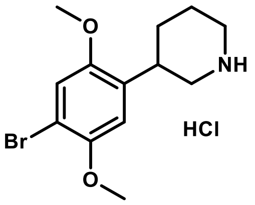

6

^{1}H-NMR (400 Mhz, methanol-d_4)

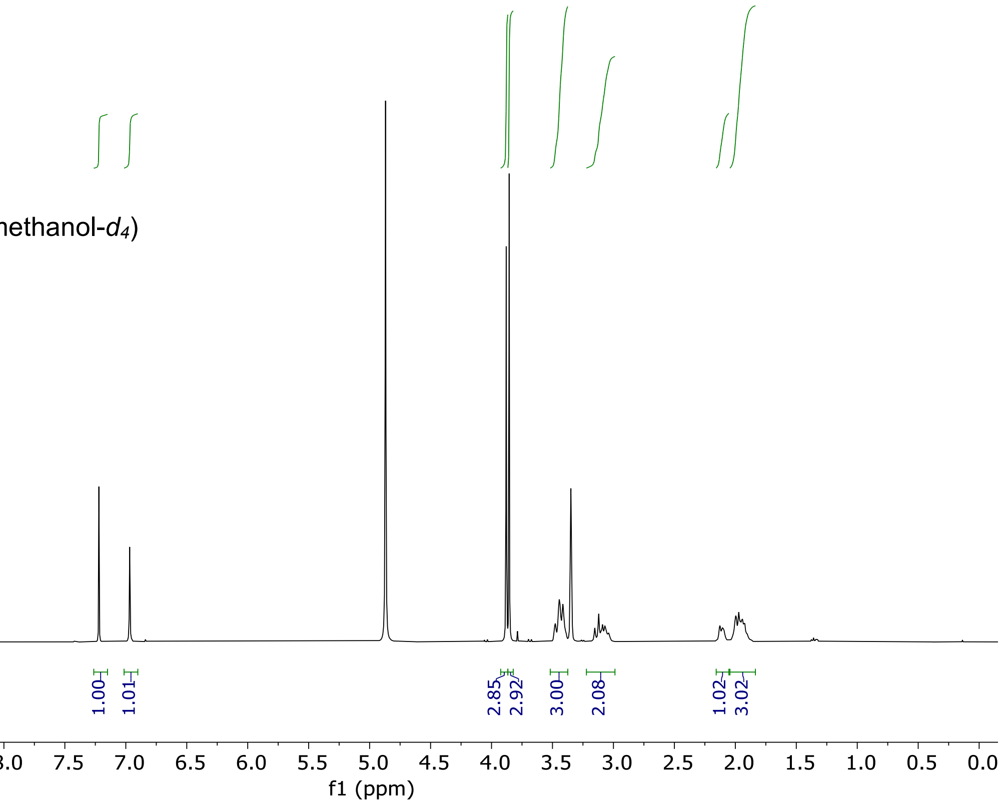

^{13}C-NMR (101 Mhz, methanol-d_4)

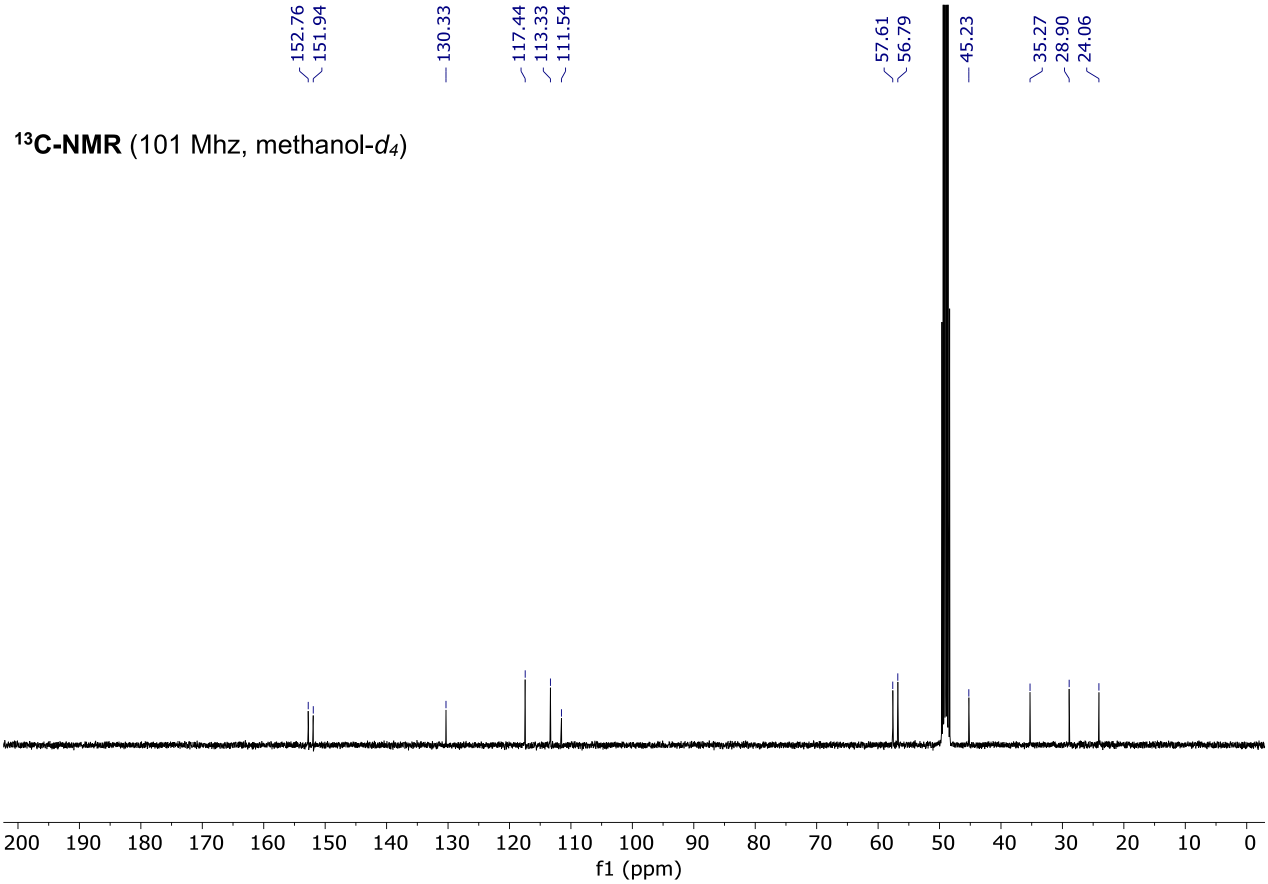

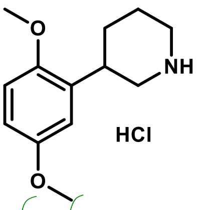

7

[1]**H-NMR** (400 Mhz, $CDCl_3$)

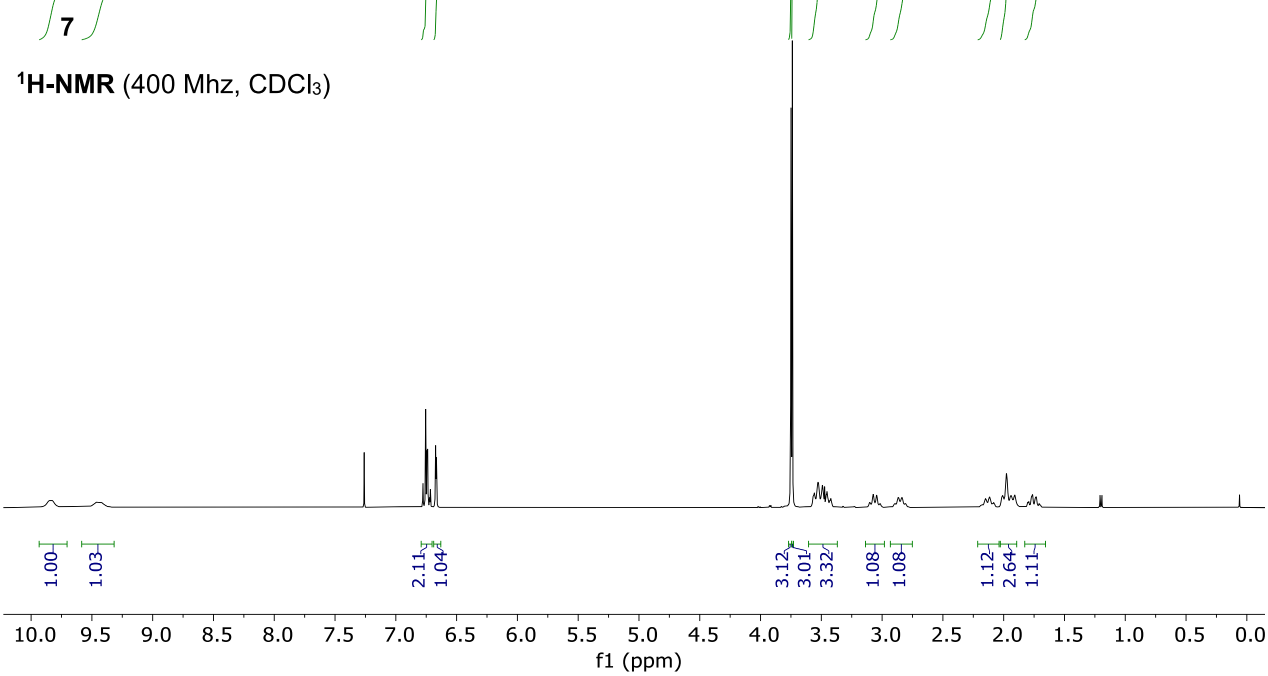

[13]**C-NMR** (101 Mhz, $CDCl_3$)

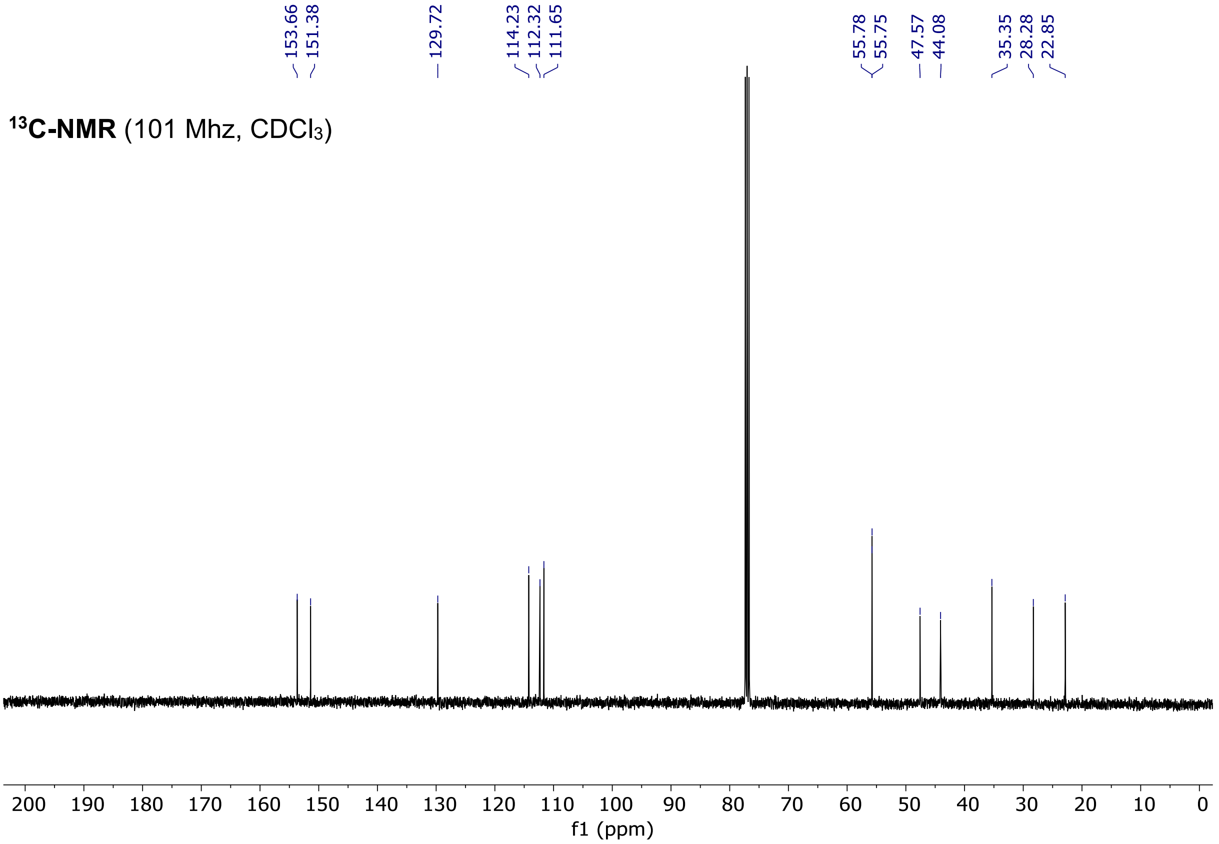

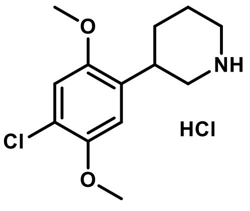

8

^{1}H-NMR (400 Mhz, $CDCl_3$)

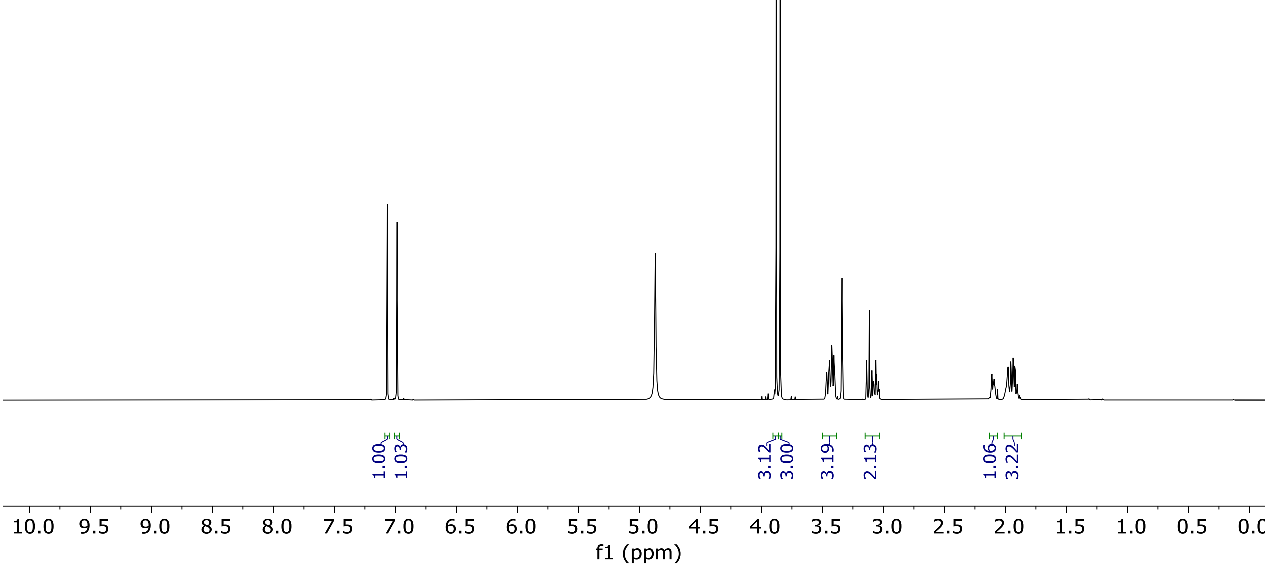

^{13}C-NMR (101 Mhz, $CDCl_3$)

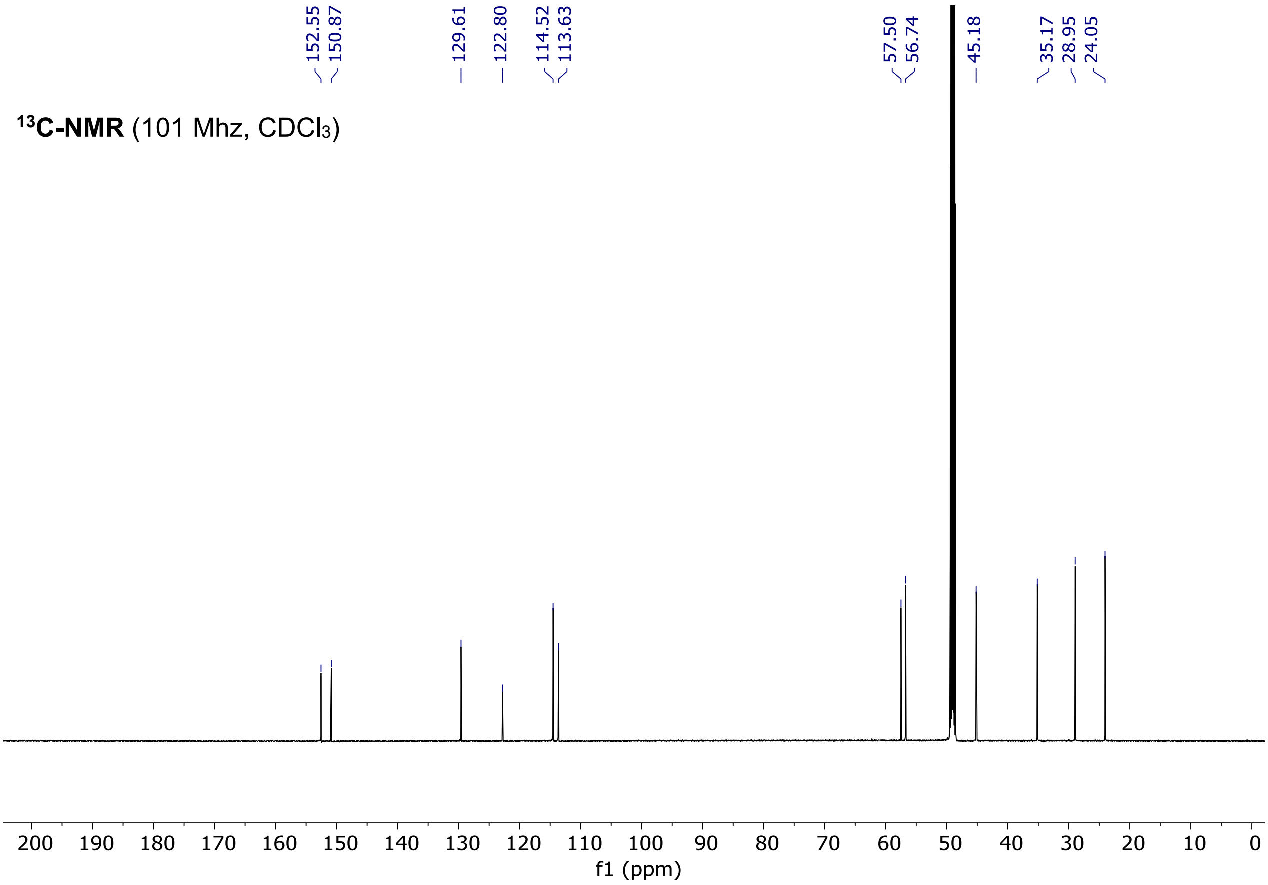

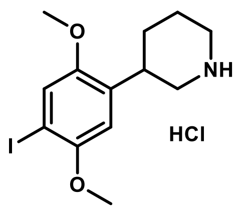

9

[1]**H-NMR** (400 Mhz, CDCl3)

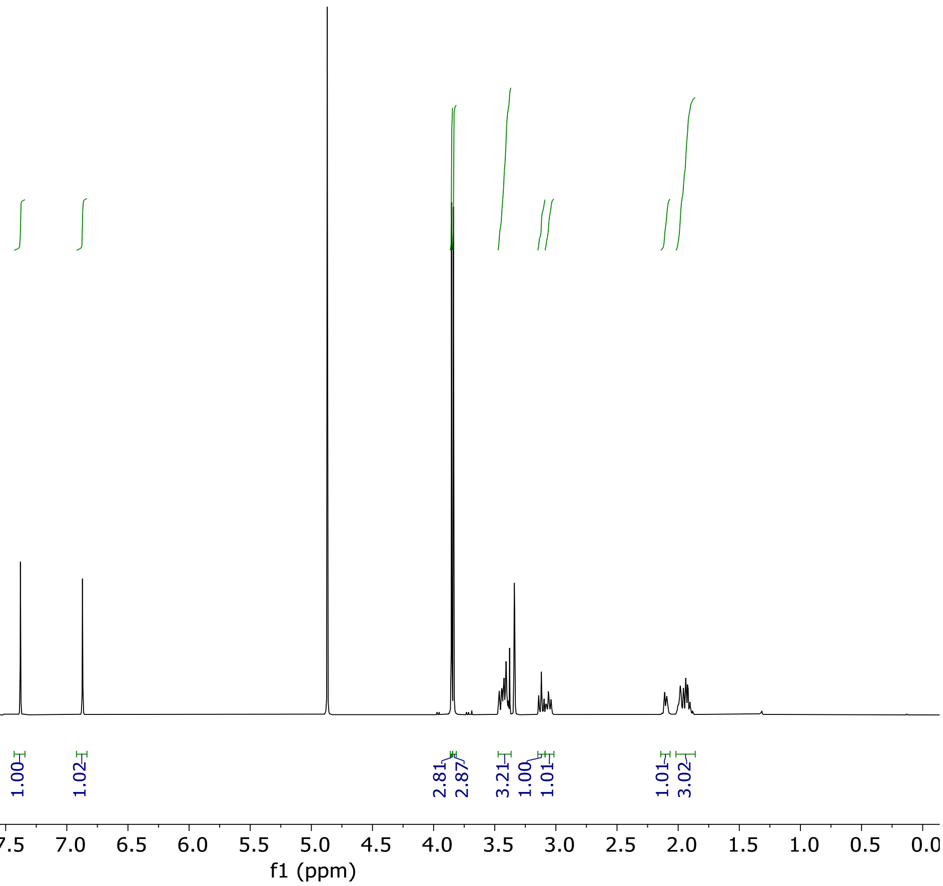

[13]**C-NMR** (101 Mhz, CDCl3)

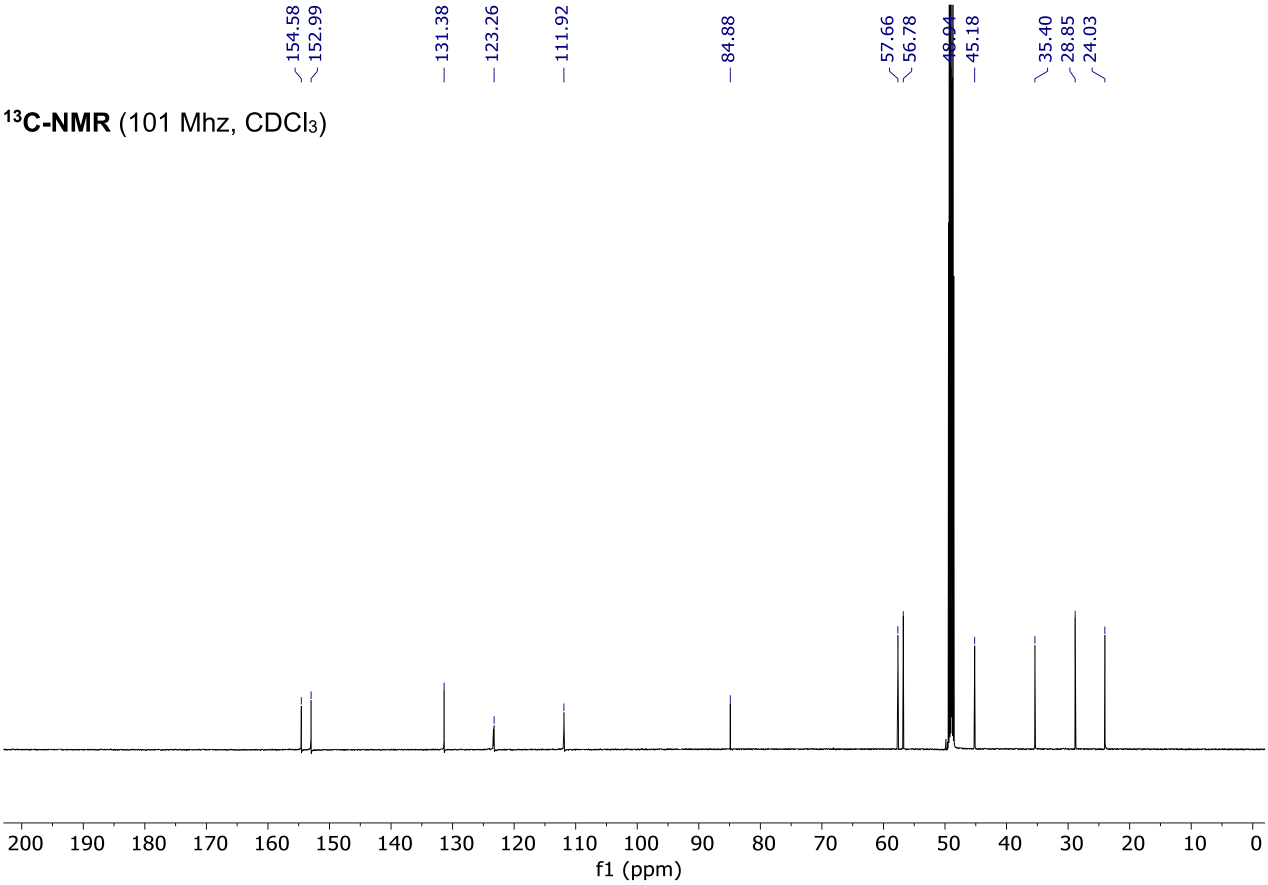

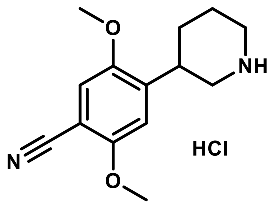

10

^{1}H-NMR (400 Mhz, $CDCl_3$)

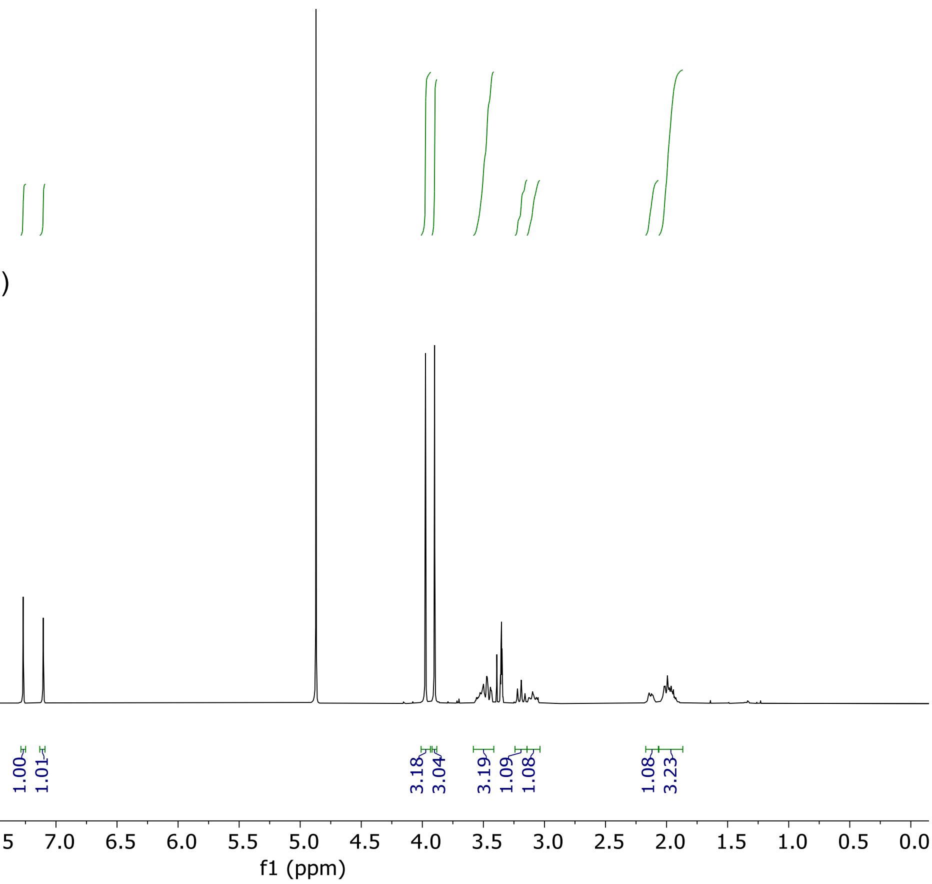

^{13}C-NMR (101 Mhz, $CDCl_3$)

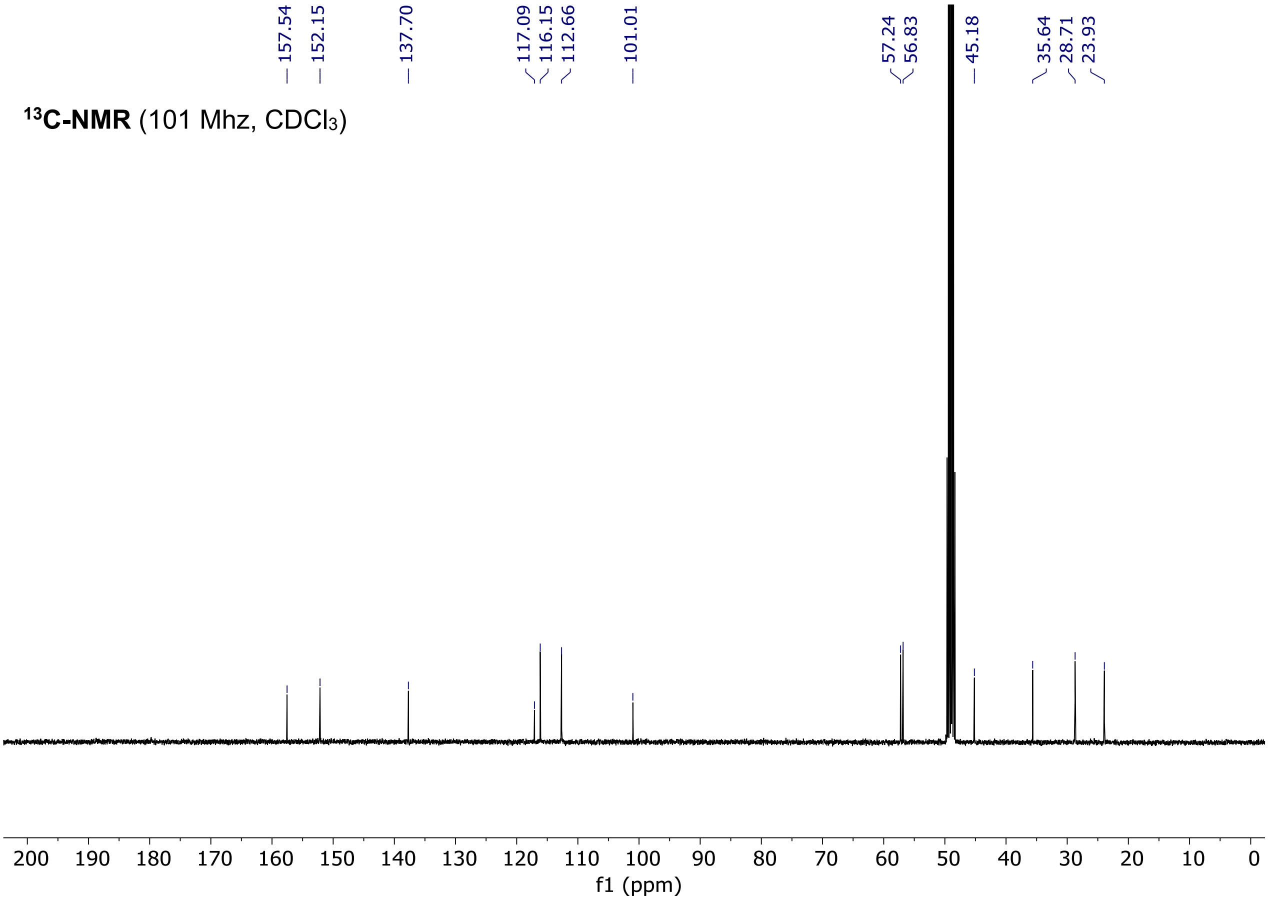

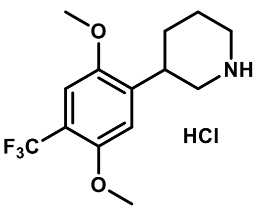

11

^{1}H-NMR (400 Mhz, $CDCl_3$)

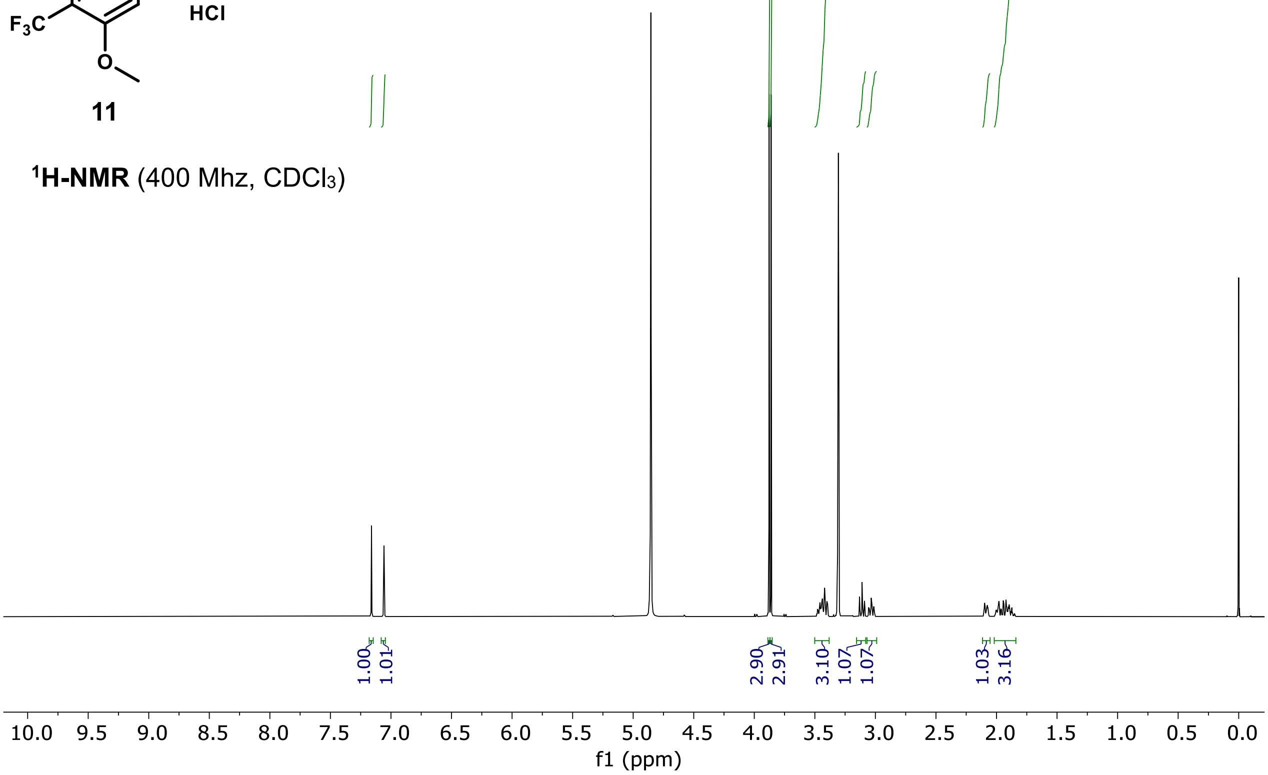

^{13}C-NMR (151 Mhz, $CDCl_3$)

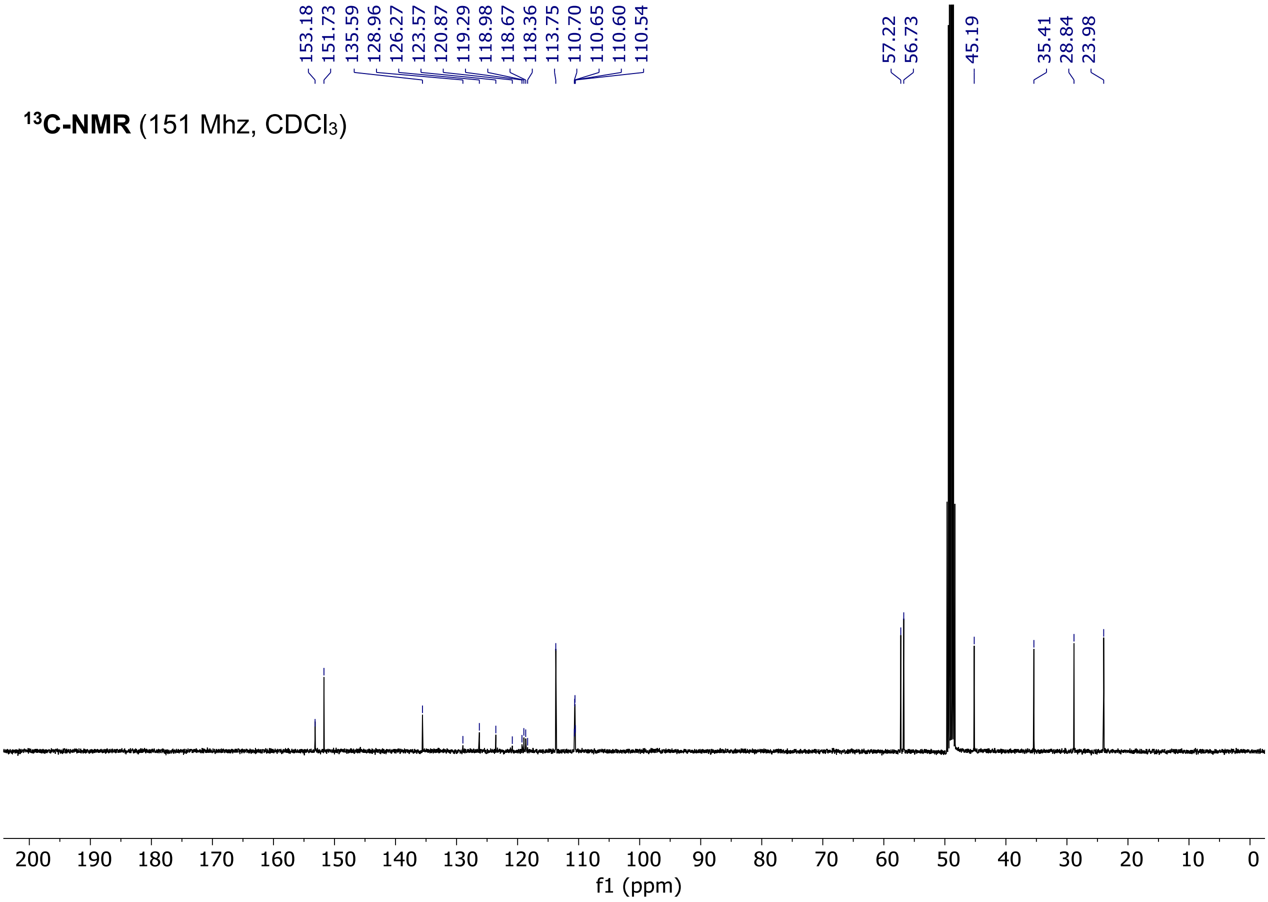

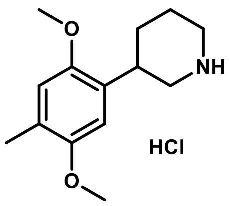

12

^{1}H-NMR (400 Mhz, methanol-*d*4)

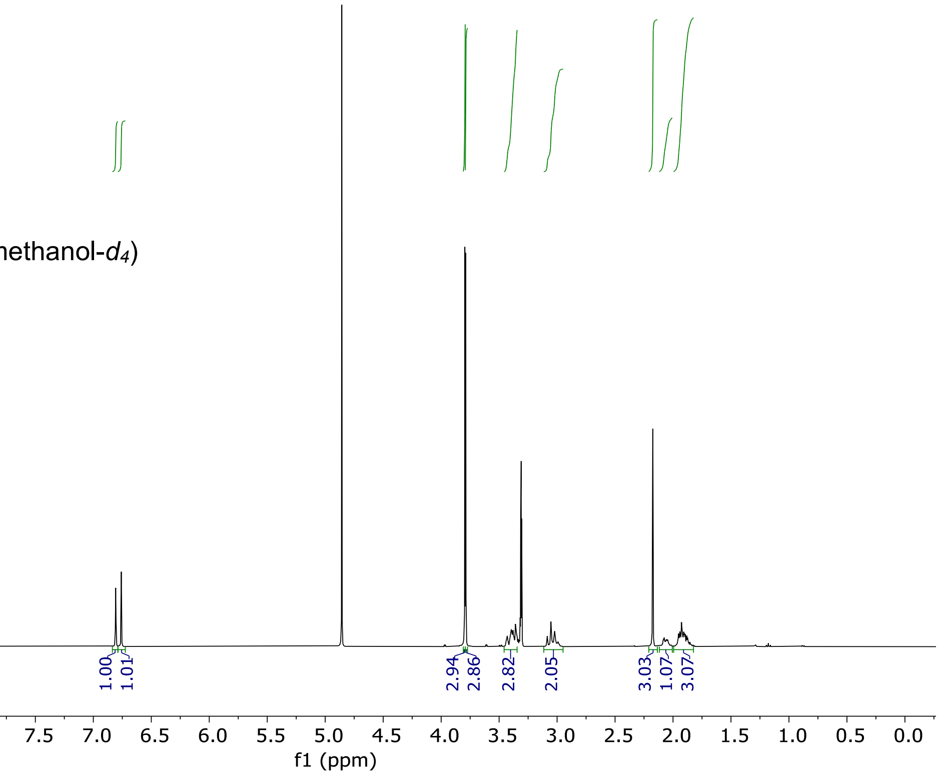

^{13}C-NMR (101 Mhz, methanol-*d*4)

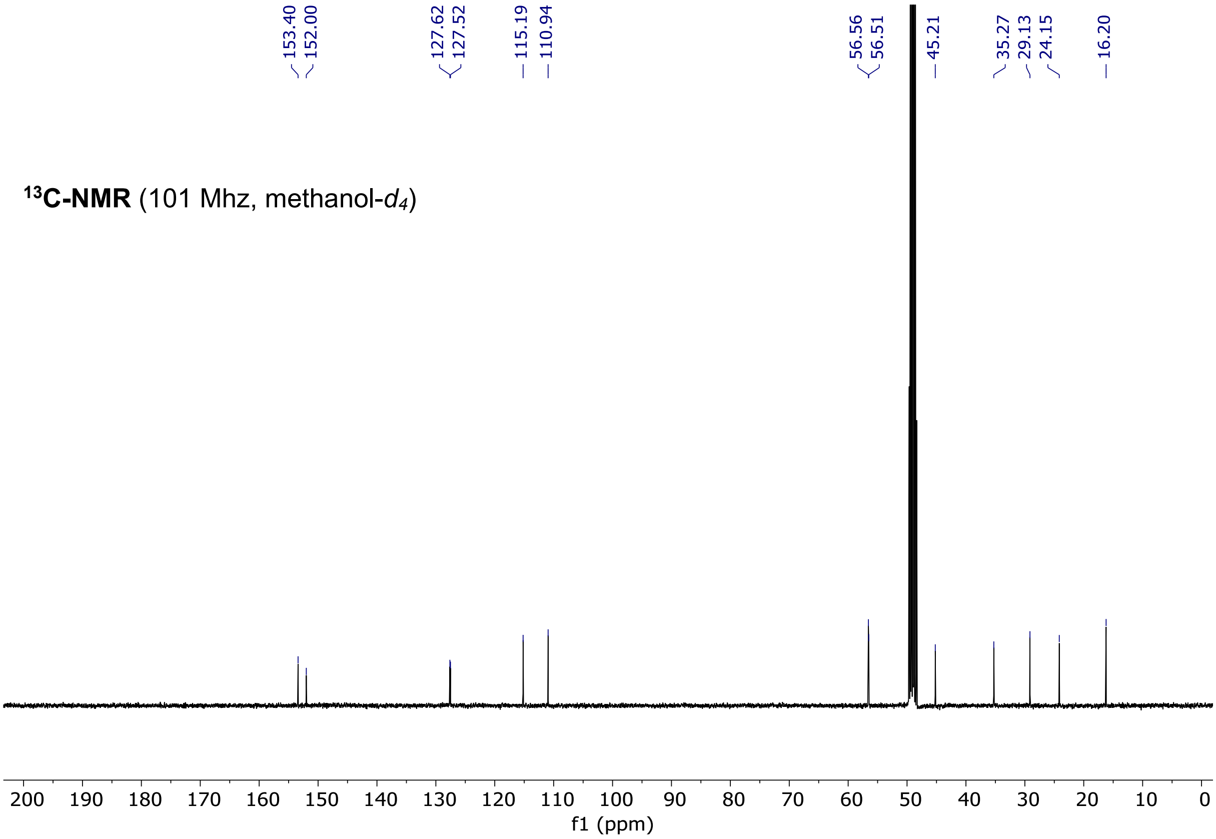

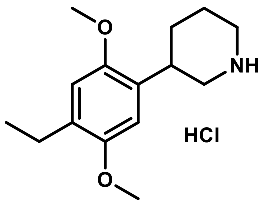

13

^{1}H-NMR (400 Mhz, methanol-d_4)

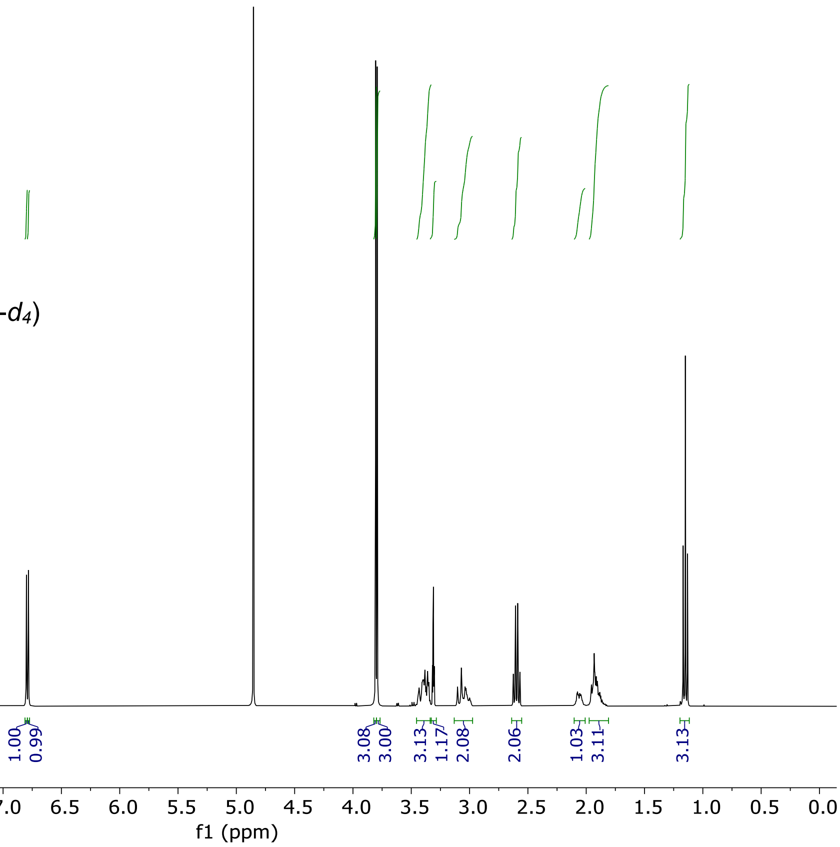

^{13}C-NMR (101 Mhz, methanol-d_4)

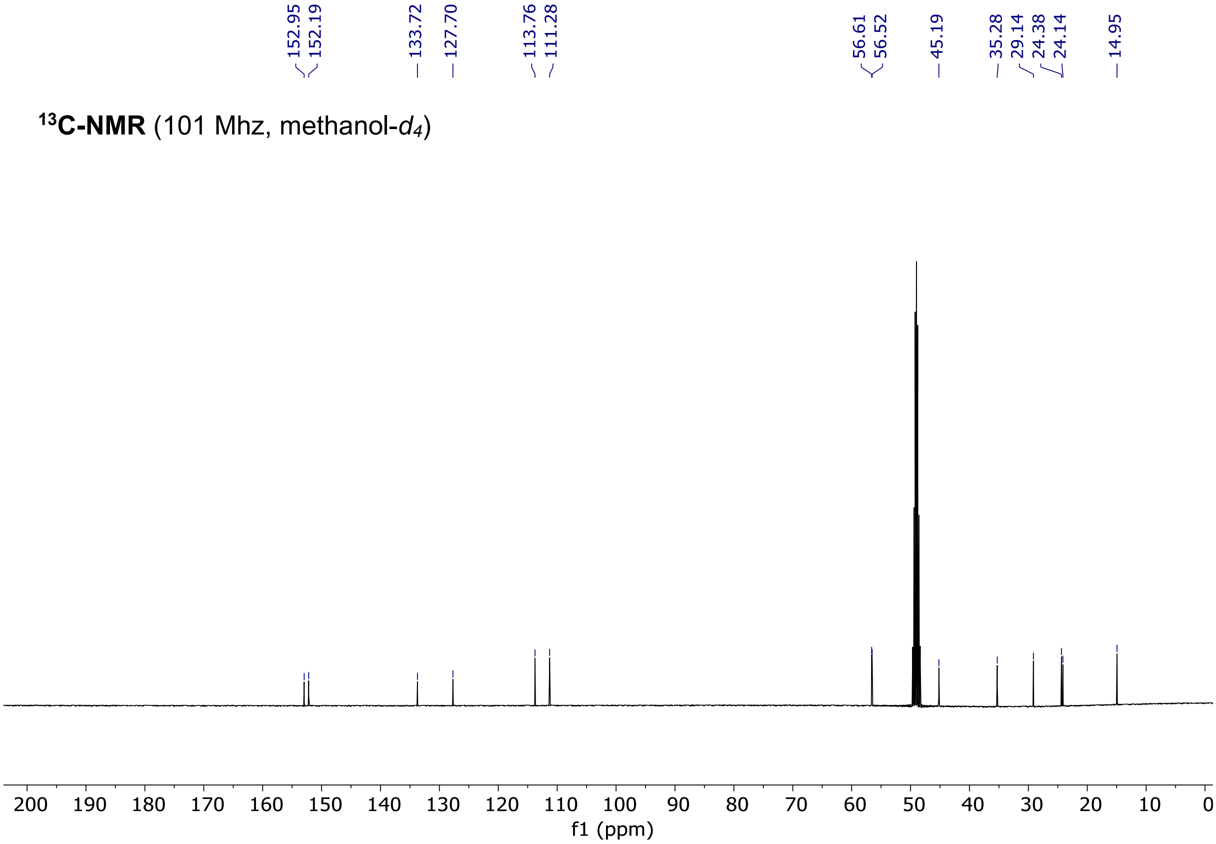

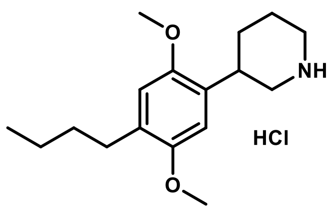

14

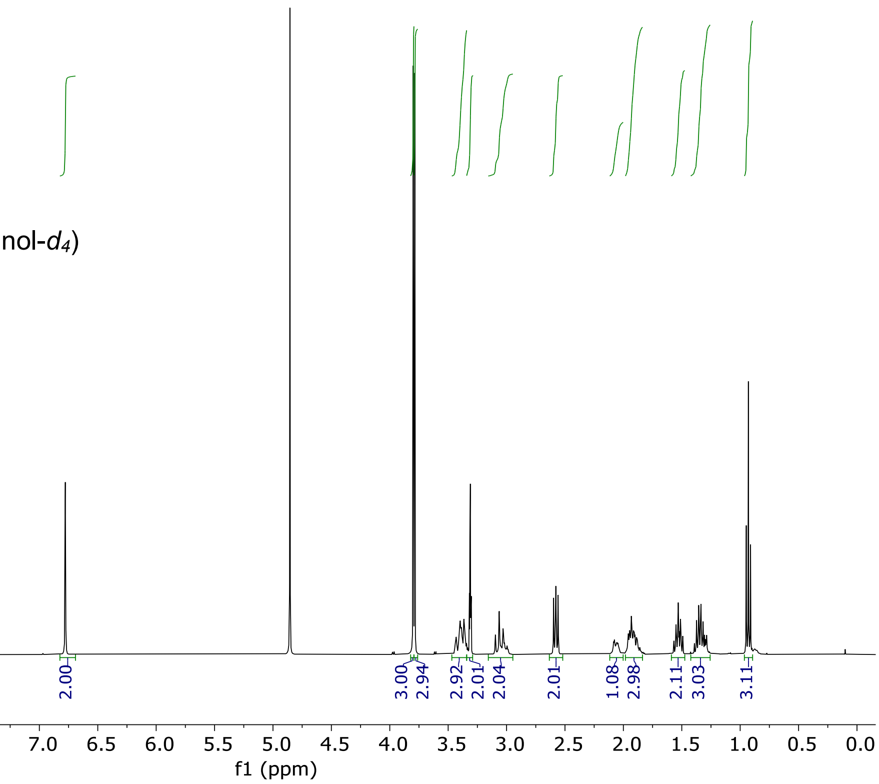

^{1}H-NMR (400 Mhz, methanol-d_4)

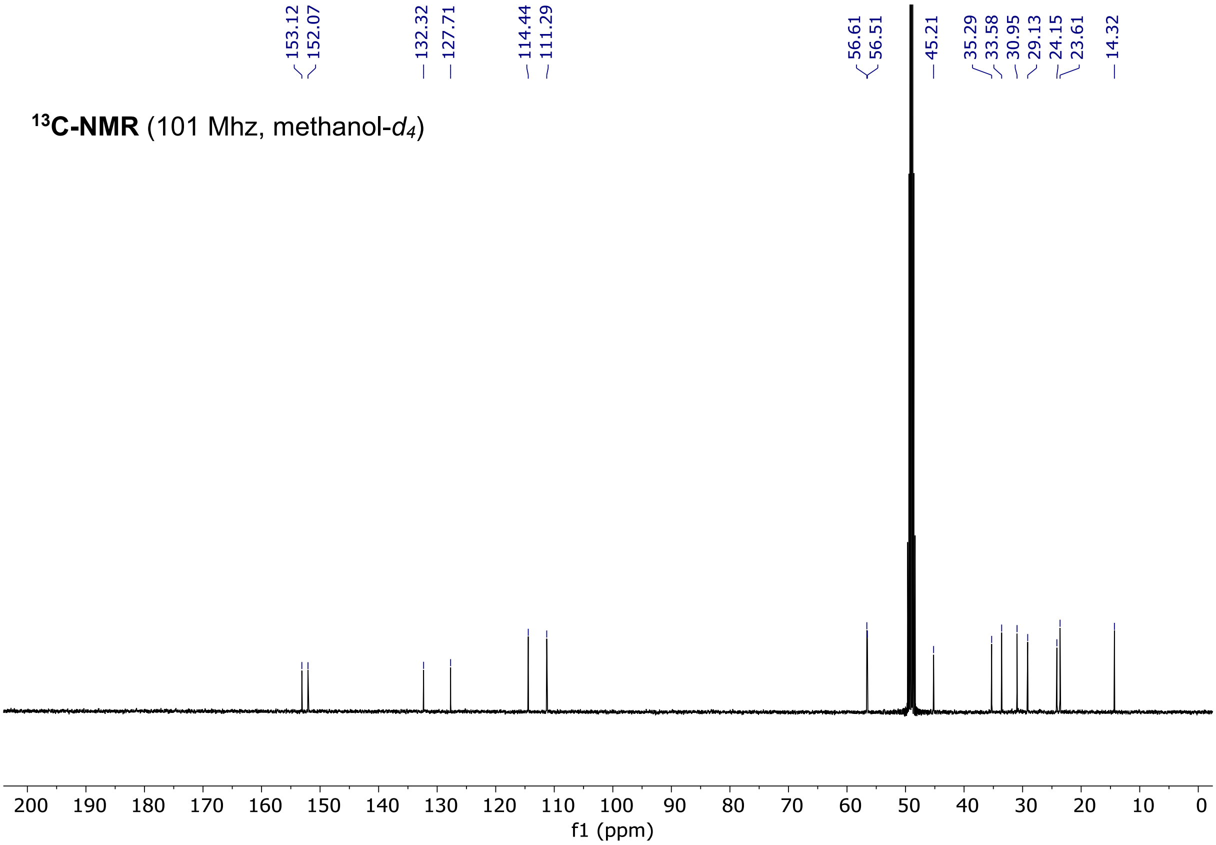

^{13}C-NMR (101 Mhz, methanol-d_4)

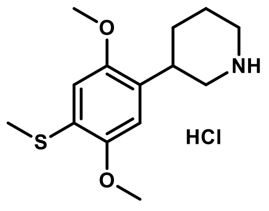

15

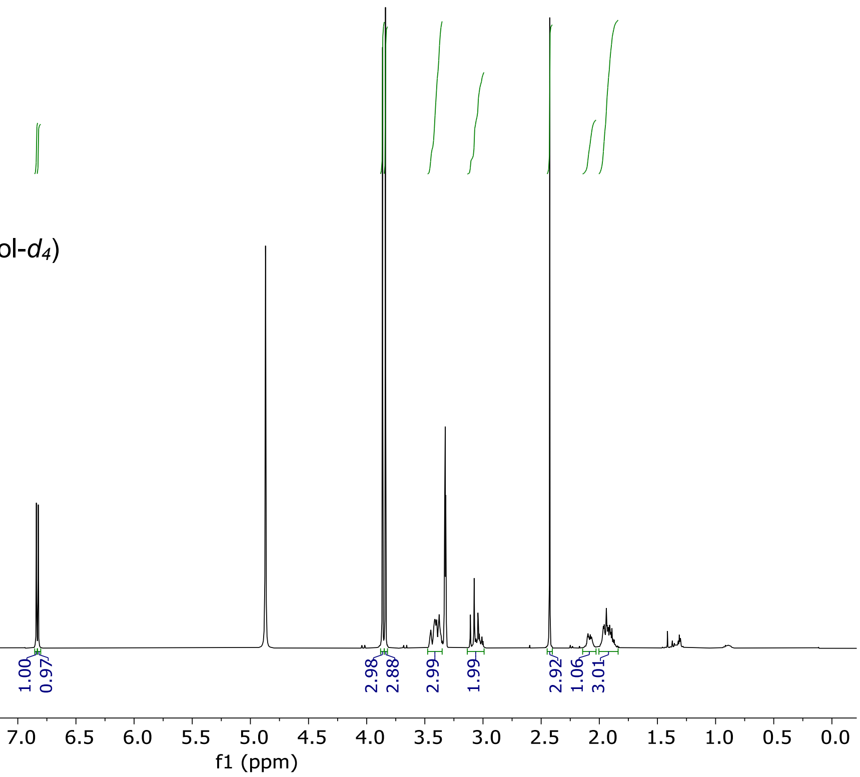

¹H-NMR (400 Mhz, methanol-*d₄*)

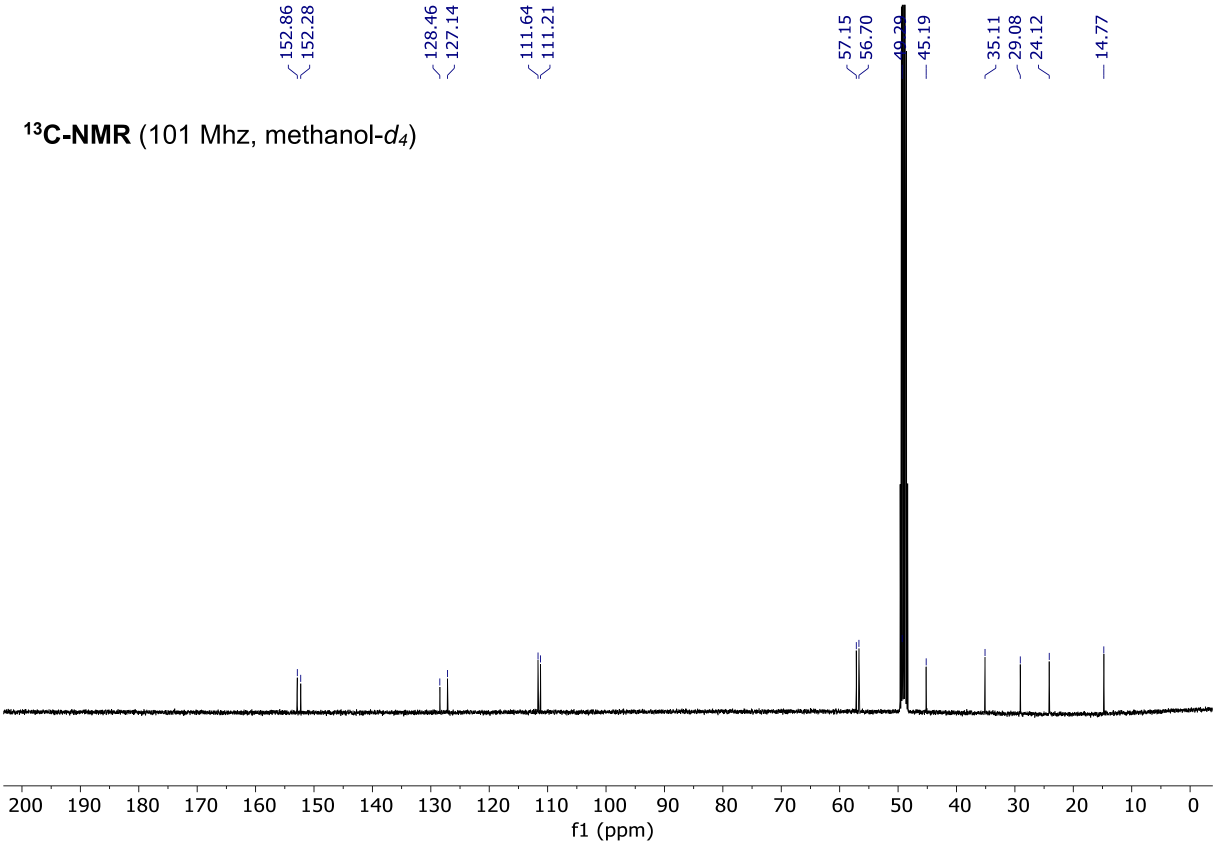

¹³C-NMR (101 Mhz, methanol-*d₄*)

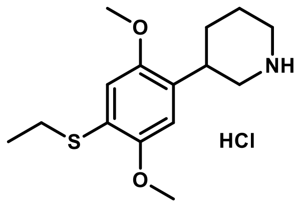

16

^{1}H-NMR (400 Mhz, methanol-d_4)

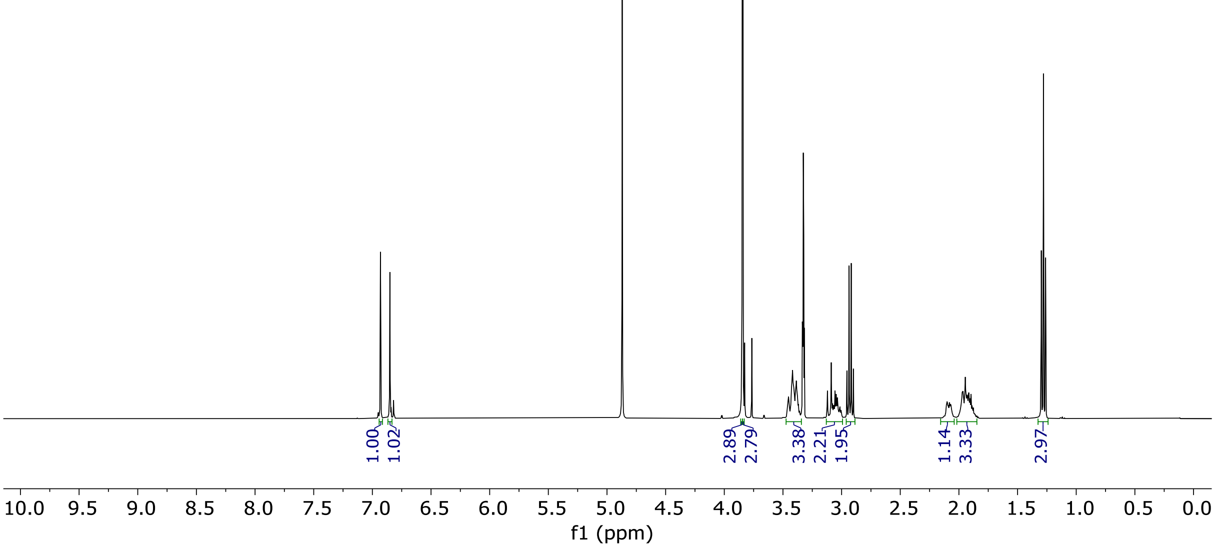

^{13}C-NMR (101 Mhz, methanol-d_4)

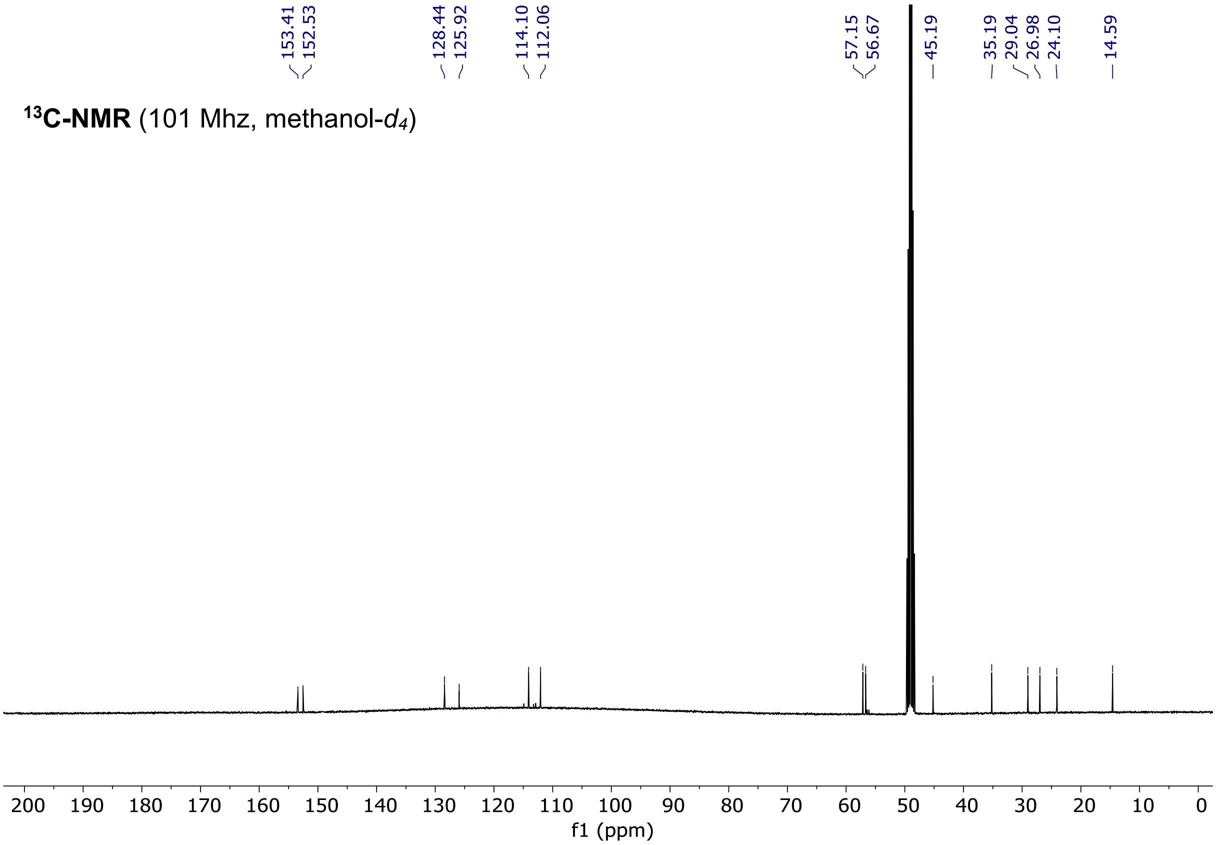

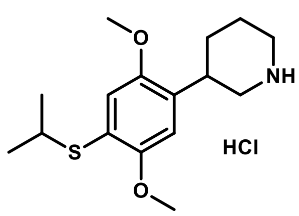

17

^{1}H-NMR (400 Mhz, methanol-d_4)

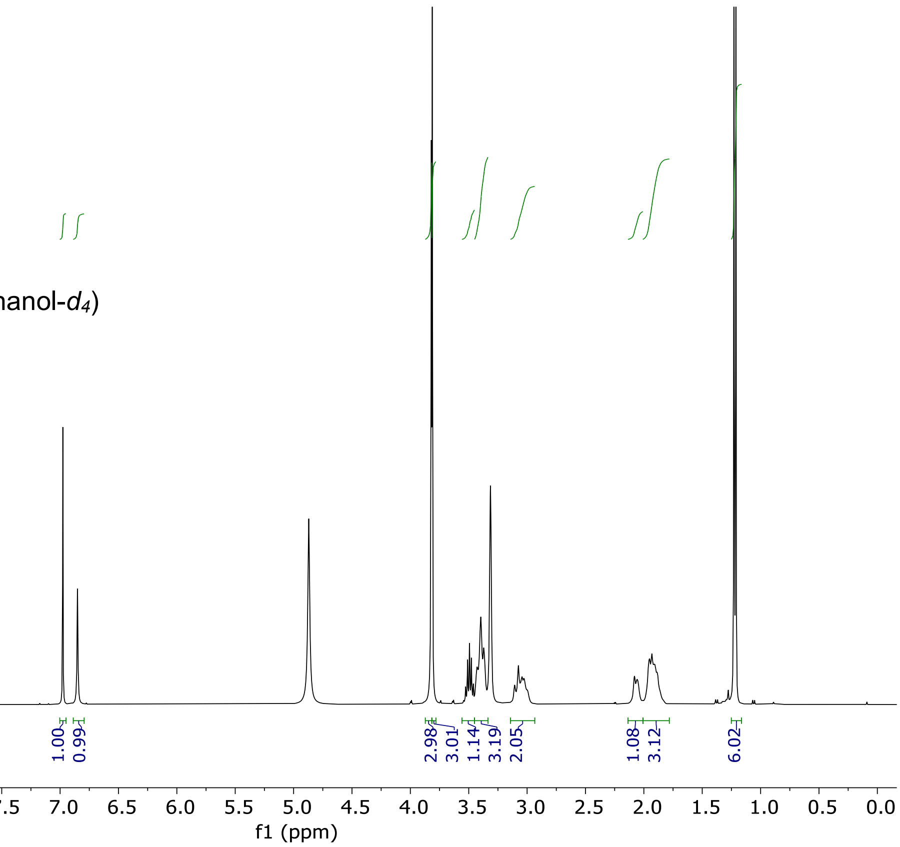

^{13}C-NMR (101 Mhz, methanol-d_4)

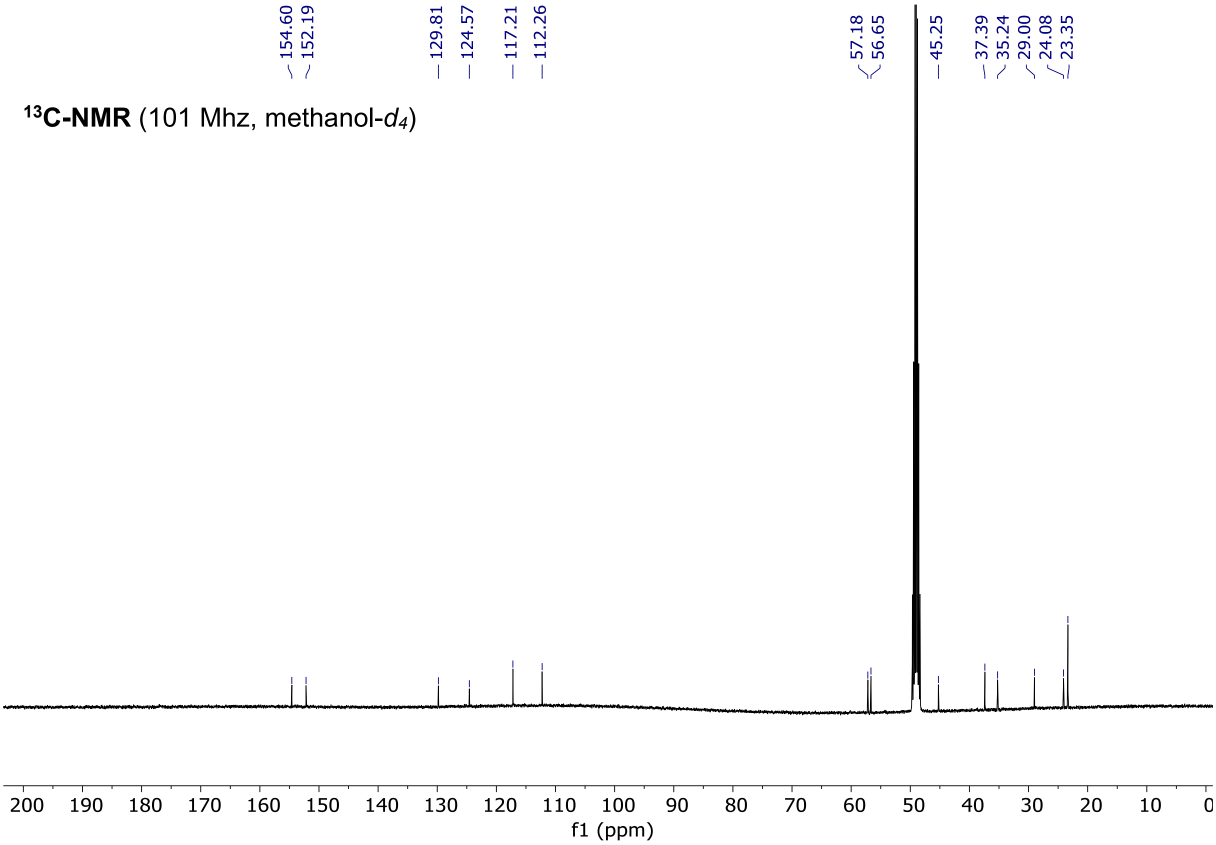

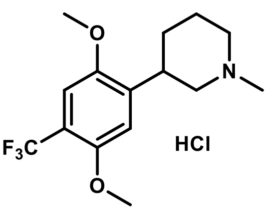

19

^{1}H-NMR (400 Mhz, methanol-*d4*)

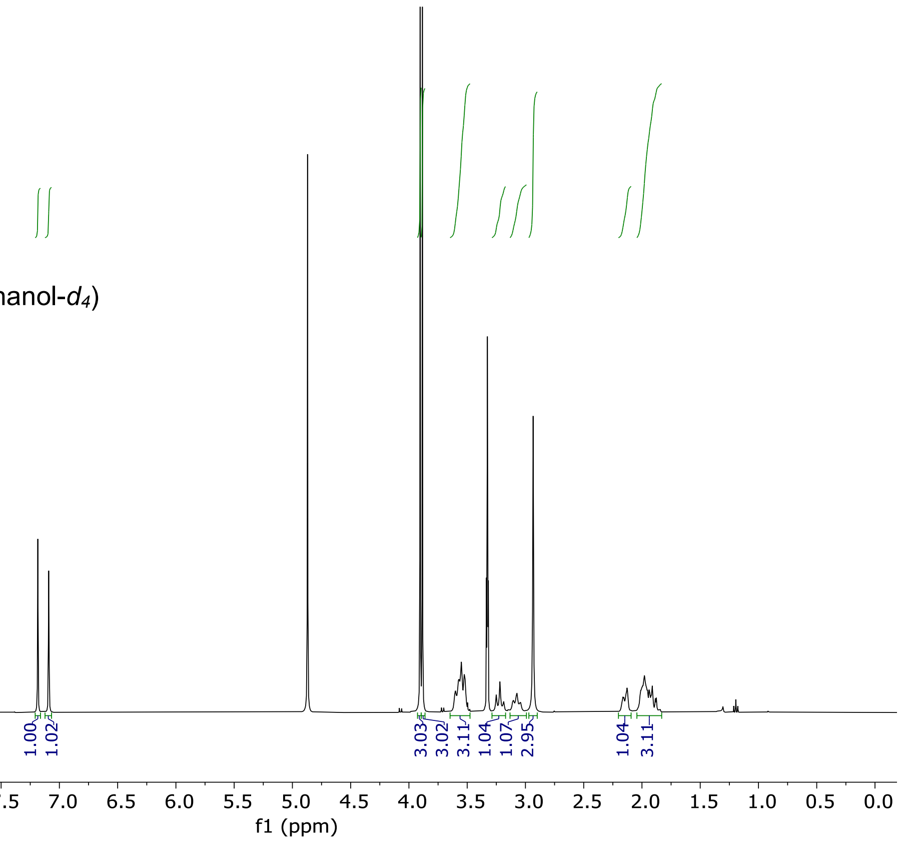

^{13}C-NMR (101 Mhz, methanol-*d4*)

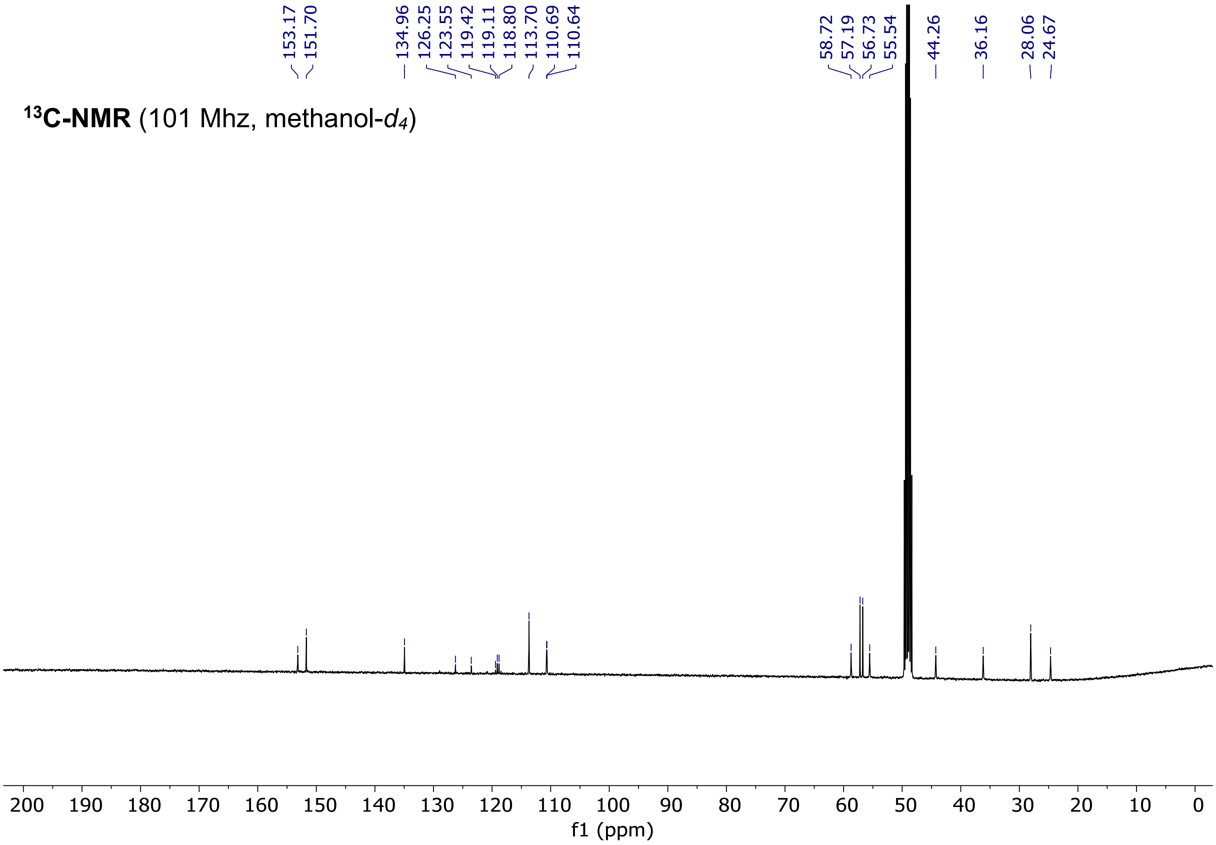

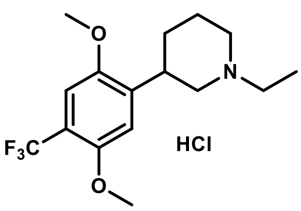

20

^{1}H-NMR (400 Mhz, methanol-d_4)

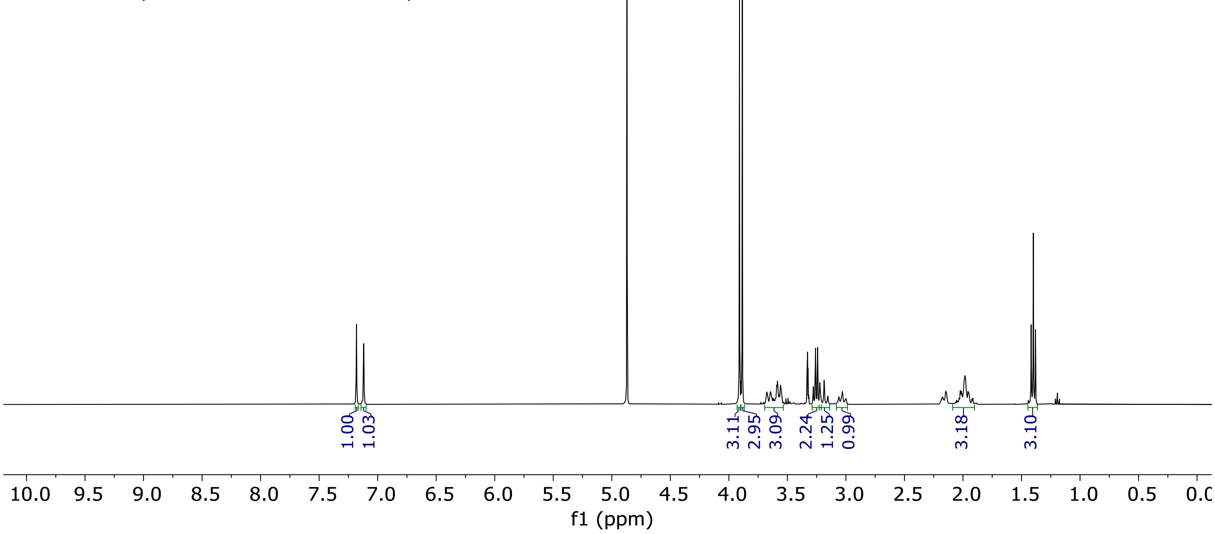

^{13}C-NMR (101 Mhz, methanol-d_4)

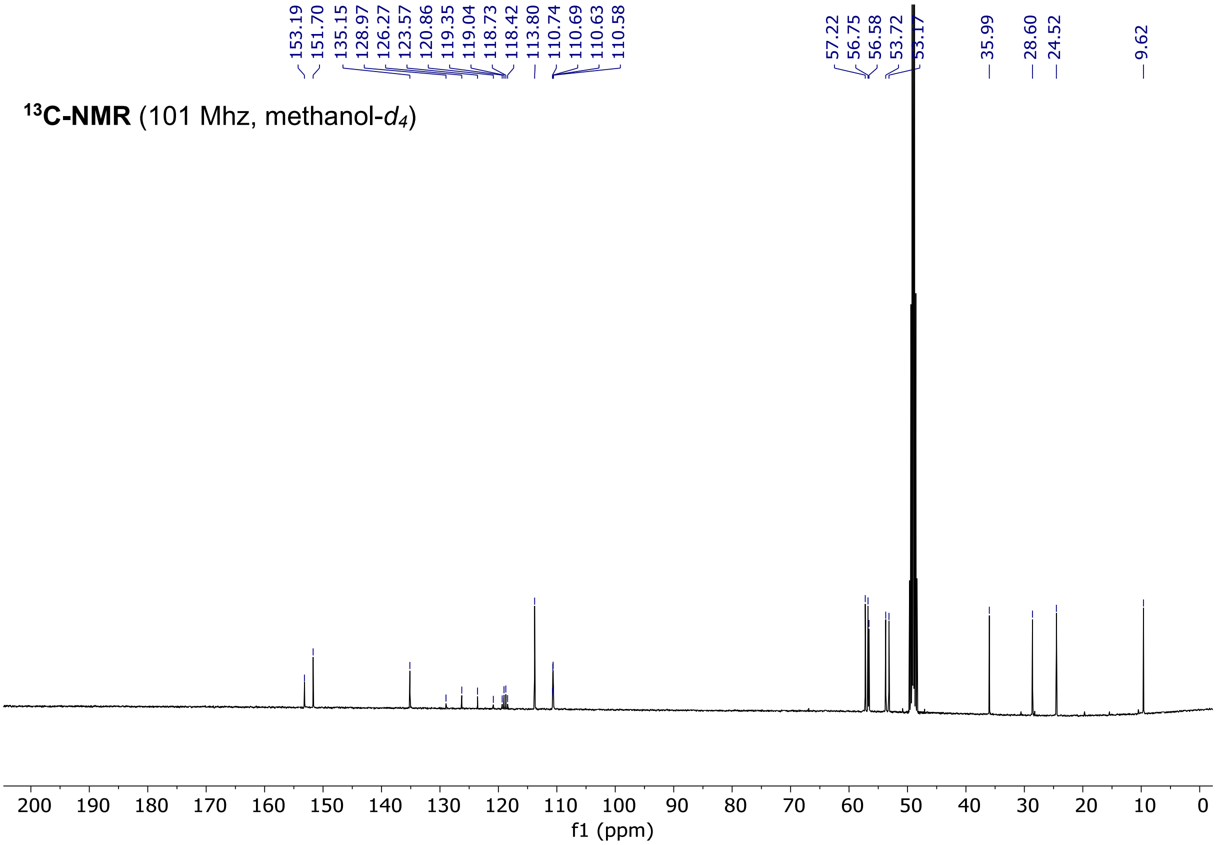

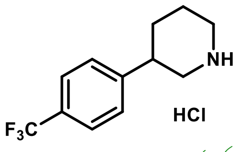

21

^{1}H-NMR (400 Mhz, CDCl3)

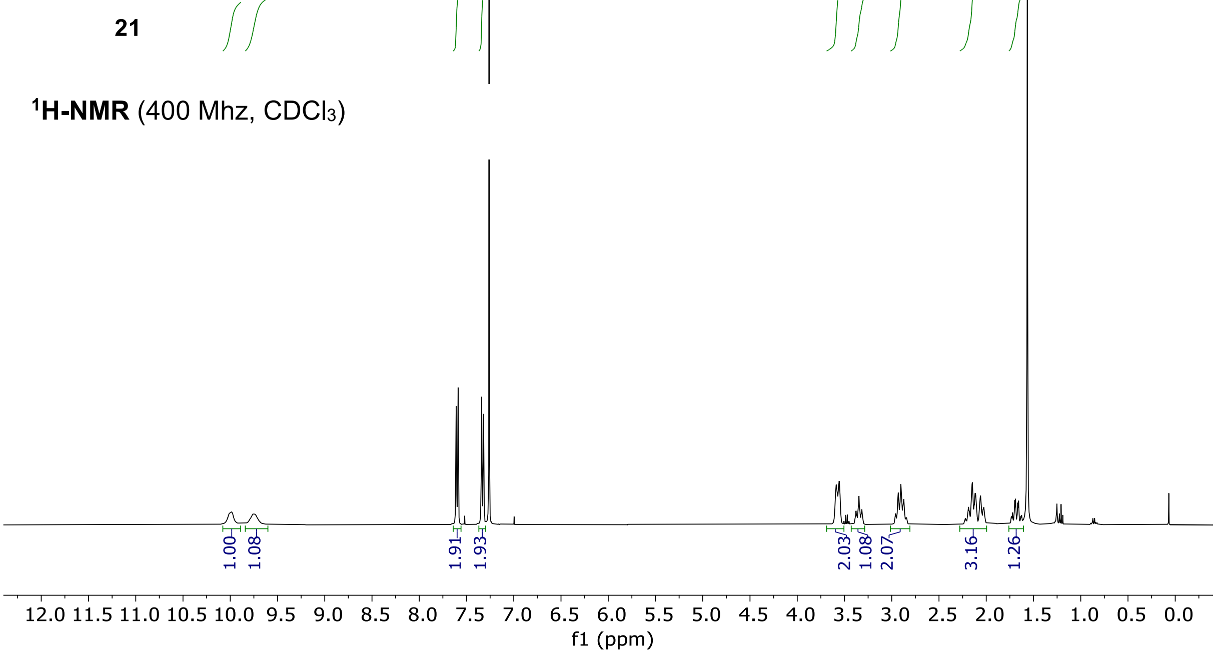

^{13}C-NMR (100 Mhz, CDCl3)

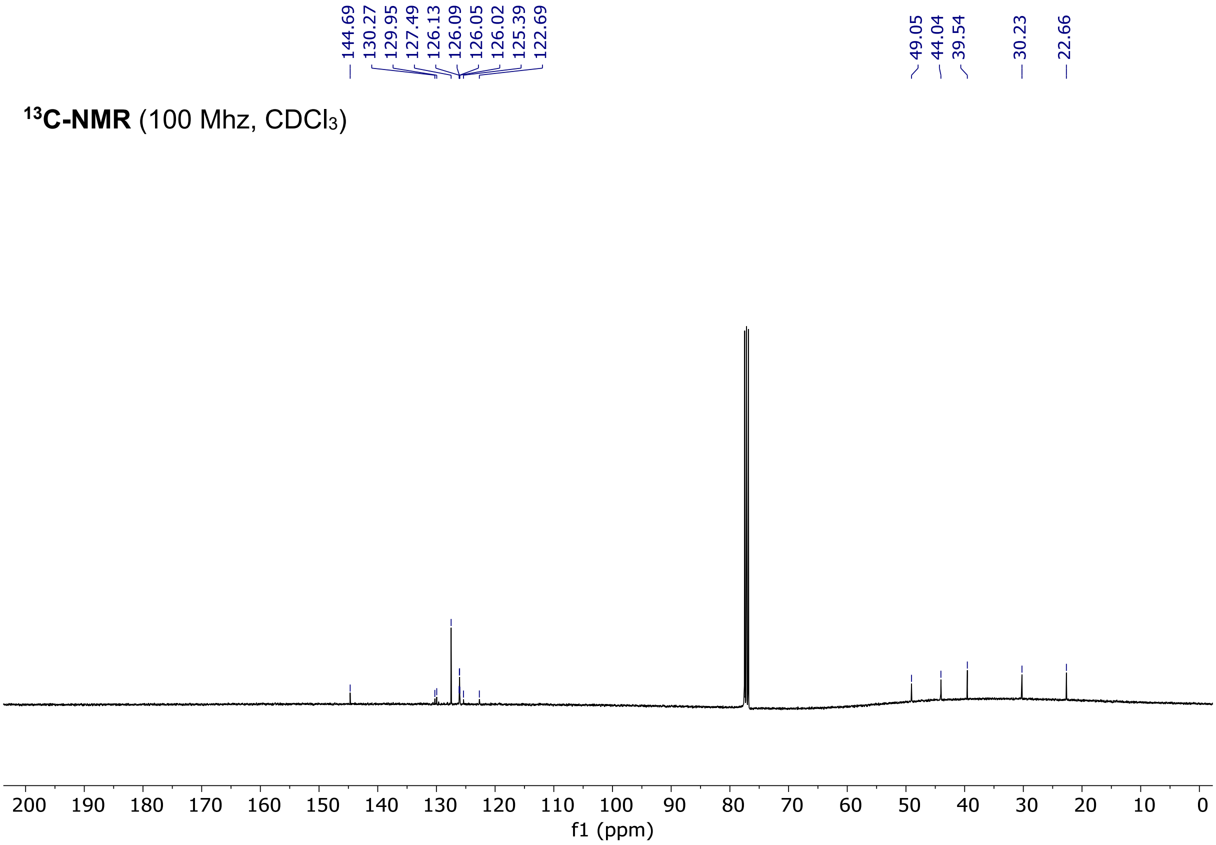

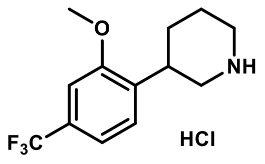

22

^{1}H-NMR (400 Mhz, methanol-*d*$_4$)

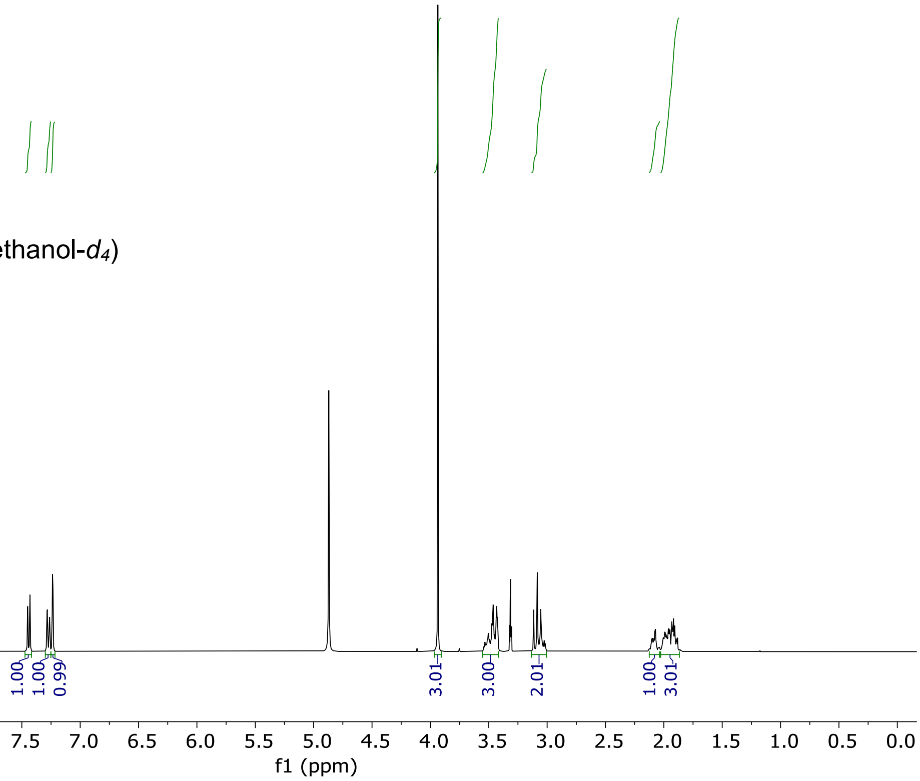

^{13}C-NMR (101 Mhz, methanol-*d*$_4$)

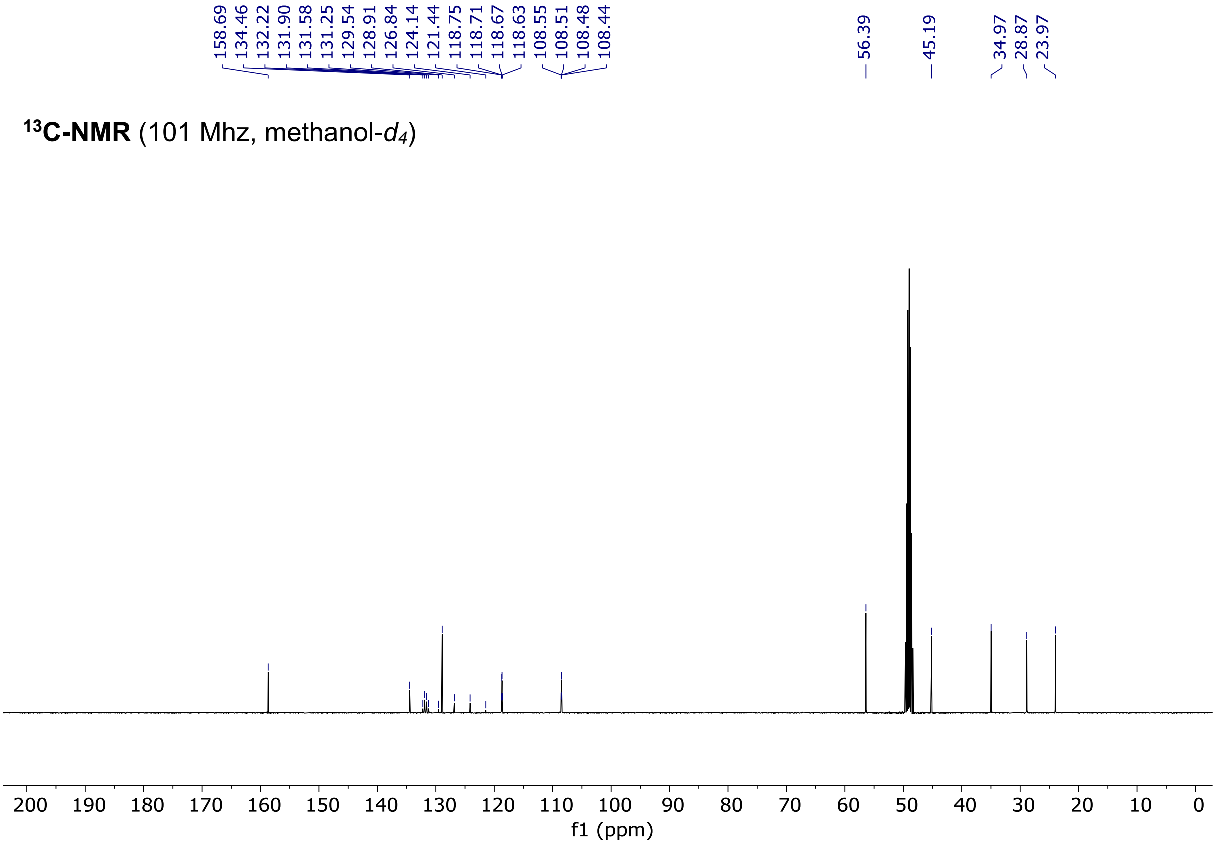

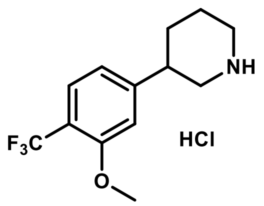

23

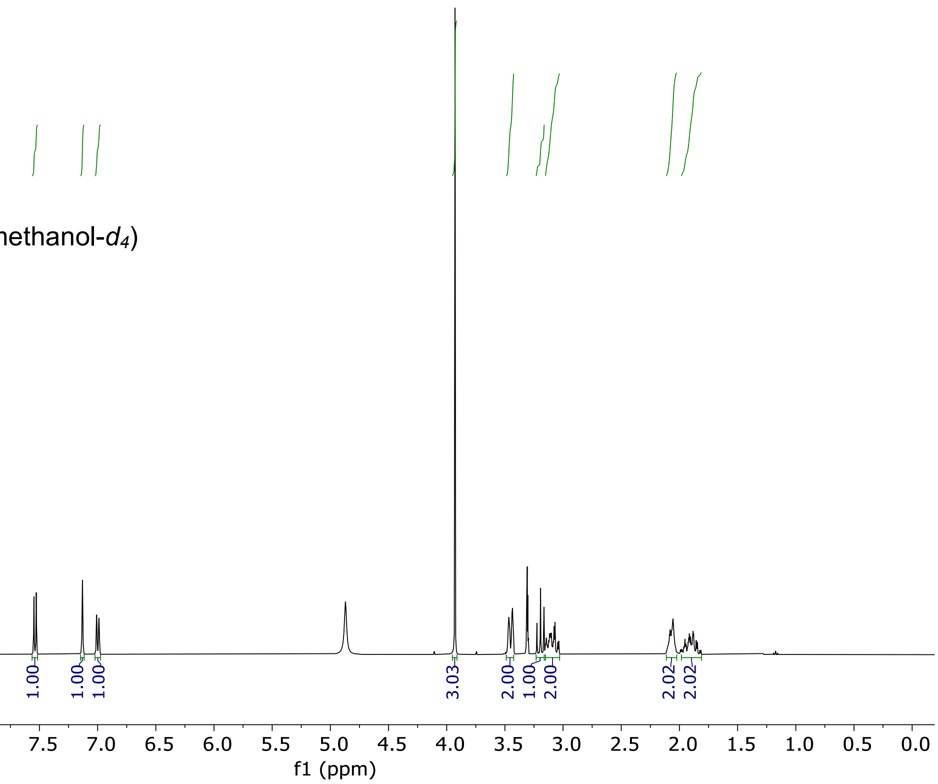

^{1}H-NMR (400 Mhz, methanol-d_4)

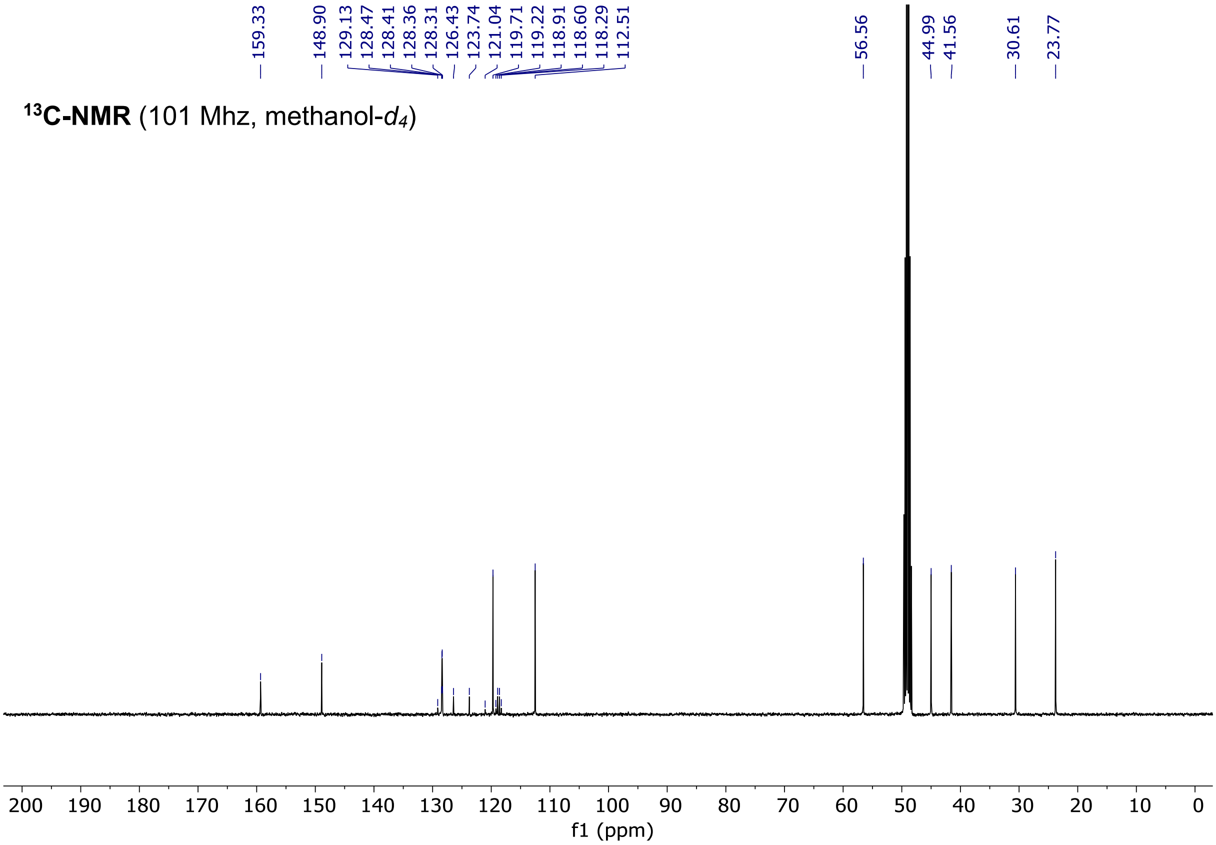

^{13}C-NMR (101 Mhz, methanol-d_4)

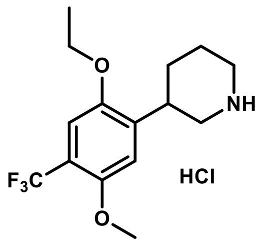

24

^{1}H-NMR (400 Mhz, methanol-*d*₄)

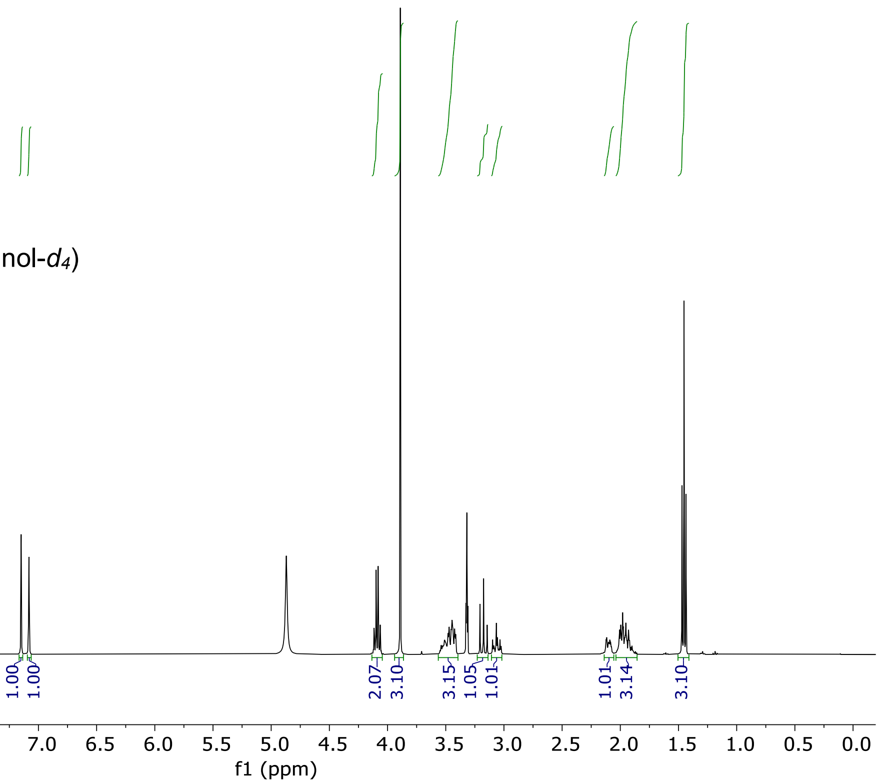

^{13}C-NMR (101 Mhz, methanol-*d*₄)

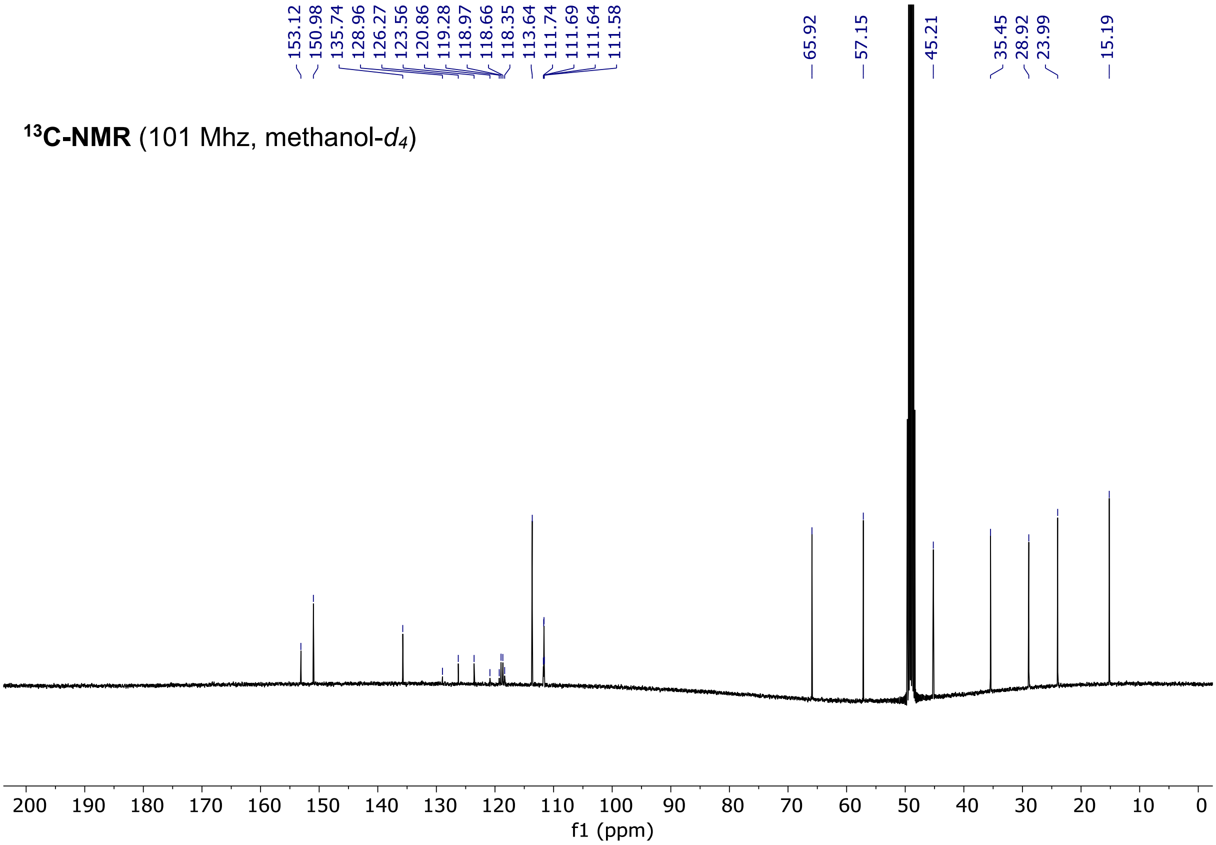

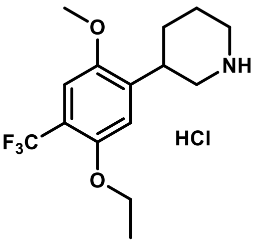

25

^{1}H-NMR (400 Mhz, methanol-*d₄*)

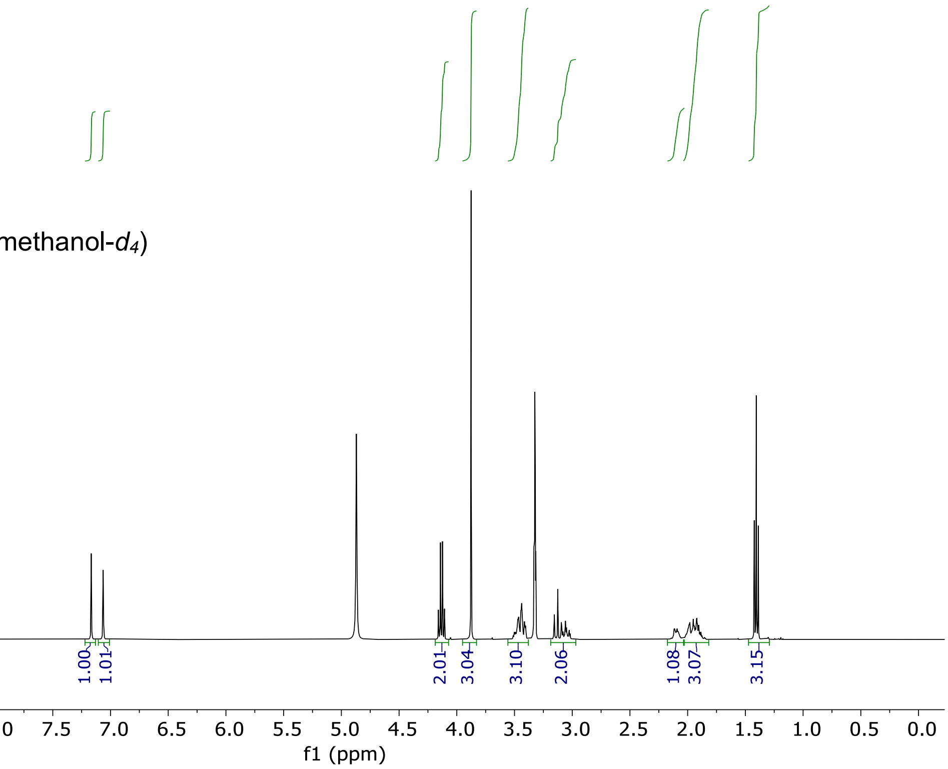

^{13}C-NMR (101 Mhz, methanol-*d₄*)

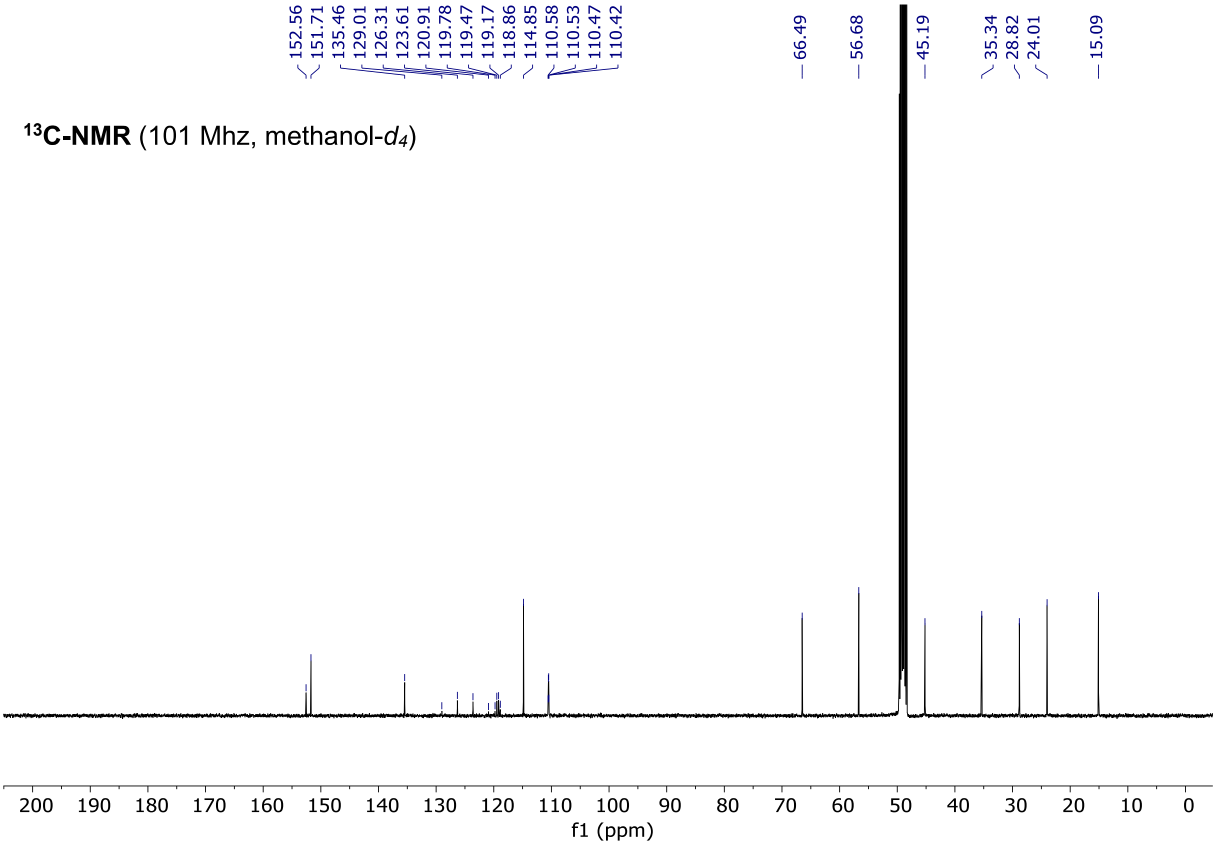

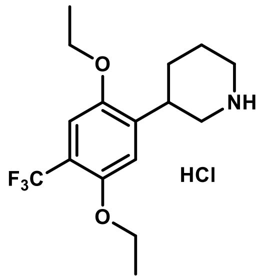

^{1}H-NMR (400 Mhz, methanol-*d₄*)

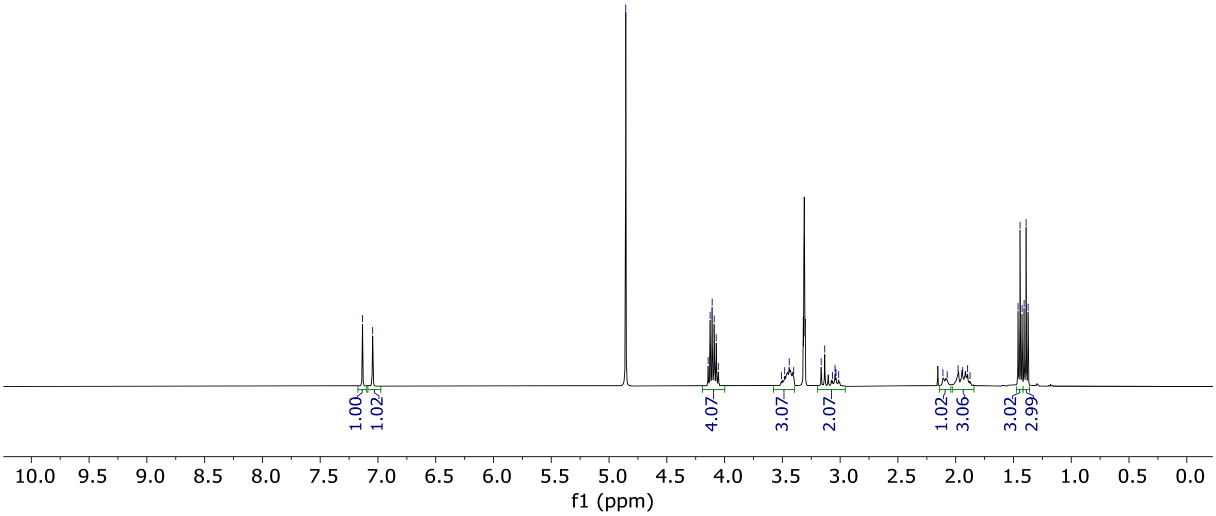

^{13}C-NMR (101 Mhz, methanol-*d₄*)

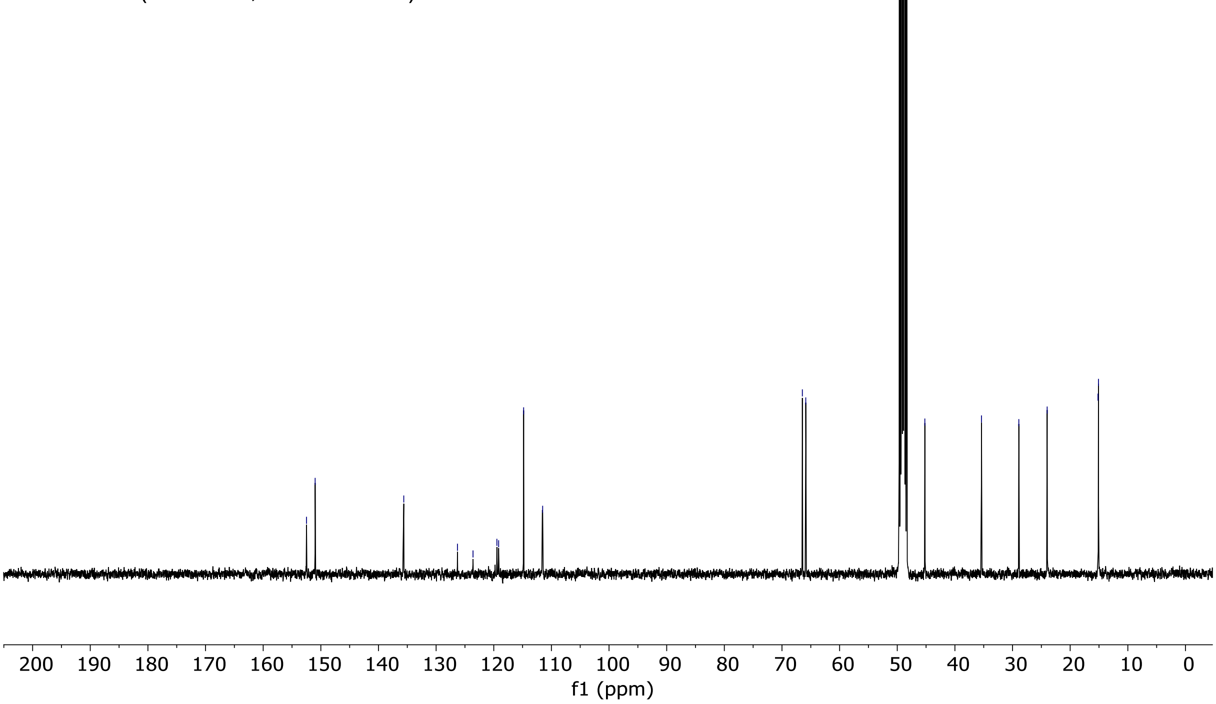

HPLC Chromatograms for Compounds 5-10

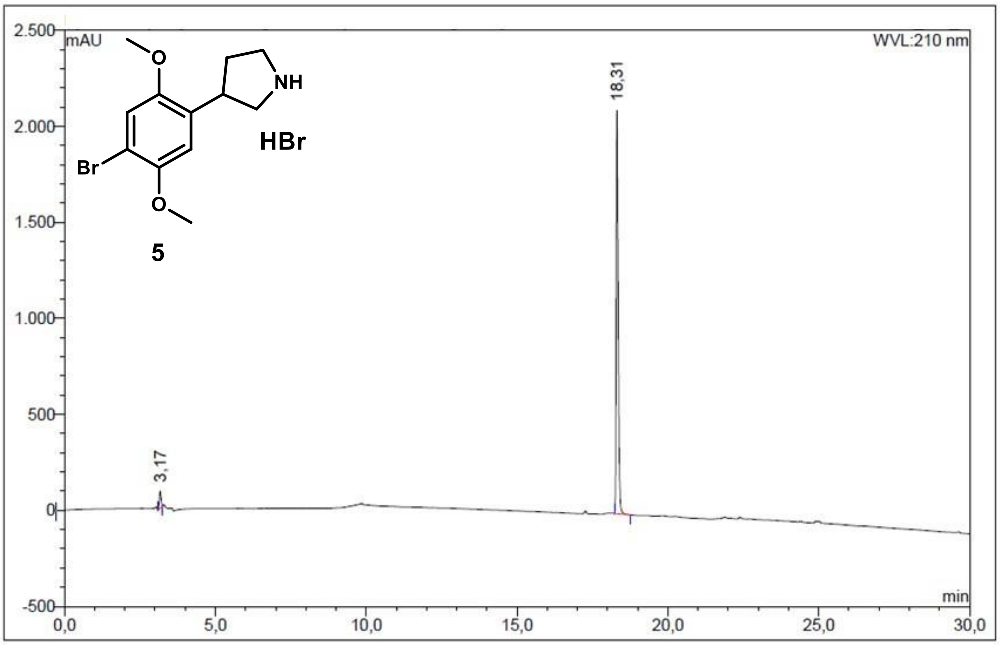

No.	Ret.Time min	Peak Name	Height mAU	Area mAU*min	Rel.Area %	Amount	Resolution(EP)
1	3,17	n.a.	91,095	6,418	3,74	n.a.	127,18
2	18,31	n.a.	2101,434	165,106	96,26	n.a.	n.a.
Total:			2192,529	171,525	100,00	0,000	

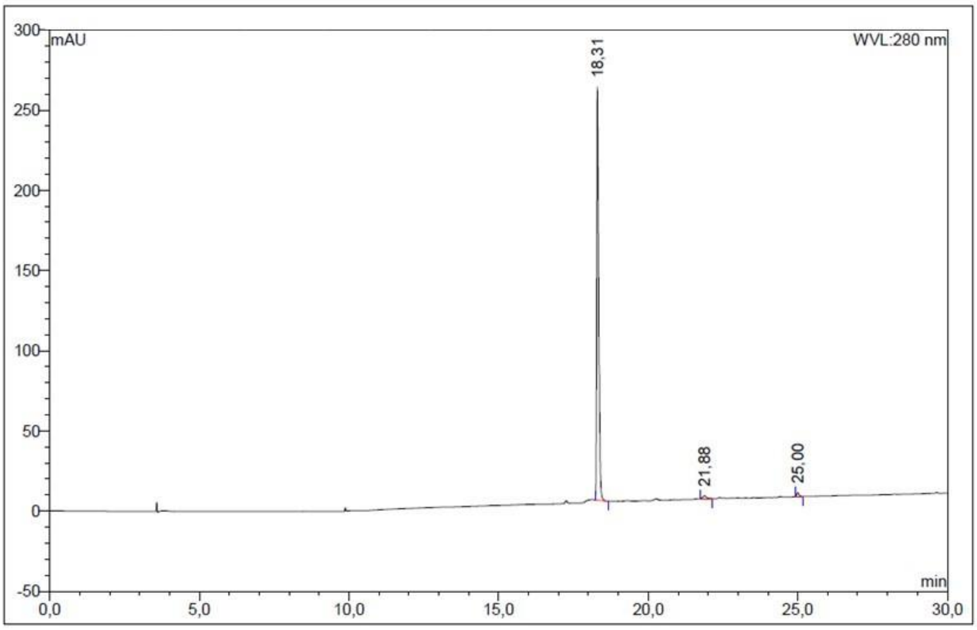

No.	Ret.Time min	Peak Name	Height mAU	Area mAU*min	Rel.Area %	Amount	Resolution(EP)
1	18,31	n.a.	257,773	19,592	97,85	n.a.	20,57
2	21,88	n.a.	1,642	0,253	1,26	n.a.	17,49
3	25,00	n.a.	2,240	0,179	0,89	n.a.	n.a.
Total:			261,655	20,023	100,00	0,000	

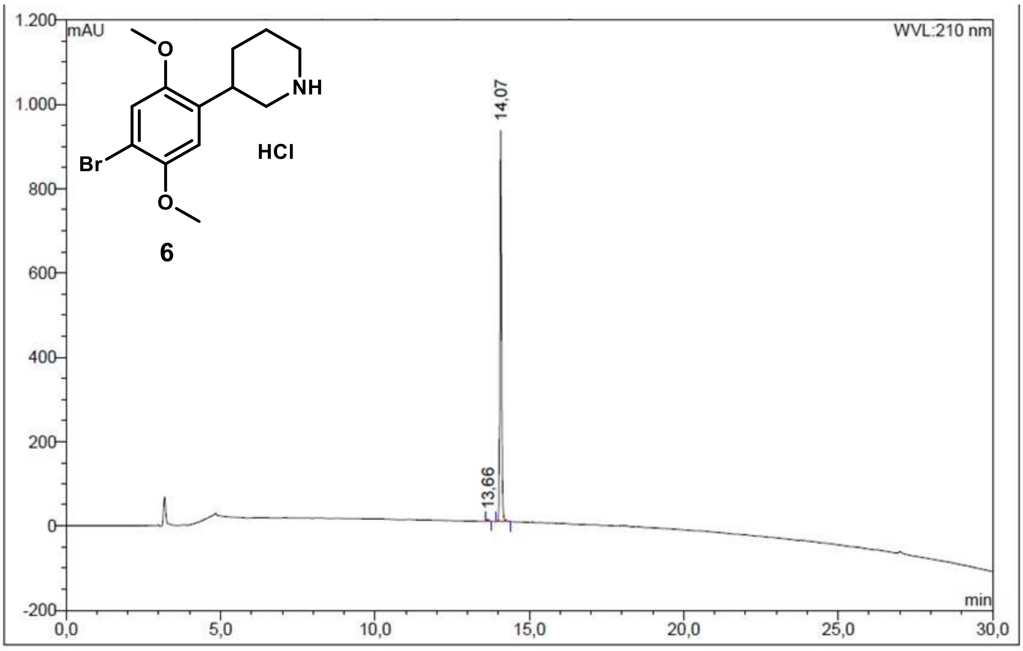

No.	Ret.Time min	Peak Name	Height mAU	Area mAU*min	Rel.Area %	Amount	Resolution(EP)
1	13,66	n.a.	4,537	0,308	0,54	n.a.	4,15
2	14,07	n.a.	928,125	57,267	99,46	n.a.	n.a.
Total:			932,662	57,575	100,00	0,000	

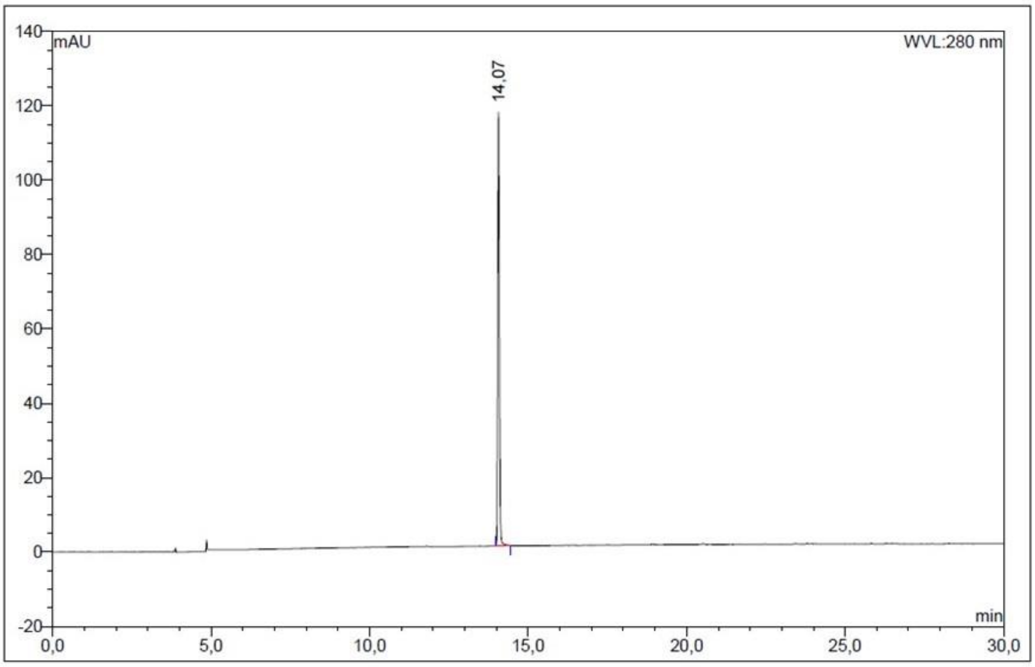

No.	Ret.Time min	Peak Name	Height mAU	Area mAU*min	Rel.Area %	Amount	Resolution(EP)
1	14,07	n.a.	116,654	7,115	100,00	n.a.	n.a.
Total:			116,654	7,115	100,00	0,000	

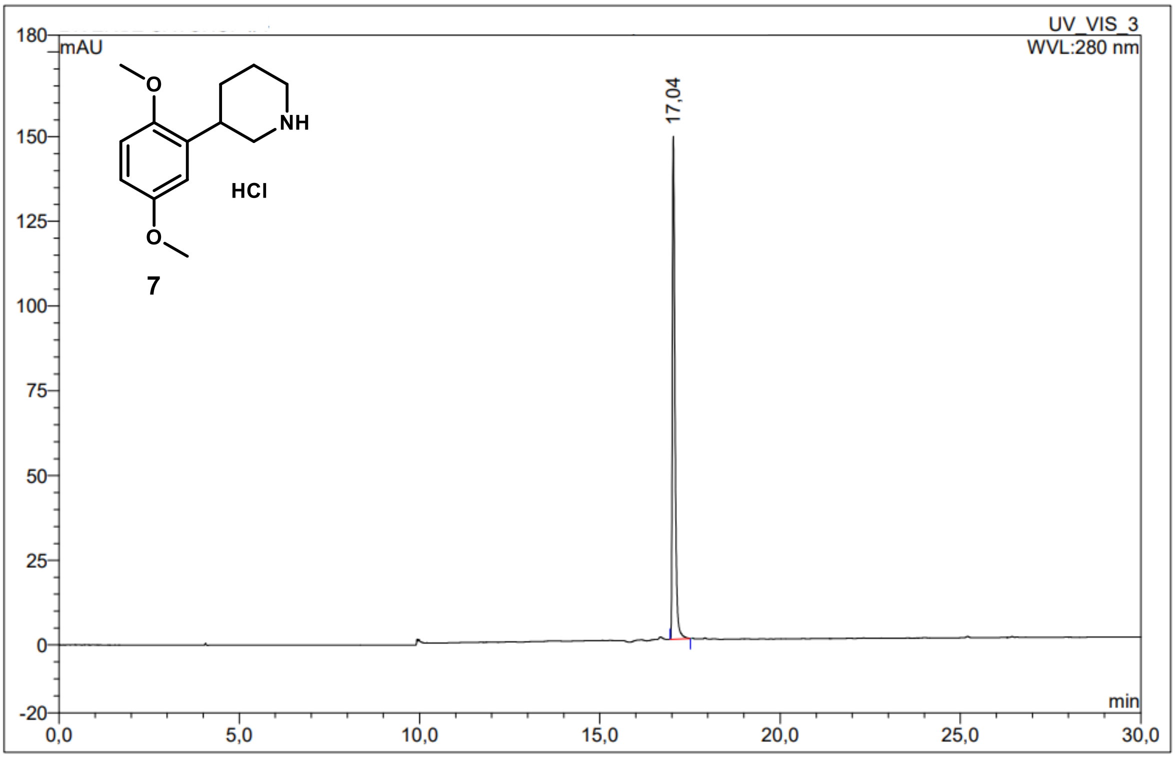

No.	Ret.Time min	Peak Name	Height mAU	Area mAU*min	Rel.Area %	Amount	Resolution(EP)
1	17,04	n.a.	148,479	12,884	100,00	n.a.	n.a.
Total:			148,479	12,884	100,00	0,000	

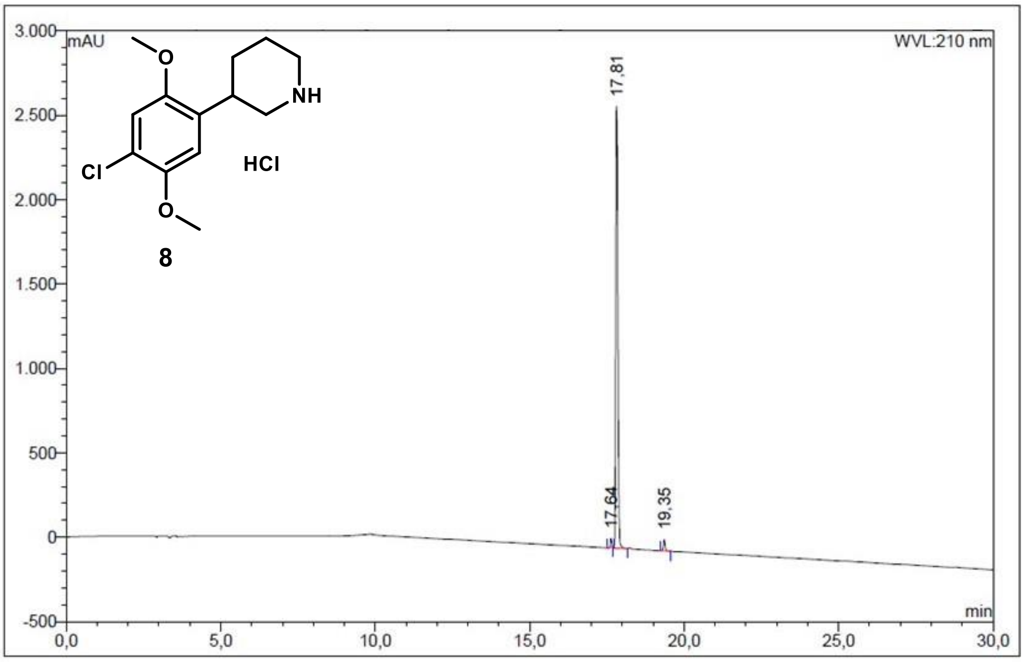

No.	Ret.Time min	Peak Name	Height mAU	Area mAU*min	Rel.Area %	Amount	Resolution(EP)
1	17,64	n.a.	52,737	2,499	1,23	n.a.	1,75
2	17,81	n.a.	2617,884	197,121	96,84	n.a.	14,64
3	19,35	n.a.	68,342	3,940	1,94	n.a.	n.a.
Total:			2738,963	203,561	100,00	0,000	

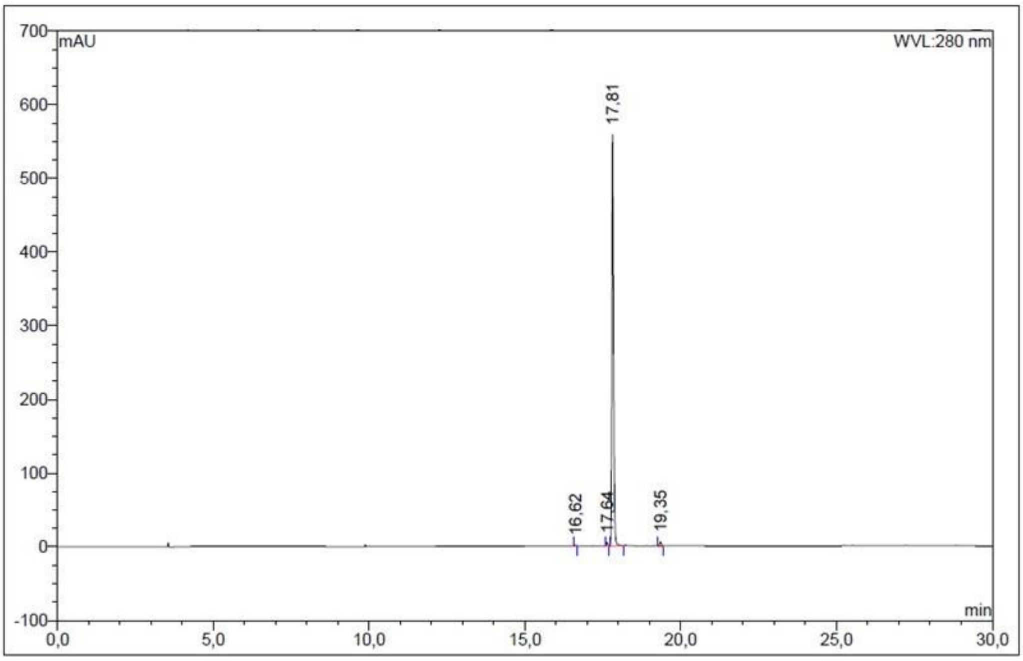

No.	Ret.Time min	Peak Name	Height mAU	Area mAU*min	Rel.Area %	Amount	Resolution(EP)
1	16,62	n.a.	0,823	0,050	0,13	n.a.	11,00
2	17,64	n.a.	4,602	0,239	0,64	n.a.	1,89
3	17,81	n.a.	558,420	36,745	98,45	n.a.	16,08
4	19,35	n.a.	5,230	0,291	0,78	n.a.	n.a.
Total:			569,076	37,325	100,00	0,000	

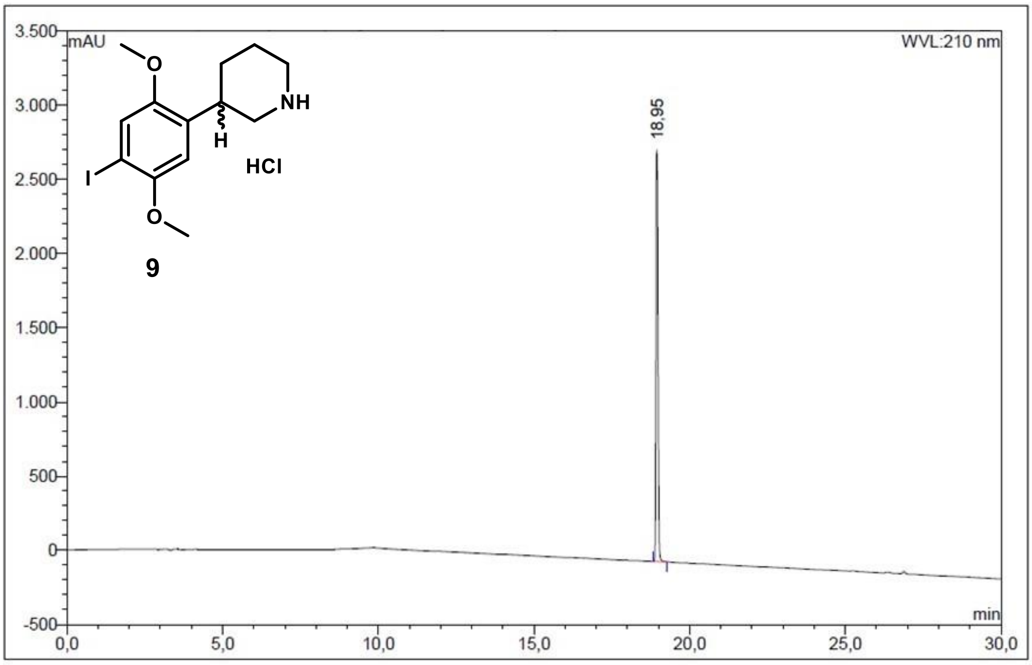

No.	Ret.Time min	Peak Name	Height mAU	Area mAU*min	Rel.Area %	Amount	Resolution(EP)
1	18,95	n.a.	2776,597	196,345	100,00	n.a.	n.a.
Total:			2776,597	196,345	100,00	0,000	

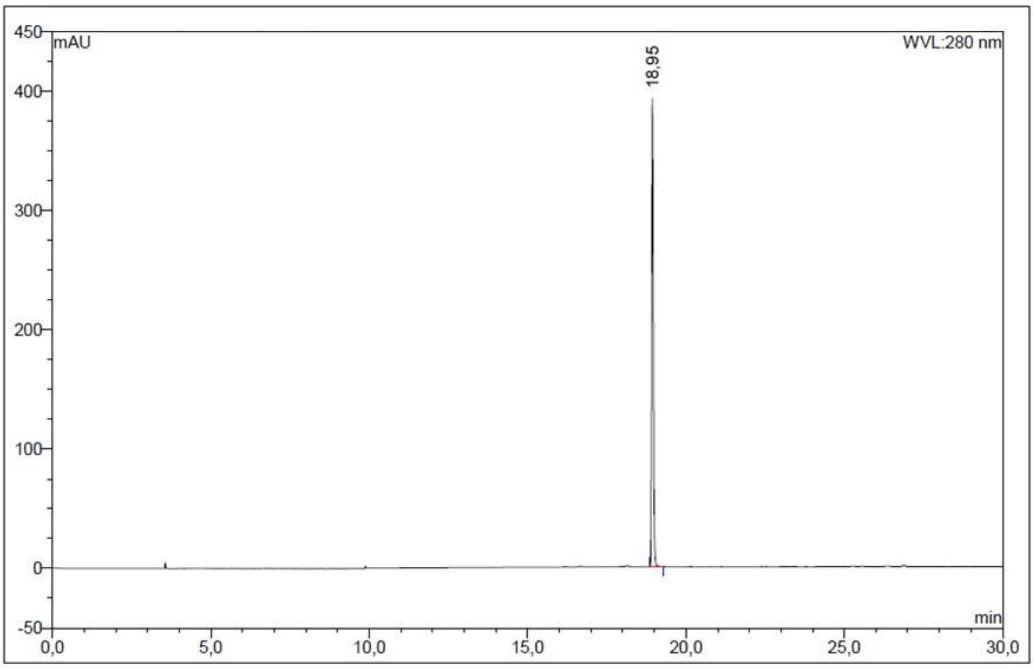

No.	Ret.Time min	Peak Name	Height mAU	Area mAU*min	Rel.Area %	Amount	Resolution(EP)
1	18,95	n.a.	393,061	24,464	100,00	n.a.	n.a.
Total:			393,061	24,464	100,00	0,000	

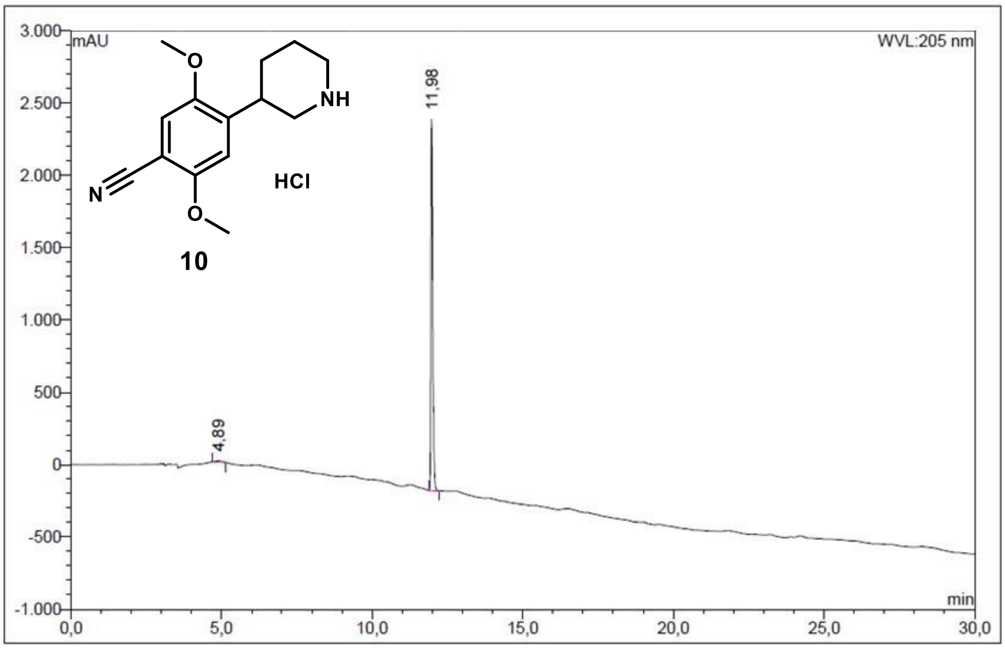

No.	Ret.Time min	Peak Name	Height mAU	Area mAU*min	Rel.Area %	Amount	Resolution(EP)
1	4,89	n.a.	8,494	2,366	1,29	n.a.	23,46
2	11,98	n.a.	2566,620	181,309	98,71	n.a.	n.a.
Total:			2575,114	183,675	100,00	0,000	

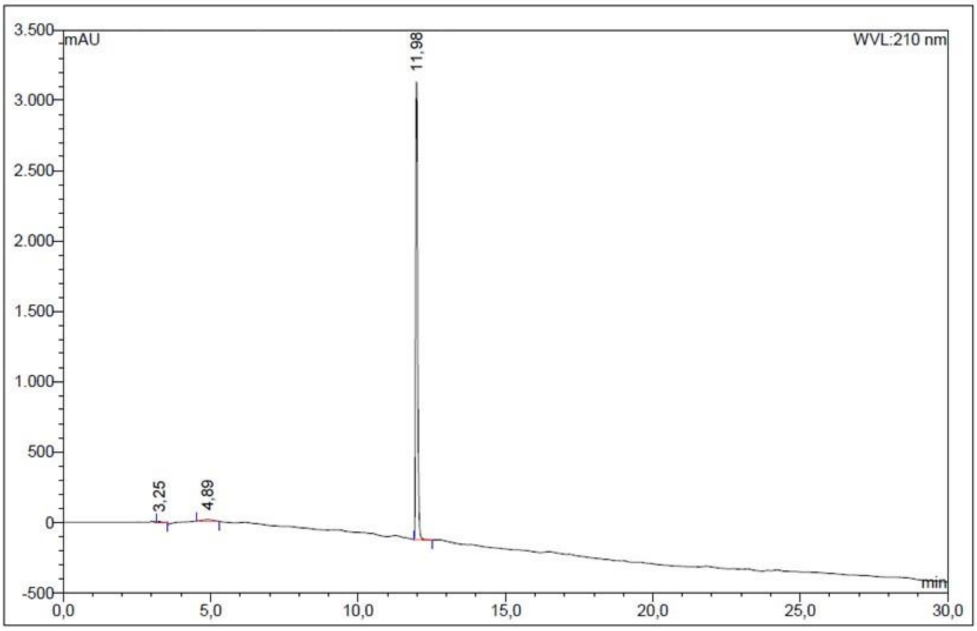

No.	Ret.Time min	Peak Name	Height mAU	Area mAU*min	Rel.Area %	Amount	Resolution(EP)
1	3,25	n.a.	8,399	1,581	0,65	n.a.	3,54
2	4,89	n.a.	11,433	4,922	2,01	n.a.	16,67
3	11,98	n.a.	3256,569	237,789	97,34	n.a.	n.a.
Total:			3276,401	244,291	100,00	0,000	

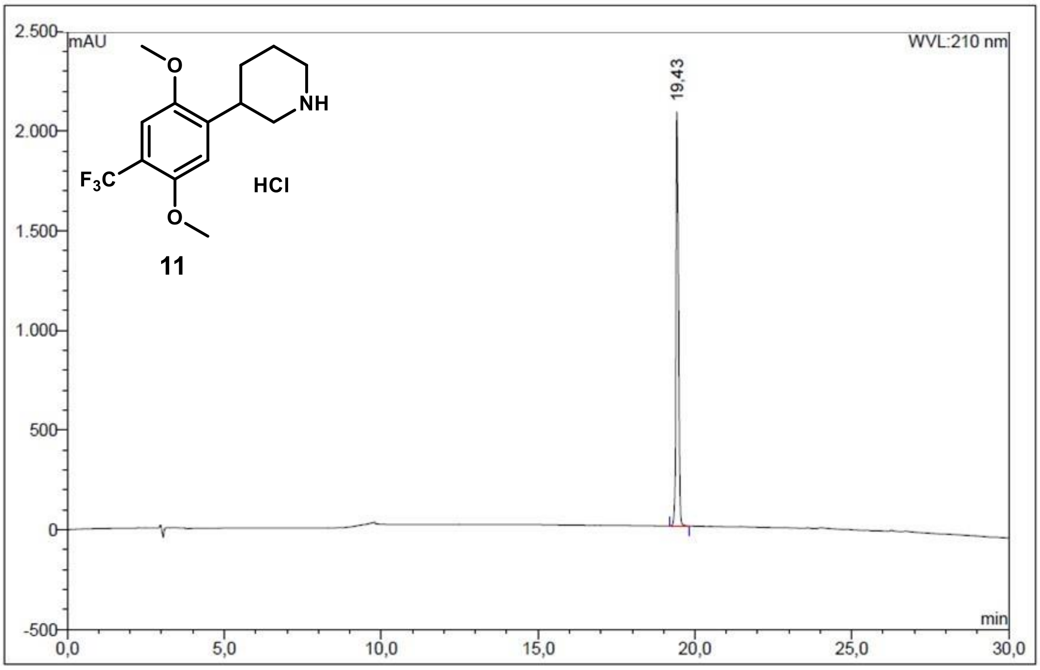

No.	Ret.Time min	Peak Name	Height mAU	Area mAU*min	Rel.Area %	Amount	Resolution(EP)
1	19,43	n.a.	2078,183	179,528	100,00	n.a.	n.a.
Total:			2078,183	179,528	100,00	0,000	

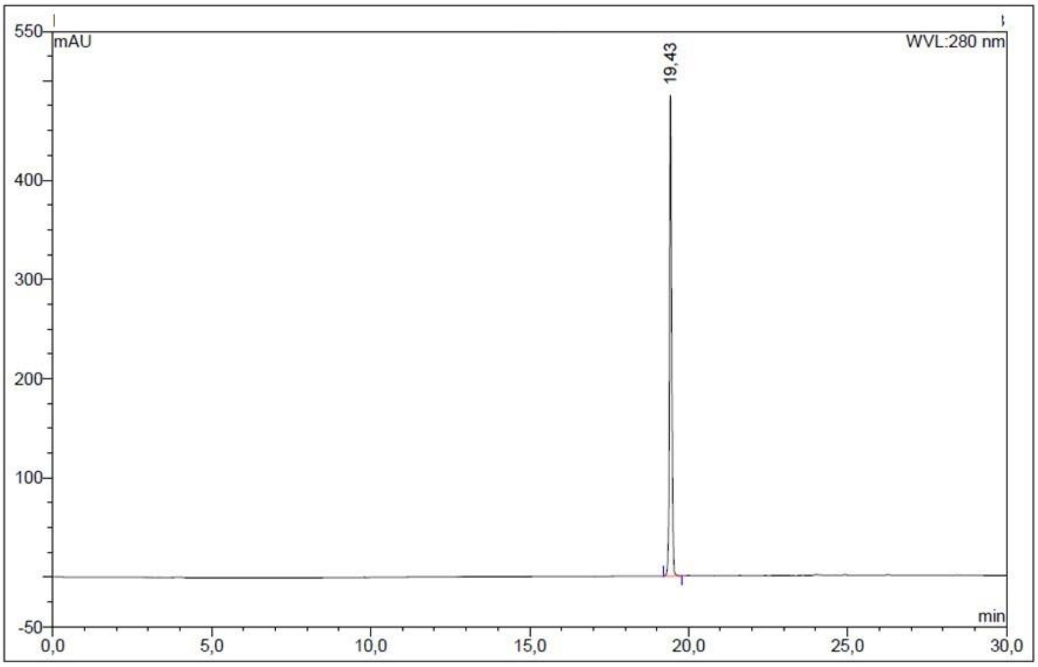

No.	Ret.Time min	Peak Name	Height mAU	Area mAU*min	Rel.Area %	Amount	Resolution(EP)
1	19,43	n.a.	484,777	39,247	100,00	n.a.	n.a.
Total:			484,777	39,247	100,00	0,000	

Chiral HPLC Chromatograms for Compounds 5-6, 8-17 & 19-26

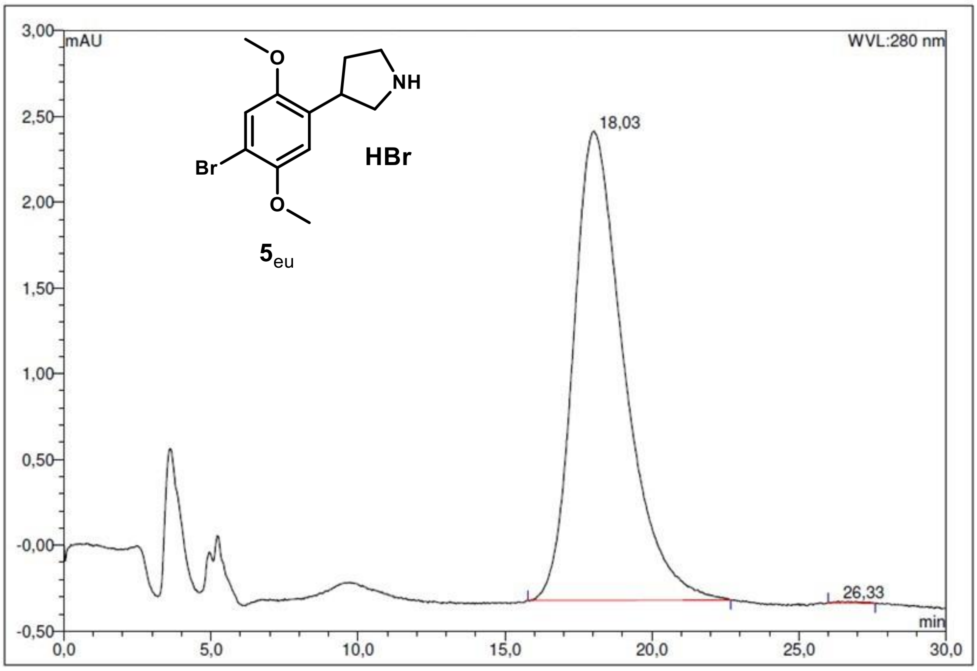

No.	Ret.Time min	Peak Name	Height mAU	Area mAU*min	Rel.Area %	Amount	Type
1	18,03	n.a.	2,734	5,473	99,87	n.a.	BMB*
2	26,33	n.a.	0,009	0,007	0,13	n.a.	BMB*
Total:			2,743	5,480	100,00	0,000	

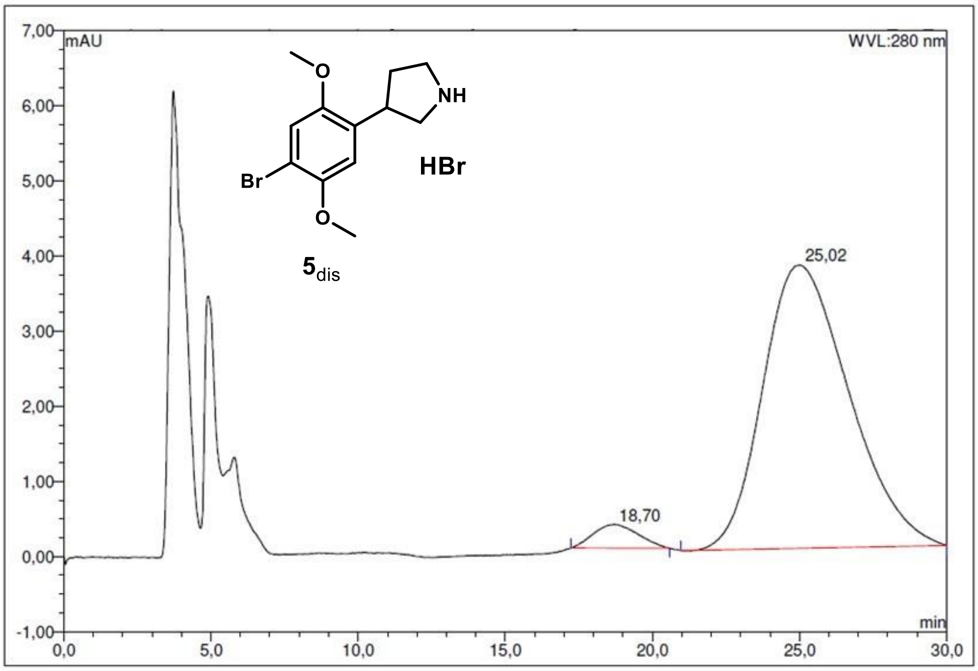

No.	Ret.Time min	Peak Name	Height mAU	Area mAU*min	Rel.Area %	Amount	Type
1	18,70	n.a.	0,318	0,554	4,15	n.a.	BMB*
2	25,02	n.a.	3,771	12,799	95,85	n.a.	BMB*
Total:			4,089	13,353	100,00	0,000	

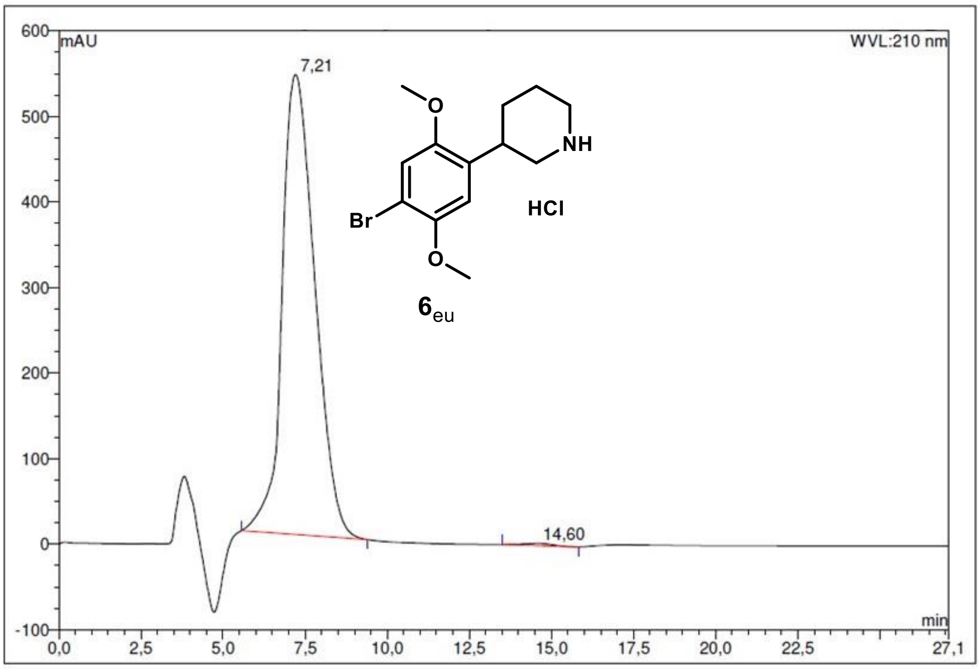

No.	Ret.Time min	Peak Name	Height mAU	Area mAU*min	Rel.Area %	Amount	Type
1	7,21	n.a.	537,632	622,753	99,61	n.a.	BMB*
2	14,60	n.a.	2,441	2,438	0,39	n.a.	BMB*
Total:			540,073	625,191	100,00	0,000	

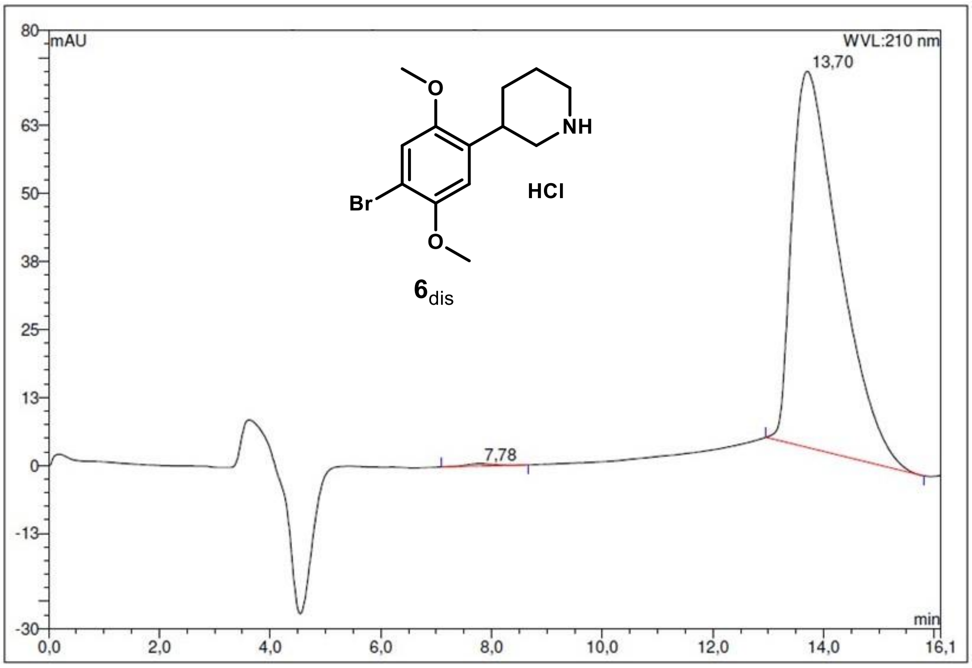

No.	Ret.Time min	Peak Name	Height mAU	Area mAU*min	Rel.Area %	Amount	Type
1	7,78	n.a.	0,441	0,231	0,33	n.a.	BMB*
2	13,70	n.a.	69,198	68,875	99,67	n.a.	BMB*
Total:			69,639	69,106	100,00	0,000	

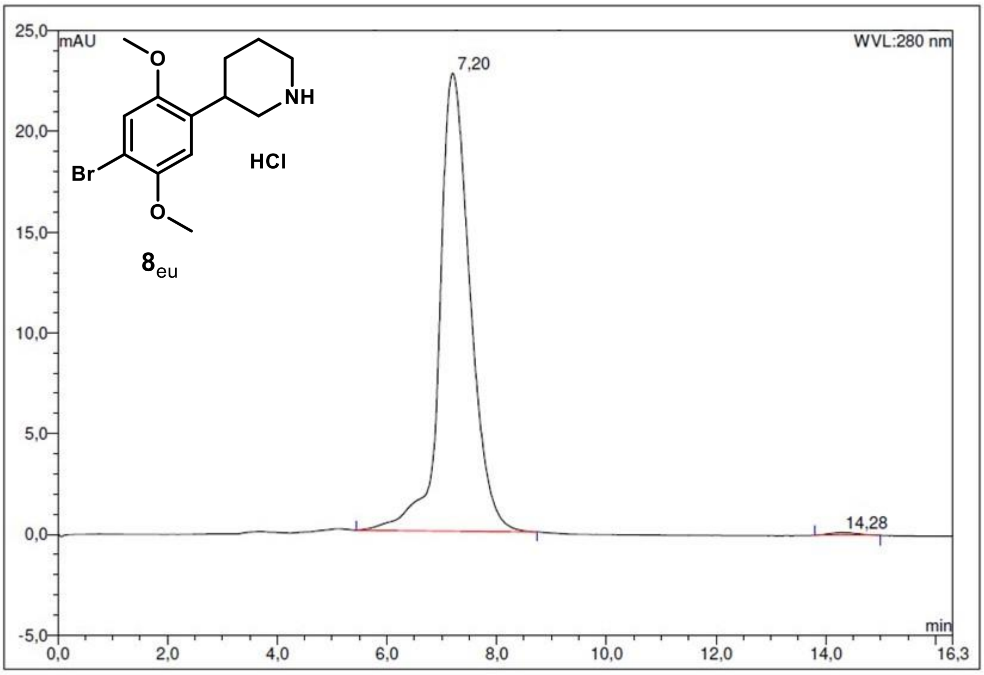

No.	Ret.Time min	Peak Name	Height mAU	Area mAU*min	Rel.Area %	Amount	Type
1	7,20	n.a.	22,739	14,724	99,44	n.a.	BMB
2	14,28	n.a.	0,139	0,083	0,56	n.a.	BMB*
Total:			22,878	14,807	100,00	0,000	

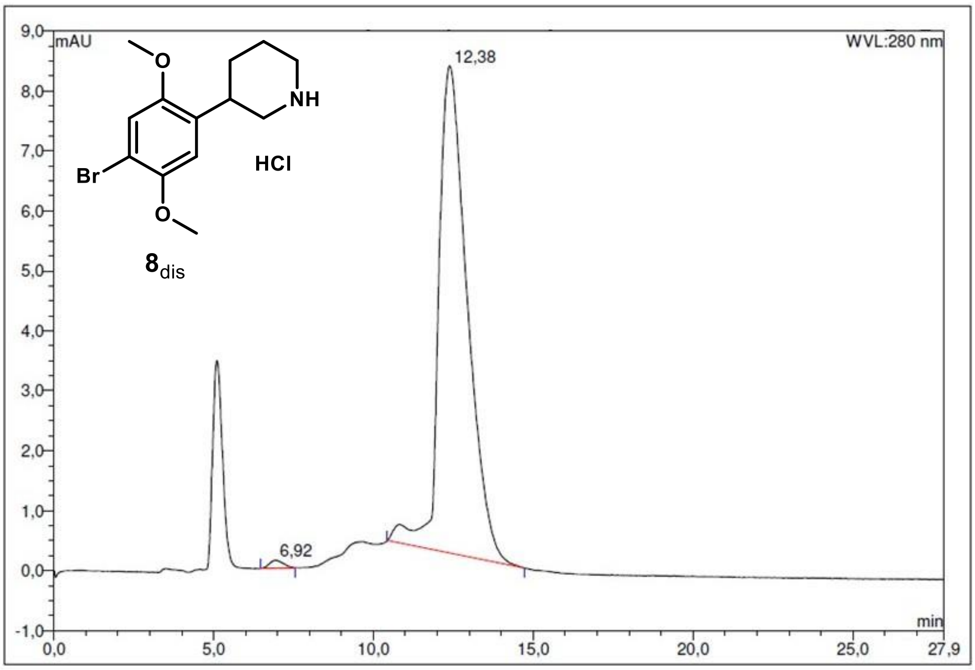

No.	Ret.Time min	Peak Name	Height mAU	Area mAU*min	Rel.Area %	Amount	Type
1	6,92	n.a.	0,133	0,066	0,77	n.a.	BMB*
2	12,38	n.a.	8,125	8,475	99,23	n.a.	BMB*
Total:			8,257	8,541	100,00	0,000	

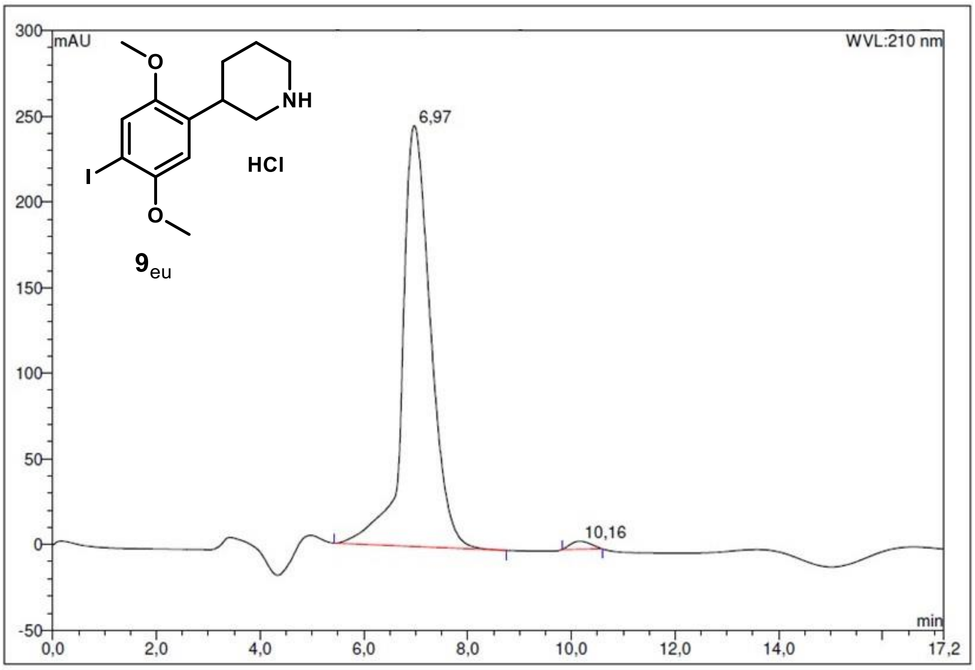

No.	Ret.Time min	Peak Name	Height mAU	Area mAU*min	Rel.Area %	Amount	Type
1	6,97	n.a.	245,697	155,342	98,67	n.a.	BMB*
2	10,16	n.a.	4,666	2,099	1,33	n.a.	BMB*
Total:			250,363	157,440	100,00	0,000	

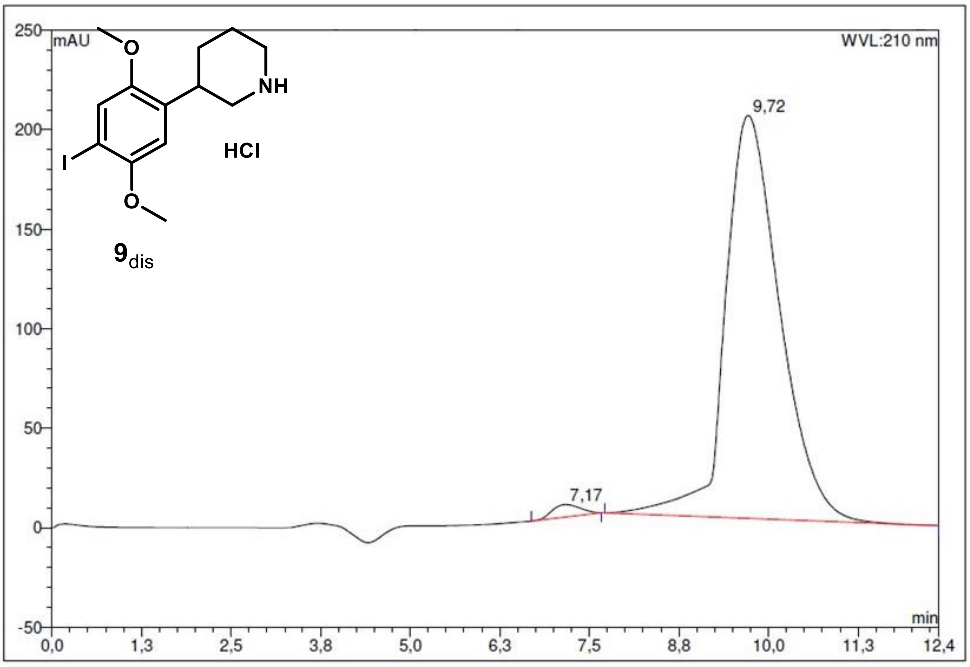

No.	Ret.Time min	Peak Name	Height mAU	Area mAU*min	Rel.Area %	Amount	Type
1	7,17	n.a.	6,240	2,849	1,57	n.a.	BMB*
2	9,72	n.a.	202,583	179,059	98,43	n.a.	BMB*
Total:			208,822	181,908	100,00	0,000	

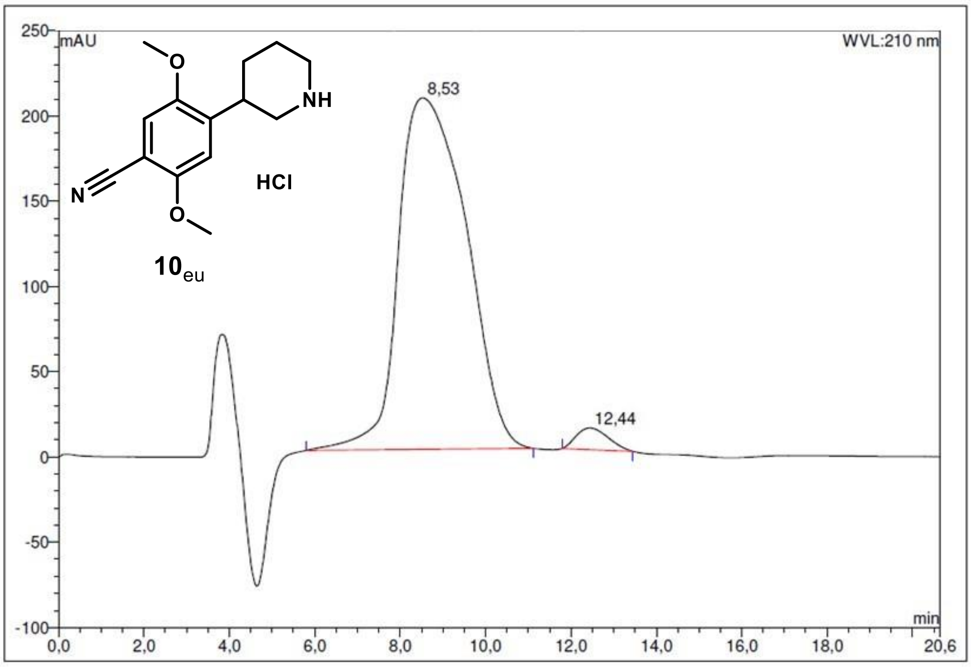

No.	Ret.Time min	Peak Name	Height mAU	Area mAU*min	Rel.Area %	Amount	Type
1	8,53	n.a.	206,075	379,839	97,19	n.a.	BMB*
2	12,44	n.a.	12,662	10,981	2,81	n.a.	BMB*
Total:			218,737	390,820	100,00	0,000	

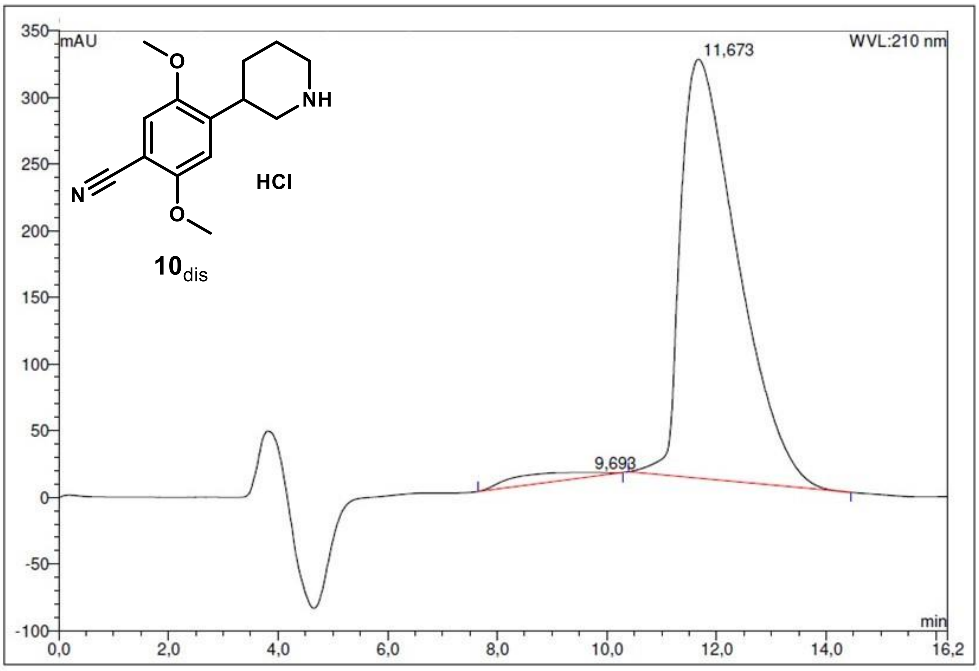

No.	Ret.Time min	Peak Name	Height mAU	Area mAU*min	Rel.Area %	Amount	Type
1	9,69	n.a.	3,342	11,552	2,94	n.a.	BMB*
2	11,67	n.a.	314,378	380,796	97,06	n.a.	BMB*
Total:			317,720	392,349	100,00	0,000	

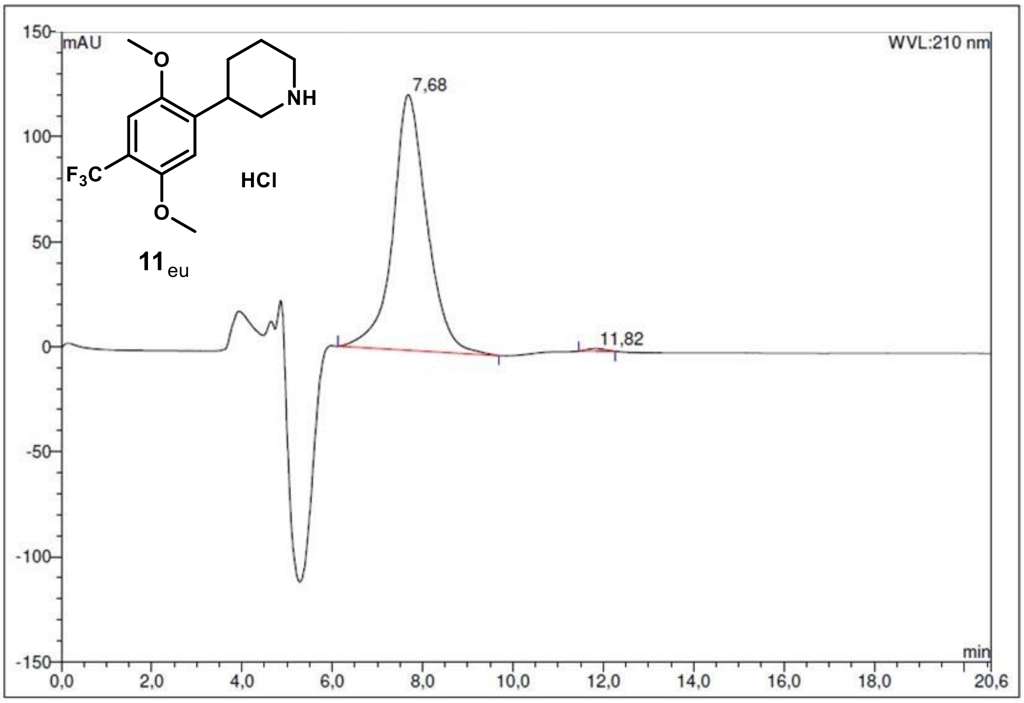

No.	Ret.Time min	Peak Name	Height mAU	Area mAU*min	Rel.Area %	Amount	Type
1	7,68	n.a.	121,557	105,994	99,48	n.a.	BMB*
2	11,82	n.a.	1,230	0,553	0,52	n.a.	BMB*
Total:			122,787	106,548	100,00	0,000	

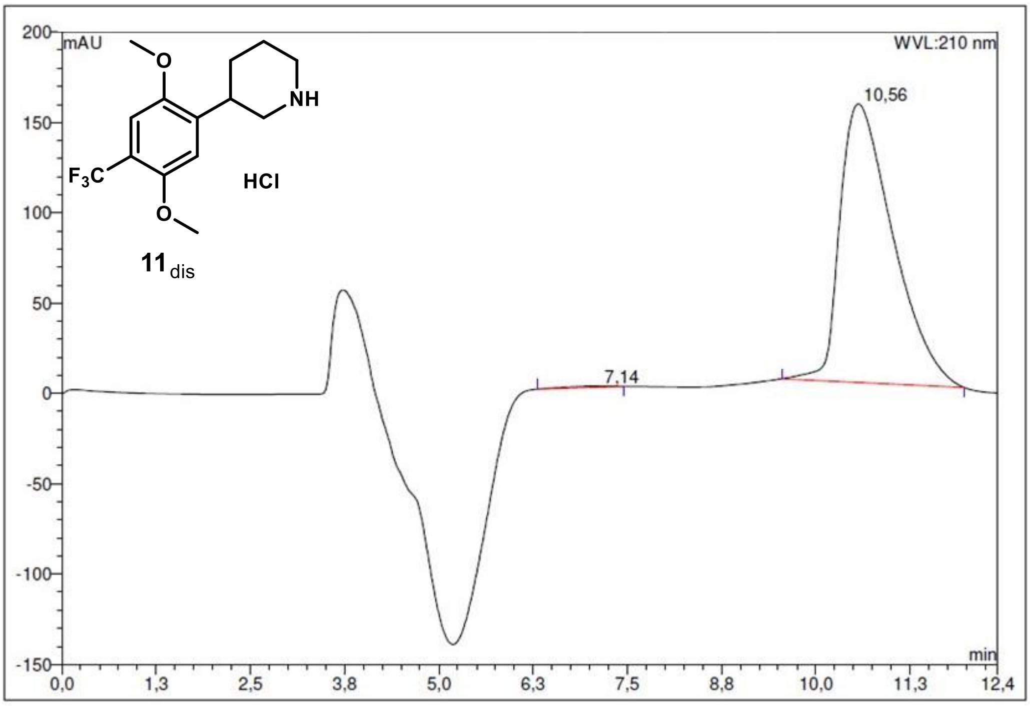

No.	Ret.Time min	Peak Name	Height mAU	Area mAU*min	Rel.Area %	Amount	Type
1	7,14	n.a.	0,528	0,502	0,40	n.a.	BMB*
2	10,56	n.a.	154,264	125,869	99,60	n.a.	BMB*
Total:			154,792	126,371	100,00	0,000	

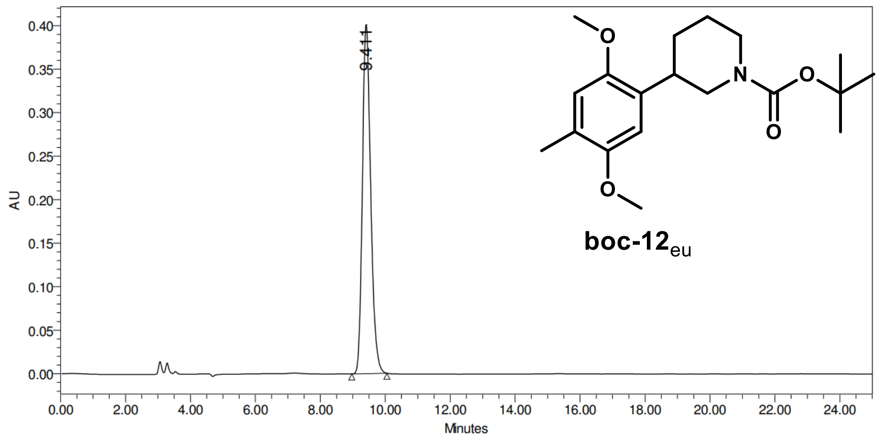

	RT	Height	Area	% Area
1	9.411	401753	7137632	100.00

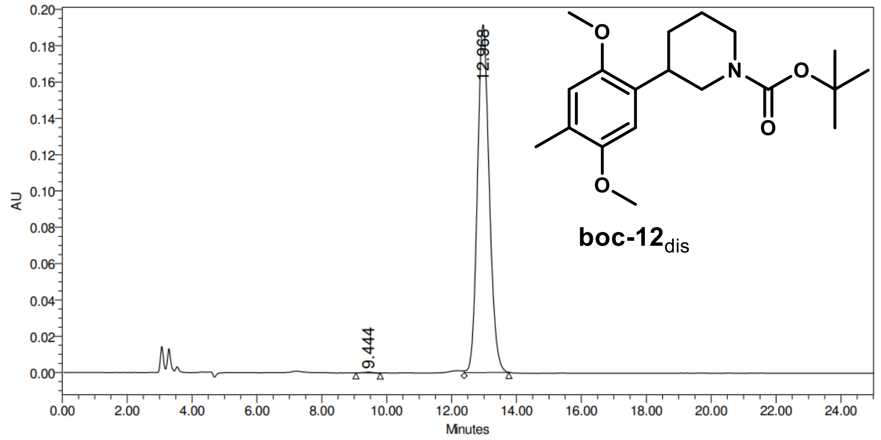

	RT	Height	Area	% Area
1	9.444	468	8040	0.17
2	12.968	191453	4841110	99.83

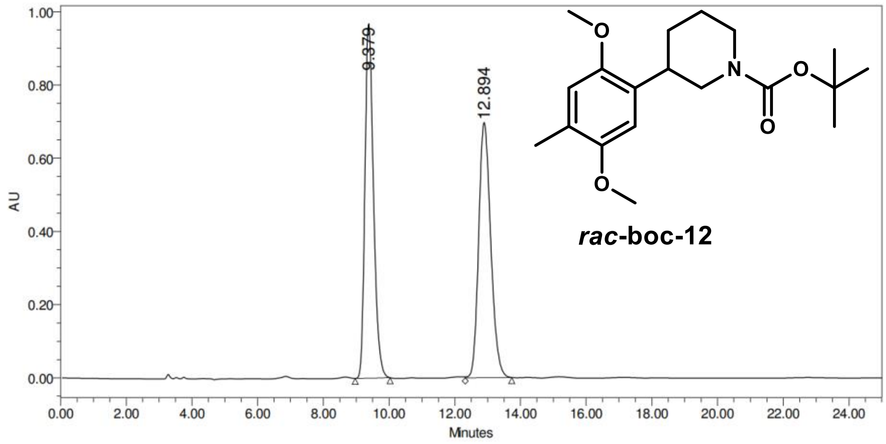

	RT	Height	Area	% Area
1	9.379	968504	17516097	49.58
2	12.894	697305	17815373	50.42

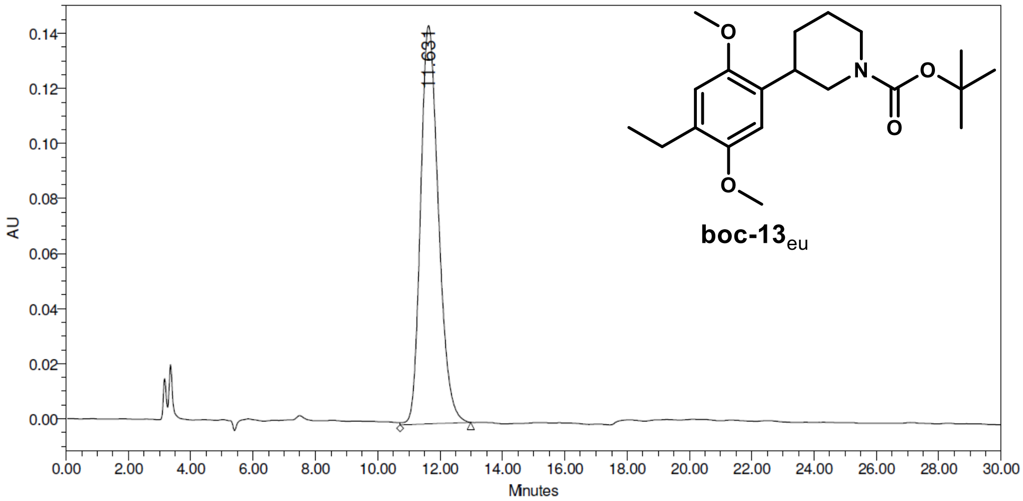

	RT	Height	Area	% Area
1	11.631	144825	5758810	100.00

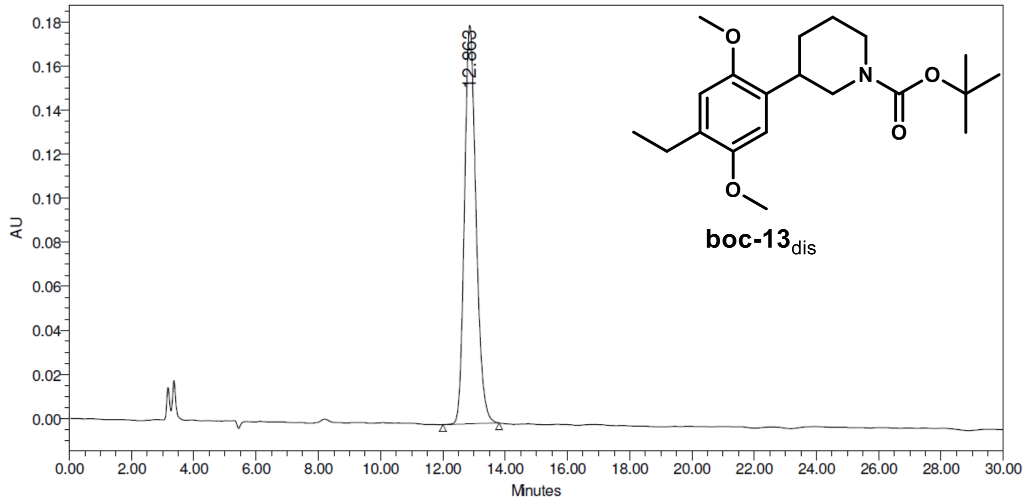

	RT	Height	Area	% Area
1	12.863	181022	4675751	100.00

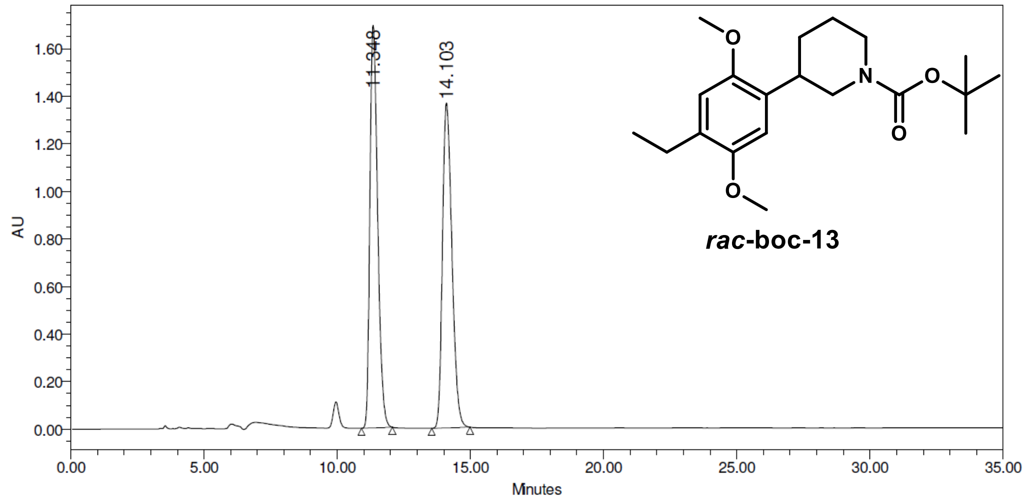

	RT	Height	Area	% Area
1	11.348	1692925	34180328	50.35
2	14.103	1364870	33701656	49.65

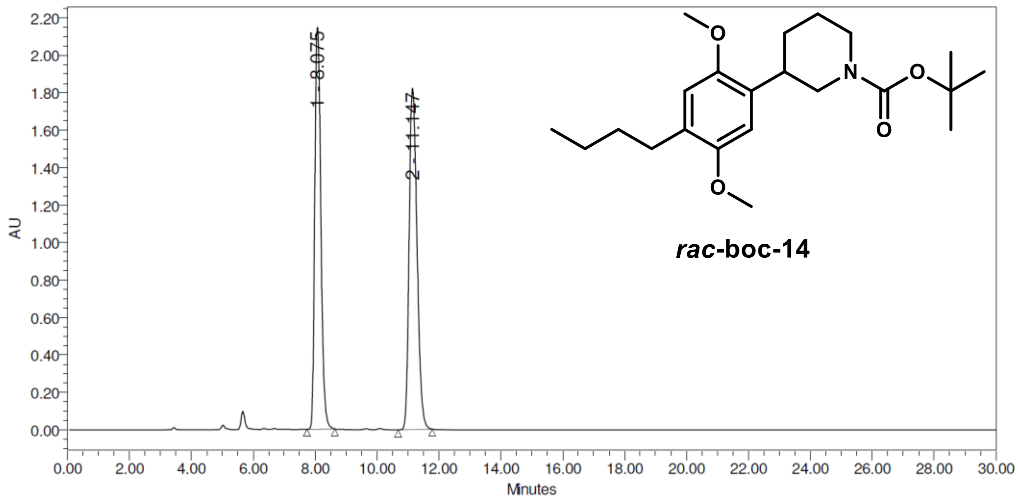

	Peak Name	RT	Height	Area	% Area
1	1	8.075	2154949	29214858	47.59
2	2	11.147	1824138	32172449	52.41

Chiral HPLC traces for individuel enantiomers 1 and 2 of compound **14** were not aquired.

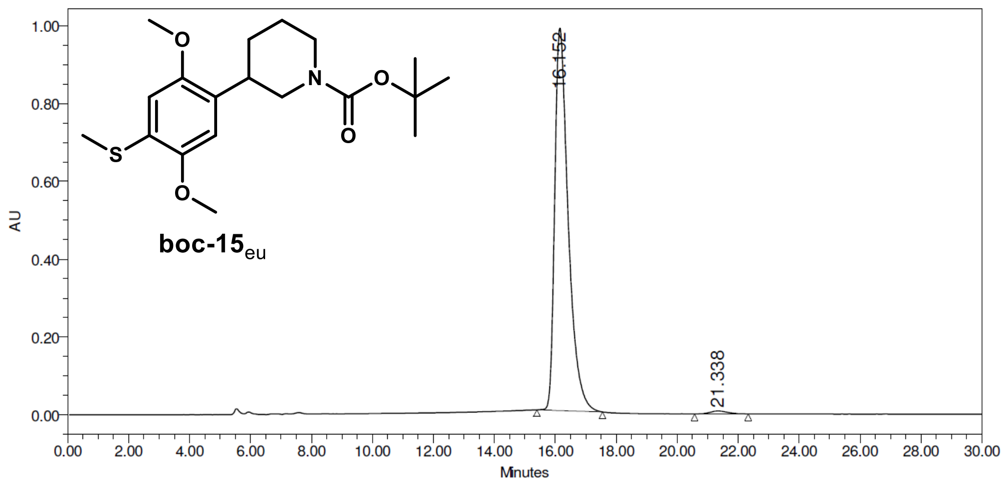

	RT	Height	Area	% Area
1	16.152	982144	30128769	99.00
2	21.338	7671	305086	1.00

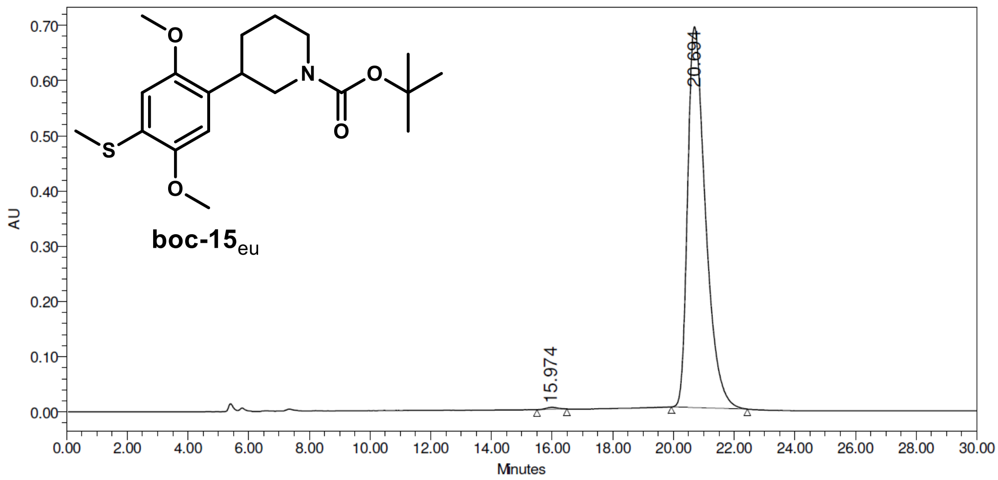

	RT	Height	Area	% Area
1	15.974	3476	94914	0.34
2	20.694	689600	28111150	99.66

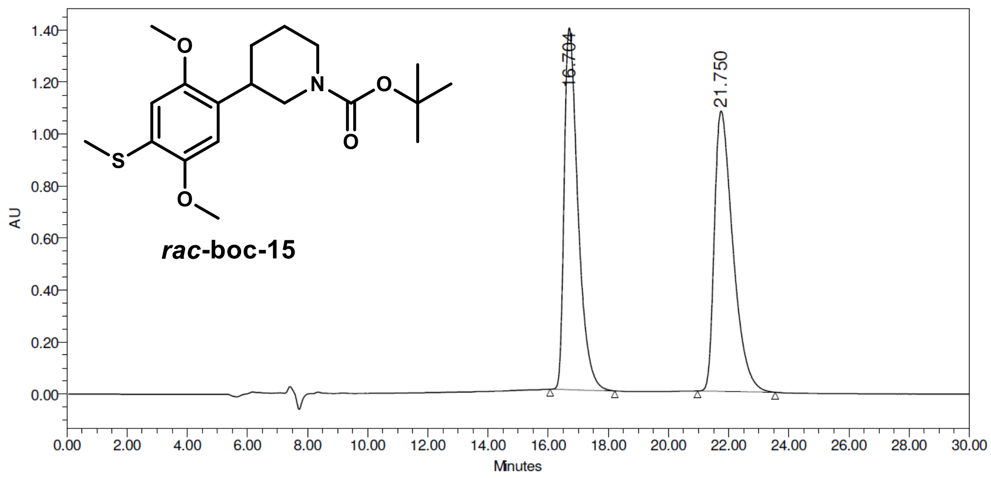

	RT	Height	Area	% Area
1	16.704	1391918	44400204	49.02
2	21.750	1077945	46171402	50.98

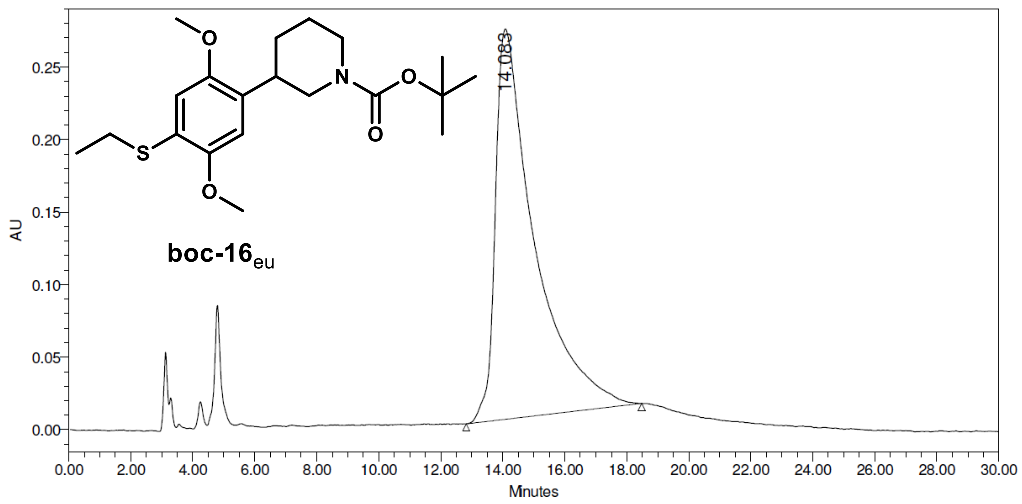

	RT	Height	Area	% Area
1	14.083	269197	23691666	100.00

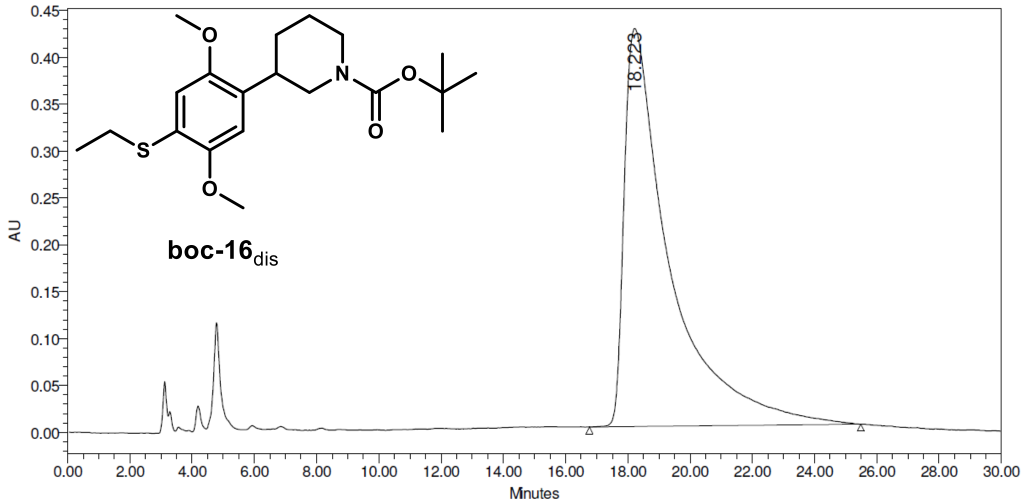

	RT	Height	Area	% Area
1	18.223	424465	43138441	100.00

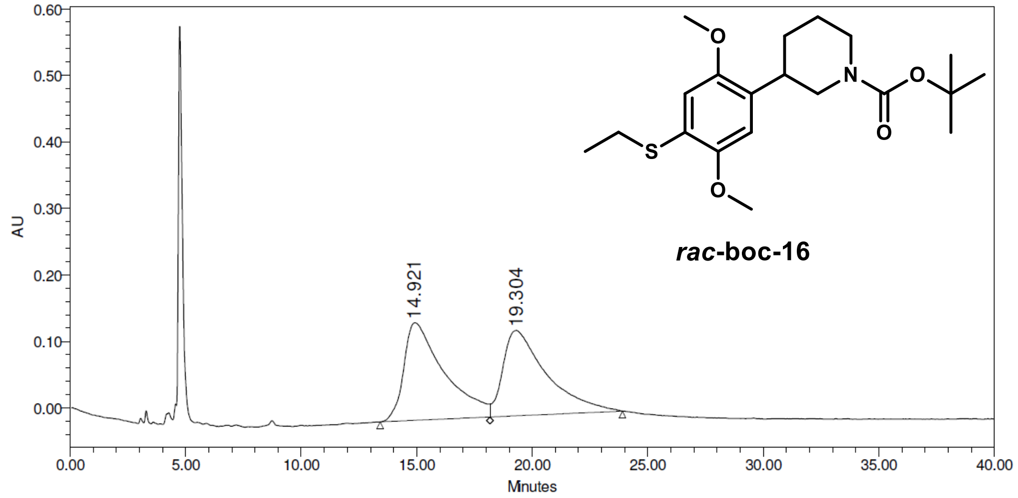

	RT	Height	Area	% Area
1	14.921	146513	17754304	51.20
2	19.304	128279	16921473	48.80

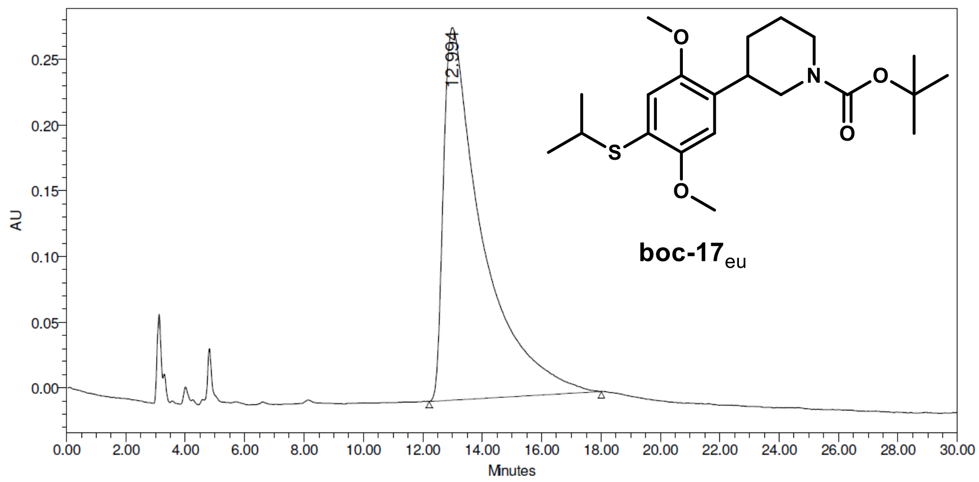

	RT	Height	Area	% Area
1	12.994	283803	25736628	100.00

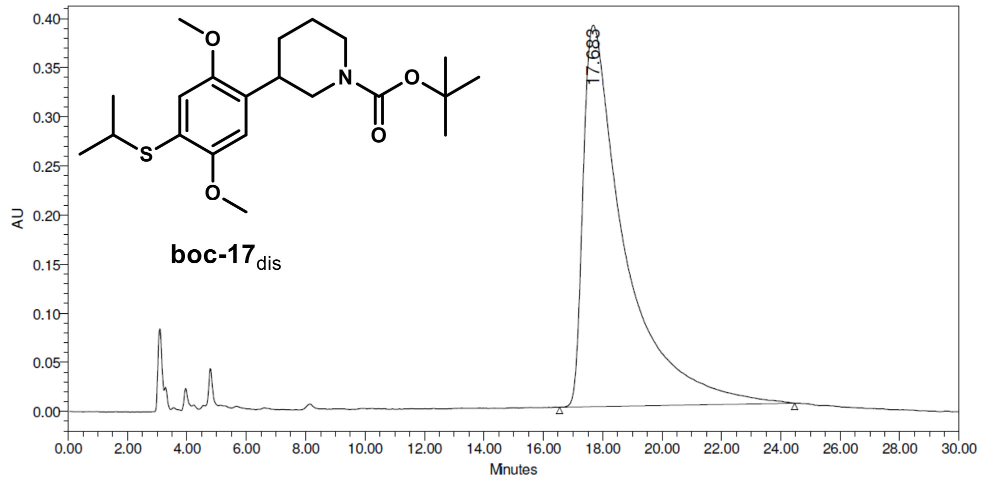

	RT	Height	Area	% Area
1	17.683	388426	37915467	100.00

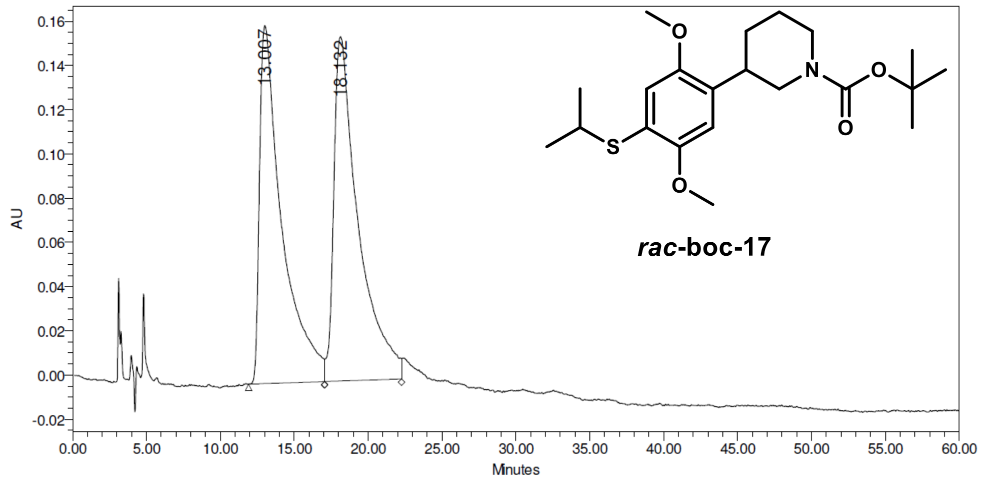

	RT	Height	Area	% Area
1	13.007	162065	16993510	48.65
2	18.132	155808	17938920	51.35

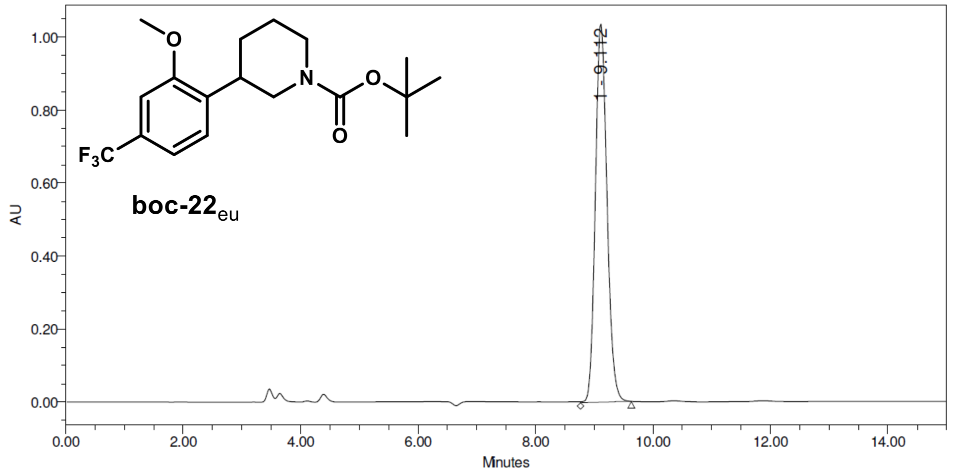

	Peak Name	RT	Height	Area	% Area
1	1	9.112	1037467	14343183	100.00
2	2	9.642			

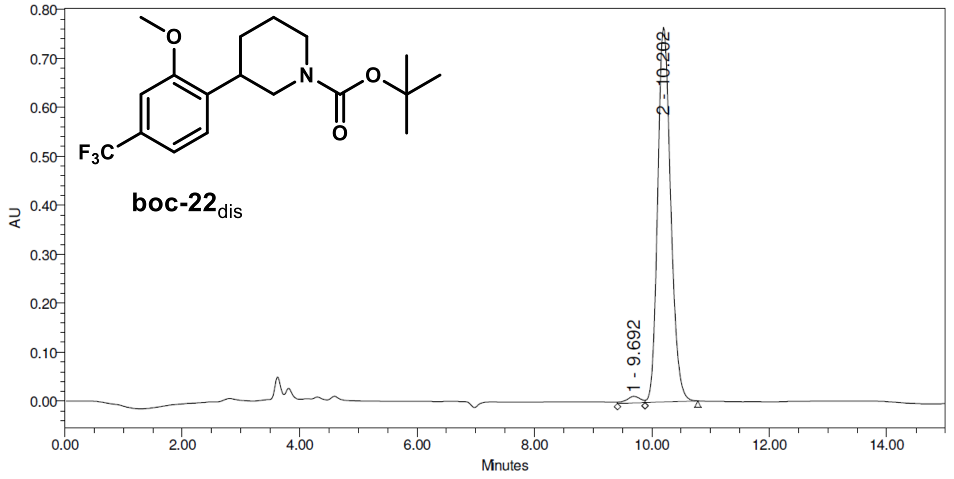

	Peak Name	RT	Height	Area	% Area
1	1	9.692	13178	213301	1.77
2	2	10.202	765529	11854252	98.23

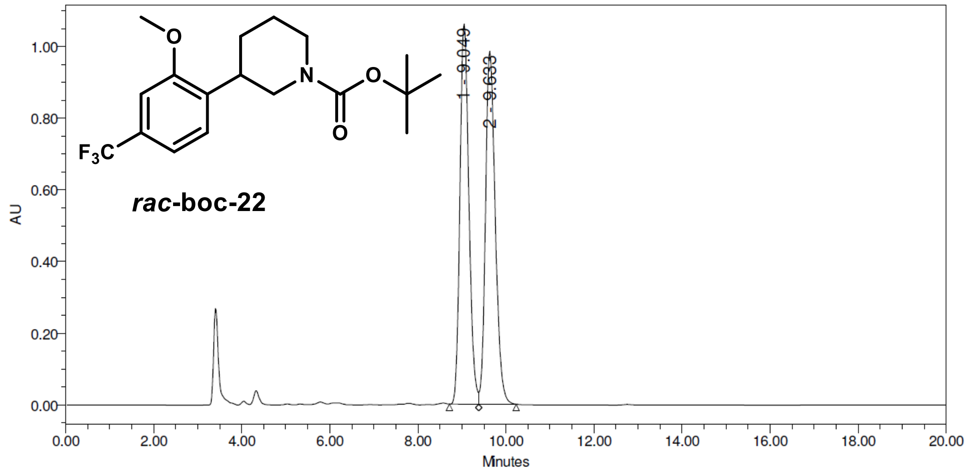

	Peak Name	RT	Height	Area	% Area
1	1	9.049	1060740	14986782	49.65
2	2	9.633	983806	15196937	50.35

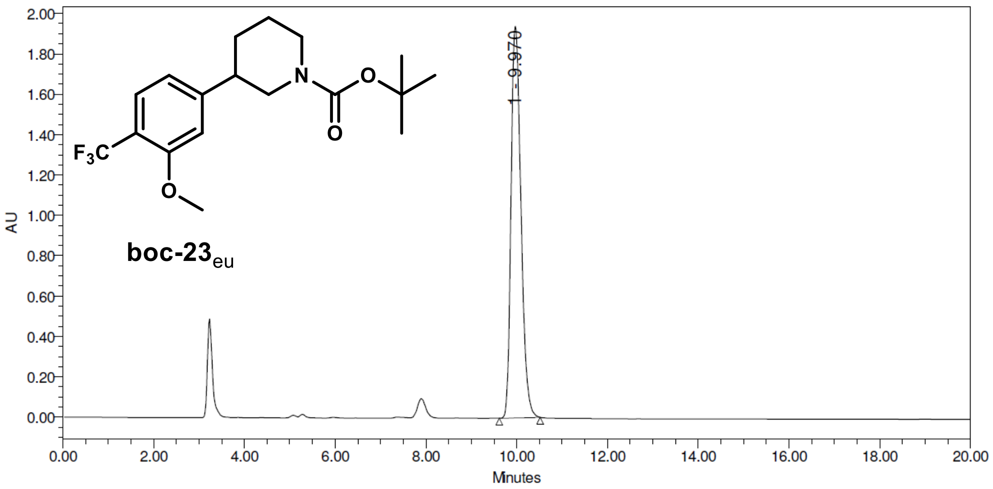

	Peak Name	RT	Height	Area	% Area
1	1	9.970	1942638	29360245	100.00
2	2	13.820			

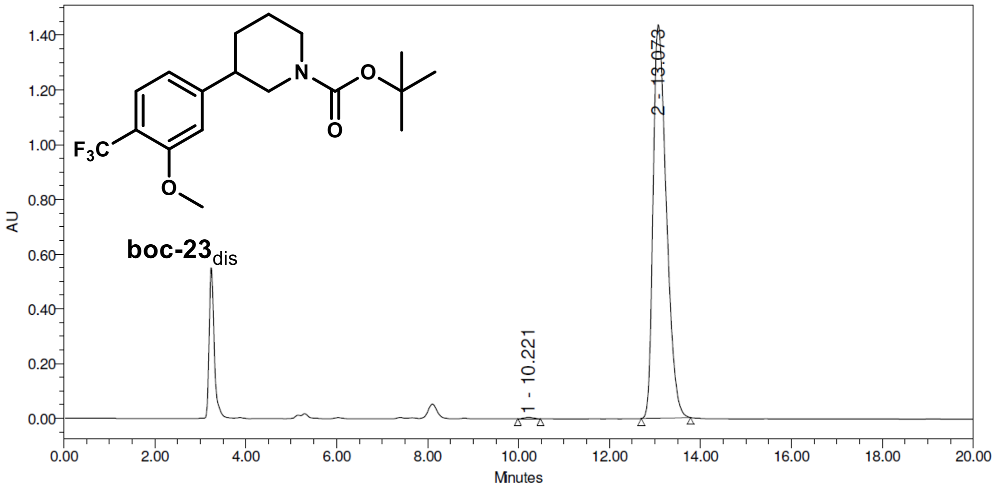

	Peak Name	RT	Height	Area	% Area
1	1	10.221	5131	66948	0.23
2	2	13.073	1440269	29096573	99.77

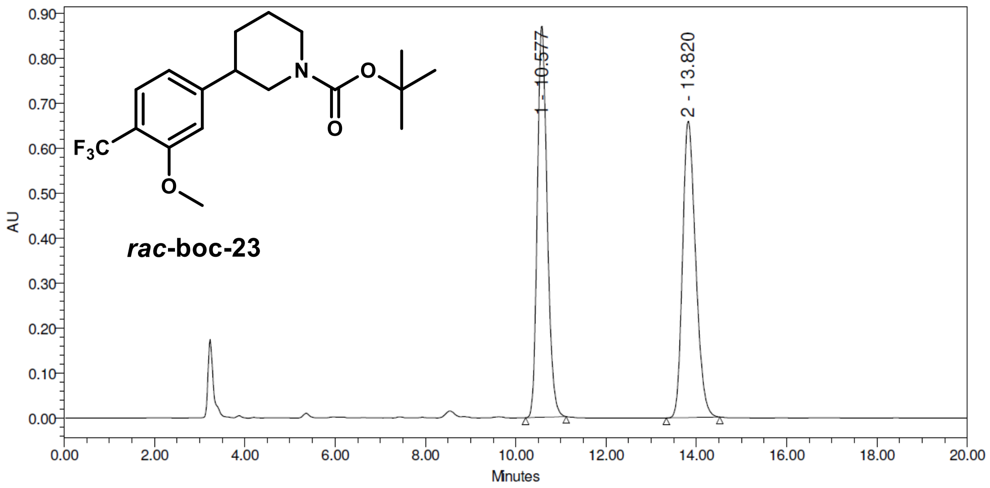

	Peak Name	RT	Height	Area	% Area
1	1	10.577	872500	13182937	49.83
2	2	13.820	659855	13272804	50.17

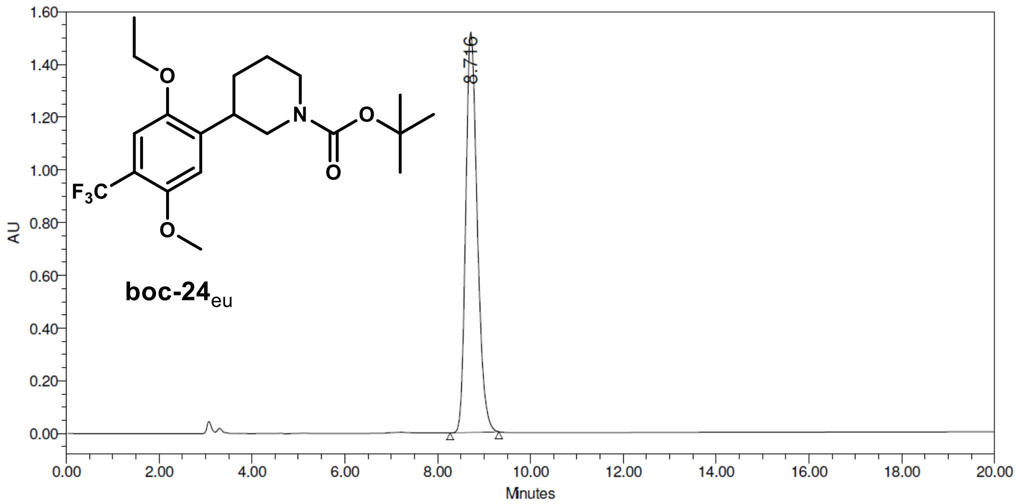

	RT	Height	Area	% Area
1	8.716	1519454	26233882	100.00

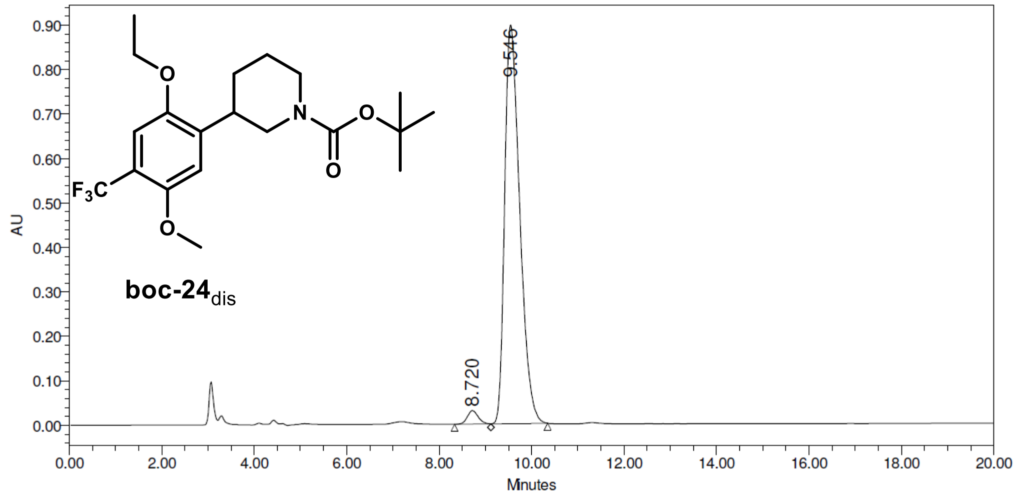

	RT	Height	Area	% Area
1	8.720	30126	486242	2.33
2	9.546	897557	20350342	97.67

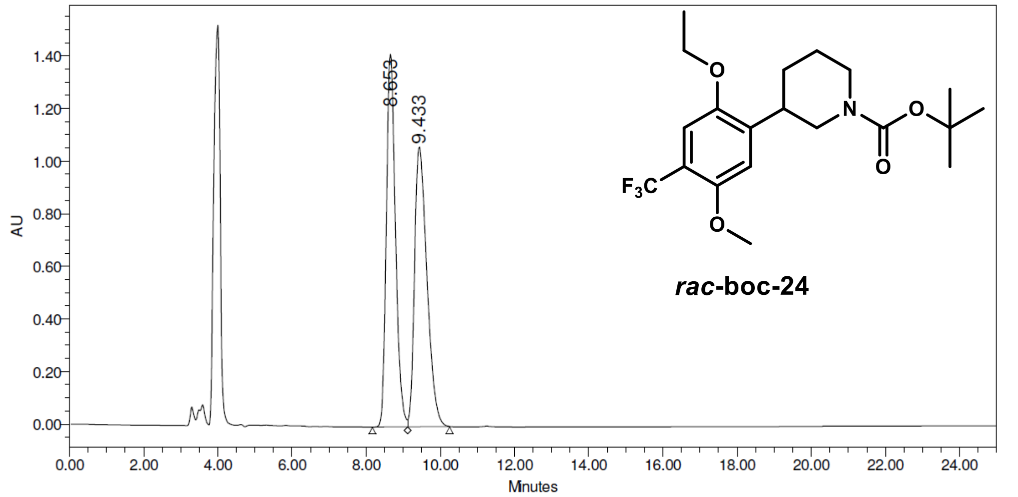

	RT	Height	Area	% Area
1	8.653	1417321	24399372	49.67
2	9.433	1062970	24720489	50.33

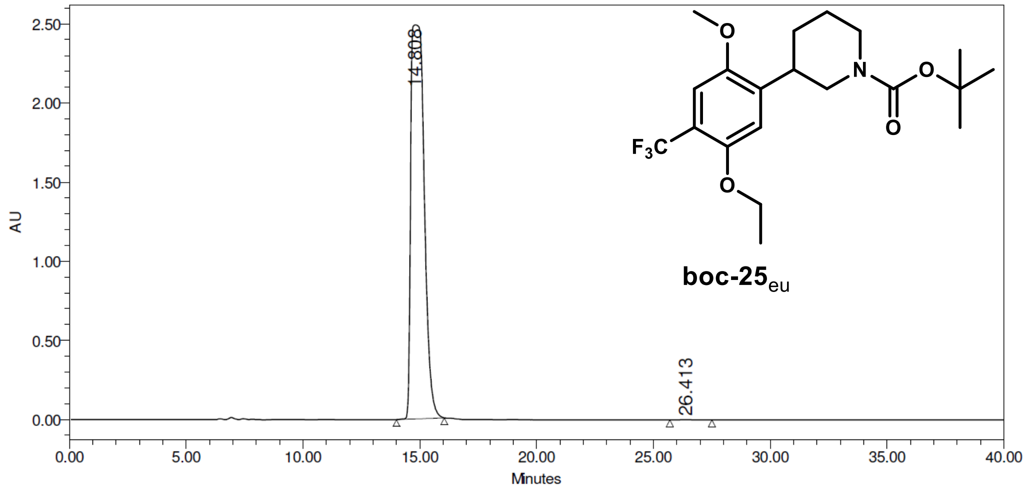

	RT	Height	Area	% Area
1	14.808	2489964	99660902	99.96
2	26.413	875	43174	0.04

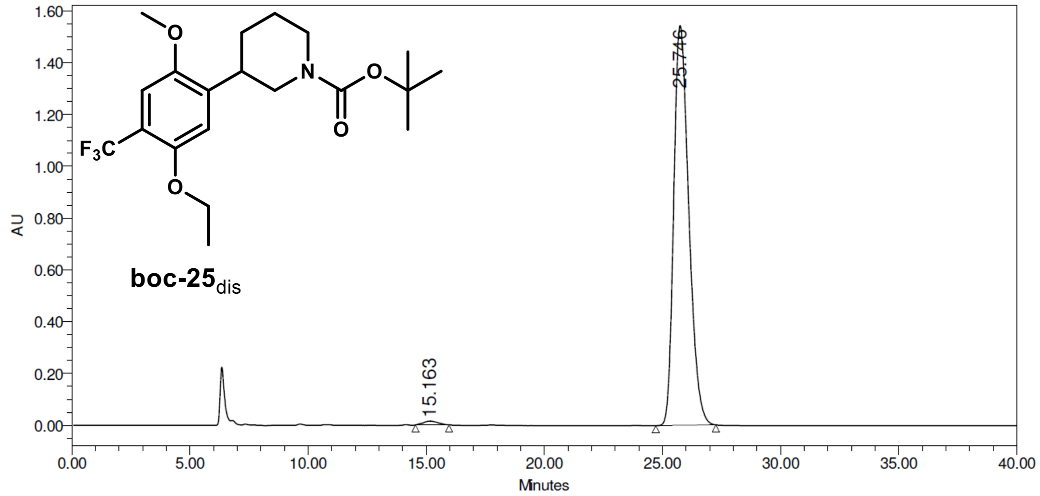

	RT	Height	Area	% Area
1	15.163	14657	635046	0.92
2	25.746	1543404	68239017	99.08

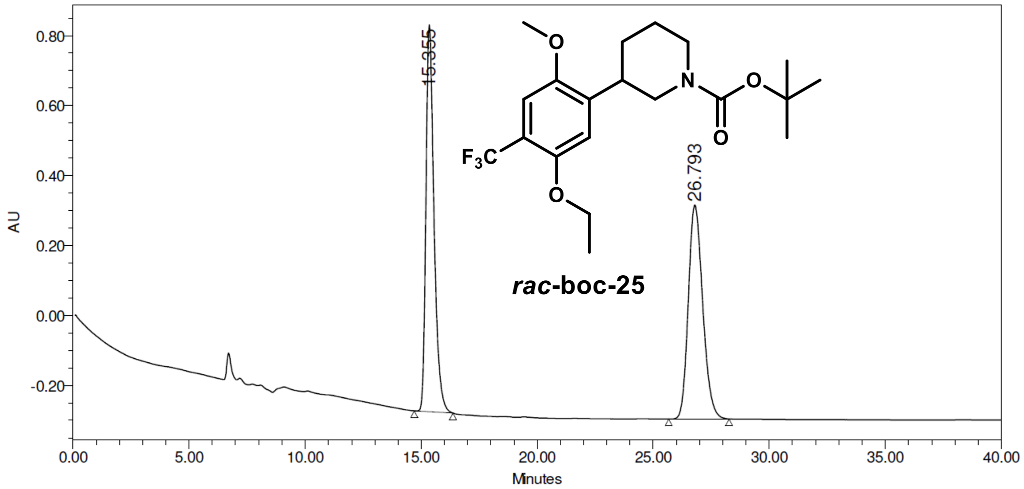

	RT	Height	Area	% Area
1	15.355	1105992	26876798	50.58
2	26.793	611312	26264078	49.42

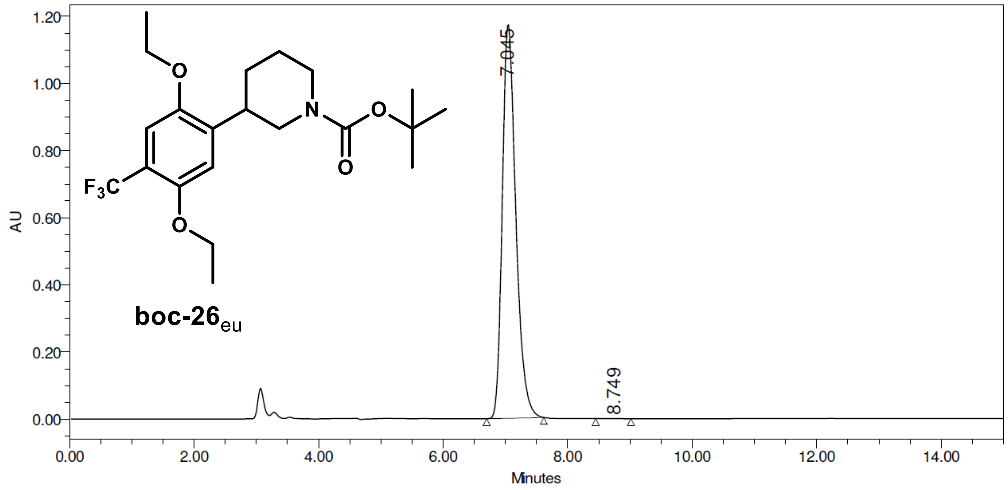

	RT	Height	Area	% Area
1	7.045	1173862	17717338	99.98
2	8.749	203	3443	0.02

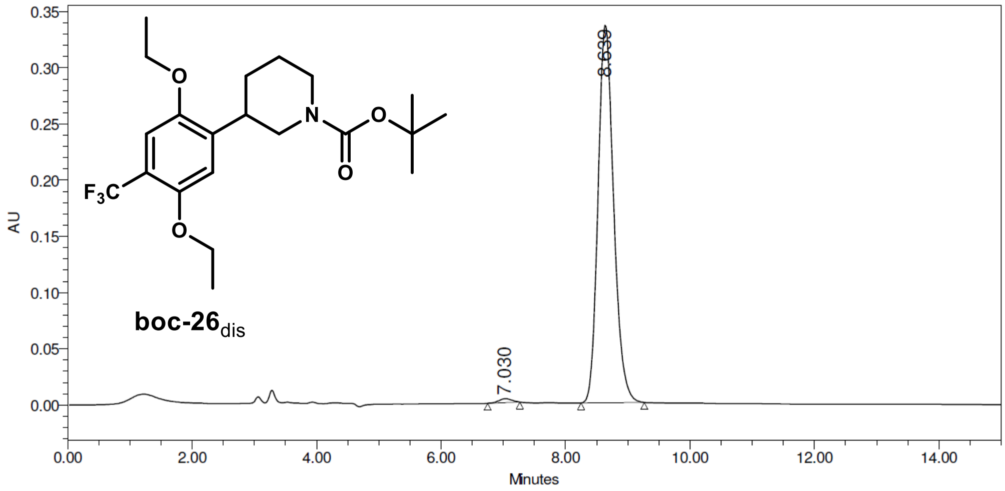

	RT	Height	Area	% Area
1	7.030	3494	49304	0.83
2	8.639	336258	5856931	99.17

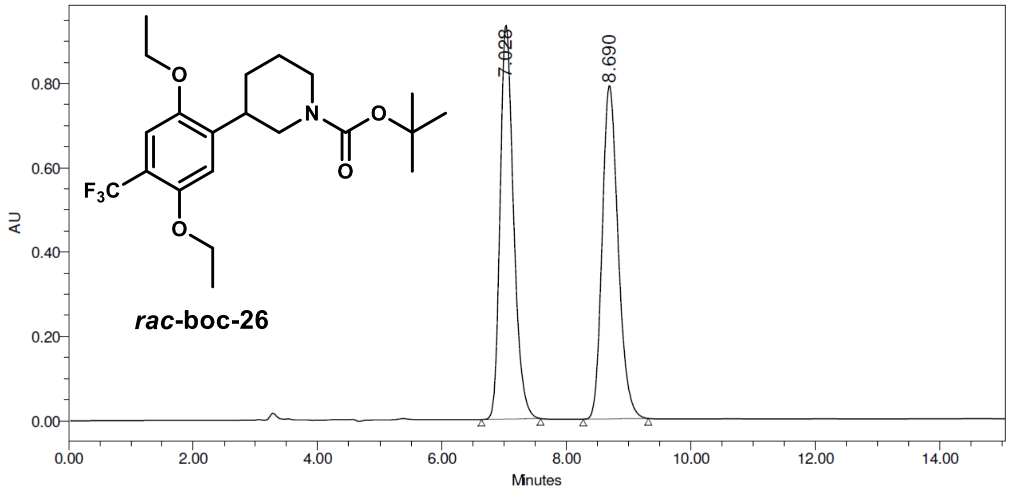

	RT	Height	Area	% Area
1	7.028	935124	14098422	49.98
2	8.690	791404	14110603	50.02

X-Ray Crystallography Experimental Details

X-ray Crystallographic Analysis of Compound *11 ent 2* ((*R*)-11) as salt with (2*S,3S*)-tartaric acid.

Single crystals suitable for X-ray diffraction studies were grown from a solution in methanol. A single crystal was mounted and immersed in a stream of nitrogen gas [T = 123(1) K]. Data were collected, using graphite-monochromated Mo$K\alpha$ radiation (λ = 0.71073 Å) on a Bruker D8 Venture diffractometer. Data collection and cell refinement were performed using the Bruker Apex2 Suite software.[1] Data reduction using SAINT and multi-scan correction for absorption using SADABS-2016-2 were performed within the Apex2 Suite.[2, 3] The crystal data, data collection and the refinement data are given in Table S1.

Structure Solution and Refinement of Compound *11 ent 2* ((*R*)-11) as salt with (*S,S*)-tartaric acid.

Positions of all non-hydrogen atoms were found by direct methods (SHELXS97).[4] Full-matrix least-squares refinements (SHELXL) were performed on F^2, minimizing $\Sigma w(F_o^2 - kF_c^2)^2$, with anisotropic displacement parameters of the non-hydrogen atoms.[5] The positions of hydrogen atoms were located in subsequent difference electron density maps and were included in calculated position with fixed isotropic displacement parameters (U_{iso} = 1.2U_{eq} for CH, CH_2 and U_{iso} = 1.5U_{eq} for CH_3), except for hydrogen atoms connected to chiral C, O or N atoms. These hydrogen atoms were refined with fixed isotropic displacement parameters (U_{iso} = 1.2U_{eq} for CH and NH_2; U_{iso} = 1.5U_{eq} for OH).

Refinement (606 parameters, 11628 unique reflections) converged at R_F = 0.0318, wR_F^2 = 0.0777 [10809 reflections with Fo > 4$\sigma(F_o)$; $w^{-1} = (\sigma^2(F_o^2) + (0.0473P)^2 + 0.1249P)$, where $P = (F_o^2 + 2F_c^2)/3$; S = 1.038]. The residual electron density varied between -0.26 and 0.31 e Å^{-3}. Non-centrosymmetric space group is assigned and the absolute configuration can be determined relative to the already known configuration of the (2*S,3S*)-tartaric acid.

Complex scattering factors for neutral atoms were taken from International Tables for Crystallography as incorporated in SHELXL.[6] Fractional atomic coordinates, a list of anisotropic displacement parameters, and a complete list of geometrical data has been deposited in the Cambridge Crystallographic Data Centre (CCDC 2321050).

Figure S1: Perspective drawing of Compound 11, enantiomer 2, (*R*)-11.

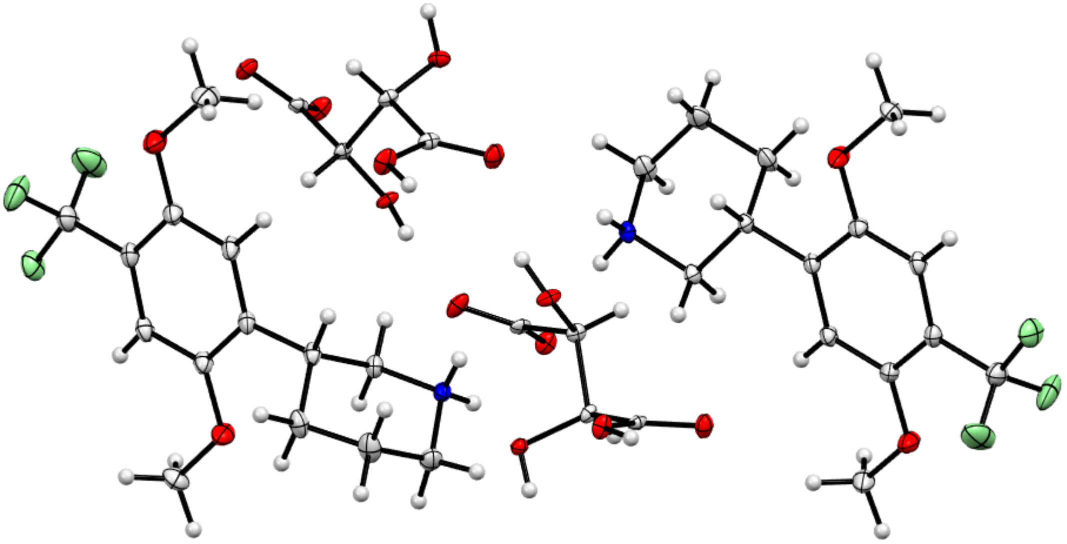

Figure. S1: Perspective drawing of **Compound *11 ent 2* ((*R*)-11) as salt with (2*S,3S*)-tartaric acid**. [7] Displacement ellipsoids of the non-hydrogen atoms are shown at the 50% probability level. Hydrogen atoms have been shown as spheres of arbitrary size. Nitrogen atoms are blue, fluor atoms green and oxygen atoms red.

Table S1: Crystal Data for Compound (*R*)-11.

Crystal data, data collection and refinement data for Compound *R*)-11 as salt with (2*S,3S*)-tartaric acid.

Formula:	$C_{14}H_{19}NO_2F_3$; $C_4H_5O_6$
Mw (g/mol)	439.38
Temperature, K	122(2)
Crystal class	triclinic
Space group	*P*1
Cell parameters	a = 7.642(5) Å
	b = 8.652(4) Å
	c = 14.953(7) Å
	α = 84.257(17)°
	β = 84.80(2)°
	γ = 87.837(19)°
V (Å^3), Z	979.2(9) / 2
F(000)	460
d_{calc} (g/cm3)	1.490
λ	0.71073 Å (Mo$K\alpha$)
θ_{max} (°)	2.37 < θ < 30.58
Reflections total/unique	71215/11628
R_{merge}	0.0400
No. parameters/restraints	606/3
Reflections ($I > 2\ \sigma(I)$)	10809
R1/wR2 ($I > 2\ \sigma(I)$)	0.0318/0.0777
R1/wR2 (all reflections)	0.0367/0.0804
Goodness-of-fit on F^2	1.038
ρ_{max} / ρ_{min} (eÅ^{-3})	0.31 / -0.26
Flack parameter	0.0(4)

Table S2. Extended Functional Properties for Compounds 1 & 4-26 at 5-HT_{2A}R- and 5-HT_{2C}R.

Table S2. Functional properties exhibited by all analogs at stable 5-HT_{2A}R- and 5-HT_{2C}R-HEK293 cell lines in the Ca^{2+}/Fluo-4 assay. EC_{50} values are given in nM with pEC_{50} ± S.E.M. values in brackets, and R_{max} values are given as % of the 5-HT R_{max} at the respective receptors determined on the same 96-well plate. For the compounds also tested in antagonist mode at 5-HT_{2C}R (using 5-HT EC_{90} as agonist), IC_{50} values are given in nM with pIC_{50} ± S.E.M. values in brackets. The numbers of independent experiments (n) performed in duplicate underlying the data are given in superscript after the data.

	**5-HT_{2A}R**		**5-HT_{2C}R**		
	**EC_{50} [pEC_{50} ± S.E.M.]**	**R_{max} ± S.E.M. $^{(n)}$**	**EC_{50} [pEC_{50} ± S.E.M.]**	**R_{max} ± S.E.M. $^{(n)}$**	**IC_{50} [pIC_{50} ± S.E.M.] $^{(n)}$**
5-HT	7.3 [8.14 ± 0.02]	100 $^{(15)}$	2.6 [8.58 ± 0.03]	100 $^{(14)}$	
**1** (2C-B) [a]	1.6 [8.79 ± 0.08]	68 ± 6 $^{(4)}$	4.1 [8.36 ± 0.02]	74 ± 3 $^{(4)}$	
**4**	1.6 [8.80 ± 0.10]	97 ± 3 $^{(4)}$	5.8 [8.24 ± 0.07]	90 ± 3 $^{(4)}$	
**5**$_{eu}$	5.3 [8.28 ± 0.07]	57 ± 3 $^{(4)}$	26 [7.59 ± 0.03]	73 ± 3 $^{(3)}$	
**5**$_{dis}$	7.7 [8.11 ± 0.08]	39 ± 4 $^{(4)}$	18 [7.75 ± 0.08]	16 ± 3 $^{(3)}$	
**6** $_{eu}$	69 [7.16 ± 0.10]	37 ± 4 $^{(6)}$	n.a. [b]	n.d. $^{(5)}$	640 [6.20 ± 0.03] $^{(5)}$
**6** $_{dis}$	370 [6.43 ± 0.11]	67 ± 6 $^{(4)}$	1,900 [5.72 ± 0.04]	34 ± 4 $^{(5)}$	
**7**	w.a. [@ 2-10-50 μM] [c]	n.d. $^{(3)}$	w.a. [@ 10-50 μM] [c]	n.d. $^{(3)}$	
**8**$_{eu}$	66 [7.18 ± 0.05]	32 ± 6 $^{(5)}$	n.a. [b]	n.d. $^{(5)}$	860 [6.06 ± 0.04] $^{(5)}$

**8**$_{dis}$	690 [6.16 ± 0.11]	61 ± 2 (5)	1,800 [5.75 ± 0.04]	26 ± 2 (5)	
**9**$_{eu}$	37 [7.43 ± 0.02]	53 ± 3 (4)	n.a. [b]	n.d. (5)	1,100 [5.96 ± 0.10] (5)
**9**$_{dis}$	260 [6.58 ± 0.03]	58 ± 4 (5)	2,800 [5.55 ± 0.08]	20 ± 2 (6)	
**10**$_{eu}$	270 [6.56 ± 0.13]	25 ± 4 (5)	n.a. [b]	n.d. (5)	~15,000 [~4.8] (5) [d]
**10**$_{dis}$	2,200 [5.66 ± 0.06]	41 ± 6 (4)	~10,000 [~5.0] [c]	14 ± 1 (5)	
(*S*)-**11**	3.2 [8.49 ± 0.11]	78 ± 5 (6)	n.a. [b]	n.d. (6)	320 [6.50 ± 0.07] (5)
(*R*)-**11**	150 [6.82 ± 0.04]	65 ± 5 (6)	860 [6.07 ± 0.06]	26 ± 2 (5)	
**12**$_{eu}$	100 [6.98 ± 0.10]	75 ± 3 (4)	w.a. [@ 10-50 μM] [c]	n.d. (3)	
**12**$_{dis}$	260 [6.58 ± 0.06]	89 ± 4 (4)	1,200 [5.92 ± 0.08]	53 ± 6 (4)	
**13**$_{eu}$	41 [7.39 ± 0.11]	75 ± 4 (4)	380 [6.42 ± 0.12]	20 ± 4 (4)	
**13**$_{dis}$	110 [6.94 ± 0.09]	93 ± 5 (4)	640 [6.19 ± 0.12]	44 ± 5 (4)	
**14**$_{eu}$	270 [6.57 ± 0.06]	82 ± 2 (3)	w.a. [@ 2-10-50 μM] [c]	n.d. (4)	
**14** $_{dis}$	340 [6.46 ± 0.05]	93 ± 5 (3)	1,600 [5.80 ± 0.07]	68 ± 6 (4)	
**15**$_{eu}$	26 [7.59 ± 0.11]	84 ± 6 (4)	510 [6.29 ± 0.06]	16 ± 2 (4)	
**15**$_{dis}$	180 [6.75 ± 0.06]	85 ± 7 (4)	2,300 [5.63 ± 0.03]	38 ± 5 (4)	
**16**$_{eu}$	38 [7.42 ± 0.09]	80 ± 6 (4)	570 [6.25 ± 0.08]	8 ± 2 (3)	
**16**$_{dis}$	190 [6.73 ± 0.10]	83 ± 6 (4)	780 [6.11 ± 0.08]	52 ± 5 (4)	
**17**$_{eu}$	160 [6.81 ± 0.09]	92 ± 4 (3)	w.a. [@ 2-10-50 μM] [c]	n.d. (4)	
**17**$_{dis}$	290 [6.54 ± 0.09]	91 ± 4 (3)	2,300 [5.64 ± 0.05]	50 ± 1 (3)	
**18** (2C-TFM)	0.72 [9.14 ± 0.08]	67 ± 5 (4)	7.6 [8.12 ± 0.04]	71 ± 2 (5)	
(*S*)-**19**	390 [6.40 ± 0.07]	19 ± 2 (3)	920 [6.04 ± 0.03]	14 ± 2 (3)	
(*R*)-**19**	1,100 [5.96 ± 0.09]	53 ± 8 (4)	w.a. [@ 2-10-50 μM] [c]	n.d. (3)	
(*S*)-**20**	1,500 [5.82 ± 0.08]	44 ± 3 (4)	w.a. [@ 10-50 μM] [c]	n.d. (3)	

(*R*)-**20**	w.a. [@ 2-10-50 μM] [c]	n.d. (3)	w.a. [@ 2-10-50 μM] [c]	n.d. (3)
**21**	w.a. [@ 2-10-50 μM] [c]	n.d. (3)	w.a. [@ 2-10-50 μM] [c]	n.d. (3)
**22**$_{eu}$	69 [7.16 ± 0.05]	68 ± 2 (3)	w.a. [@ 2-10-50 μM] [c]	n.d. (4)
**22**$_{dis}$	660 [6.18 ± 0.07]	80 ± 5 (4)	1,400 [5.85 ± 0.05]	38 ± 6 (3)
**23**$_{eu}$	1,800 [5.74 ± 0.09]	68 ± 7 (3)	w.a. [@ 50 μM] [c]	n.d. (3)
**23**$_{dis}$	2,500 [5.61 ± 0.07]	65 ± 3 (4)	1,400 [5.85 ± 0.13]	35 ± 3 (4)
**24**$_{eu}$	38 [7.42 ± 0.10]	40 ± 3 (4)	w.a. [@ 50 μM] [c]	n.d. (3)
**24**$_{dis}$	410 [6.39 ± 0.08]	77 ± 7 (3)	2,700 [5.56 ± 0.02]	34 ± 1 (3)
**25**$_{eu}$	10 [7.99 ± 0.11]	95 ± 1 (3)	290 [6.54 ± 0.04]	44 ± 5 (4)
**25**$_{dis}$	320 [6.50 ± 0.10]	86 ± 6 (3)	w.a. [@ 2-10-50 μM] [c]	n.d. (3)
**26**$_{eu}$	100 [6.99 ± 0.07]	47 ± 6 (4)	w.a. [@ 10-50 μM] [c]	n.d. (3)
**26**$_{dis}$	3,000 [5.52 ± 0.06]	69 ± 4 (4)	w.a. [@ 10-50 μM] [c]	n.d. (3)

---

[a] Data for **1** (2C-B) has been published previously.[8] n.a., no agonist activity. The compound either did not display significant agonist activity or displayed negligible levels of agonist activity at concentrations up to 50 μM at 5-HT_{2C}R. Thus, the EC_{50} and R_{max} could not be determined. [c] w.a., weak agonist activity. The compound displayed weak but significant agonist activity at the indicated concentrations. The agonist concentration-response relationship exhibited by the compound was not saturated at 50 μM, and thus the EC_{50} and R_{max} could not be determined. [d] The concentration-inhibition relationship exhibited by the compound when tested in antagonist mode was not complete at 50 μM. The mean IC_{50} value is an approximate value estimated based on the fitted curves. n.d., not determinable. The R_{max} value for this compound could not be determined since a complete concentration-response curve was not obtained in the tested concentration range (up to 50 μM).

Figure S2: Binding Properties Exhibited by (*S*)-11 at Human 5-HT$_{2A}$R, 5-HT$_{2B}$R, 5-HT$_{2C}$R.

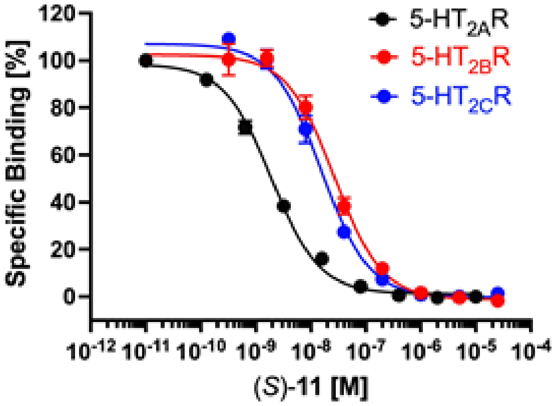

Figure S2. Binding properties exhibited by (*S*)-**11** at human 5-HT$_{2A}$R, 5-HT$_{2B}$R, 5-HT$_{2C}$R expressed in mammalian cell lines in a [^{125}I]DOI competition binding assay using 0.1 nM [^{125}I]DOI as radioligand concentration. Data are given as mean ± S.E.M. values based on three independent determinations performed in duplicate. Full experimental details are included in the manuscript text and the specific radioligand binding assays are described at Eurofins website (www.eurofinsdiscoveryservices.com).

Table S3: Binding Properties Exhibited by (*S*)-11 at Human 5-HT_{2A}R, 5-HT_{2B}R, 5-HT_{2C}R.

[^{125}I]DOI competition binding assay

K_i [pK_i ± S.E.M.] $^{(n)}$

	**5-HT_{2A}R**	**5-HT_{2B}R**	**5-HT_{2C}R**
(±)-DOI	0.34 [9.47 ± 0.08] $^{(5)}$	1.2 [8.91 ± 0.09] $^{(3)}$	0.51 [9.29 ± 0.08] $^{(4)}$
(*S*)-**11**	1.3 [8.87 ± 0.06] $^{(3)}$	13 [7.88 ± 0.07] $^{(3)}$	13 [7.89 ± 0.10] $^{(3)}$

Table S3. Binding properties exhibited by (*S*)-**11** at human 5-HT_{2A}R, 5-HT_{2B}R, 5-HT_{2C}R expressed in mammalian cell lines in a [^{125}I]DOI competition binding assay. The binding properties of (±)-DOI is given for reference. K_i values are given in nM with pK_i ± S.E.M. values in brackets, and the numbers of experiments (n) (all perfomed in duplicate) are given in superscript. Full experimental details are included in the manuscript text and the specific radioligand binding assays are described at Eurofins website (www.eurofinsdiscoveryservices.com).

Table S4: Off-target screen of (*S*)-11.

Table S4. Pharmacological profile exhibited by (*S*)-**11** in radioligand binding competition assays at various targets expressed in radioligand competition binding assays. The testing was performed using concentrations of the respective radioligands close to their respective K_D values. Unless otherwise indicated, the compounds have been tested at the human targets. The specific radioligand binding assays are described at Eurofins website (www.eurofinsdiscoveryservices.com).

First part of the table: Binding properties of (*S*)-**11** (0.1 µM, 1 µM, 10 µM) at various serotonergic, adrenergic, dopaminergic, histaminergic and muscarinic receptors and at the three monoaminergic transporters are based on two independent determinations (n=2) performed in duplicate. The average inhibition observed for 0.1 µM, 1 µM and/or 10 µM (*S*)-**11** is given as % inhibition of the specific radioligand binding, and the IC_{50} value or IC_{50} range estimated for (*S*)-**11** based on the data is given in µM.

Second part of the table: Binding properties of (*S*)-**11** (10 µM) at various targets based on a single determination (n=1) performed in duplicate. The average inhibition observed for 10 µM (*S*)-**11** is given as % inhibition of the specific radioligand binding, and the IC_{50} value or IC_{50} range estimated for (*S*)-**11** based on the data is given in µM.

Target	Radioligand	*0.1 µM [%]*	*1 µM [%]*	*10 µM [%]*	IC_{50} [µM]
5-HT_{1A}	[^{3}H]8-OH-DPAT	*20*	*64*	*90*	0.1-1
5-HT_{1B}	[^{3}H]GR125743	-	*21*	*68*	1-10
5-HT_{1D} [a]	[^{3}H]serotonin	*18*	*64*	*93*	0.1-1
5-HT_3	[^{3}H]BRL 43694	–	–	*6*	>10
5-HT_{4e}	[^{3}H]GR 113808	–	–	*30*	>10
5-HT_{5a}	[^{3}H]LSD	–	–	*4*	>10
5-HT_6	[^{3}H]LSD	–	*24*	*77*	1-10
5-HT_7	[^{3}H]LSD	–	*17*	*62*	1-10
α_{1A}	[^{3}H]Prazosin	–	–	*32*	>10
α_{1D}	[^{3}H]Prazosin	–	–	*27*	>10
α_{2A}	[^{3}H]RX 821002	–	*1*	*34*	>10
α_{2C}	[^{3}H]RX 821002	–	–	*36*	>10

β_1	[^{3}H](-)CGP 12177	–	–	*38*	>10
β_2	[^{3}H](-)CGP 12177	–	*2*	*55*	~10
D_1	[^{3}H]SCH 23390	–	–	*30*	>10
D_{2S}	[^{3}H]7-OH-DPAT	–	–	*59*	~10
D_3	[^{3}H]methyl-spiperone	–	–	*40*	~10
$D_{4.4}$	[^{3}H]methyl-spiperone	–	–	*9*	>10
D_5	[^{3}H]SCH 23390	–	–	*26*	>10
H_1	[^{3}H]pyrilamine	–	*19*	*64*	1-10
H_2	[^{125}I]APT	–	–	*10*	>10
H_3	[^{3}H]*N*-α-methyl-histamine		–	*11*	>10
M_1	[^{3}H]pirenzepine	–	*51*	*90*	~1
M_2	[^{3}H]AF-DX 384	–	–	*40*	~10
M_3	[^{3}H]4-DAMP	–	*41*	*90*	~1
M_4	[^{3}H]4-DAMP	9	*38*	*80*	1-10
M_5	[^{3}H]4-DAMP	9	*57*	*92*	~1
SERT	[^{3}H]imipramine	–	–	*22*	>10
NET	[^{3}H]nisoxetine	–	–	*-4*	>10
DAT	[^{3}H]BTCP	–	–	*12*	>10

**Target**	**Radioligand**	***10 μM [%]***	**IC_{50} [μM]**
Adenosine receptor A_1	[^{3}H]DPCPX	*4*	>10
Adenosine receptor A_{2A}	[^{3}H]CGS-21680	*-19*	>10
Adenosine receptor A_3	[^{125}I]AB-MECA	*-10*	>10
Adrenergic receptor α_{1B}	[^{3}H]Prazosin	*33*	>10
Androgen receptor (NHR)	[^{3}H]Methyltrienolone	*1*	>10
Bradykinin receptor B_1	[^{3}H](Des-Arg10, Leu9)-Kallidin	*12*	>10
Bradykinin receptor B_2	[^{3}H]Bradykinin	*18*	>10
Ca^{2+} Channel L-Type, (Benzothiazepine) [a]	[^{3}H]Diltiazem	*66*	1-10
Ca^{2+} Channel L-Type, (Dihydropyridine) [a]	[^{3}H]Nitrendipine	*-6*	>10
Ca^{2+} Channel N-Type [a]	[^{125}I]ω-conotoxin GVIA	*-5*	>10

Cannabinoid receptor CB_1	[^{3}H]SR141716A	*14*	>10
Endothelin receptor ET_A	[^{125}I]Endothelin-1	*12*	>10
Endothelin receptor ET_B	[^{125}I]Endothelin-1	*-6*	>10
Epidermal Growth Factor receptor	[^{125}I]EGF	*-18*	>10
Estrogen receptor ERα	[^{3}H]Estradiol	*-2*	>10
$GABA_A$ receptor (GABA) [a]	[^{3}H]Muscimol	*1*	>10
$GABA_A$ receptor (benzod.) [a]	[^{3}H]Flunitrazepam	*-14*	>10
$GABA_{B(1a,2)}$ receptor	[^{3}H]CGP-54626	*6*	>10
Glucocorticoid receptor	[^{3}H]Dexamethasone	*11*	>10
Kainate receptor [a]	[^{3}H]Kainic acid	*9*	>10
NMDA receptor (Glutamate)[a]	[^{3}H]CGP-39653	*-8*	>10
NMDA receptor (Glycine)[a]	[^{3}H]MDL 105,519	*-20*	>10
NMDA receptor (PCP) [a]	[^{3}H]TCP	*4*	>10
Imidazoline receptor I_2 [a]	[^{3}H]Idazoxan	*17*	>10
Leukotriene receptor $CysLT_1$	[^{125}I]LTD4	*-7*	>10
Melatonin receptor MT_1	[^{125}I]2-Iodomelatonin	*7*	>10
Neuropeptide Y receptor Y_1	[^{125}I]Peptide YY	*-4*	>10
Neuropeptide Y receptor Y_2	[^{125}I]Peptide YY	3	>10
Nicotinic acetylcholine receptor (muscle-type)	[^{125}I]α-Bungarotoxin	*3*	>10
Nicotinic acetylcholine receptor α3β4	[^{125}I]Epibatidine	51	~10
Opioid receptor δ	[^{3}H] Naltrindole	*18*	>10
Opioid receptor κ	[^{3}H]Diprenorphine	*18*	>10
Opioid receptor μ	[^{3}H]Diprenorphine	*18*	>10
Phorbol ester [b]	[^{3}H]PDBu	*15*	>10
Platelet Activating Factor Receptor	[^{3}H]PAF	*-11*	>10
K^+ Channel (K_{ATP}) [c]	[^{3}H]Glyburide	*-2*	>10
K^+ Channel (hERG)	[^{3}H]Astemizole	*32*	>10
Prostanoid receptor EP_4	[^{3}H]Prostaglandin E2	*9*	>10
Purinergic receptor P2X [a]	[^{3}H]α,β-Methylene-ATP	*-30*	>10

Purinergic receptor P2Y [a]	$[^{35}S]dATP\alpha S$	*2*	>10
Phosphodiesterase [a]	$[^{3}H]$Rolipram	*8*	>10
Sigma receptor σ_1	$[^{3}H]$Pentazocine	*84*	1-10
Na^+ Channel (Site 2) [a]	$[^{3}H]$Batrachotoxinin	*86*	1-10
Tachykinin receptor NK_1	$[^{3}H]$Substance P	*31*	>10
Tachykinin receptor NK_2	$[^{125}I]$Neurokinin A	*1*	>10
Tachykinin receptor NK_3	$[^{125}I]MePhe^{7}$-Neurokinin B	*14*	>10
Thyroid hormone receptor [a]	$[^{125}I]$Triiodothyronine	*-1*	>10
GABA transporter [a]	$[^{3}H]$GABA	*29*	>10

Targets of other species than human: [a] rat, [b]mouse, [c]hamster.

Table S5: LogP determination of (*S*)-11.

**LogP**	Log D (pH 1.8)	log D (pH2.5)	log D (pH4.5)	log D (pH7.4)	log D (pH9.5)	log D (pH11.0)	log D (pH12.0)
**3,45**	-0.815	-0.536	-0.376	0.659	1.99	3.40	3.50

Phosphate buffer (PB) with the pH ranging from 1.8 to 12.0 and Octanol saturated with water was used. 2 μL of 10 mM DMSO stock solution of test and control compounds was aliquoted into tubes in duplicate. Added 149 μL of PB (pH 1.8, 2.5, 4.5, 7.4, 9.5,11.0 and 12.0)-Saturated with 1-Octanol and 149 μL of 1-Octanol Saturated PB (pH 1.8, 2.5, 4.5, 7.4, 9.5,11.0 and 12.0) was added, respectively. Each of the tubes was vigorously mixed for 2 minutes and then shook for 1 hour at a speed of 800 rpm at room temperature. Following centrifugation at 4000 rpm for 5 minutes at room temperature, appropriate volume of buffer layer samples and 1-octanol layer samples was aliquoted. These samples were diluted and detected by using LC-MS/MS method without running a calibration curve.

The log D values were calculated with the following equation:

$$Log\ D_{octanol/buffer} = \log(\frac{Mean\ Octanol\ Layer\ Peak\ Area\ Ratio\ *\ Octanol\ Layer\ Dilution\ Factor}{Mean\ Buffer\ Layer\ Peak\ Area\ Ratio\ *\ Buffer\ Layer\ Dilution\ Factor}$$

Table S6: MDR1-MDCK II permeability assay.

Compound	Mean P_{app} (10^{-6} cm/s)		Efflux Ratio	Mean Recovery		Rank
	A to B	B to A		A to B	B to A	P_{app}
Nadolol	0.54	ND	ND	93 %	ND	Low
Metoprolol	24.22	ND	ND	97 %	ND	High
Digoxin	0.22	12.36	56	93 %	92 %	Low
(*S*)-**11**	18.36	17.30	0.94	70 %	92 %	High

Test compounds were diluted with the transport buffer (HBSS with 10mM Hepes, pH7.4) from DMSO stock solution to a concentration of 2 µM (DMSO<1%) and applied to the apical or basolateral side of the cell monolayer. Permeation of the test compounds from A to B direction or B to A direction was determined in duplicate. Digoxin was tested at 10 µM from A to B direction or B to A direction as well, while nadolol and metoprolol were tested at 2 µM in A to B direction in duplicate. The plate was incubated for 2.5 hours in CO_2 incubator at 37±1°C, with 5% CO_2 at saturated humidity without shaking. In addition, the efflux ratio of each compound was also determined. Test and reference compounds were quantified by LC-MS/MS analysis based on the peak area ratio of analyte/IS.

After transport assay, Lucifer yellow rejection assay are applied to determine the cell monolayer integrity. Buffers are removed from both apical and basolateral chambers, followed by the addition of 75 µL of 100 µM lucifer yellow in transport buffer and 250 µL transport buffer in apical and basolateral chambers, respectively. The plate is incubated for 30 minutes at 37°C with 5% CO_2 and saturated humidity without shaking. After 30 minutes incubation, 20 µL of lucifer yellow samples are taken from the apical sides, followed by the addition of 60 µL of Transport Buffer. And then 80 µL of lucifer yellow samples are taken from the basolateral sides. The relative fluorescence unit (RFU) of lucifer yellow is measured at 425/528 nm (excitation/emission) with an Envision plate reader.

The apparent permeability coefficient Papp (cm/s) was calculated using the equation:

$P_{app} = (dC_r/dt) \times V_r / (A \times C_0)$

Where dC_r/d_t is the cumulative concentration of compound in the receiver chamber as a function of time (µM/s); V_r is the solution volume in the receiver chamber (0.075 mL on the apical side, 0.25 mL on the basolateral side); A is the surface area for the transport, i.e. 0.0804 cm^2 for the area of the monolayer; C_0 is the initial concentration in the donor chamber (µM).

References

(1) *Apex2 Suite*; Madison, Wisconsin, USA., **2013**.
(2) *SAINT Version 8.37A*; Bruker AXS Inc.: Madison, Wisconsin, USA., **2016**.
(3) *SADABS-2016-2*; Bruker AXS Inc.: Madison, Wisconsin, USA., **2016**.
(4) Sheldrick, G. A short history of SHELX. *Acta Crystallographica Section A* **2008**, *64* (1), 112-122.
(5) Sheldrick, G. Crystal structure refinement with SHELXL. *Acta Crystallographica Section C* **2015**, *71* (1), 3-8.
(6) Wilson, A. J. C., Ed. . International Tables for Crystallography. In *I*, Kluwer Academic Publishers: Dordrecht, The Netherlands, **2006**; Vol. Volume C, p Tables 4.2.6.8 and 6.1.1.4. .
(7) Macrae, C. F.; Sovago, I.; Cottrell, S. J.; Galek, P. T. A.; McCabe, P.; Pidcock, E.; Platings, M.; Shields, G. P.; Stevens, J. S.; Towler, M.; et al. Mercury 4.0: from visualization to analysis, design and prediction. *Journal of Applied Crystallography* **2020**, *53* (1), 226-235.
(8) Marcher-Rørsted, E.; Halberstadt, A. L.; Klein, A. K.; Chatha, M.; Jademyr, S.; Jensen, A. A.; Kristensen, J. L. Investigation of the 2,5-Dimethoxy Motif in Phenethylamine Serotonin 2A Receptor Agonists. *ACS Chemical Neuroscience* **2020**, *11* (9), 1238-1244.
